# Total Synthesis of the Reported Structure of Cahuitamycin A: Insights into an Elusive Natural Product Scaffold

**DOI:** 10.1021/acs.orglett.3c03993

**Published:** 2023-12-18

**Authors:** Justin A. Shapiro, Savannah J. Post, Gavin C. Smith, William M. Wuest

**Affiliations:** † Department of Chemistry, Emory University, Atlanta, Georgia 30322, United States; ‡ Emory Antibiotic Resistance Center, Emory University, Atlanta, Georgia 30322, United States

## Abstract

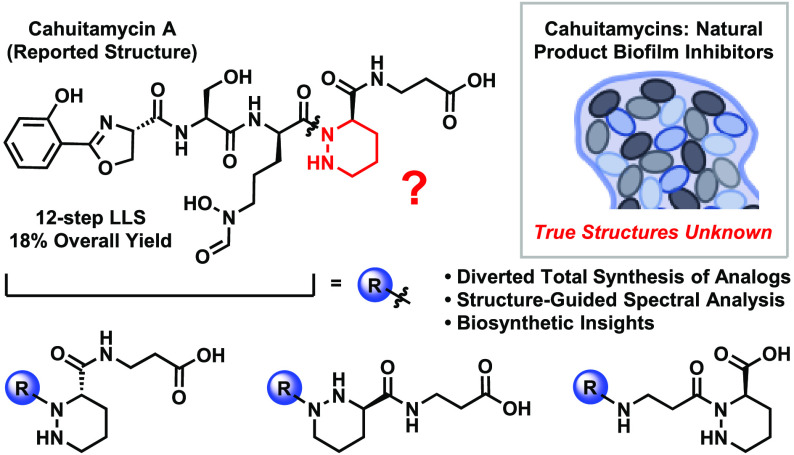

In a 2016 screen of natural product extracts, a new family of natural products, the cahuitamycins, was discovered and found to inhibit biofilm formation in the human pathogen *Acinetobacter baumannii*. The proposed molecular structures contained an unusual piperazic acid residue, which piqued interest related to their structure/function and biosynthesis. Herein we disclose the first total synthesis of the proposed structure of cahuitamycin A in a 12-step longest linear sequence and 18% overall yield. Comparison of spectral and biological data of the authentic natural product and synthetic compound revealed inconsistentancies with the isolated metabolite. We therefore executed the diverted total synthesis of three isomeric compounds, which were also found to be disparate from the isolated natural product. This work sets the stage for future synthetic and biochemical investigations of an important class of natural products.

Multidrug resistant bacterial infections represent a global health crisis, as outlined by the Centers for Disease Control and Prevention threat report on antibiotic resistance.^1^ Particularly troubling are resistant *Acinetobacter baumannii*, which were upgraded from “serious threat” to “urgent threat” in 2019 due to the lack of antibiotics in the clinic or pipeline to treat infections.^2^*A. baumannii* is an opportunistic pathogen commonly found in lung, wound, bloodstream, and urinary tract infections among immune-compromised patients and those in hospitals or military treatment facilities.^3^ Isolates are routinely found with resistance to drugs of last resort such as the carbapenems and polymyxins.^4^ With effective treatment options dwindling, new drugs with a novel phenotype are desperately needed to combat this increasing threat.

An important method by which *A. baumannii* evades treatment is by the formation of biofilms,^5^ communities of bacteria bound together by extracellular matrices which display enhanced tolerance to antibiotics^6^ and are known to lay dormant on medical equipment.^7^ In a natural product extract library screen followed by extensive ribosomal engineering, Sherman and co-workers identified a novel group of natural products from *Streptomyces gandocaensis* called the cahuitamycins^8^ (Figure 1A). These compounds were shown to selectively inhibit the formation of *A. baumannii* biofilms with minimal effects on planktonic growth. The structures of the cahuitamycins were reported as highly functionalized nonribosomal hexapeptides that originate from a single biosynthetic gene cluster. Among the interesting features of cahuitamycin A (**1**) are the *o*-phenolate oxazoline and the terminal hydroxamate, both strong iron-chelating moieties found in *A. baumannii* siderophores such as preacinetobactin^9−12^ (**6**) and fimsbactin A^13−15^ (**7**) (Figure 1B), and the piperazic acid,^16,17^ a rare amino acid residue found in highly bioactive and structurally diverse natural products such as kutzneride 1^18^ (**10**) and sanglifehrin A^19^ (**11**) (Figure 1D). By recognizing that cahuitamycin C (**2**) is derived from an off-cluster starting material, the authors were able to generate mutasynthetic analogs via feeding experiments, resulting in cahuitamycins D (**3**) and F^20^ (**4**), as well as cahuitamycin E (**5**) which contains an internal *N*-hydroxy-ornithine in place of the piperazic acid. Intriguingly, a recent report describes sequence homology between the NRPS modules for cahuitamycin biosynthesis and those of two structural isomers, the new natural product attinimicin (**8**)^21^ and the known siderophore oxachelin A (**9**)^22^ (Figure 1C). While the cahuitamycins are hypothesized by the isolation report to derive from a transformation of a *N-*hydroxy-ornithine moiety into a piperazic acid, attinamycin and oxachelin are cleaved from the assembly line via an intramolecular lactamization of a C-terminal *N*-hydroxy-ornithine residue to form a 6-membered hydroxamate.

**Figure 1:**
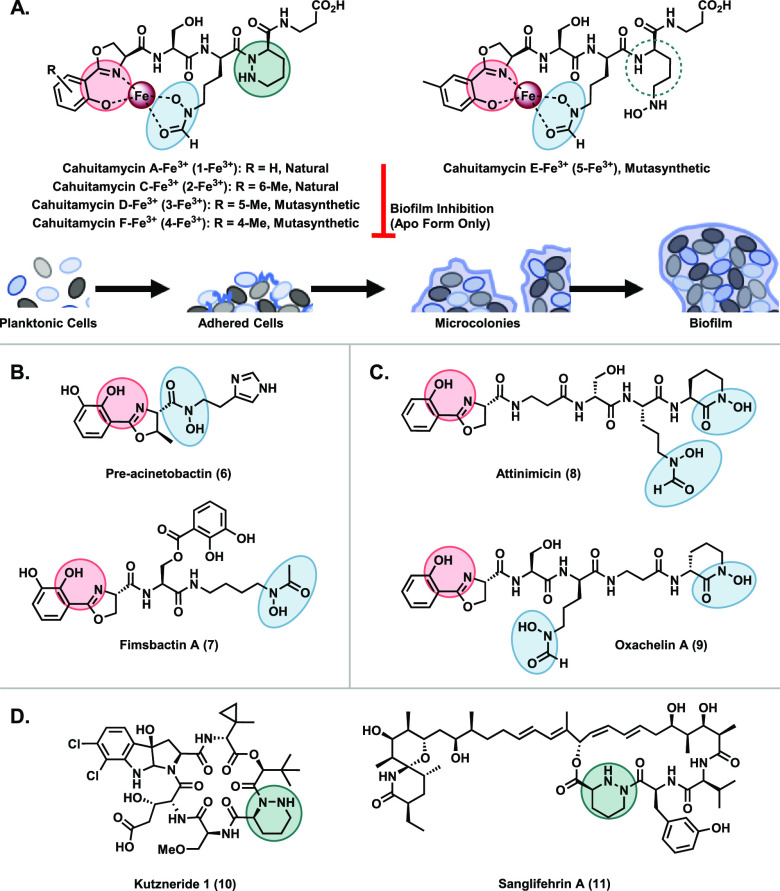
(A) Reported structures of the cahuitamycins, natural products with iron-chelation activity that inhibit *A. baumannii* biofilm formation (apo-form only); (B) Siderophores from *A. baumannii*; (C) Natural product structural isomers to cahuitamycin A; (D) Piperazic acid-containing natural products. *Key*: Red = *o*-phenolate oxazoline, Blue = hydroxamate, Green = piperazic acid.

The biosynthetic origins of piperazates in natural products are a relatively recent area of study. Foundational work by Walsh and co-workers demonstrated via isotopic labeling experiments that ornithine and *N*-hydroxy-ornithine are direct precursors to piperazic acid monomer *in vivo*.^23^ To date, only KtzT from the kutzneride pathway and homologues have been functionally characterized and demonstrated to generate monomeric piperazic acid.^24^ The existence of cahuitamycin E, as the only member of the cahuitamycin family to contain *N*-hydroxy-ornithine in place of the piperazate residue, can be explained one of two ways: (1) Piperazic acid monomer is synthesized by the producing strain by an as-of-yet unidentified off-cluster enzyme and incorporated into the scaffold via a promiscuous adenylation domain that can accept piperazic acid or *N-*hydroxy-ornithine, or (2) the enzymatic assembly line that synthesizes the cahuitamycins has an as-of-yet uncharacterized activity that mediates the N–N bond formation leading to a piperazate motif in the growing peptide, with premature cleavage leading to cahuitamycin E. This ambiguity, along with the intriguing bioactivity of the scaffold, naturally invites a complementary synthetic approach to answer key questions regarding the structure and activity of the cahuitamycins. We embarked on the diverted total synthesis of cahuitamycin A and structural analogs to provide a framework for future study of this important natural product.

Our synthetic approach relied on the convergent coupling of three peptides, wherein a linchpin ornithine fragment would join the piperazic acid-β-alanine and phenolic oxazoline moieties (Scheme 1). To this end, N2-Cbz-d-piperazic acid **(+)-12** was accessed from 5-bromo-pentanol in seven steps (relying on organocatalytic l-proline to form the asymmetric C–N bond) and was coupled with β-alanine benzyl ester to furnish protected dipeptide **(+)-13** (enantiomeric excess >97%, Figure S1). In parallel, *N*_α_-Fmoc-*N*_δ_-formyl-*N*_δ_-benzyloxy-d-ornithine **(−)-14** was derived from *N*_α_-Boc-*N*_δ_-Cbz-d-ornithine in seven steps. As expected, amide bond formation between these two fragments was nontrivial, owing to the well documented non-nucleophilicity of the N2-position.^25^ After standard procedures failed (Table S6), we turned to the literature for procedures known to mediate couplings between non-nucleophilic amines and hindered acids. Ultimately, we found that prestirring acid **(−)-14** in Ghosez’s reagent^26^ followed by reflux in benzene with **(+)-13** and silver cyanide resulted in protected tripeptide **(+)-15** in 80% isolated yield.

**Scheme 1:**
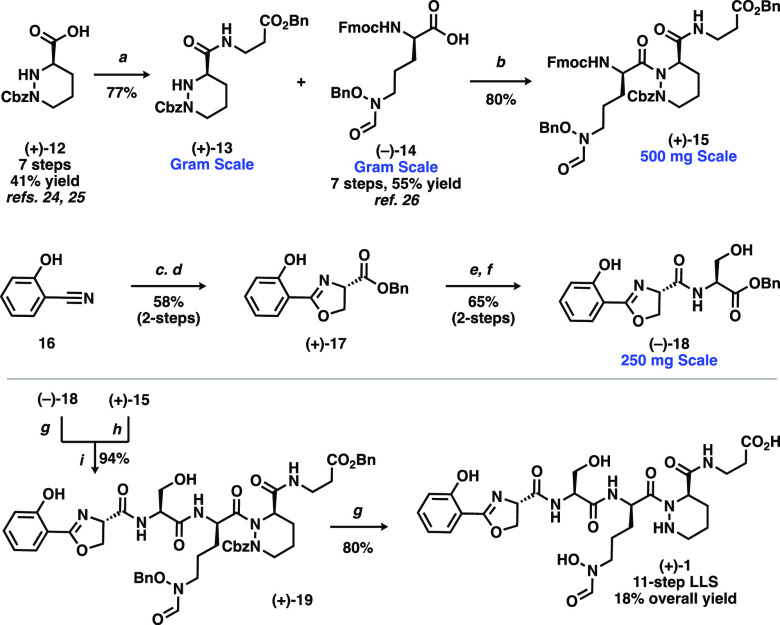
Synthesis of the Reported Structure of Cahuitamycin A **(+)-1** Conditions: (a) EDC, HOBt, β-ala-CO_2_Bn, NEt_3_, DMF, 0 °C to RT; (b) Ghosez’s reagent, DCM, 0 °C then **(+)-13**, AgCN, benzene, reflux; (c) AcCl, MeOH; (d) l-ser-CO_2_Bn, 1,2-DCE, reflux; (e) Pd/C, EtOAc, H_2_; (f) EDC, HOBt, l-ser-CO_2_Bn, NEt_3_, DMF, 0 °C to RT; (g) Pd/C, MeOH, H_2_; (h) 4-(aminomethyl)piperidine, DCM, then pH 5.5 buffer; (i) EDC, HOBt, NEt_3_, CH_3_CN, 0 °C to RT.

Finally, nitrile **16** was transformed into the corresponding imidate and cyclized with l-ser-CO_2_Bn to give **(+)-17**. After hydrogenolysis, EDC/HOBt coupling with l-ser-CO_2_Bn gave fragment **(−)-18**. Reductive deprotection was performed in parallel to basic Fmoc-cleavage of **(+)-15**, and the deprotected materials were coupled to furnish **(+)-19** in 94% yield over two steps. Global deprotection provided final product **(+)-1** in 80% yield. In total, the target structure was achieved in a longest linear sequence of 12 steps and 18% overall yield.

With a synthetic sample and natural material in hand, we sought to fully characterize the natural product and confirm its structure. ^1^H NMR of our synthetic material showed substantial differences from that of the authentic isolated material. HPLC coinjection confirmed the distinct identities of the two materials (Figure S2). The chemical shift of the western portion of synthetic **(+)-1** matched well with that of the authentic material; however, multiple signals associated with the piperazic acid residue, as well as the signal of the ornithine stereocenter, differed by up to ∼1.0 ppm.

We thus set out to determine the true structure of cahuitamycin A by diverted total synthesis. We pursued three alternative structures based on our ^1^H NMR data (Table S1) and bioinformatic analysis from the isolation report. (1) Piperazic acid residues are well-known to confer structural rigidity onto peptides and have large effects on three-dimensional conformation and intramolecular interactions;^25^ therefore, we hypothesized that an inactive epimerase domain in NRPS module CahC could lead to epimeric structure **(−)-22**. (2) Piperazic acid natural products consist of N2-connected structures (as in the kutznerides and the reported cahuitamycin scaffold) and N1-connected structures (as in the sanglifehrins (Figure 1). The isolation report lists HMBC correlations from the ornithine carbonyl to the proximal and distal protons of the piperazic acid residue, which would in theory also be present from the reported scaffold *de novo*. (3) Cahuitamycins are assembled by four NRPS enzymes, of which the piperazic acid and β-alanine residues are the only units reported to be appended by their own individual modules. Based on the large parts per million differences in chemical shifts of ^1^H signals of the piperazic acid, as well as *J*-coupling discrepancies of ^1^H signals of the β-alanine, we hypothesized alternate scaffold **(+)-31**, containing swapped C-terminal residues arising from swapped CahD and CahC modules.

Toward this end, we sought to access three distinct isomeric compounds through a diverted synthetic approach (Scheme 2). By strategic modification of our synthetic route, we conveniently arrived at these targeted isomeric analogs from branch-point intermediates. Enantiomeric dipeptide **(−)-13** (accessed using organocatalytic d-proline) was taken through analogous coupling/deprotection sequences with **(−)-14** and **20** to give the epimeric **(−)-22** in the same 12-step LLS and 13% overall yield. By altering the protecting scheme en route to the piperazic acid subunit, we arrived at diprotected piperazic alcohol **(−)-23**, which was oxidized to acid **(+)-24** and subjected to amide coupling to give **(+)-25**. After double Boc-deprotection, regioselective amide coupling at the N1-position with **(−)-14** was accomplished to give **(−)-26**, which was regioselective owing to the poor nucleophilicity of the N2-position. Another analogous deprotection/coupling sequence provides **(+)-27** in the same step-count and an overall yield of 21%. Finally, C-terminal benzyl protection of **(+)-12** gave **(+)-28**, which could be coupled with Fmoc-β-alanine via the optimized Ghosez procedure. From here, sequential deprotections and couplings with **(−)-14** and **20** gave **(+)-31** in 14 steps and 13% overall yield.

**Scheme 2:**
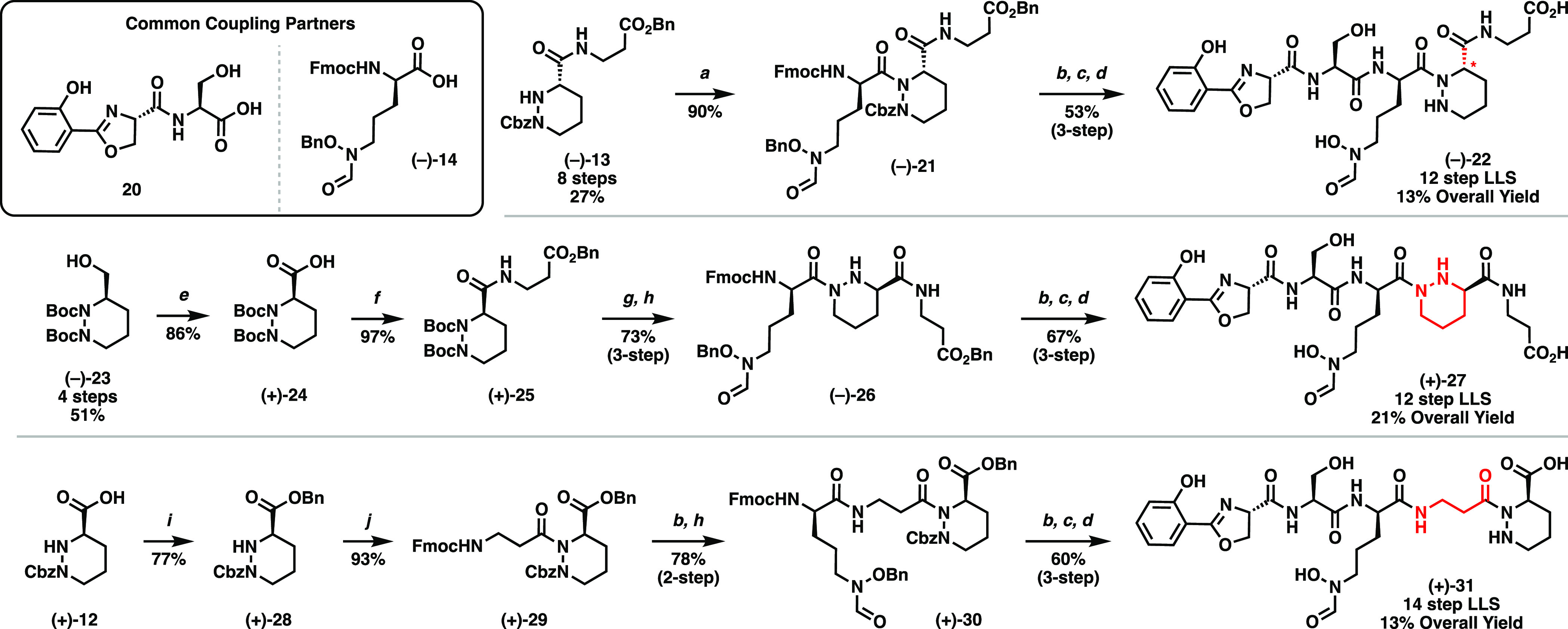
Synthesis of Hypothesis-Driven Analogs of Cahuitamycin A Conditions: (a) (−)-**14**, Ghosez’s reagent, DCM, 0 °C then (−)-**13**, AgCN, benzene, reflux; (b) 4-(aminomethyl)piperidine, DCM, then pH 5.5 buffer; (c) **20**, EDC, HOBt, NEt_3_, CH_3_CN, 0 °C to RT; (d) Pd/C, MeOH, H_2_; (e) TEMPO, NaClO_2_, CH_3_CN, pH 6.4 buffer, NaClO; (f) EDC, HOBt, β-alanine benzyl ester TsOH, DMF, NEt_3_, DMF, 0 °C to RT; (g) TFA, DCM; (h) **(−)-14**, EDC, HOBt, NEt_3_, CH_3_CN, 0 °C to RT; (i) K_2_CO_3_, BnBr, DMF; (j) Fmoc-β-alanine, Ghosez’s reagent, DCM, 0 °C then **(+)-28**, AgCN, benzene, reflux.

With the synthetic isomers in hand, we turned to spectral and biological analyses. Unfortunately, the spectral data of each were inconsistent with those of authentic cahuitamycin A. Specifically, while our four synthetic cahuitamycin isomers have differences between their ^1^H NMR spectra, they all qualitatively contain peaks associated with the piperazic acid residue in similar chemical shifts regions (namely between 4.3–5.5 ppm and 2.75–3.25 ppm, Figure 2). These findings were borne out by inhibition studies of *A. baumannii* ATCC 17978, in which planktonic growth was measured in the presence of varying concentrations of natural and synthetic compounds (Figure S3). Authentic cahuitamycin A inhibited at levels comparable to those of the isolation report; by contrast, no synthetic isomer displayed such inhibition. These results provide evidence that the chemotype present in the cahuitamycins responsible for their biological activity is not present in a diverse array of piperazic-acid-containing structural isomers.

**Figure 2:**
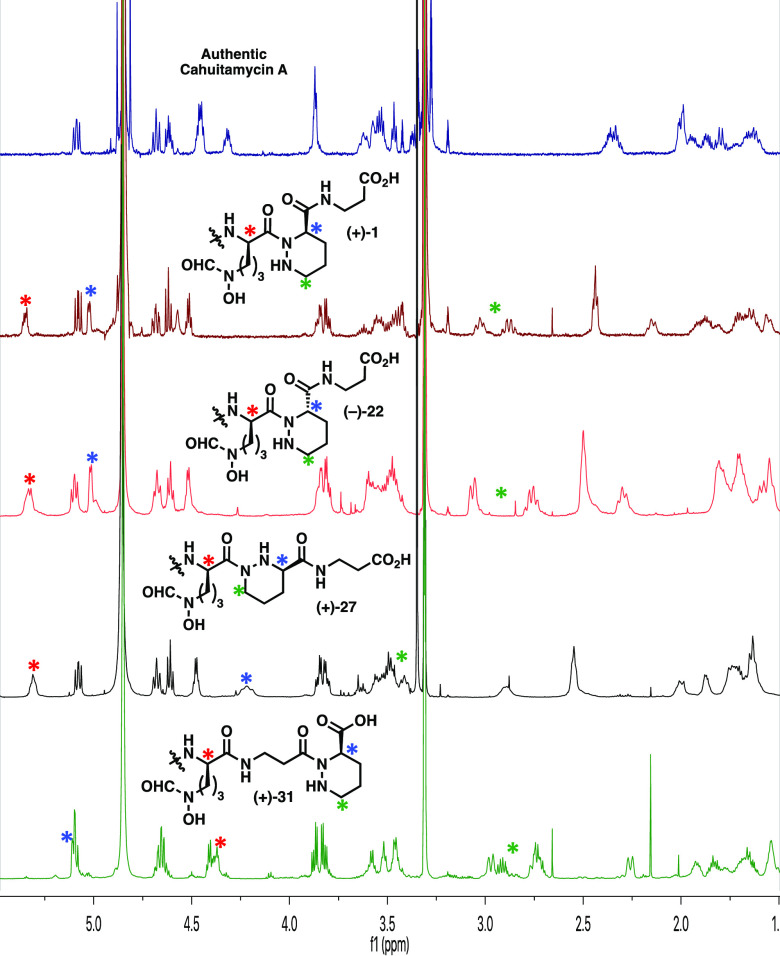
^1^H-NMR spectra of cahuitamycin A and synthetic isomers. From top-to-bottom: Authentic natural product, synthetic reported structure **(+)-1**, diastereomer **(−)-22**, N1-connected analogue **(+)-27**, and C-terminal piperazic acid **(+)-31**. Individual signals of interest are marked with colored asterisks.

As of this publication, the true structure of cahuitamycin A remains unknown. However, we believe the characterization data of our isomeric compounds provide key insights for future structure elucidation. Considering the recent report by Clardy and co-workers on the shared evolutionary origins of the biosynthetic enzymology of the cahuitamycins, we re-examined whether the cahuitamycin scaffold definitively contains a piperazic acid. Upon close examination of the characterization data of authentic cahuitamycin A, we find the evidence of this claim to be inconclusive. The existence of cahuitamycin E also raises questions about the presence of the piperazate in the other cahuitamycins and provides further insights into a potential alternative scaffold. While the biosynthesis of the piperazic acid monomer has recently been functionally characterized,^23,24^ there remains no other examples of a piperazate being assembled as part of a growing nonribosomal peptide chain. At the time of the initial isolation report of the cahuitamycins, the enzymes that incorporated piperazic acid into nonribosomal peptides had yet to be elucidated. Recently, Ryan et al. disclosed the biosynthetic process for piperazic acid formation and its adenylation through a combination of isotopic incorporation studies, *in vitro* reconstitution, and site-directed mutagenesis.^27^ With this new information in hand, 167 potential piperazic acid adenylation domains were identified, and **11** specificity-conferring codes of piperazic acid adenylation domains were summarized in addition to CahC, which was the putative adenylation domain for the piperazate in the cahuitamycin gene cluster. Interestingly, CahC was not related to the other Piz adenylation domains and instead may be specific for N^5^-OH-Orn given its similarity to the sequence identity for N^6^-OH-Lys activation.^28^ These reports, taken together with our synthetic work, provide compelling evidence that the cahuitamycin family of natural products does not contain piperazic acid.

In an effort to reconcile these discrepancies, we hypothesize four alternative structures wherein intramolecular lactamization leads to nonpiperazate cyclic peptide isomers of the proposed structure (Figure S4). Importantly, each of these would result in a hexadentate chelator, which is generally considered to be optimal for siderophores. Of these, we favor the structure in which an uncyclized hydroxyornithine residue performs an intramolecular cyclization via nucleophilic attack onto the C-terminal enzyme-linked carbonyl forming a 10-membered hydroxamate lactam (Figure 3). This structure explains the existence of cahuitamycin E and preserves the overall biosynthetic logic while accounting for the absence of the proposed piperazic acid residue. While not directly analogous, the existence of the 10-membered hydroxamate lactone fuscachelin A, a siderophore of *Thermobifida fusca*, serves as a precedent of this type of medium-sized cyclic hydroxamate.^29^ Further investigation is required to definitively assign the true structures of the cahuitamycins.

**Figure 3:**
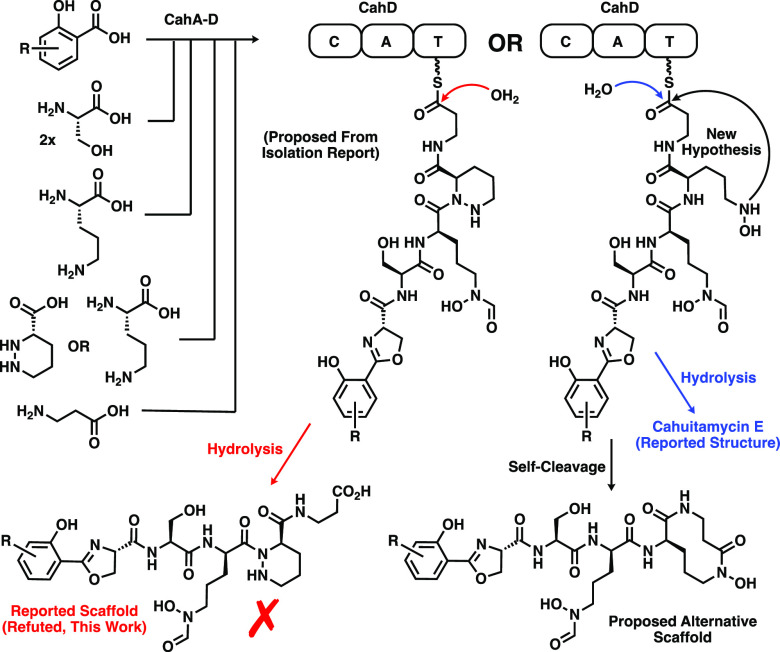
Biosynthesis of cahuitamycins proposed in isolation report. The piperazate containing scaffold (bottom left) has been refuted. One of four alternative scaffolds, hypothesized to derive from self-cleavage from the enzyme by cyclization of the *N*-hydroxy-ornithine residue onto the C-terminus to form a 10-membered lactam, is shown (bottom right).

Since the initial report of the cahuitamycins, over a dozen reviews and articles noting the bioactivity^30^ and biosynthesis^20,21,31^ of this natural product family have been reported. Notably, the antibiofilm activity of these natural products has further garnered synthetic interest beyond the work of our own lab, leading to at least one other known attempted synthesis of the unresolved cahuitamycin structures.^32^ The sustained interest in these antimicrobial natural products, coupled with their partially undetermined structures, highlights the need for further investigations into these promising scaffolds. Through this work, we have executed the first total synthesis of the reported structure of cahuitamycin A, unveiling the ambiguity surrounding the true structure of this natural product. Accordingly, we synthesized three rationally designed piperazate-containing isomers. By careful analysis of the spectral data and biological activity, we demonstrate that the four synthesized structural isomers do not align with the reported data of authentic cahuitamycin A. This sets the stage for future synthetic efforts to definitively elucidate the true structure of this ambiguous natural product. This work, yet again, underscores the power of synthetic organic chemistry as a complementary tool to probe complex biological phenomena and unlock the secrets of nature’s chemical assembly.

## Data Availability

The data underlying this study are available in the published article and its Supporting Information.

## Supporting Information Available

Reaction conditions, NMR spectra, HPLC traces and supporting figures (PDF)

## Dedication

This paper is dedicated in memory of my former advisor Prof. Christopher T. Walsh. His legacy and the impact on the field of natural product biosynthesis and chemical biology has been transformational.
