# Supplementary material for: Total Synthesis of the Reported Structure of Cahuitamycin A: Insights into an Elusive Natural Product Scaffold
Source: Org Lett. 2023 Dec 18;25(51):9243–8. doi: 10.1021/acs.orglett.3c03993 (PMC10758118; doi:10.1021/acs.orglett.3c03993)
Supplement: Supplementary file 1 — ol3c03993_si_001.pdf [file ol3c03993_si_001.pdf]

Supporting Information

Total Synthesis of the Reported Structure of Cahuitamycin A: Insights into an Elusive Natural Product Scaffold

Justin A. Shapiro[1], Savannah J. Post[1], Gavin C. Smith[1], William M. Wuest[1,2]

[1] Department of Chemistry, Emory University, Atlanta, GA 30322, United States.

[2] Emory Antibiotic Resistance Center, Emory University, Atlanta, GA 30322, United States.

Table of Contents

1. **Supporting Figures** … S2
2. **Supporting Tables** … S4
3. **Supporting Schemes** … S9
4. **General Methods** … S11
5. **Synthetic Procedures** … S12
6. **Biological Procedures** … S35
7. **Spectral Data** … S36

1. Supporting Figures

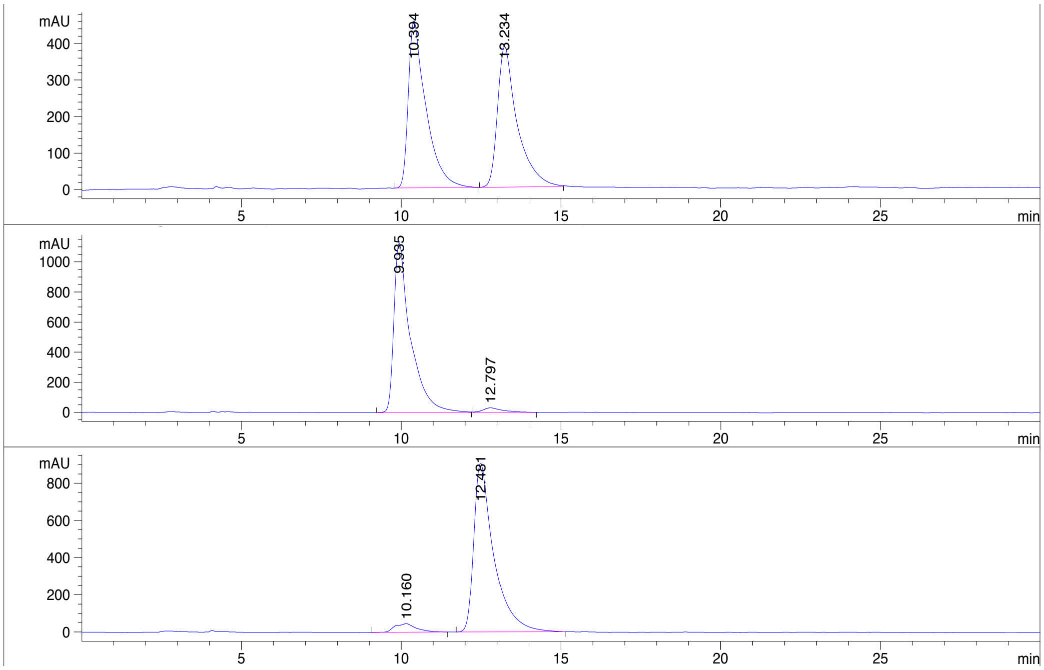

Supporting Figure 1: Chiral HPLC chromatograms of racemic **13** (top), **(+)-13** (middle), and **(–)-13** (bottom).

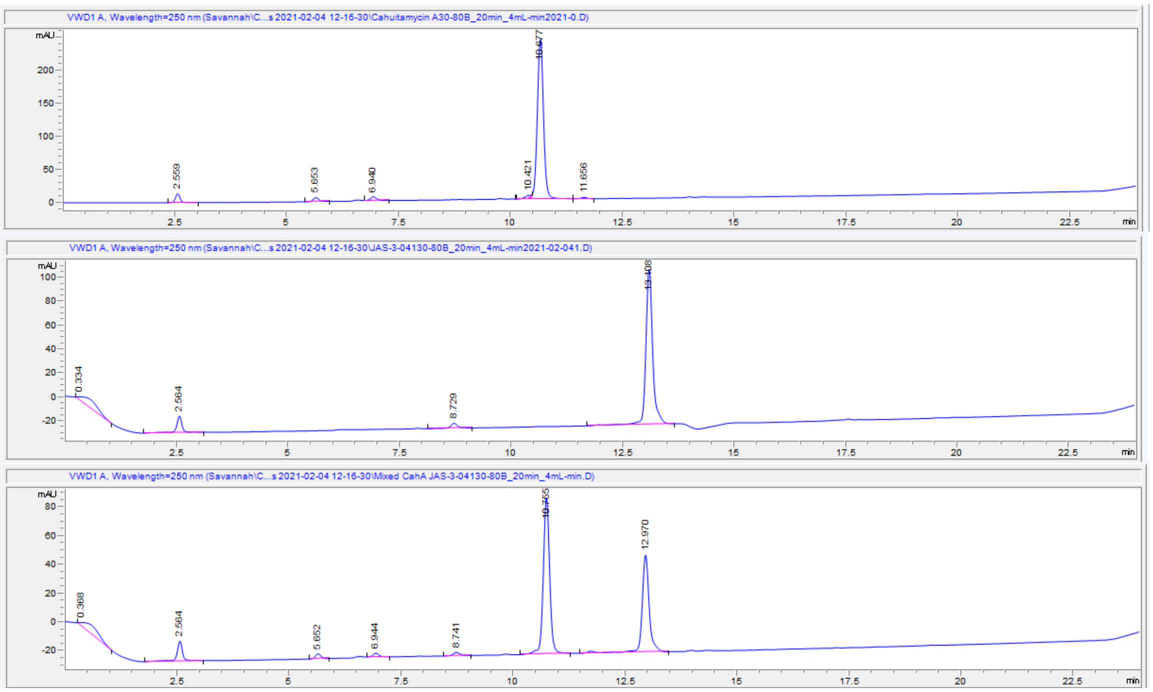

Supporting Figure 2: Analytical C18-HPLC chromatogram of authentic cahuitamycin A (top), synthetic **(+)-1** (middle), and a co-injecting of cahuitamycin A and **(+)-1** (bottom).

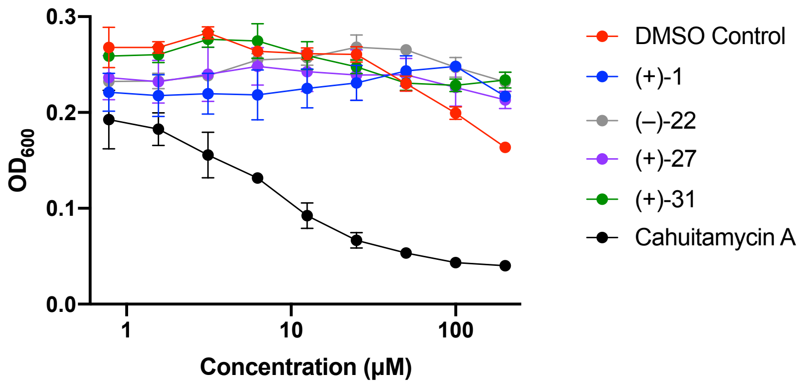

Supporting Figure 3: Growth of A. baumannii ATCC 17978 in the presence of authentic cahuitamycin A (black), synthetic (+)-1 (blue), (-)-22 (grey), (+)-27 (purple), (+)-31 (green), and DMSO control (red). Error bars represent standard deviations from three independent replicates.

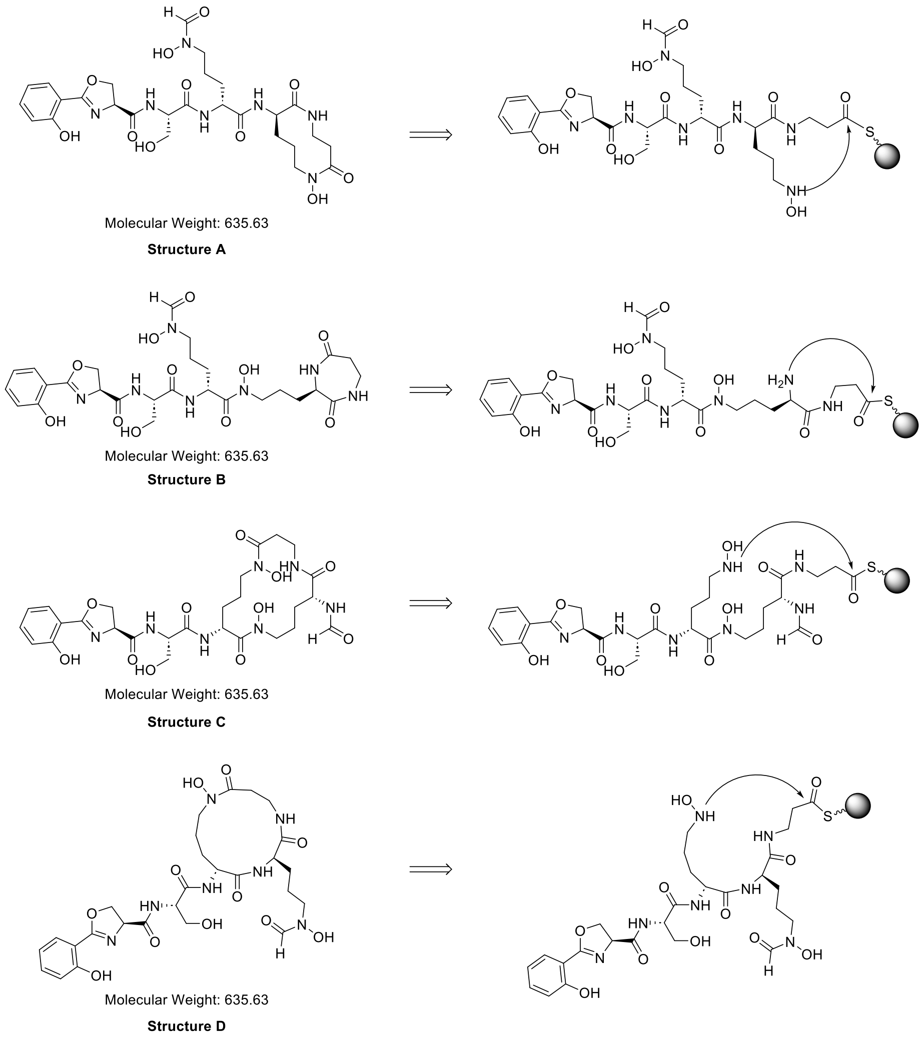

Supporting Figure 4: Proposed Cahuitamycin A structures and biosynthetic pathways.

2. Supporting Tables

Position	Authentic Cahuitamycin A	Synthetic (+)-1
1		
2	2.35 (m)	2.43 (t, 6.4)
3	3.37 (dd, 8.2, 5.2), 3.53 (m)	3.43 (m), 3.48 (m)
4		
5	4.45 (m)	5.02 (dd, 5.4, 1.3)
6	1.73 (m), 2.02 (m)	1.86 (m), 2.16 (m)
7	1.94 (m), 1.98 (m)	1.58 (m), 1.66 (m)
8	3.53 (m), 3.62 (m)	2.88 (q, 12.8), 3.03 (t, 12.7)
9		
10	4.31 (m)	5.35 (dd, 8.9, 4.3)
11	1.63 (m), 1.85 (m)	1.64 (m), 1.88 (m)
12	1.63 (m), 1.67 (m)	1.71 (m), 1.81 (m)
13	3.47 (t, 6.4)	3.54 (m)
14	8.25 (s)	8.29 (s)
15		
16	4.46 (m)	4.52 (q, 5.3)
17	3.87 (d, 4.8)	3.81 (dd, 11.1, 5.1), 3.85 (m)
18		
19	5.09 (dd, 10.5, 8.0)	5.08 (dd, 10.5, 8.0)
20	4.61 (td, 8.3, 5.3), 4.68 (t, 9.6)	4.62 (t, 8.2), 4.68 (td, 10.6, 2.7)
21		
22		
23	7.70 (d, 7.8)	7.70 (dd, 7.9, 1.7)
24	6.91 (t, 7.6)	6.90 (t, 7.6)
25	7.42 (t, 7.8)	7.42 (t, 7.9)
26	6.97 (d, 8.4)	6.97 (d, 8.3)
27		

Supporting Table 1. Comparison of ^{1}H-NMR signals in CD_3OD of authentic cahuitamycin A* and synthetic **(+)-1**. Notable differences highlighted in yellow.

*Note: As the reported structure of cahuitamycin A has been refuted and the true structure remains unknown, the atom numbering for authentic cahuitamycin A shown in Supporting Table 1 is based on the assignments found in the isolation report. Assignments for synthetic **(+)-1** based on 2D-NMR (Supporting Table 2).

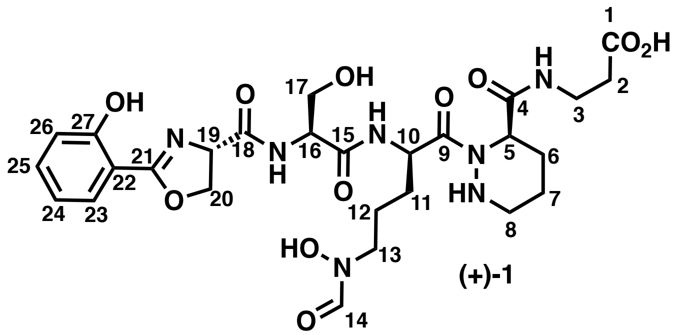

(+)-1

Position	^{1}H	^{13}C	COSY	TOCSY	HMBC
1		177.76			2, 3
2	2.43 (t, 6.4)	35.48	3	3	1, 3
3	3.43 (m), 3.48 (m)	35.42	2	2	1, 2, 4
4		171.57			3, 5
5	5.02 (dd, 5.4, 1.3)	51.31	6	6, 7, 8	4, 6, 7
6	1.92 (m), 2.16 (m)	25.76	5, 7	5, 7, 8	5, 7
7	1.58 (m), 1.66 (m)	20.72	6, 8	5, 6, 8	5, 6
8	2.88 (q, 12.8), 3.03 (t, 12.7)	46.5	7	5, 6, 7	6, 7
9		173.94			10
10	5.35 (dd, 8.9, 4.3)	49.03	11	11, 12, 13	9, 11
11	1.68 (m), 1.88 (m)	27.85	10, 12	10, 12, 13	10, 12, 13
12	1.72 (m), 1.82 (m)	22.4	11, 13	10, 11, 13	11, 13
13	3.54 (m)	45.8	12	10, 11, 12	11, 12, 14
14	8.29 (s)	162.59			13
15		170.53			16, 17
16	4.52 (q, 5.3)	55.33	17	17	15, 17
17	3.81 (dd, 11.1, 5.1), 3.85 (m)	61.65	16	16	15, 16
18		171.87			19, 20
19	5.08 (dd, 10.5, 8.0)	68.08	20	20	18, 20
20	4.62 (t, 8.2), 4.68 (td, 10.6, 2.7)	69.27	19	19	19, 21
21		167.42			19, 20, 23, 26
22		110.31			24, 26
23	7.70 (dd, 7.9, 1.7)	128.11	24	24, 25, 26	21, 22, 24, 25, 27
24	6.90 (t, 7.6)	118.65	23, 25	23, 25, 26	22, 23, 25, 26, 27
25	7.42 (t, 7.9)	133.62	24, 26	23, 24, 26	23, 24, 27
26	6.97 (d, 8.3)	116.33	25	23, 24, 25	22, 23, 24, 25
27		159.6			23, 25, 26

Supporting Table 2. 2D-NMR characterization of synthetic **(+)-1**.

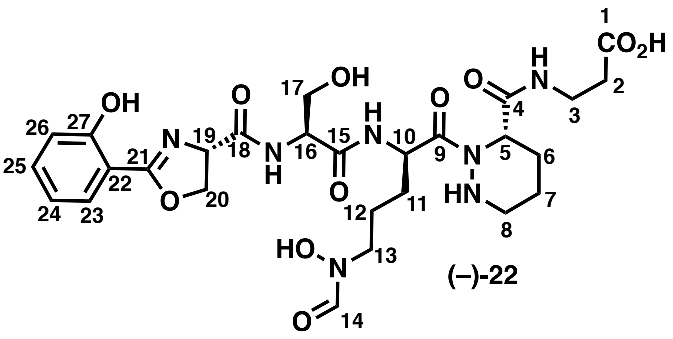

(–)-22

Position	^{1}H	^{13}C	COSY	TOCSY	HMBC
1		175.44			
2	2.53 (s)	34.36	3	3	1, 3
3	3.49 (m)	35.71	2	2	1, 2, 4
4		170.49			
5	5.02 (d, 5.6)	51.72	6	6, 7, 8	4, 6, 7
6	1.81 (m), 2.30 (13.2)	24.83	5, 7	5, 7, 8	5, 8
7	1.54 (m), 1.79 (m)	20.8	6, 8	5, 6, 8	4, 5
8	2.77 (q, 12.8), 3.07 (d, 13.7)	46.81	7	5, 6, 7	6
9					
10	5.35 (d, 9.4)	49.09	11	11, 12, 13	
11	1.69 (m)	22.36	10, 12	10, 12, 13	12, 13
12	1.80 (m)	28.07	11, 13	10, 11, 13	11, 13
13	3.57 (m)	45.7	12	10, 11, 12	11, 12, 14
14	8.29 (s)	45.83			
15		170.58			
16	4.52 (q, 5.5)	55.15	17	17	15, 17
17	3.82 (m)	61.45	16	16	15, 16
18		171.89			
19	5.10 (dd, 10.5, 7.9)	67.94	20	20	18, 20, 21
20	4.61 (t, 8.3), 4.68 (t, 9.6)	69.31	19	19	18, 19, 21
21		167.34			
22		110.03			
23	7.69 (dd, 7.9, 1.7)	128.11	24, 25	24, 25, 26	21, 25, 27
24	6.90 (t, 7.6)	118.54	23, 25, 26	23, 25, 26	22, 26
25	7.39 (t, 8.2)	133.61	23, 24, 26	23, 24, 26	23, 27
26	6.97 (d, 8.3)	116.31	24, 25	23, 24, 25	22, 24, 27
27		159.53			

Supporting Table 3. 2D-NMR characterization of synthetic **(–)-22**.

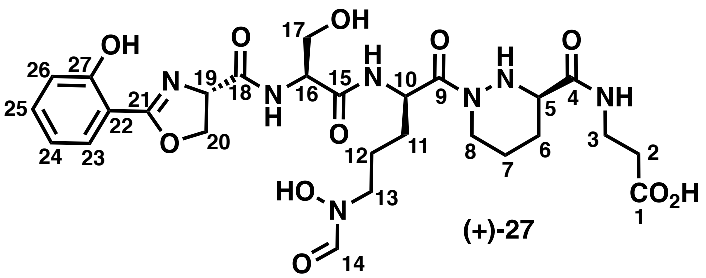

(+)-27

Position	^{1}H	^{13}C	COSY	TOCSY	HMBC
1		174.65			
2	2.50 (t, 6.5)	33.71	3	3	1, 3
3	3.50 (m)	35.25	2	2	1, 2
4					
NH(piz)	2.85 (d, 9.6)		5, 6	6	
5	4.23 (m)	41.41	NH(piz), 6	NH(piz), 6, 8	7, 8
6	1.65 (m), 1.90 (m)	22.3	NH(piz) 5, 7	NH(piz), 5, 7, 8	5, 7, 8
7	1.90 (m), 2.01 (m)	26.91	6, 8	5, 6, 8	5, 6, 8
8	3.47 (m), 3.41 (m)	59.67	7	NH(piz), 5, 6, 7	5, 7, 8, 9
9		172.44			
10	5.28 (m)	49.44	11	11, 12, 13	9, 11, 12, 15
11	1.67 (m), 1.77 (m)	22.35	10, 12	10, 12, 13	10, 12, 13
12	1.73 (m), 1.65 (m)	27.73	11, 13	10, 11, 13	11, 13
13	3.57 (m), 3.65 (m)	45.81	12	10, 11, 12	11, 12, 14
14	8.30 (s)	171.45			
15		170.39			
16	4.44 (q, 5.2)	55.4	17	17	15, 17
17	3.82 (m)	61.61	16	16	15, 16
18		171.89			
19	5.04 (dd, 10.5, 7.9)	67.92	20	20	18, 20, 21, 22
20	4.57 (t, 8.2), 4.64 (t, 8.2)	69.25	19	19	18, 20, 21
21		167.36			
22		110.14			
23	7.65 (dd, 7.9, 1.7)	128.15	24	24, 25, 26	21, 25, 26, 27
24	6.86 (t, 7.5)	118.59	23, 25	23, 25, 26	22, 23, 25, 26, 27
25	7.38 (ddd, 8.6, 7.2, 1.7)	133.66	24, 26	23, 24, 26	22, 23, 24, 26, 27
26	6.93 (dd, 8.4, 1.1)	116.35	25	23, 24, 25	22, 24, 25, 26, 27
27		159.79			

Supporting Table 4. 2D-NMR characterization of synthetic **(+)-27**.

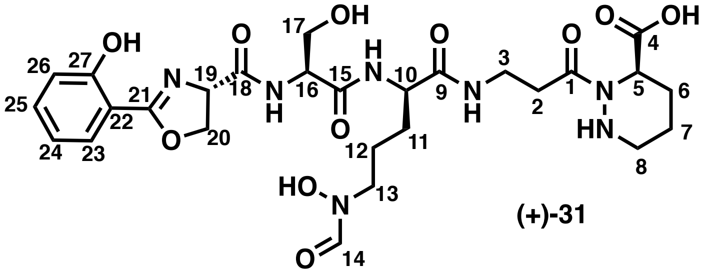

(+)-31

Position	^1H	^{13}C	COSY	TOCSY	HMBC
1		173.97			
2	2.89 (m), 2.80 (m)	31.87	3	3	1, 3
3	3.46 (dt, 7.0, 3.7)	35.56	2	2	2, 9
4		171.97			
5	5.10 (m)	51.93	6	6, 7, 8	1, 4, 6, 7
6	1.82 (dt, 13.4, 5.7), 2.26 (d, 13.5)	25.44	5, 7	5, 7, 8	4, 7, 8
7	1.55 (m), 1.63 (m)	21.57	6, 8	5, 6, 8	6, 8
8	2.97 (d, 13.8), 2.78 (m)	46.7	7	6, 7, 8	1, 7
9		172.2			
10	4.38 (m)	52.83	11	11, 12, 13	9, 11, 12, 15
11	1.68 (m), 1.88 (m)	22.74	10, 12	10, 12, 13	10, 12, 13
12	1.64 (m), 1.94 (m)	27.96	11, 13	10, 12, 13	10, 11, 13
13	3.58 (td, 6.4, 2.5), 3.52 (m)	45.6	12	10, 11, 12	11, 12, 14
14	8.29 (s)	157.92			13
15		171.2			
16	4.42 (m)	55.76	17	17	15, 17, 18
17	3.84 (qd, 11.1, 5.2)	61.39	16	16	15, 16
18		172.3			
19	5.09 (m)	67.97	20	20	18, 20, 21
20	4.66 (m)	69.14	19	19	19, 21
21		166.98			
22		159.3			
23	7.70 (dd, 7.9, 1.7)	128.49	24	24, 25, 26	21, 22, 25, 26
24	6.90 (t, 7.8)	118.55	23, 25	23, 25, 26	22, 23, 25, 26, 27
25	7.42 (ddd, 8.8, 7.3, 1.7)	133.56	24, 26	23, 24, 26	22, 23, 26, 27
26	6.97 (dd, 8.4, 1.1)	116.44	25	24, 25, 26	21, 22, 23, 24, 27
27		110.05			

Supporting Table 5. 2D-NMR characterization of synthetic **(+)-31**.

Reagents (eq.)	Solvent	Temp.	Time	eq. (+)-15	% yield
DCC (1.5), DMAP (0.1)	DCC	0 °C → RT	16 hr	1.1	0%
EDC (3.0), HOBt (3.0), NEt_3 (6.0)	DMF	0 °C → RT	16 hr	1.1	0%
HATU (1.2), DiPEA (4.5)	DMF	0 °C → RT	16 hr	1.1	0%
PyBroP (1.), DiPEA (3.0)	DCM	0 °C → RT	16 hr	1.1	0%
1) $SOCl_2$ (xs), then 2) $NaHCO_3$ (3.0)	1) DCM, then 2) DCM/H_2O	1) reflux, then 2) 0 °C → RT	1) 30 min, then 2) 16 hr	1.1	0%
1) $(COCl)_2$ (2.0), DMF (cat.), then 2) AgCN (0.1)	1) DCM, then 2) benzene	1) 0 °C, then 2) reflux	1) 30 min, then 2) 1 hr	1.1	0%
1) PivCl (1.0), NEt_3 (1.0), then 2) LiCl (cat.), NEt_3 (3.0)	1) DCM, then 2) DCM	1) 0 °C, then 2) RT → reflux	1) 30 min, then 2) 12 hr	1.5	0%
1) DAST (1.5), then 2) NEt_3 (2.0)	1) DCM, then 2) DCM	1) 0 °C, then 2) reflux	1) 1 hr, then 2) 16 hr	0.5	0%
BTFFH (1.15), DiPEA (3.5)	DCM	RT → 80 °C (sealed tube)	16 hr	1.0	0%
TCFH (1.0), NMI (2.0)	MeCN	RT → 60 °C	16 hr	1.0	0%
1) Cyanuric chloride (0.33), NEt_3 (cat.), then 2) $NaHCO_3$ (xs)	1) DCM, then 2) DCM/H_2O	1) RT, then 2) 0 °C → RT	1) 3 hr, then 2) 16 hr	0.5	0%
1) Ghosez's reagent (1.4), then 2) $Sc(Otf)_3$ (0.1)	toluene	1) 0 °C, then 2) RT	1) 30 min, then 2) 2 hr	0.5	trace
1) Ghosez's reagent (1.4), then 2) AgCN (0.5)	Benzene	1) 0 °C, then 2) reflux	1) 30 min, then 2) 1 hr	0.5	57%
1) Ghosez's reagent (1.4), then 2) AgCN (0.33)	Benzene	1) 0 °C, then 2) reflux	1) 30 min, then 2) 1 hr	0.33	80%

Supporting Table 6. Reaction screen for the synthesis of key intermediate **(+)-15**. All reactions are done relative to 1.0 equivalents of **(–)-14**. For all two-step sequences, **(–)-14** was added in the first step and **(+)-13** was added in the second step.

3. Supporting Schemes

Supporting Scheme 1. Synthesis of key intermediate **(+)-15**, featuring compounds not shown in main text.

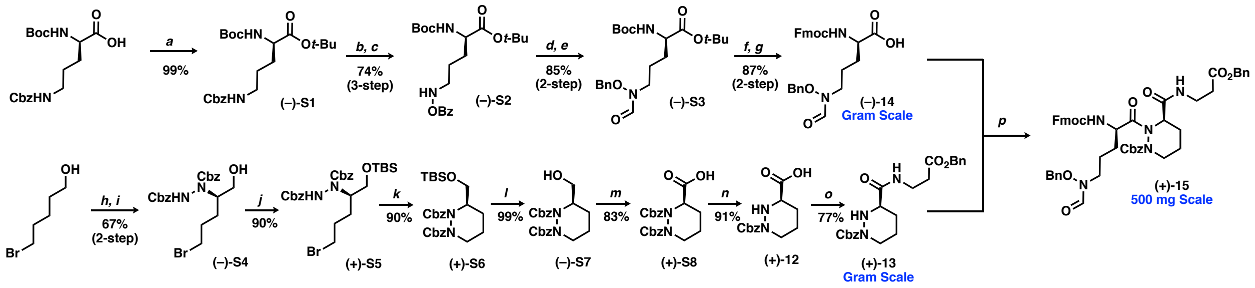

a.) O-*tert*-buty-*N,N'*-diisopropylisourea, *tert*-BuOH, reflux *b.)* Pd/C, H_2, EtOAc *c.)* 75% BPO, Cs_2CO_3, DCM *d.)* EDC, formic acid, DCM, 0° C to RT *e.)* BnBr, Hünig's base, MeOH *f.)* TFA, DCM *g.)* Fmoc-OSu, H_2O, THF, $NaHCO_3$ *h.)* PCC, DCM *i.)* Dibenzyl azodicarboxylate, L-proline, CH_3CN, 0° C, *then* $NaBH_4$, EtOH *j.)* TBSCl, imidazole, DMF *k.)* NaH, DMF, 0° C *l.)* TBAF, THF, 0° C *m.)* TEMPO, $NaClO_2$, NaClO, CH_3CN *n.)* KOH, THF *o.)* EDC, HOBt, DMF, NEt_3, β-alanine benzyl ester TsOH, 0° C to RT *p.)* (–)-15, Ghosez's reagent, DCM, 0° C, then (+)-20, AgCN, benzene, reflux

Supporting Scheme 2. Synthesis of **(+)-1**

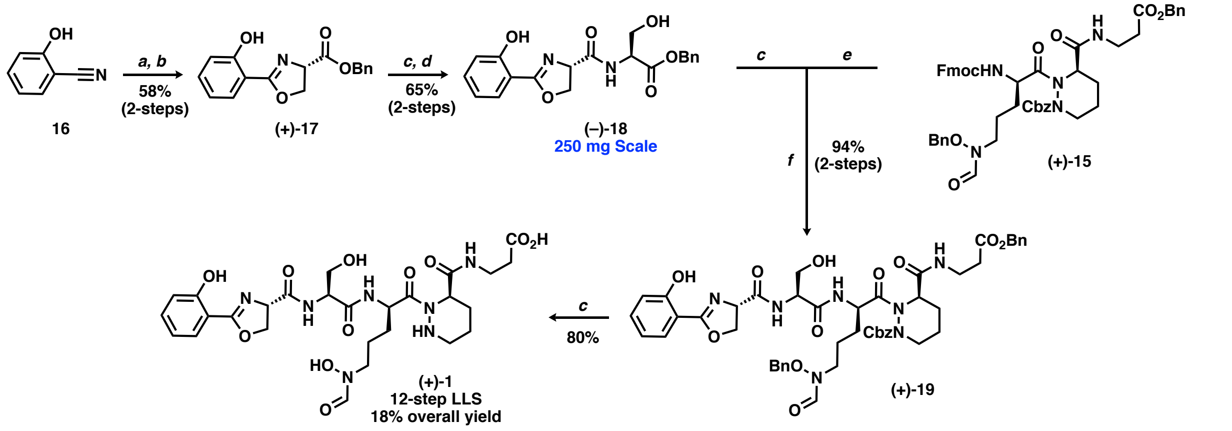

Conditions: *a.)* AcCl, MeOH *b.)* 1,2-DCE, L-serine benzyl ester HCl, reflux *c.)* Pd/C, H_2, MeOH *d.)* EDC, HOBt, DMF, NEt_3, L-serine benzyl ester HCl, 0° C to RT *e.)* 4-(aminomethyl)piperidine, DCM, then pH 5.5 buffer *f.)* EDC, HOBt, CH_3CN, NEt_3, 0° C to RT

Supporting Scheme 3. Synthesis of isomeric analog **(–)-22**.

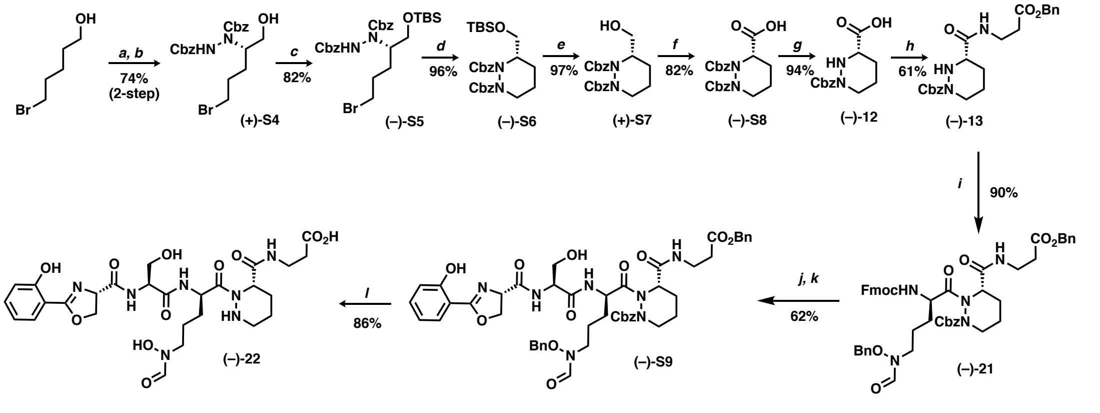

a.) PCC, DCM *b.)* Dibenzyl azodicarboxylate, D-proline, CH_3CN, 0° C, *then* $NaBH_4$, EtOH *c.)* TBSCl, imidazole, DMF *d.)* NaH, DMF, 0° C *e.)* TBAF, THF, 0° C *f.)* TEMPO, $NaClO_2$, NaClO, CH_3CN *g.)* KOH, THF *h.)* EDC, HOBt, DMF, NEt_3, β-alanine benzyl ester TsOH, 0° C to RT *i.)* (–)-15, Ghosez's reagent, DCM, 0° C, then (+)-20, AgCN, benzene, reflux *j.)* 4-(aminomethyl)piperidine, DCM, then pH 5.5 buffer *k.)* EDC, HOBt, CH_3CN, NEt_3, Hydrogenation Product of (–)-18 (see Supporting Scheme 2) *l.)* Pd/C, MeOH, H_2

Supporting Scheme 4. Synthesis of isomeric analog **(+)-27**.

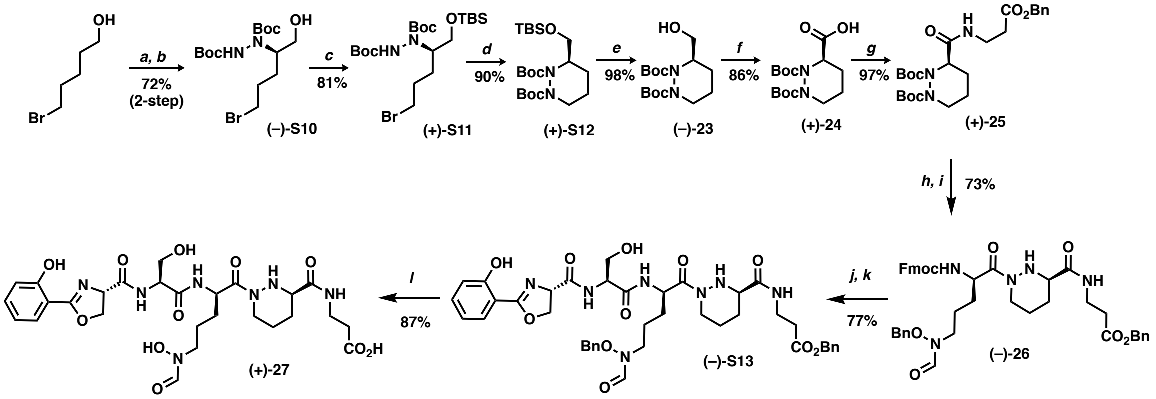

a.) PCC, DCM *b.)* Di-*tert*-butyl azodicarboxylate, L-proline, CH_3CN, 0° C, *then* $NaBH_4$, EtOH *c.)* TBSCl, imidazole, DMF *d.)* NaH, DMF, 0° C *e.)* TBAF, THF, 0° C *f.)* TEMPO, $NaClO_2$, NaClO, CH_3CN *g.)* EDC, HOBt, DMF, NEt_3, β-alanine benzyl ester TsOH, 0° C to RT *h.)* TFA, DCM *i.)* EDC, HOBt, CH_3CN, NEt_3, (–)-12, 0° C to RT *j.)* 4-(aminomethyl)piperidine, DCM, then pH 5.5 buffer *k.)* EDC, HOBt, CH_3CN, NEt_3, Hydrogenation Product of (–)-18 (see Supporting Scheme 2), 0° C to RT *l.)* Pd/C, MeOH, H_2

Supporting Scheme 5. Synthesis of isomeric analog **(+)-31**.

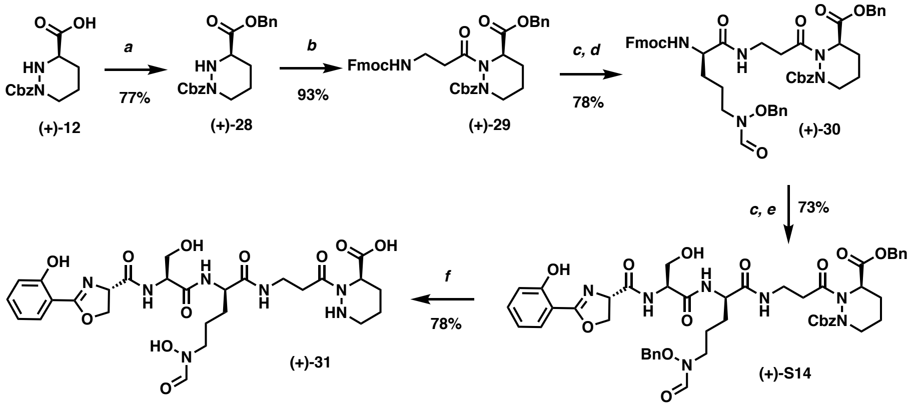

a.) K_2CO_3, BnBr, DMF *b.)* Fmoc-β-ala, Ghosez's reagent, DCM, 0° C, then (+)-21, AgCN, benzene, reflux *c.)* 4-(aminomethyl)piperidine, DCM, then pH 5.5 buffer *d.)* EDC, HOBt, CH_3CN, NEt_3, (–)-12, 0° C to RT *e.)* EDC, HOBt, CH_3CN, NEt_3, Hydrogenation Product of (–)-18 (see Supporting Scheme 2) 0° C to RT *f.)* Pd/C, MeOH, H_2

4. General Methods

NMR spectra were recorded using the following spectrometers: Varian INOVA500, Varian INOVA400, VNMR400, and Bruker Ascend 600. All NMR spectra were recorded under ambient temperature. Chemical shifts are quoted in ppm relative to solvents used (^{1}H: δ = 7.26 and ^{13}C: δ = 77.16 for residual $CHCl_3$, ^{1}H: δ = 3.49, 1.09 and ^{13}C: δ = 50.41 for residual CH_3OH, ^{1}H: δ = 1.56 for residual H_2O. The abbreviations used to describe splitting are as follows: s (singlet), d (doublet), t (triplet), q (quartet), m (multiplet), dd (doublet of doublets), dt (doublet of triplets), etc. Structural assignments were made with additional information from gCOSY, gHSQC, gTOCSY and gHMBC experiments. Chiral HPLC was performed on an Agilent 1100 series HPLC utilizing a CHIRALPAK OJ-H 4.6 x 150 mm analytical column.

Accurate mass spectra were recorded on a Thermo LTQ-FTMS using either APCI or ESI techniques. Infrared spectra were obtained using a Thermoscientific Nicolet with an attenuated total reflectance (ATR) with a Germanium crystal. Samples were tested neat or in chloroform. Peaks are reported in cm^{-1} and described as either weak (w), strong (s), or broad (b).

Specific rotations were obtained with 1 dm path length using a Perkin Elmer Model 341 Polarimeter with a Na/Hal lamp set to 598 nm. Samples were dissolved in either chloroform or water depending on the solubility restrictions of some compounds. In all cases, the polarimeter was zeroed to the solvent first. Measurements were taken over several minutes and then adjusted based on concentration.

Non-aqueous reactions were performed under an atmosphere of argon in flame-dried glassware with HPLC-grade solvents dried by passage through alumnina. Amine bases were freshly distilled over CaH_2 prior to use. Brine refers to a saturated aqueous solution of sodium chloride. Purification via flash chromatography refers to usage of Biotage Isolera One Automated column. Reactions monitored via thin-layer chromatography (TLC) using EMD Millipore® TLC silica gel glass plates with various stains specified in each procedure. Reactions monitored by LCMS were injected into an Agilent Technologies 1220 Infinity HPLC Liquid Chromatograph connected to an Advion Expression Compact Mass Spectrometer. Solvents used were HPLC grade water and acetonitrile each spiked with 0.1% formic acid.

Under sufficiently acidic conditions, residual metals in glassware and celite can be leeched out into solution where they can be tightly bound by unprotected bidentate chelating motifs present in siderophore-like compounds (usually resulting in a strongly colored complex). This can be avoided by pre-rinsing glassware with 6M HCl three times, water three times, and methanol three times. After air-drying, the glassware can be exposed to highly acidic solutions of siderophore-like compounds without further precautions."

5. Synthetic Procedures

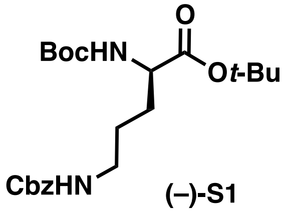

(–)-S1

(–)-S1: To a flask containing commercially available N_α-Boc-N_δ-Cbz-D-Ornithine (1.082 g, 2.95 mmol) and dichloromethane (16 mL) was added *O*-*tert*-Butyl-*N*,*N*'-diisopropylisourea (3.55 g, 14.77 mmol) followed by *tert*-butanol (16 mL). The reaction was heated to 50 °C (oil bath) and stirred for 24 hours at which point solvent was removed by rotary evaporation. The crude residue was purified by flash chromatography (gradient of 0->100% ethyl acetate in hexanes) to yield pure **(–)-S1** as a pale oil in quantitative yield (1.24 g, 2.94 mmol). Characterization data matched those previously reported.[1]

^{1}H NMR (500 MHz, CDCl$_3$): δ 7.40 – 7.27 (m, 5H), 5.08 (s, 2H), 4.92 (s, 1H), 4.16 (q, *J* = 7.3 Hz, 1H), 3.21 (q, *J* = 6.5 Hz, 2H), 1.84 – 1.76 (m, 1H), 1.67 – 1.49 (m, 3H), 1.45 (s, 9H), 1.43 (s, 9H).

^{13}C NMR (126 MHz, CDCl$_3$): δ 171.8, 156.5, 155.5, 136.7, 128.6, 128.2, 110.1, 82.2, 79.9, 66.7, 53.7, 40.8, 30.4, 28.5, 28.1, 25.9.

IR (neat): 1695 (s), 2930 (w), 2980 (w), 3353 (b) cm^{-1}.

$[\alpha]^{25}_D$: -0.8 ° (10 mg/mL in chloroform)

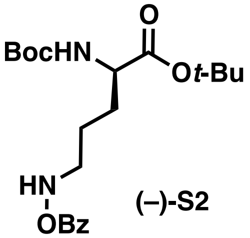

(–)-S2

(–)-S2: To a flask containing **(–)-S1** (9.05 g, 21.45 mmol) and ethyl acetate (200 mL) was added 10% palladium on activated charcoal (10 wt. % of starting material, 0.9 g). The flask was purged, backfilled with hydrogen gas, and stirred at room temperature until consumption of starting material was observed by TLC. The reaction mixture was filtered through celite and solvent was removed by rotary evaporation. The crude amine was taken on without further purification.

To a flask containing 75% benzoyl peroxide (13.85 g, 42.9 mmol) and cesium carbonate (20.95 g, 64.35 mmol) was added dichloromethane (20 mL). The suspension was stirred for two hours at which time a solution of the crude material in 10 mL dichloromethane and added. The reaction was stirred for 20 hours, at which point the mixture was filtered through celite and the solvent was removed by rotary evaporation. The crude residue was purified by flash chromatography (gradient of 0->100% ethyl acetate in hexanes) to yield pure **(–)-S2** as a pale oil in 74% yield (6.48 g, 15.87 mmol). Characterization data matched those previously reported.[2]

^{1}H NMR (600 MHz, CDCl$_3$): δ 7.99 (d, *J* = 7.8 Hz, 2H), 7.56 (t, *J* = 7.4 Hz, 1H), 7.43 (t, *J* = 7.1 Hz, 2H), 5.13 (d, *J* = 8.3 Hz, 1H), 4.19 (q, *J* = 7.5, 6.6 Hz, 1H), 3.15 (h, *J* = 5.6, 5.0 Hz, 2H), 1.90 (dq, *J* = 14.9, 9.0, 8.1 Hz, 1H), 1.77 – 1.62 (m, 3H), 1.43 (s, 9H), 1.41 (s, 9H).

^{13}C NMR (151 MHz, CDCl$_3$): δ 171.7, 166.9, 155.4, 133.4, 129.3, 128.5, 128.3, 82.0, 79.7, 53.7, 52.0, 30.5, 28.3, 28.0, 23.2.

IR (neat): 1716 (s), 2977 (w), 3367 (b) cm^{-1}.
$[\alpha]^{25}_D$: -12 ° (10 mg/mL in chloroform)

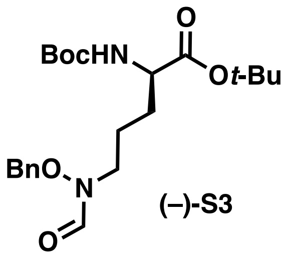

(–)-S3

(–)-S3: To a flask containing EDC (5.75 g, 30.1 mmol) and dichloromethane (25 mL) at 0 °C was added formic acid (1.137 mL, 30.1 mmol) and the mixture was stirred for 15 minutes. **(–)-S3** (4.15 g, 10.1 mmol) was dissolved in dichloromethane (25 mL) and added to the reaction mixture. The reaction was stirred for one hour at 0 °C and one hour at room temperature. The reaction was cooled to 0 °C and an additional portion of EDC (1.90 g, 10.1 mmol) and formic acid (0.379 mL, 10.1 mmol) were added. The reaction was stirred at 0 °C for 15 minutes and room temperature for 3 hours. The reaction was quenched with water and the aqueous layer was extracted three times with dichloromethane. The combined organic layers were washed with brine, dried over anhydrous sodium sulfate, and concentrated under rotary evaporation. The crude amide was taken on without further purification.

To a solution of the crude material dissolved in methanol at 0 °C was added benzyl bromide (3.60 mL, 30.1 mmol) and diisopropylethylamine (3.50 mL, 20.1 mmol). The reaction was stirred at 0 °C to room temperature for 12 hours, at which time solvent was removed by rotary evaporation. The residue was partitioned between water and ethyl acetate and the aqueous layer was extracted three times with ethyl acetate. The combined organic layers were washed with brine, dried over anhydrous sodium sulfate, and concentrated under rotary evaporation. The crude residue was purified by flash chromatography (gradient of 0->100% ethyl acetate in hexanes) to yield pure **(–)-S3** as a pale oil in 85% yield (3.62 g, 8.51 mmol). Characterization data matched those previously reported.[2]
^{1}H NMR (600 MHz, $CDCl_3$): δ 8.18 (s, 1H), 7.44 – 7.29 (m, 5H), 5.07 (d, J = 8.2 Hz, 1H), 4.81 (s, 2H), 4.16 (s, 1H), 3.58 (s, 2H), 1.82 – 1.75 (m, 1H), 1.74 – 1.62 (m, 2H), 1.62 – 1.54 (m, 1H), 1.42 (s, 18H).
^{13}C NMR (151 MHz, $CDCl_3$): δ 171.6, 163.1, 155.4, 134.3, 129.5, 129.2, 128.8, 82.0, 79.7, 77.8, 53.5, 43.8, 30.2, 28.3, 28.0, 25.7, 22.8.
IR (neat): 1684 (s), 1713 (s), 2336 (w), 2935 (w), 2977 (w), 3333 (b) cm^{-1}.
$[\alpha]^{25}_D$: -20 ° (10 mg/mL in chloroform)

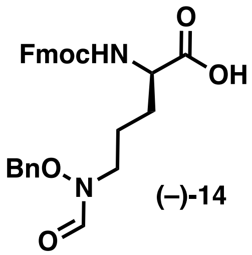

(–)-14

(–)-14: To a flask containing **(–)-S3** (2.64 g, 6.26 mmol) and dichloromethane (4 mL) was added trifluoroacetic acid (4 mL). The reaction was stirred in open air for 3 hours, at which point the solvent was removed by rotary evaporation. The crude residue was re-dissolved in dichloromethane and concentrated five times to remove excess trifluoroacetic acid. The crude amino acid was taken on without further purification.

To a flask containing the crude material dissolved in tetrahydrofuran (9 mL) and water (9 mL) was added Fmoc-OSu (2.15 g, 6.37 mmol) and sodium bicarbonate (3.06 g, 36.43 mmol). The pH was confirmed to be basic and the reaction was stirred for 16 hours, at which time organic solvent was removed by rotary evaporation. The remaining aqueous solution was diluted with water, adjusted to pH 2 by addition of 2M HCl, and extracted three times with ethyl acetate. The combined organic layers were dried over anhydrous sodium sulfate and concentrated by rotary evaporation. The crude residue was purified by flash chromatography (isocratic 94:3:3 dichloromethane/methanol/acetic acid). Fractions containing product were concentrated by rotary evaporation, re-dissolved in pentane and concentrated ten times to remove excess acetic acid. The residue was then partitioned between ethyl acetate and water to remove trace residual acetic acid and the aqueous layer was extracted three times with ethyl acetate at a pH of 2 (maintained by addition of 2M HCl throughout extraction). The combined organic layers were washed with brine, dried over anhydrous sodium sulfate, and concentrated by rotary evaporation to yield pure **(–)-14** as an amorphous off-white solid in 87% yield (2.65 g, 5.43 mmol). Characterization data matched those previously reported.[2]

^{1}H NMR (400 MHz, $CDCl_3$): δ 8.16 (s, 1H), 7.73 (d, *J* = 7.5 Hz, 2H), 7.57 (t, *J* = 6.6 Hz, 2H), 7.41 – 7.22 (m, 9H), 5.61 (d, *J* = 8.1 Hz, 1H), 4.78 (s, 3H), 4.37 (qd, *J* = 7.2, 5.1, 3.1 Hz, 2H), 4.18 (t, *J* = 7.2 Hz, 1H), 3.60 (d, *J* = 22.5 Hz, 2H), 1.88 (s, 1H), 1.69 (s, 3H).
^{13}C NMR (151 MHz, $CDCl_3$): δ 171.3, 163.2, 156.0, 143.9, 143.8, 141.3, 133.2, 129.5, 129.2, 128.8, 128.8, 127.8, 127.1, 125.2, 120.0, 82.4, 67.0, 54.0, 47.2, 43.7, 30.0, 28.04, 28.00, 22.7.
IR (neat): 1677 (s), 1721 (s), 2976 (w), 3323 (b) cm^{-1}.
$[\alpha]^{25}_{D}$: -10 ° (10 mg/mL in chloroform)

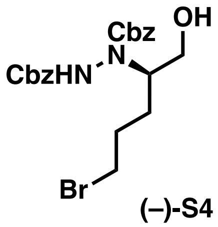

(–)-S4

(–)-S4: To a flask containing PCC (26.0 g, 120.6 mmol) and silica gel (20 g) in dichloromethane (250 mL) was added a solution of commercially available 5-bromo-pentanol (10 g, 59.9 mmol) in dichloromethane (50 mL) at a rate of 1mL/minute. The reaction was stirred for 1 hour and then filtered through celite and concentrated by rotary evaporation. The residue was redissolved in dichloromethane, passed through a silica plug, and concentrated by rotary evaporation. The crude aldehyde was taken on without further purification.

To a flask containing the crude material in acetonitrile (110 mL) was brought to 0 °C and dibenzyl azodicarboxylate (11.78 g, 39.53 mmol) and L-proline (0.454 g, 3.95 mmol) were added as solids. The reaction was stirred at 0 °C for 16 hours, at which point sodium borohydride (1.18 g, 31.2 mmol) and ethanol (48 mL) were added and the reaction was stirred for 40 minutes. The reaction was quenched with 10% citric acid and solvent was removed under rotary evaporation. The residue was partitioned between ethyl acetate and brine and the aqueous layer was extracted three times with ethyl acetate. The combined organic layers were dried over anhydrous sodium sulfate and concentrated under rotary evaporation. The crude residue was purified by flash chromatography (gradient of 0->100% ethyl acetate in hexanes) to yield pure **(–)-S4** as a white solid in 67% yield (12.31 g, 26.48 mmol). Characterization data matched those previously reported.[3]

^{1}H-NMR (600 MHz, $CDCl_3$): δ 7.39 –7.22 (m, 10H), 5.29 – 5.07 (m, 4H), 4.50 (s, 1H), 4.24 (s, 1H), 3.55 – 3.48 (br. s, 1H), 3.48 – 3.33 (m, 2H), 3.29 (s, 1H), 1.91 – 1.79 (br. s, 1H), 1.80 – 1.67 (br. s, 1H), 1.51 – 1.41 (br. s, 1H), 1.41 – 1.30 (m, 1H).

^{13}C NMR (151 MHz, $CDCl_3$): δ 159.2, 157.0 156.2, 135.7, 135.6, 135.1, 128.70, 128.66, 128.6, 128.5, 128.3, 128.1, 127.8, 68.7, 68.6, 68.4, 62.0, 60.3, 59.1, 33.6, 33.1, 29.1, 29.0, 26.3, 26.2.

IR (neat): 1712 (s), 2960 (w), 3033 (s), 3274 (b) cm^{-1}.

$[\alpha]^{25}_D$: -5.4 ° (10 mg/mL in chloroform)

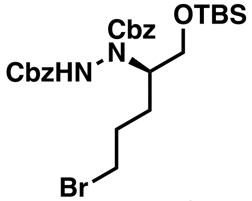

(+)-S5

(+)-S5: To a flask containing **(–)-S4** (7.34 g, 15.8 mmol) and imidazole (11 g, 161.5 mmol) in dimethylformamide (50 mL) was added TBSCl (5.51 g, 36.5 mmol). The reaction was stirred for 16 hours, at which point the reaction was quenched with brine and the aqueous layer was extracted three times with ethyl acetate. The combined organic layers were dried over anhydrous sodium sulfate and concentrated under rotary evaporation. The crude residue was purified by flash chromatography (gradient of 0->100% ethyl acetate in hexanes) to yield pure **(+)-S5** as a white solid in 90% yield (8.22 g, 14.22 mmol). Characterization data matched those previously reported.[3]

¹H NMR (600 MHz, CDCl₃): δ 7.44 – 7.28 (m, 10H), 6.46 (s, 1H), 5.27 – 5.07 (m, 4H), 4.39 – 4.10 (m, 1H), 3.72 – 3.03 (m, 4H), 2.28 – 1.23 (m, 4H), 0.88 – 0.85 (s, 9H), 0.06 (m, 6H).
^{13}C NMR (151 MHz, $CDCl_3$): δ 156.4, 136.0, 135.7, 135.3, 128.6, 128.5, 128.4, 128.3, 128.2, 128.1, 127.9, 127.7, 68.4, 68.0, 67.8, 63.0, 62.4, 59.6, 58.4, 58.3, 45.3, 44.8, 34.7, 34.4, 33.8, 31.6, 29.0, 26.9, 26.7, 25.8, 25.5, 25.3, 22.7, 20.7, 18.0, 14.2, -5.45, -5.53.
IR (neat): 1713 (s), 2856 (w), 2926 (w), 2953 (w), 3284 (b) cm^{-1}.
$[\alpha]^{25}_{D}$: +15 ° (10 mg/mL in chloroform)

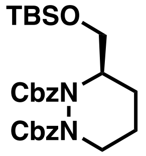

(+)-S6

(+)-S6: To a flask containing sodium hydride (0.70 g, 17.516 mmol, 60% in oil) and dimethylformamide (25 mL) at 0 °C was slowly added a solution of **(+)-S5** (5.06 g, 8.76 mmol) in DMF (5 mL). The reaction was stirred at 0 °C for 50 minutes, at which time it was quenched by dilution into 10% citric acid and the aqueous layer was extracted three times with ethyl acetate. The combined organic layers were washed with brine, dried over anhydrous sodium sulfate, and concentrated by rotary evaporation. The crude residue was purified by flash chromatography (gradient of 0->100% ethyl acetate in hexanes) to yield pure **(+)-S6** as a white solid in 90% yield (3.93 g, 7.89 mmol). Characterization data matched those previously reported.[3]
^{1}H NMR (mixture of rotamers) (600 MHz, $CDCl_3$): δ 7.41 – 7.21 (m, 10H), 5.29 – 4.92 (m, 4H), 4.45 – 4.22 (m, 1H), 4.19 + 4.06 (m, 1H), 3.86 + 3.72 (dd x 2, J = 10.1, 5.2 Hz, 1H), 3.62 + 3.51 (t x 2, J = 10.0 Hz, 1H), 3.23 – 2.95 (m, 1H), 1.93 – 1.79 (m, 2H), 1.76 – 1.66 (m, 1H), 1.55 – 1.46 (m, 1H), 0.89 (s x 2, 9H), 0.09 – 0.04 (s x 2, 6H).
^{13}C NMR (151 MHz, $CDCl_3$): δ 155.6, 155.4, 155.0, 136.3, 136.1, 128.6, 128.5, 128.5, 128.2, 128.1, 127.9, 127.8, 127.7, 127.5 67.8, 67.6, 60.8, 54.3, 45.9, 45.4, 45.0, 44.5, 25.9, 25.8, 22.4, 19.3, 18.9, 18.2, -5.4, -5.4, -5.48, -5.54.
IR (neat): 1709 (s), 2361 (w), 2856 (w), 2953 (w) cm^{-1}.
$[\alpha]^{25}_{D}$: +19.3 ° (10 mg/mL in chloroform)

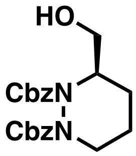

(–)-S7

(–)-S7: To a flask containing **(+)-S6** (3.89 g, 7.81 mmol) and tetrahydrofuran (40 mL) at 0 °C was added slowly a 1M solution of TBAF in THF (9.43 mL, 9.43 mmol). The reaction was stirred for 50 minutes at which time it was quenched by dilution into brine and extracted three times with ethyl acetate. The combined organic layers were washed with brine, dried over anhydrous sodium sulfate, and concentrated by rotary evaporation. The crude residue was purified by flash chromatography (gradient of 0->100% ethyl acetate in hexanes) to yield pure **(–)-S7** as an amorphous solid in quantitative yield (3.0 g, 7.81 mmol). Characterization data matched those previously reported.[3]

^{1}H NMR (600 MHz, CDCl$_3$): δ 7.39 – 7.31 (m, 10H), 5.30 – 5.07 (m, 4H), 4.52 (d, *J* = 53.6 Hz, 1H), 4.24 – 4.05 (m, 1H), 3.66 (dt, J = 60.9, 11.0 Hz, 1H), 3.56 – 3.39 (m, 1H), 3.27 – 3.01 (m, 1H), 2.09 (d, J = 7.8 Hz, 1H), 1.83 – 1.68 (m, 2H), 1.58 – 1.47 (m, 2H).
^{13}C NMR (151 MHz, CDCl$_3$): δ 156.8, 156.2, 155.7, 154.9, 136.2, 135.8, 135.7, 135.4, 128.71, 128.66, 128.6, 128.54, 128.46, 128.3, 128.2, 128.1, 127.8, 127.6, 68.5, 68.4, 68.2, 68.0, 60.6, 60.0, 56.2, 55.0, 45.8, 45.4, 23.1, 22.6, 19.7, 19.4.
IR (neat): 1705 (s), 2947 (w), 3477 (b) cm^{-1}.
[α]$^{25}_{D}$: -7.2 ° (10 mg/mL in chloroform)

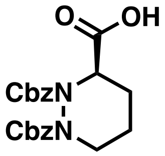

(+)-S8

(+)-S8: To a solution of **(–)-S7** (3.0 g, 7.81 mmol) in acetonitrile (40 mL) was added TEMPO (0.193 g, 1.23 mmol) and sodium chlorite (1.42 g, 15.7 mmol) as solids and 1M pH 6.4 phosphate buffer (40 mL). Sodium hypochlorite was diluted in water to 1.6 M and added (1 mL, 1.6 mmol), at which point a purple color was observed, and the reaction was stirred for 4 hours. The reaction was quenched with 1M sodium hydroxide (20 mL) followed by 1M sodium sulfite. The reaction was adjusted to pH 3 by addition of 1M sodium hydrogen sulfate and the aqueous layer was extracted three times with ethyl acetate. The combined organic layers were washed with brine, dried over anhydrous sodium sulfate, and concentrated by rotary evaporation. The crude residue was purified by flash chromatography (gradient of 0->100% ethyl acetate in hexanes) to yield pure **(+)-S8** as pale oil in 83% yield (2.57 g, 6.52 mmol). Characterization data matched those previously reported.[3]
^{1}H NMR (mixture of rotamers) (600 MHz, CDCl$_3$): δ 7.46 – 7.21 (m, 10H), 5.32 – 5.02 (m, 4H), 4.23 – 4.04 (m, 1H), 3.17 – 2.89 (m, 2H), 2.29 – 2.24 (m, 1H), 1.94 (dddd, *J* = 14.2, 10.6, 6.5, 4.1 Hz, 1H), 1.87 – 1.69 (m, 1H), 1.66 – 1.58 (m, 1H).
^{13}C NMR (151 MHz, CDCl$_3$): δ 171.2, 170.7, 135.4, 135.2, 134.9, 128.74, 128.70, 128.67, 128.6, 128.4, 128.24, 128.16, 128.0, 69.4, 69.2, 69.0, 68.5, 44.9, 43.4, 24.0, 23.8, 20.4, 20.1.
IR (neat): 1721 (s), 2334 (w), 2361 (w), 2954 (w), 3033 (w) cm^{-1}.
[α]$^{25}_{D}$: +30.5 ° (10 mg/mL in chloroform)

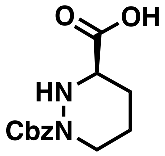

(+)-12

(+)-12: To a flask containing **(+)-S8** (2.57 g, 6.52 mmol) in THF (50 mL) was added freshly pulverized potassium hydroxide (1.82 g, 32.48 mmol). The reaction was stirred for 12 hours, after which time a gel like consistency was observed. The reaction was partitioned between saturated sodium bicarbonate and hexanes and the aqueous layer was washed twice with hexanes. The aqueous layer was then acidified to pH 4 with concentrated hydrochloric acid and extracted three times with ethyl acetate, maintaining pH throughout extraction. The combined organic layers were concentrated by rotary evaporation and the residue was recrystallized in dichloromethane to yield

(+)-12 as an off-white solid in 91% semi-pure yield (1.56 g, 5.93 mmol). Although the material was resistant to further purification, characterization data qualitatively matched those previously reported.[4]

^{1}H NMR (600 MHz, DMSO-*d*$_6$): 7.40 – 7.29 (m, 5H), 5.13 – 5.05 (m, 2H), 3.82 (d, *J* = 13.2 Hz, 1H), 3.36 (dd, *J* = 9.7, 3.1 Hz, 1H), 3.07 (s, 1H), 1.90 (dq, *J* = 8.9, 4.4, 3.9 Hz, 1H), 1.69 (m, 1H), 1.54 (m, 2H).

^{13}C NMR (151 MHz, DMSO-*d*$_6$): δ 173.2, 155.2, 137.5, 128.8, 128.7, 128.5, 128.3, 128.0, 127.1, 126.9, 66.7, 58.4, 40.4, 40.3, 40.1, 40.0, 39.9, 39.7, 39.6, 27.6, 23.4.

IR (neat): 1721 (s), 2948 (w) cm^{-1}.

[α]$^{25}_{D}$: +33 ° (10 mg/mL in methanol)

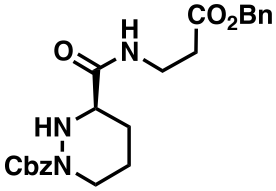

(+)-13

(+)-13: To a flask containing EDC (2.36 g, 12.37 mmol), HOBt (1.94 g, 12.37 mmol) and β-alanine benzyl ester tosylate salt (4.34 g, 12.37 mmol) at 0 °C was added a solution of **(+)-12** (1.08 g, 4.12 mmol) in dimethylformamide (20 mL). Triethylamine (2.2 mL, 15.8 mmol) was added slowly and pH was confirmed to be basic before stirring at 0 °C to room temperature for 12 hours. The reaction was quenched by dilution in saturated sodium bicarbonate and the aqueous layer was extracted three times with ethyl acetate. The combined organic layers were washed with brine, dried over anhydrous sodium sulfate, and concentrated by rotary evaporation. The crude residue was purified by flash chromatography (gradient of 0->100% ethyl acetate spiked with 5% methanol in hexanes) to yield pure **(+)-13** as pale oil in 77% yield (1.38 g, 3.18 mmol). Enantiomeric excess, as determined by chiral HPLC, was measured to be greater than 97%. HPLC (OJ-H, 2-propanol/n-hexane = 10/90, flow rate = 1.0 mL/min, λ = 254 nm) t_R = 9.9 min (major), 12.8 min (minor).

^{1}H NMR (600 MHz, CDCl$_3$): δ 7.41 – 7.30 (m, 10H), 5.17 (s, 2H), 5.14 (s, 2H), 3.93 (d, *J* = 13.3 Hz, 1H), 3.50 (d, *J* = 19.6 Hz, 2H), 3.17 (br. s, 1H), 2.50 (br. s, 2H), 2.33 – 2.25 (m, 1H), 1.73 – 1.53 (m, 3H).

^{13}C NMR (151 MHz, CDCl3): δ 171.6,171.6, 170.7, 136.3, 135.9, 128.6, 128.6, 128.4, 128.3, 128.3, 128.3, 67.8, 66.6, 66.4, 58.0, 35.0, 34.1, 25.4, 21.7.

IR (neat): 1668 (s), 1701 (s), 1733 (s), 2946 (w), 3274 (b) cm^{-1}.

[α]$^{25}_{D}$: +25 ° (10 mg/mL in chloroform)

HRMS (ESI) *m*/*z*: $[M+H]^+$ calcd for $C_{23}H_{28}N_3O_5$ 426.2029, found 426.2012.

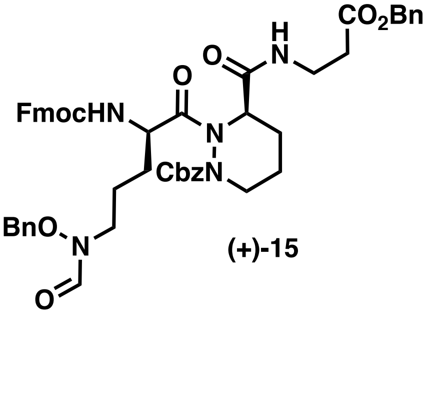

(+)-15

(+)-15: To a flask containing **(–)-14** (1.47 g, 3.01 mmol) and dichloromethane (5 mL) at 0 °C was added dropwise Ghosez's reagent (0.56 mL, 4.21 mmol). The reaction was stirred at 0 °C for 45 minutes, at which point it was transferred to a flask containing **(+)-13** (0.425 g, 1.00 mmol), silver cyanide (0.135 g, 1.00 mmol), and benzene (10 mL). The reaction was brought to 80 °C (oil bath) and stirred at reflux for 50 minutes. The reaction was allowed to cool to room temperature and quenched by dilution in saturated sodium bicarbonate. The aqueous layer was extracted three times with ethyl acetate. The combined organic layers were washed with brine, dried over anhydrous sodium sulfate, and concentrated by rotary evaporation. The crude residue was purified by flash chromatography (gradient of 0->100% ethyl acetate in hexanes) to yield pure **(+)-15** as a white solid in 80% yield (0.717 g, 0.80 mmol).
^{1}H NMR (600 MHz, $CDCl_3$): δ 8.22 (s, 1H), 7.78 (d, J = 7.5 Hz, 2H), 7.68 (s, 1H), 7.58 (d, J = 7.5 Hz, 2H), 7.42 (t, J = 7.4 Hz, 2H), 7.38 – 7.30 (m, 15H), 5.36 (br. s, 1H), 5.19 (s, 2H), 5.11 (s, 2H), 5.08 (m, 1H) 4.95 (s, 1H), 4.81 (br. s, 2H), 4.49 (br. s, 1H), 4.40 (d, J = 6.9 Hz, 2H), 4.22 (t, J = 7.1 Hz, 1H), 4.14 (d, J = 12.7 Hz, 1H), 3.57 – 3.49 (m, 2H), 3.44 – 3.35 (m, 2H), 2.50 (s, 2H), 2.20 (m, 1H), 1.93 (s, 1H), 1.81 (s, 1H), 1.69 (s, 2H), 1.60 (s, 2H), 1.53 (s, 1H).
^{13}C NMR (151 MHz, $CDCl_3$): δ 171.4, 168.9, 163.2, 156.8, 156.2, 143.7, 141.4, 141.3, 135.9, 135.4, 129.5, 128.7, 128.6, 128.6, 128.24, 128.18, 128.0, 127.8, 127.1, 125.1, 120.0, 77.8, 69.1, 67.1, 66.3, 56.0, 47.1, 46.7, 35.2, 33.9, 28.7, 23.5, 20.1.
IR (neat): 1674 (s), 1717 (s), 2949 (w), 3034 (w), 3319 (b) cm^{-1}.
$[\alpha]^{25}_D$: +12.7 ° (10 mg/mL in chloroform)
HRMS (ESI) *m/z*: $[M+H]^+$ calcd for $C_{51}H_{54}N_5O_{10}$ 896.3865, found 896.3853.

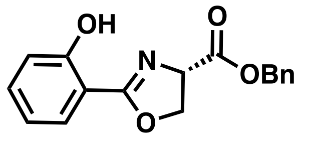

(+)-17

(+)-17: To a flask containing commercially available 2-hydroxybenzonitrile (5.0 g, 41.9 mmol) and methanol (83 mL) was slowly added acetyl chloride (95 mL), resulting in the evolution of heat. The reaction was stirred for 48 hours at which point an orange precipitate was observed. The reaction was brought to 0 °C and adjusted to pH 7 with saturated sodium bicarbonate. The aqueous layer was extracted three times with ethyl acetate and the combined organic layers were washed with water and brine, dried over anhydrous sodium sulfate, and concentrated by rotary evaporation. The crude methyl imidate was taken on without further purification.

To a flask containing L-serine benzyl ester hydrochloride (8.01 g, 34.5 mmol) was added a solution of the crude residue in 1,2-dichloroethane (80 mL). The reaction was brought to 83 °C (oil bath) and stirred at reflux for 12 hours, at which point the reaction was allowed to cool to room temperature and concentrated under rotary evaporation. The residue was partitioned between 5% citric acid and ethyl acetate, and the aqueous layer was extracted three times with ethyl acetate. The combined organic layers were washed with brine, dried over anhydrous sodium sulfate, and concentrated by rotary evaporation. The crude residue was purified by flash chromatography (gradient of 0->100% ethyl acetate in hexanes) to yield pure **(+)-17** as a pale yellow solid in 58% yield (7.22 g, 24.3 mmol).

^{1}**H NMR (600 MHz, CDCl$_3$):** δ 11.55 (s, 1H), 7.54 (d, *J* = 7.8 Hz, 1H), 7.33 – 7.18 (m, 6H), 6.89 (d, *J* = 8.3 Hz, 1H), 6.75 (t, *J* = 7.6 Hz, 1H), 5.15 – 5.07 (m, 3H), 4.89 (ddd, *J* = 9.9, 7.7, 1.5 Hz, 1H), 4.55 (t, *J* = 8.1 Hz, 1H), 4.45 (t, *J* = 9.6 Hz, 1H).
^{13}**C NMR (151 MHz, CDCl$_3$):** δ 170.3, 167.7, 160.0, 135.2, 134.1, 128.7, 128.6, 128.4, 128.3, 118.8, 117.0, 110.1, 68.9, 67.5, 67.4.
IR (neat): 1614 (s), 1637 (s), 1741 (s), 2957 (w), 3031 (w) cm^{-1}.
[α]$^{25}_{D}$: +76 ° (10 mg/mL in chloroform)
HRMS (ESI) *m/z*: [M+H]$^+$ calcd for $C_{17}H_{16}NO_4$ 298.1074, found 298.1075.

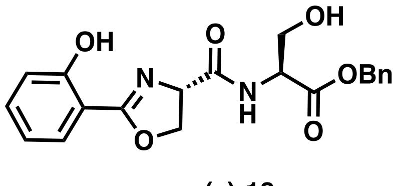

(–)-18

(–)-18: To a flask containing **(+)-17** (0.278 g, 0.935 mmol) and methanol (5 mL) was added 10% palladium on activated charcoal (10 wt % of starting material, 0.028 g). The flask was purged, backfilled with hydrogen gas, and stirred at room temperature until consumption of starting material was observed by TLC. The mixture was passed through a Whatman filter and concentrated by rotary evaporation. The crude acid was taken on without further purification.

To a flask containing EDC (0.536 g, 2.81 mmol), HOBt (0.441 g, 2.81 mmol), and L-serine benzyl ester hydrochloride (0.433 g, 1.87 mmol) at 0 °C was added a solution of crude residue in dimethylformamide (6 mL). Triethylamine (1.0 mL, 7.19 mmol) was added slowly and pH was confirmed to be basic before stirring from 0 °C to room temperature for 16 hours. The reaction was diluted in saturated sodium bicarbonate and the aqueous layer was extracted three times with ethyl acetate. The combined organic layers were washed with brine, dried over anhydrous sodium sulfate, and concentrated by rotary evaporation. The crude residue was purified by flash chromatography (gradient of 0->100% ethyl acetate in hexanes) to yield pure **(–)-18** as a fluffy white solid in 65% yield (0.234 g, 0.61 mmol).
^{1}**H NMR (600 MHz, CDCl$_3$):** δ 11.37 (br. s, 1H), 7.70 (dd, *J* = 7.9, 1.7 Hz, 1H), 7.47 – 7.42 (t, *J* = 7.9, 1H), 7.42 – 7.33 (m, 4H), 7.31 (d, *J* = 7.4 Hz, 1H), 7.05 (dd, *J* = 8.3, 1.1 Hz, 1H), 6.93 (ddd, *J* = 8.2, 7.2, 1.1 Hz, 1H), 5.30 – 5.22 (m, 2H), 5.00 (dd, *J* = 10.9, 8.2 Hz, 1H), 4.74 – 4.69 (m, 1H), 4.71 – 4.67 (m, 1H), 4.66 (dd, *J* = 8.9, 8.3 Hz, 1H), 4.03 (dd, *J* = 11.3, 4.1 Hz, 1H), 3.94 (dd, *J* = 11.3, 3.4 Hz, 1H).
^{13}**C NMR (151 MHz, CDCl$_3$):** δ 171.3, 169.7, 168.0, 159.8, 135.0, 134.4, 128.7, 128.6, 128.6, 128.3, 119.2, 117.0, 110.0, 69.5, 68.1, 68.0, 67.7, 63.1, 54.9, 54.8.
IR (neat): 1614 (w), 1638 (s), 1740 (s), 2362 (w), 2954 (w), 3357 (b) cm^{-1}.
[α]$^{25}_{D}$: -19 ° (10 mg/mL in chloroform)
HRMS (ESI) *m/z*: [M+H]$^+$ calcd for $C_{20}H_{21}N_2O_6$ 385.1394, found 385.1384.

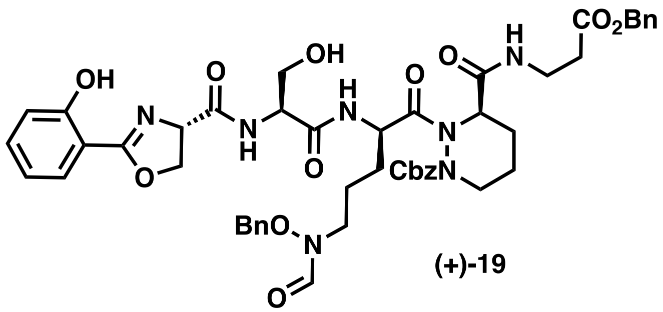

(+)-19

(+)-19: To a flask containing **(+)-15** (0.028 g, 0.03124 mmol) and dichloromethane (0.9 mL) was added 4-(aminomethyl)piperidine (0.1 mL). The reaction was stirred in open air for 30 minutes, diluted in dichloromethane and washed five times with pH 5.5 phosphate buffer and once with brine. The organic layer was dried over anhydrous sodium sulfate and concentrated under rotary evaporation. The crude amine was taken on without further purification.

To a flask containing **(–)-18** (0.008 g, 0.0208 mmol) and methanol (3 mL) was added 10% palladium on activated charcoal (20 wt. % of starting material, 0.002 g). The flask was purged, backfilled with hydrogen gas, and stirred at room temperature until consumption of starting material was observed by TLC. The mixture was passed through a Whatman filter and concentrated by rotary evaporation. The crude acid was taken on without further purification (*note: this crude product is referred to as compound **20** in the main text).

To a flask containing EDC (0.012 g, 0.0623 mmol) and HOBt (0.010 g, 0.0623 mmol) at 0 °C was added a solution of crude acid residue in acetonitrile (3 mL) followed by a solution of crude amine residue in acetonitrile (3 mL). A solution of triethylamine (0.0086 mL, 0.0623 mmol) in acetonitrile (0.1 mL) was added and the pH was confirmed to be basic before stirring from 0 °C to room temperature for 16 hours. The reaction was diluted in saturated sodium bicarbonate and the aqueous layer was extracted three times with ethyl acetate. The combined organic layers were washed with brine, dried over anhydrous sodium sulfate, and concentrated by rotary evaporation. The crude residue was purified by two rounds of flash chromatography (1st: gradient of 0->100% ethyl acetate spiked with 2% methanol in hexanes. 2nd: 0->10% methanol in dichloromethane) to yield pure **(+)-19** as colorless oil in 94% yield (0.0187 g, 0.0197 mmol).
^{1}H NMR (600 MHz, CDCl$_3$): δ 11.32 (br. s, 1H), 8.19 (s, 1H), 7.69 (dd, *J* = 7.9, 1.7 Hz, 1H), 7.46 – 7.41 (t, *J* = 7.9, 1H), 7.35 (q, *J* = 9.7, 8.0 Hz, 16H), 7.02 (dd, *J* = 8.4, 1.1 Hz, 1H), 6.94 – 6.88 (m, 1H), 5.24 – 5.17 (m, 2H), 5.13 (s, 2H), 5.14 – 5.07 (m, 1H), 4.95 (t, *J* = 9.5 Hz, 1H), 4.81 (s, 2H), 4.68 (s, 1H), 4.66 – 4.59 (m, 2H), 4.47 (s, 1H), 4.17 (d, *J* = 13.9 Hz, 1H), 3.97 (d, *J* = 11.4 Hz, 1H), 3.70 – 3.58 (m, 3H), 3.53 (m, 1H), 3.49 – 3.39 (m, 2H), 2.51 (dt, *J* = 8.6, 4.2 Hz, 2H), 2.17 (d, *J* = 8.4 Hz, 1H), 1.92 (s, 1H), 1.87 – 1.71 (m, 3H), 1.71 – 1.52 (m, 3H).
^{13}C NMR (151 MHz, CDCl$_3$): δ 176.1, 171.5, 171.3, 170.9, 169.1, 167.8, 163.5, 159.8, 156.9, 135.8, 135.4, 134.3, 134.0, 129.5, 129.3, 128.9, 128.7, 128.6, 128.6, 128.5, 128.3, 128.2, 128.0, 119.1, 117.0, 110.1, 77.8, 69.4, 69.0, 68.1, 66.3, 62.8, 56.4, 54.6, 53.4, 49.9, 46.5, 43.8, 35.2, 33.9, 31.9, 29.7, 29.4, 27.0, 23.5, 23.4, 22.7, 20.0, 14.1.
IR (neat): 1663 (s), 1733 (w), 2930 (w), 3315 (b) cm^{-1}.
[α]$^{25}_{D}$: +28 ° (10 mg/mL in chloroform)
HRMS (ESI) *m/z*: [M+H]$^+$ calcd for $C_{49}H_{56}N_7O_{13}$ 950.3931, found 950.3899.

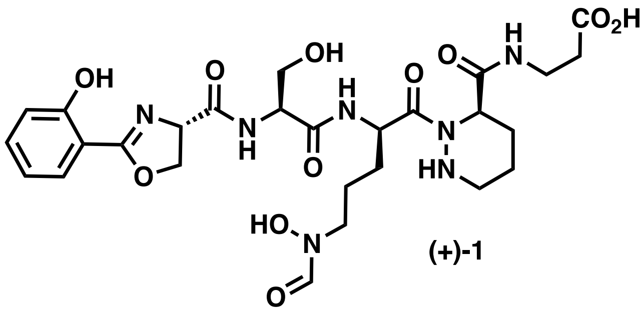

(+)-1

(+)-1: To a flask containing **(+)-19** (0.0139 g, 0.0147 mmol) and methanol (6 mL) was added 10% palladium on activated charcoal (80 wt % of starting material, 0.0111 g). The flask was purged, backfilled with hydrogen gas, and stirred at room temperature until consumption of starting material and partially deprotected intermediates was observed by LCMS. The mixture was passed through a Whatman filter, concentrated by rotary evaporation, and purified by preperatory HPLC (gradient of 5->95% methanol in water over 20 minutes, 0.1% formic acid, t_R = 15.2 min). Fractions containing product were concentrated by lyophilization to yield pure **(+)-1** as a fluffy white powder in 80% yield (0.00749 g, 0.0118 mmol).
Full NMR spectral assignments (in CD_3OD) listed in **Supplementary Table 2**.
IR (neat): 1640 (s), 1657 (s), 2937 (w), 3295 (b) cm^{-1}.
$[\alpha]^{25}_D$: +30 ° (10 mg/mL in methanol)
HRMS (ESI) *m/z*: $[M-H]^+$ calcd for $C_{27}H_{36}N_7O_{11}$ 634.2478, found 634.2478.

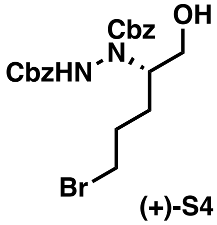

(+)-S4

(+)-S4: Synthesized from commercially available 5-bromo-pentanol (1.78 g, 10.69 mmol) and proportional amounts (by molar equivalents) of all reagents/solvents following the same procedure as **(–)-S4** with the use of D-proline in place of L-proline. Isolated as white solid in 74% yield (1.5297 g, 3.30 mmol).
^{1}H-NMR (600 MHz, $CDCl_3$): δ 7.40 –7.18 (m, 10H), 5.28 – 5.07 (m, 4H), 4.51 – 4.18 (m, 1H), 3.55 – 3.48 (br. s, 1H), 3.48 – 3.33 (m, 2H), 3.29 (s, 1H), 1.91 – 1.79 (br. s, 1H), 1.80 – 1.67 (br. s, 1H), 1.51 – 1.41 (br. s, 1H), 1.41 – 1.30 (br. s, 1H).
^{13}C NMR (151 MHz, $CDCl_3$): δ 157.0, 156.2, 135.7, 135.6, 135.1, 128.7, 128.7, 128.6, 128.5, 128.33, 128.29, 128.1, 127.8 , 68.7, 68.6, 68.4, 62.0, 60.3, 33.6, 33.0, 29.1, 29.0, 26.3, 26.1.
IR (neat): 1711 (s), 2960 (w), 3033 (s), 3280 (b) cm^{-1}.
$[\alpha]^{25}_D$: +5.7 ° (10 mg/mL in chloroform)

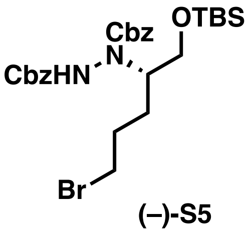

(–)-S5

(–)-S5: Synthesized from **(+)-S4** (1.386 g, 2.98 mmol) and proportional amounts (by molar equivalents) of all reagents/solvents following the same procedure as **(+)-S5**. Isolated as white solid in 82% yield (1.41 g, 2.43 mmol).
^{1}H NMR (600 MHz, CDCl$_3$): δ 7.44 – 7.28 (m, 10H), 6.46 (s, 1H), 5.27 – 5.07 (m, 4H), 4.39 – 4.10 (m, 1H), 3.72 – 3.03 (m, 4H), 2.28 – 1.23 (m, 4H), 0.88 – 0.85 (s, 9H), 0.06 (m, 6H).
^{13}C NMR (151 MHz, CDCl$_3$): δ 156.4, 136.0, 135.7, 135.3, 128.6, 128.5, 128.4, 128.3, 128.2, 128.1, 127.9, 127.7, 68.4, 68.0, 67.8, 63.0, 62.4, 59.6, 58.4, 58.3, 45.3, 44.8, 34.6, 34.4, 33.8, 31.6, 29.0, 26.9, 26.7, 25.8, 25.5, 25.3, 22.7, 20.7, 18.0, 14.2, -5.45, -5.53.
IR (neat): 1712 (s), 1755 (s), 2856 (w), 2928 (w), 2963 (w), 3285 (b) cm^{-1}.
[α]$^{25}_{D}$: -14.4 ° (10 mg/mL in chloroform)

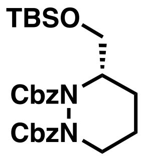

(–)-S6

(–)-S6: Synthesized from **(–)-S5** (1.36 g, 2.359 mmol) and proportional amounts (by molar equivalents) of all reagents/solvents following the same procedure as **(+)-S6**. Isolated as white solid in 96% yield (1.12 g, 2.25 mmol).
^{1}H NMR (mixture of rotamers) (600 MHz, CDCl$_3$): δ 7.41 – 7.21 (m, 10H), 5.29 – 4.92 (m, 4H), 4.45 – 4.22 (m, 1H), 4.19 + 4.06 (m, 1H), 3.86 + 3.72 (dd x 2, *J* = 10.1, 5.2 Hz, 1H), 3.62 + 3.51 (t x 2, *J* = 10.0 Hz, 1H), 3.23 – 2.95 (m, 1H), 1.93 – 1.79 (m, 2H), 1.76 – 1.66 (m, 1H), 1.55 – 1.46 (m, 1H), 0.89 (s x 2, 9H), 0.09 – 0.04 (s x 2, 6H).
^{13}C NMR (151 MHz, CDCl$_3$): δ 155.5, 155.4, 155.0, 136.3, 136.1, 128.6, 128.50, 128.46, 128.2, 128.1, 127.9, 127.8, 127.7, 127.5, 67.8, 67.6, 60.8, 54.3, 45.9, 45.4, 45.0, 44.5, 25.9, 25.8, 22.4, 19.3, 18.9, 18.2, -5.4, -5.45, -5.48, -5.6.
IR (neat): 1707 (s), 2856 (w), 2929 (w) cm^{-1}.
[α]$^{25}_{D}$: -19.6 ° (10 mg/mL in chloroform)

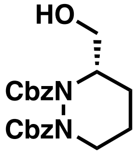

(+)-S7

(+)-S7: Synthesized from **(–)-S6** (1.10 g, 2.21 mmol) and proportional amounts (by molar equivalents) of all reagents/solvents following the same procedure as **(–)-S7**. Isolated as amorphous solid in 97% yield (0.829 g, 2.16 mmol).
^{1}H NMR (600 MHz, CDCl$_3$): δ 7.39 – 7.31 (m, 10H), 5.30 – 5.07 (m, 4H), 4.52 (d, *J* = 53.6 Hz, 1H), 4.24 – 4.05 (m, 1H), 3.66 (dt, J = 60.9, 11.0 Hz, 1H), 3.56 – 3.39 (m, 1H), 3.27 – 3.01 (m, 1H), 2.09 (d, J = 7.8 Hz, 1H), 1.83 – 1.68 (m, 2H), 1.58 – 1.47 (m, 2H).
^{13}C NMR (151 MHz, CDCl$_3$): δ 156.8, 156.2, 155.7, 154.9, 136.2, 135.8, 135.7, 135.4, 128.71, 128.66, 128.6, 128.54, 128.46, 128.3, 128.2, 128.1, 127.8, 127.6, 68.5, 68.4, 68.2, 68.0, 60.6, 60.0, 56.2, 55.0, 45.8, 45.4, 23.1, 22.6, 19.7, 19.4.
IR (neat): 1704 (s), 2361 (s), 2948 (w), 3491 (b) cm^{-1}.
[α]$^{25}_{D}$: +7.5 ° (10 mg/mL in chloroform)

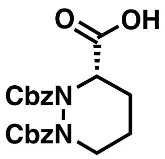

(–)-S8

(–)-S8: Synthesized from **(+)-S7** (0.7807 g, 2.03 mmol) and proportional amounts (by molar equivalents) of all reagents/solvents following the same procedure as **(+)-S8**. Isolated as pale oil in 82% yield (0.657 g, 1.67 mmol).

^{1}H NMR (mixture of rotamers) (600 MHz, $CDCl_3$): δ 7.46 – 7.21 (m, 10H), 5.32 – 5.02 (m, 4H), 4.23 – 4.04 (m, 1H), 3.17 – 2.89 (m, 2H), 2.29 – 2.24 (m, 1H), 1.94 (dddd, *J* = 14.2, 10.6, 6.5, 4.1 Hz, 1H), 1.87 – 1.69 (m, 1H), 1.66 – 1.58 (m, 1H).

^{13}C NMR (151 MHz, $CDCl_3$): δ 171.2, 170.7, 135.4, 135.2, 134.9, 128.74, 128.70, 128.67, 128.6, 128.4, 128.2, 128.2, 128.0, 69.4, 69.2, 69.0, 68.5, 44.9, 43.4, 23.9, 23.8, 20.4, 20.1.

IR (neat): 1707 (s), 2954 (w) cm^{-1}.

$[\alpha]^{25}_D$: -30.4 ° (10 mg/mL in chloroform)

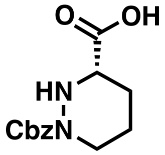

(–)-12

(–)-12: Synthesized from **(–)-S8** (0.529 g, 1.33 mmol) and proportional amounts (by molar equivalents) of all reagents/solvents following the same procedure as **(+)-12**. Isolated as an off white solid in 94% semi-pure yield (0.33 g, 1.25 mmol). Although the material was resistant to further purification, characterization data qualitatively matched those previously reported.

^{1}H NMR (600 MHz, DMSO-d_6): 7.40 – 7.29 (m, 5H), 5.13 – 5.05 (m, 2H), 3.82 (d, *J* = 13.2 Hz, 1H), 3.36 (dd, *J* = 9.7, 3.1 Hz, 1H), 3.07 (s, 1H), 1.90 (dq, *J* = 8.9, 4.4, 3.9 Hz, 1H), 1.69 (m, 1H), 1.54 (m, 2H).

^{13}C NMR (151 MHz, DMSO-d_6): δ 173.2, 155.2, 137.5, 128.8, 128.7, 128.5, 128.3, 128.0, 127.1, 126.9, 66.7, 58.4, 40.4, 40.3, 40.1, 40.0, 39.9, 39.7, 39.6, 27.6, 23.4.

IR (neat): 1731 (s), 2951 (w) cm^{-1}.

$[\alpha]^{25}_D$: -31 ° (10 mg/mL in methanol)

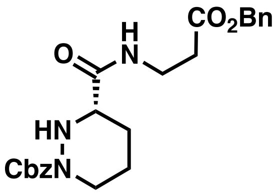

(–)-13

(–)-13: Synthesized from **(–)-12** (0.323 g, 1.224 mmol) and proportional amounts (by molar equivalents) of all reagents/solvents following the same procedure as **(+)-13**. Isolated as a pale oil in 61% yield (0.320 g, 0.7529 mmol). Enantiomeric excess, as determined by chiral HPLC, was measured to be greater than 95%. HPLC (OJ-H, 2-propanol/n-hexane = 10/90, flow rate = 1.0 mL/min, λ = 254 nm) t_R = 10.2 min (minor), 12.5 min (major).

^{1}H NMR (600 MHz, CDCl$_3$): δ 7.41 – 7.30 (m, 10H), 5.17 (s, 2H), 5.14 (s, 2H), 3.93 (d, *J* = 13.3 Hz, 1H), 3.50 (d, *J* = 19.6 Hz, 2H), 3.17 (br. s, 1H), 2.50 (br. s, 2H), 2.33 – 2.25 (m, 1H), 1.73 – 1.53 (m, 3H).
^{13}C NMR (151 MHz, CDCl3): δ 171.6, 171.6, 170.7, 136.3, 135.9, 128.6, 128.6, 128.4, 128.3, 128.3, 128.3, 67.8, 66.6, 66.4, 58.0, 35.0, 34.1, 25.4, 21.7.
IR (neat): 1669 (s), 1704 (s), 1734 (s), 2853 (w), 2921 (w) cm^{-1}.
[α]$^{25}_{D}$: -24 ° (10 mg/mL in chloroform)
HRMS (ESI) *m/z*: [M+H]$^+$ calcd for $C_{23}H_{28}N_3O_5$ 426.2029, found 426.2012.

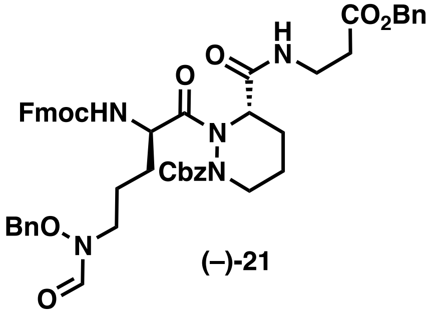

(–)-21

(–)-21: Synthesized from **(–)-13** (0.0967 g, 0.228 mmol), **(–)-14** (0.333 g, 0.683 mmol), and proportional amounts (by molar equivalents) of all reagents/solvents following the same procedure as **(+)-15**. Isolated as a white foam in 90% yield (0.183 g, 0.205 mmol).
^{1}H NMR (600 MHz, CDCl$_3$): δ 8.20 (s, 1H), 7.76 (d, J = 7.5 Hz, 2H), 7.58 (dd, J = 19.1, 7.5 Hz, 2H), 7.40 (t, J = 7.2 Hz, 2H), 7.37 – 7.28 (m, 16H), 5.67 (s, 1H), 5.15 (m, 3H), 5.07 (s, 2H), 4.96 (br. s, 1H), 4.79 (br. s, 3H), 4.38 (s, 2H), 4.31 (s, 1H), 4.19 (m, 2H), 3.66 (s, 1H), 3.51 (s, 2H), 3.33 (m, 1H) 3.04 (m, 1H), 2.45 (m, 2H), 2.22 (m, 1H), 1.72 (m, 4H) 1.66 (s, 3H), 1.46 (m, 1H).
^{13}C NMR (151 MHz, CDCl$_3$): δ 171.5, 163.0, 155.7, 143.7, 141.34, 141.29, 135.8, 129.5, 128.8, 128.7, 128.5, 128.3, 127.7, 127.1, 125.2, 120.0, 69.4, 67.0, 66.436, 50.7, 47.2, 43.3, 33.7, 22.2, 20.2.
IR (neat): 1673 (s), 1726 (s), 2947 (w), 3323 (w) cm^{-1}.
[α]$^{25}_{D}$: -12.9 ° (10 mg/mL in chloroform)
HRMS (ESI) *m/z*: [M+H]$^+$ calcd for $C_{51}H_{54}N_5O_{10}$ 896.3865, found 896.3871.

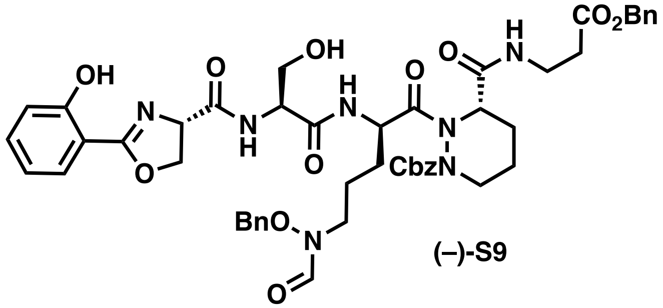

(–)-S9

(–)-S9: Synthesized from **(–)-21** (0.100 g, 0.112 mmol) and **(–)-18** (0.0429 g, 0.112 mmol) and proportional amounts (by molar equivalents) of all reagents/solvents following the same procedure as **(+)-19**. Isolated as a white foam in 62% yield (0.0662 g, 0.0698 mmol).
^{1}H NMR (600 MHz, CDCl$_3$): δ 11.34 (s, 1H), 8.17 (s, 1H), 7.67 (dd, J = 7.9, 1.7 Hz, 1H), 7.59 (s, 1H), 7.43 – 7.27 (m, 16H), 7.11 (s, 1H), 6.98 (d, J = 8.4 Hz, 1H), 6.91 – 6.85 (t, J = 7.9, 1H), 5.19 (m, 1H), 5.12 (s, 3H), 5.04 (s, 1H), 4.95 (t, J = 9.6 Hz, 1H), 4.89 – 4.76 (m, 2H), 4.71 – 4.58 (m,

2H), 4.53 (s, 1H), 4.15 (d, J = 31.0 Hz, 1H), 3.72 – 3.58 (m, 2H), 3.57 – 3.37 (m, 3H), 3.37 – 3.07 (m, 1H), 2.53 (s, 2H), 2.05 (s, 1), 1.94 – 1.78 (m, 1H), 1.78 – 1.55 (s, 6H), 1.54 – 1.43 (m, 1H).
^{13}C NMR (151 MHz, $CDCl_3$): δ 172.3, 171.3, 169.8, 168.6, 163.1, 159.9, 157.1, 135.8, 134.9, 129.5, 129.3, 128.8, 128.7, 128.5, 128.2, 128.2, 119.0, 117.1, 110.0, 69.6, 69.3, 68.0, 66.6, 62.6, 54.6, 48.0, 47.1, 43.3, 35.1, 33.8, 29.2, 23.3, 22.4, 19.9.
IR (neat): 1665 (s), 1729 (s), 2946 (w), 3325 (w) cm^{-1}.
$[\alpha]^{25}_{D}$: -4.6 ° (10 mg/mL in chloroform)
HRMS (ESI) *m/z*: $[M+H]^+$ calcd for $C_{49}H_{56}N_7O_{13}$ 950.3931, found 950.3937.

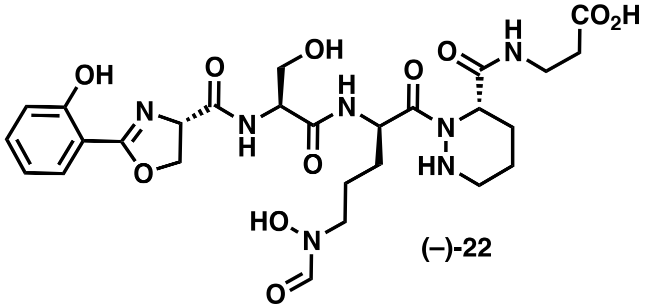

(–)-22

(–)-22: Synthesized from **(–)-S9** (0.020 g, 0.02107 mmol) and proportional amounts (by molar equivalents) of all reagents/solvents following the same procedure as **(+)-1**. Isolated as a white solid in 86% yield (0.0115 g, 0.01811 mmol).
Full NMR spectral assignments (in CD_3OD) listed in **Supplementary Table 3**.
IR (neat): 1657 (s), 2935 (w), 3269 (b) cm^{-1}.
$[\alpha]^{25}_{D}$: -3.4° (10 mg/mL in methanol)
HRMS (ESI) *m/z*: $[M-H]^+$ calcd for $C_{27}H_{36}N_7O_{11}$ 634.2478, found 634.2487.

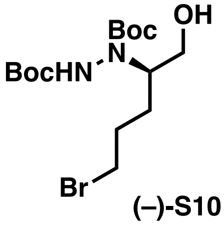

(–)-S10

(–)-S10: To a flask containing PCC (2.76 g, 12.8 mmol) and silica gel (10 g) in dichloromethane (50 mL) was added a solution of commercially available 5-bromo-pentanol (1.78 g, 10.7 mmol) in dichloromethane (15 mL) at a rate of 0.5 mL/minute. The reaction was stirred for 2 hour and then filtered through celite and concentrated by rotary evaporation. The residue was redissolved in dichloromethane, passed through a silica plug, and concentrated by rotary evaporation. The crude aldehyde was taken on without further purification.

A flask containing the crude material in acetonitrile (32 mL) was brought to 0 °C and di-*tert*-butyl azodicarboxylate (1.026 g, 4.46 mmol) and L-proline (0.051 g, 0.443 mmol) were added as solids. The reaction was stirred at 0 °C for 16 hours, at which point sodium borohydride (0.168 g, 4.44 mmol) and ethanol (13 mL) were added and the reaction was stirred for 40 minutes. The reaction was quenched with 10% citric acid and solvent was removed under rotary evaporation. The residue was partitioned between ethyl acetate and brine and the aqueous layer was extracted three times with ethyl acetate. The combined organic layers were dried over anhydrous sodium sulfate and

concentrated under rotary evaporation. The crude residue was purified by flash chromatography (gradient of 0->100% ethyl acetate in hexanes) to yield pure **(–)-S10** as a white solid in 72% yield (1.28 g, 3.21 mmol). Characterization data matched those previously reported.[3]

^{1}H-NMR (600 MHz, CDCl$_3$): 1H NMR (600 MHz, Chloroform-d) δ 6.32 (s, 1H), 4.39 (s, 1H), 4.23 – 4.19 (m, 1H), 3.54 – 3.34 (m, 4H), 1.85 (m, 2H), 1.48 + 1.45 (s x 2, 18H).

^{13}C NMR (151 MHz, CDCl$_3$): δ 158.5, 158.0, 155.9, 155.2, 82.6, 82.5, 82.3, 81.6, 62.2, 62.1, 57.6, 33.6, 33.0, 29.2, 29.1, 28.2, 28.1, 28.10, 28.07, 26.5, 26.3.

IR (neat): 1709 (s), 2978 (s), 3225 (w) cm^{-1}.

[α]^{25}$_D$: -10.3° (10 mg/mL in chloroform)

HRMS (ESI) *m/z*: [M+Na]$^+$ calcd for $C_{15}H_{29}O_5N_2{}^{79}Br^{23}Na$ 419.1152, found 419.1149.

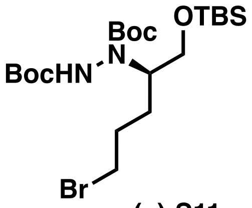

(+)-S11

(+)-S11: Synthesized from **(–)-S10** (1.1 g, 2.75 mmol) and proportional amounts (by molar equivalents) of all reagents/solvents following the same procedure as **(+)-S5**. Isolated as white solid in 81% yield (1.134 g, 2.23 mmol).

^{1}H NMR (600 MHz, CDCl$_3$): δ 6.04 (s, 1H), 4.19 (s, 1H), 4.07 – 3.97 (m, 1H), 3.61 – 3.50 (m, 3H), 3.52 – 3.45 (m, 2H), 3.40 (dt, J = 9.8, 6.5 Hz, 1H), 2.28 – 1.72 (m, 3H), 1.44 (s, 18H), 0.86 (s, 9H), 0.02 (s, 6H).

^{13}C NMR (151 MHz, CDCl$_3$): δ 155.5, 81.4, 80.9, 63.1, 62.4, 59.1, 57.0, 45.0, 34.8, 33.8, 32.4, 32.0, 31.6, 29.0, 28.3, 28.2, 28.1, 27.1, 26.8, 25.9, 25.8, 18.1, 14.1, -5.3, -5.4, -5.48, -5.54.

IR (neat): 1705 (s), 1750 (s), 2929 (s), 2956 (s), 3270 (w) cm^{-1}.

[α]^{25}$_D$: +6.3 ° (10 mg/mL in chloroform)

HRMS (ESI) *m/z*: [M+Na]$^+$ calcd for $C_{21}H_{43}O_5N_2{}^{79}Br^{23}Na^{28}Si$ 533.2017, found 533.2021.

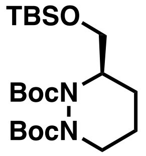

(+)-S12

(+)-S12: Synthesized from **(+)-S11** (1.08 g, 2.12 mmol) and proportional amounts (by molar equivalents) of all reagents/solvents following the same procedure as **(+)-S6**. Isolated as white solid in 90% yield (0.823 g, 1.91 mmol).

^{1}H NMR (600 MHz, CDCl$_3$): δ 4.31 (s, 1H), 4.07 (dq, J = 12.7, 4.7, 4.1 Hz, 1H), 3.95 – 3.79 (m, 1H), 3.74 (dd, J = 9.8, 5.1 Hz, 1H), 3.56 (t, J = 10.2 Hz, 1H), 3.04 – 2.84 (m, 1H), 1.88 – 1.74 (m, 2H), 1.69 – 1.59 (m, 1H) 1.52 – 1.38 (m, 18H), 0.88 (s, 9H), 0.05 (s, 6H).

^{13}C NMR (151 MHz, CDCl$_3$): δ 154.6, 154.5, 80.7, 80.4, 60.7, 53.3, 43.5, 43.2, 28.34, 28.31, 28.28, 28.2, 25.9, 22.3, 18.9, 18.3, -5.3, -5.4, -5.5.

IR (neat): 1701 (s), 2857 (s), 2930 (s) cm^{-1}.

[α]^{25}$_D$: +20.6 ° (10 mg/mL in chloroform)

HRMS (ESI) *m/z*: $[M+H]^+$ calcd for $C_{21}H_{43}O_5N_2{}^{28}Si$ 431.2936, found 431.2935.

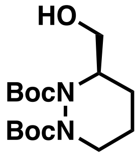

(–)-23

(–)-23: Synthesized from **(+)-S12** (0.802 g, 1.865 mmol) and proportional amounts (by molar equivalents) of all reagents/solvents following the same procedure as **(–)-S7**. Isolated as amorphous solid in 98% yield (0.577 g, 1.83 mmol).

^{1}H NMR (mixture of rotamers) (600 MHz, CDCl$_3$): δ 4.49 – 4.27 (m, 1H), 4.11 – 4.05 (m, 1/2H), 3.95 (m, 1/2H), 3.73 + 3.58 (t x 2, J = 11.1 Hz, 1H), 3.46 (br. s, 1H), 3.05 + 2.91 (br. s x 2, 1H), 2.43 (s, 1H), 1.80 – 1.63 (m, 2H), 1.48 (s, 18H).

^{13}C NMR (151 MHz, CDCl$_3$): δ 155.5, 154.0, 81.8, 60.5, 60.1, 55.9, 53.7, 45.3, 42.8, 28.2, 22.6, 19.8, 19.5.

IR (neat): 1700 (s), 2934 (s), 2976 (s), 3473 (w) cm^{-1}.

$[\alpha]^{25}_D$: -3.5 ° (10 mg/mL in chloroform)

HRMS (ESI) *m/z*: $[M+Na]^+$ calcd for $C_{15}H_{28}O_5N_2{}^{23}Na$ 339.1890, found 339.1886.

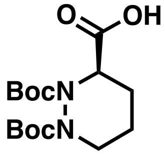

(+)-24

(+)-24: Synthesized from **(–)-23** (0.548 g, 1.73 mmol) and proportional amounts (by molar equivalents) of all reagents/solvents following the same procedure as **(+)-S8**. Isolated as pale oil in 86% yield (0.488 g, 1.48 mmol).

^{1}H NMR (mixture of rotamers) (600 MHz, CDCl$_3$): δ 5.03 – 4.65 (m, 1H), 4.06 + 3.92 (dt x 2, *J* = 12.8, 4.5 Hz, 1H), 3.28 – 2.79 (m, 1H), 2.32 – 2.01 (m, 1H), 1.91 (s, 1H), 1.83 – 1.64 (m, 1H), 1.50 + 1.46 (s x 2, 18H).

^{13}C NMR (151 MHz, CDCl$_3$): δ 171.2, 170.6, 152.5, 83.6, 83.2, 44.2, 42.2, 28.2, 28.10, 28.08, 23.8, 20.3.

IR (neat): 1650 (s), 2970 (s), 2930 (s) cm^{-1}.

$[\alpha]^{25}_D$: +27.6 ° (10 mg/mL in chloroform)

HRMS (ESI) *m/z*: $[M+H]^+$ calcd for $C_{15}H_{27}O_6N_2$ 331.1864, found 331.1862.

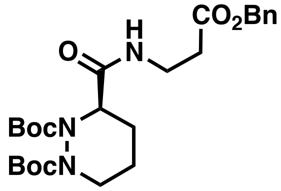

(+)-25

(+)-25: To a flask containing EDC (0.801 g, 4.191 mmol), HOBt (0.658 g, 4.191 mmol), and β-alanine benzyl ester tosylate salt (1.47 g, 4.191 mmol) at 0 °C was added a solution of **(+)-24** (0.461g, 1.397 mmol) in DMF (15 mL). Triethylamine (0.8245 mL, 5.937 mmol) was added and

the pH was confirmed to be basic before stirring from 0 °C to room temperature for 16 hours. The reaction was diluted in saturated sodium bicarbonate and the aqueous layer was extracted three times with ethyl acetate. The combined organic layers were washed with brine, dried over anhydrous sodium sulfate, and concentrated by rotary evaporation. The crude residue was purified by flash chromatography (gradient of 0->100% ethyl acetate in hexanes) to yield pure **(+)-25** as pale oil in 97% yield (0.6679 g, 1.36 mmol).
^{1}H NMR (mixture of rotamers) (600 MHz, CDCl$_3$): δ 7.53 – 7.32 (m, 5H), 5.14 (s, 2H), 4.86 (br. s, 1H), 4.05 – 3.82 (m, 1H), 3.71 – 3.60 (m, 1H), 3.51 (br. s, 1H), 3.09 – 2.77 (m, 1H), 2.71 – 2.55 (m, 2H), 2.31 (br. s, 1H), 1.80 – 1.71 (m, 1H), 1.70 – 1.62 (m, 2H), 1.56 – 1.48 (m, 18H).
^{13}C NMR (151 MHz, CDCl$_3$): δ 171.2, 170.1, 135.9, 135.7, 128.6, 128.5, 128.3, 128.22, 128.21, 128.17, 82.8, 82.0, 66.4, 66.3, 35.4, 35.2, 34.3, 34.2, 28.4, 28.2, 28.1, 20.6, 20.4.
IR (neat): 1678 (s), 1709 (s), 1736 (s), 2976 (s), 3323 (w) cm^{-1}.
[α]$^{25}_{D}$: +14.8 ° (10 mg/mL in chloroform)
HRMS (ESI) *m/z*: [M+H]$^+$ calcd for $C_{25}H_{38}O_7N_3$ 492.2704, found 492.2702.

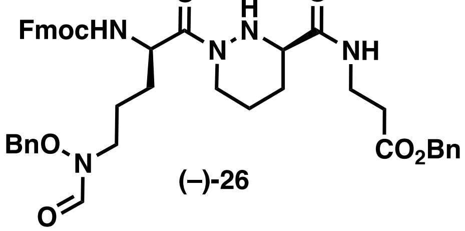

(–)-26

(–)-26: To a vial containing **(+)-25** (0.2689 g, 0.5476 mmol) dissolved in dichloromethane (3 mL) was added trifluoroacetic acid (3 mL). The mixture was stirred for two hours and concentrated by rotary evaporation. The residue was dissolved in toluene and concentrated again (repeat 5x). The residue was taken on without further purification.

To a flask containing EDC (0.251 g, 1.314 mmol), and HOBt (0.206 g, 1.314 mmol) at 0 °C was added a solution of **(–)-14** (0.213 g, 0.43811 mmol) in acetonitrile (3 mL), followed by a solution of crude residue from the previous step in acetonitrile (3 mL). Triethylamine (0.31 mL, 2.19 mmol) was added slowly and pH was confirmed to be basic before stirring from 0 °C to room temperature for 16 hours. The reaction was diluted in saturated sodium bicarbonate and the aqueous layer was extracted three times with ethyl acetate. The combined organic layers were washed with brine, dried over anhydrous sodium sulfate, and concentrated by rotary evaporation. The crude residue was purified by flash chromatography (gradient of 0->100% ethyl acetate s in hexanes) to yield pure **(–)-26** as a white foam in 73% yield (0.234 g, 0.319 mmol).
^{1}H NMR (600 MHz, CDCl$_3$): δ 8.20 (s, 1H), 7.87 (s, 1H), 7.75 (d, J = 7.6 Hz, 2H), 7.59 (d, J = 7.5 Hz, 2H), 7.43 – 7.28 (m, 14H), 7.19 (br. s, 1H) 5.90 (d, J = 7.0 Hz, 1H), 5.62 – 5.50 (m, 1H), 5.21 – 5.08 (m, 2), 5.04 – 4.90 (s, 1H), 4.86 – 4.75 (m, 1H), 4.44 – 4.29 (m, 3H), 4.19 (t, J = 7.1 Hz, 1H), 3.80 – 3.57 (m, 2H), 3.49 (dq, J = 13.4, 6.4 Hz, 2H), 3.35 – 3.19 (m, 1H), 3.18 – 3.00 (m, 1H), 2.85 – 2.48 (m, 2H), 1.87 – 1.05 (m, 8H).
^{13}C NMR (151 MHz, CDCl$_3$): δ 171.5, 170.8, 159.1, 144.1, 143.9, 141.3, 136.0, 134.1, 130.0, 129.3, 128.8, 128.6, 128.3, 127.7, 127.0, 125.2, 119.98, 119.96, 67.0, 66.4, 60.1, 51.0, 47.8, 47.3, 42.1, 35.1, 34.1, 28.0, 22.6, 20.7.
IR (neat): 1660 (s), 1723 (s), 2942 (w), 3322 (w) cm^{-1}.
[α]$^{25}_{D}$: -11.6 ° (10 mg/mL in chloroform)
HRMS (ESI) *m/z*: [M+Na]$^+$ calcd for $C_{43}H_{47}O_8N_5{}^{23}Na$ 784.3317, found 784.3316.

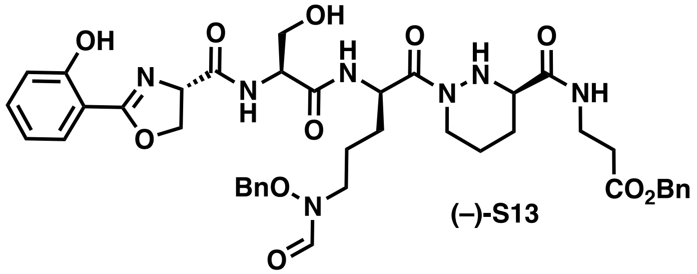

(–)-S13

(–)-S13: Synthesized from **(–)-26** (0.082 g, 0.112 mmol), **(–)-18** (0.0431 g, 0.112 mmol), and proportional amounts (by molar equivalents) of all reagents/solvents following the same procedure as **(+)-19**. Isolated as a white foam in 77% yield (0.0631 g, 0.0774 mmol).

^{1}H NMR (600 MHz, CDCl$_3$): δ 8.16 (s, 1H), 7.86 (s, 1H), 7.67 (dd, J = 7.9, 1.7 Hz, 2H), 7.57 – 7.28 (m, 16H), 7.01 (d, J = 8.4 Hz, 2H), 6.89 (td, J = 7.6, 1.1 Hz, 2H), 5.28 (s, 1H), 5.21 – 5.06 (m, 3H), 5.04 – 4.97 (m, 1H),4.97 – 4.90 (m, 1H) 4.81 (d, J = 10.1 Hz, 2H), 4.67 (d, J = 9.2 Hz, 2H), 4.52 – 4.13 (m, 1H), 4.40 – 4.16 (d, J = 13.3 Hz, 1H), 4.01 (dd, J = 23.5, 11.2 Hz, 2H), 3.90 – 3.75 (m, 1H), 3.68 (s, 2H), 3.63 – 3.46 (m, 3H), 3.35 – 3.22 (m, 1H), 3.19 – 3.09 (m, 1H) 2.73 – 2.61 (m, 2H), 2.60 – 2.50 (m, 1H), 1.94 – 1.46 (m, 8H).

^{13}C NMR (151 MHz, CDCl$_3$): δ 171.6, 170.8, 169.2, 167.9, 163.3, 159.9, 159.1, 135.9, 134.5, 134.0, 129.9, 129.5, 129.3, 128.9, 128.7, 128.6, 128.6, 128.4, 128.3, 119.1, 117.1, 109.8, 76.3, 69.5, 66.5, 66.4, 62.5, 60.0, 54.6, 50.1, 49.7, 47.7, 43.6, 42.2, 35.1, 34.1, 28.9, 28.0, 27.2, 20.8.

IR (neat): 1640 (s), 2941 (w), 3305 (w) cm^{-1}.

[α]$^{25}_{D}$: -5.4 ° (10 mg/mL in chloroform)

HRMS (ESI) *m/z*: $[M+H]^+$ calcd for $C_{41}H_{50}O_{11}N_7$ 816.3563, found 816.3564.

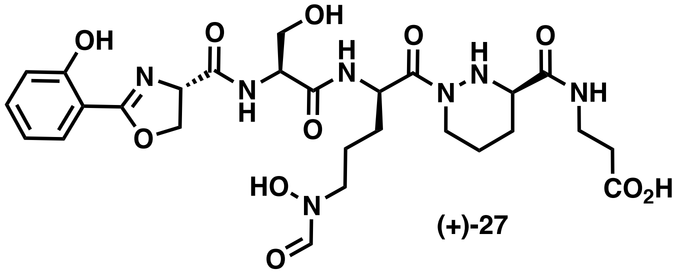

(+)-27

(+)-27: Synthesized from **(–)-S13** (0.020 g, 0.02454 mmol) and proportional amounts (by molar equivalents) of all reagents/solvents following the same procedure as **(+)-1**. Isolated as a white solid in 87% yield (0.0136 g, 0.02142 mmol).

Full NMR spectral assignments (in CD$_3$OD) listed in **Supplementary Table 4**.

IR (neat): 1657 (s), 2930 (w), 3279 (b) cm^{-1}.

[α]$^{25}_{D}$: +15° (10 mg/mL in methanol)

HRMS (ESI) *m/z*: $[M+H]^+$ calcd for $C_{27}H_{36}N_7O_{11}$ 634.2478, found 634.2496.

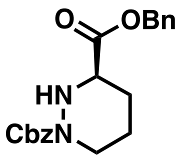

(+)-28

(+)-28: To a flask containing a solution of **(+)-12** (0.357 g, 1.35 mmol) dissolved in DMF (12 mL) was added K_2CO_3 (0.279 g, 2.02 mmol) followed by benzyl bromide (0.290 g, 1.69 mmol). The reaction was allowed to stir for 12 hours, diluted in saturated sodium bicarbonate, and extracted three times with ethyl acetate. The combined organic layers were washed with brine, dried over anhydrous Na_2SO_4, and concentrated by rotary evaporation. The crude residue was purified by flash chromatography (gradient of 0->100% ethyl acetate s in hexanes) to yield pure **(+)-28** as a pale oil in 77% yield (0.368 g, 1.04 mmol).

^{1}H NMR (600 MHz, $CDCl_3$): δ 7.39 – 7.32 (m, 10H), 5.20 – 5.12 (m, 4H), 3.99 (d, *J* = 12.7 Hz, 1H), 3.59 (dd, *J* = 10.2, 3.2 Hz, 1H), 3.13 (d, *J* = 14.4 Hz, 1H), 2.08 (dt, *J* = 12.5, 3.8 Hz, 1H), 1.78 – 1.68 (m, 2H), 1.64 – 1.53 (m, 1H).

^{13}C NMR (151 MHz, $CDCl_3$): δ 170.9, 155.3, 136.4, 135.3, 128.6, 128.5, 128.5, 128.3, 128.2, 128.0, 67.6, 66.8, 58.4, 44.8, 27.5, 23.4.

IR (neat): 1695, (s), 1736 (s), 2946 (w) cm^{-1}.

$[\alpha]^{25}_D$: +24° (10 mg/mL in chloroform)

HRMS (ESI) *m/z*: $[M+H]^+$ calcd for $C_{20}H_{23}O_4N_2$ 355.1652, found 355.1651.

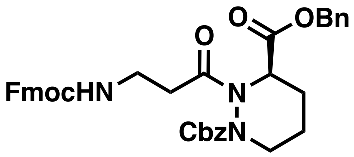

(+)-29

(+)-29: Synthesized from commercially available Fmoc-β-alanine (0.646 g, 2.07 mmol), **(+)-28** (0.245 g, 0.692 mmol), and proportional amounts (by molar equivalents) of all reagents/solvents following the same procedure as **(+)-15**. Isolated as a pale oil in 93% yield (0.420 g, 0.649 mmol).

^{1}H NMR (600 MHz, $CDCl_3$): δ 7.76 (d, J = 7.5 Hz, 2H), 7.58 (d, J = 5.8 Hz, 2H), 7.45 – 7.20 (m, 14H), 5.41 (d, J = 5.2 Hz, 2H), 5.26 – 5.14 (m, 1H), 5.08 (d, J = 12.2 Hz, 1H), 4.95 (s, 1H), 4.34 (d, J = 7.4 Hz, 3H), 4.19 (t, J = 7.0 Hz, 1H), 3.43 (s, 2H), 3.06 – 2.84 (m, 1H), 2.55 (br. s, 2H), 2.11 – 2.02 (m, 2H), 1.87 – 1.76 (m, 1H).

^{13}C NMR (151 MHz, $CDCl_3$): δ 169.3, 156.3, 144.0, 141.3, 135.6, 128.8, 128.7, 128.6, 128.6, 128.4, 128.4, 128.3, 128.1, 127.768, 127.0, 125.14, 125.10, 120.0, 68.4, 66.7, 47.2, 36.4, 32.4, 24.9, 19.3.

IR (neat): 1677 (s), 1720 (s), 2960 (w), 3363 (w) cm^{-1}.

$[\alpha]^{25}_D$: +26.4° (10 mg/mL in chloroform)

HRMS (ESI) *m/z*: $[M+H]^+$ calcd for $C_{38}H_{38}O_7N_3$ 648.2704, found 648.2706.

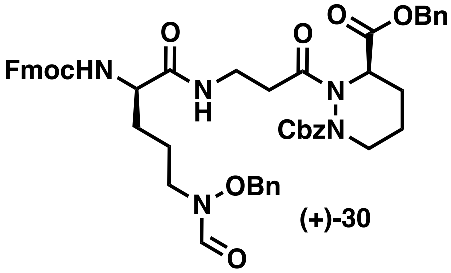

(+)-30

(+)-30: To a flask containing **(+)-29** (0.301 g, 0.0.464 mmol) and dichloromethane (3 mL) was added 4-(aminomethyl)piperidine (0.35 mL). The reaction was stirred in open air for 1.5 hours, diluted in dichloromethane and washed five times with pH 5.5 phosphate buffer and once with brine. The organic layer was dried over anhydrous sodium sulfate and concentrated under rotary evaporation. The crude amine was taken on without further purification.

To a flask containing EDC (0.119 g, 0.624 mmol) and HOBt (0.098 g, 0.624 mmol) at 0 °C was added a solution of **(–)-14** (0.113 g, 0.232 mmol) in acetonitrile (3 mL) followed by a solution of crude amine residue in acetonitrile (3 mL). Triethylamine (0.1 mL, 0.772 mmol) in was added slowly and the pH was confirmed to be basic before stirring from 0 °C to room temperature for 16 hours. The reaction was diluted in saturated sodium bicarbonate and the aqueous layer was extracted three times with ethyl acetate. The combined organic layers were washed with brine, dried over anhydrous sodium sulfate, and concentrated by rotary evaporation. The crude residue was purified by flash chromatography (gradient of 0->100% ethyl acetate in hexanes) to yield pure **(+)-30** as white foam in 78% yield (0.161 g, 0.180 mmol).

^{1}H NMR (600 MHz, CDCl$_3$): δ 8.20 (s, 1H), 7.75 (d, J = 7.5 Hz, 2H), 7.59 (t, J = 8.1 Hz, 2H), 7.44 – 7.24 (m, 19H), 6.68 (s, 1H), 5.55 (s, 1H), 5.35 (d, J = 6.0 Hz, 1H), 5.17 (br. s, 1H), 5.06 (br. s, 1H), 4.93 (br. s, 1H), 4.81 (d, J = 9.5 Hz, 2H), 4.36 (d, J = 7.0 Hz, 2H), 4.24 (s, 1H), 4.20 (t, J = 7.2 Hz, 1H), 3.84 (br. s, 1H), 3.63 – 3.16 (m, 3H) 2.91 (br. s, 1H), 2.48 (m, 2H), 1.99 (d, J = 13.3 Hz, 1H), 1.82 – 1.45 (m, 7H).

^{13}C NMR (151 MHz, CDCl$_3$): δ 171.4, 169.3, 163.5, 143.7, 141.31, 141.30, 135.6, 135.4, 134.2, 129.54, 129.52, 129.3, 128.9, 128.8, 128.60, 128.57, 128.40, 128.36, 128.29, 128.25, 128.2, 127.7, 127.13, 127.10, 125.1, 120.0, 68.4, 67.1, 53.6, 51.0, 47.2, 43.1, 34.9, 31.7, 30.5, 24.9, 23.0, 19.2.

IR (neat): 1673 (s), 1721 (s), 2943 (w), 3323 (w) cm^{-1}.

$[\alpha]^{25}_D$: +18.2° (10 mg/mL in chloroform)

HRMS (ESI) *m/z*: $[M+H]^+$ calcd for $C_{51}H_{54}O_{10}N_5$ 896.3865, found 896.3874.

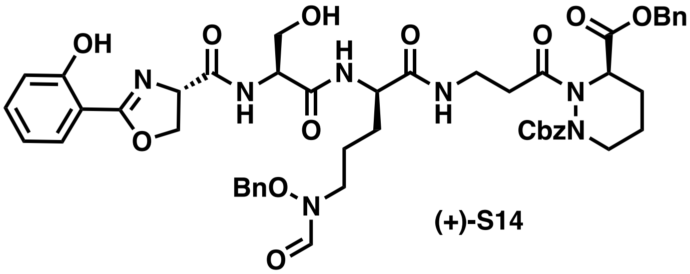

(+)-S14

(+)-S14: Synthesized from **(+)-30** (0.100 g, 0.112 mmol), **(–)-18** (0.0429 g, 0.112 mmol), and proportional amounts (by molar equivalents) of all reagents/solvents following the same procedure as **(+)-19**. Isolated as a white foam in 73% yield (0.0776 g, 0.0818 mmol).

^{1}H NMR (600 MHz, CDCl$_3$): δ 8.13 (s, 1H), 7.67 (dd, J = 7.9, 1.8 Hz, 1H), 7.42 – 7.28 (m, 16H), 7.23 (s, 1H), 7.00 – 6.95 (m, 1H), 6.90 (t, J = 7.5 Hz, 1H), 5.36 (br. s, 1H), 5.19 (d, J = 12.1 Hz, 1H), 5.12 – 5.02 (m, 1H) 4.94 (q, J = 7.5, 5.2 Hz, 2H), 4.81 (s, 2H), 4.64 – 4.59 (m, 1H), 4.53 (t, J = 10.1 Hz, 1H), 4.44 (s, 1H), 4.24 (br. s, 1H), 4.07 (d, J = 10.1 Hz, 1H), 3.69 – 3.53 (m, 3H), 3.47 (d, J = 13.1 Hz, 1H), 3.38 – 3.20 (m, 1H), 3.16 – 2.88 (m, 1H), 2.70 – 2.27 (m, 2H), 2.08 – 1.89 (m, 2H), 1.82 – 1.66 (m, 4H), 1.57 (dq, J = 12.5, 3.6, 3.2 Hz, 2H).
^{13}C NMR (151 MHz, CDCl$_3$): δ 171.3, 170.9, 167.6, 163.7, 159.7, 155.5, 135.2, 134.3, 134.1, 129.5, 129.2, 128.83, 128.75, 128.62, 128.59, 128.56, 128.4, 128.3, 119.1, 116.9, 110.1, 69.3, 68.0, 67.3, 62.7, 43.7, 34.9, 31.5, 27.7, 23.8.
IR (neat): 1640 (s), 1734 (s), 2935 (w), 3315 (w) cm^{-1}.
[α]$^{25}_{D}$: +17.4 ° (10 mg/mL in chloroform)
HRMS (ESI) *m/z*: [M+H]$^+$ calcd for $C_{49}H_{56}O_{13}N_7$ 950.3931, found 950.3938.

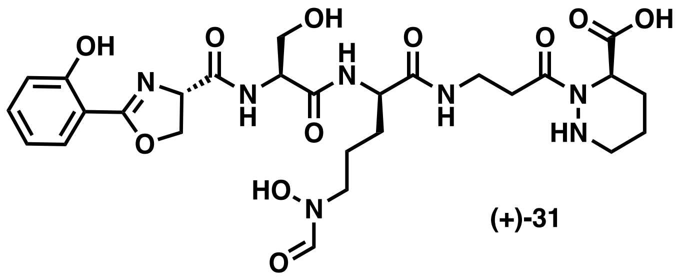

(+)-31

(+)-31: Synthesized from **(+)-S14** (0.020 g, 0.02107 mmol) and proportional amounts (by molar equivalents) of all reagents/solvents following the same procedure as **(+)-1**. Isolated as a white solid in 78% yield (0.0104 g, 0.01638 mmol).
Full NMR spectral assignments (in CD_3OD) listed in **Supplementary Table 5**.
IR (neat): 1658 (s), 2926 (w), 3281 (b) cm^{-1}.
[α]$^{25}_{D}$: +3.4° (10 mg/mL in methanol)
HRMS (ESI) *m/z*: [M+H]$^+$ calcd for $C_{27}H_{38}N_7O_{11}$ 636.2624, found 636.2678.

References

(1) Yokokawa, F.; Sugiyama, H.; Shioiri, T.; Katagiri, N.; Oda, O.; Ogawa, H. An Expeditious Synthesis of Pentosidine, an Advanced Glycation End Product. *Tetrahedron* **2001**, *57* (22), 4759–4766.
(2) Mashiach, R.; Meijler, M. M. Total Synthesis of Pyoverdin D. *Org. Lett.* **2013**, *15* (7), 1702–1705.
(3) Henmi, Y.; Makino, K.; Yoshitomi, Y.; Hara, O.; Hamada, Y. Highly Efficient Synthesis of (R)- and (S)-Piperazic Acids Using Proline-Catalyzed Asymmetric α-Hydrazination. *Tetrahedron Asymmetry* **2004**, *15* (21), 3477–3481.
(4) Chen, Y.; Lu, Y.; Zou, Q.; Chen, H.; Ma, D. A Scalable Process to the Key Intermediate of Cilazapril, (S) - 1- Benzyloxycarbonylhexahydropyridazine-3-Carboxylic Acid, Through a Novel Cascade Course. **2013**, 1209–1213.

6. Biological Procedures

To test the ability of compounds to inhibit growth the growth of *Acinetobacter baumannii* ATCC 17978, a -80 °C glycerol stock was used to inoculate 5 mL sterile cation adjusted Mueller Hinton II broth (hereafter MHII) and the culture was shaken aerobically overnight (37 °C, 200 RPM). The next day, 200 μL of overnight culture was used to inoculate 5 mL of fresh MHII and the regrowth culture was shaken aerobically (37 °C, 200 RPM) until in exponential phase (~4-5 hours). Sterile water was used to dilute fresh MHII to 10% by volume and 100 μL of 10%-MHII was added to each well of a 96-well plate. Each compound was dissolved to 10 mM in DMSO, and this stock was further diluted into 10%-MHII to give 800 μM of each compound (equal volume of pure DMSO was used as a control). To the first column was added 100 μL of compound stock, which was then serially diluted eight columns down the plate to give nine concentrations of each compound, discarding 100 μL from the final column. The regrowth culture was diluted into 10%-MHII to an OD_{600} = 0.004 and 100 μL of this inoculum was added to each well of the plate except for the final column, to which 100 μL/well of blank 10%-MHII was added as a control. At this point, each plate contained 1.) nine inoculated columns of serially diluted compound (or DMSO control) at nine concentrations from 200-0.78 μM, 2.) one column of inoculated media to measure basal growth levels, and 3.) one column of sterile media to measure background signal. Plates were then grown aerobically at 37 °C without shaking for 20 hours and OD_{600} readings were recorded as an average of three independent measurements. The background absorbance of blank media (OD = 0.032) was subtracted from each measurement to give the final values depicted in **Figure S3**.

7. Spectral Data

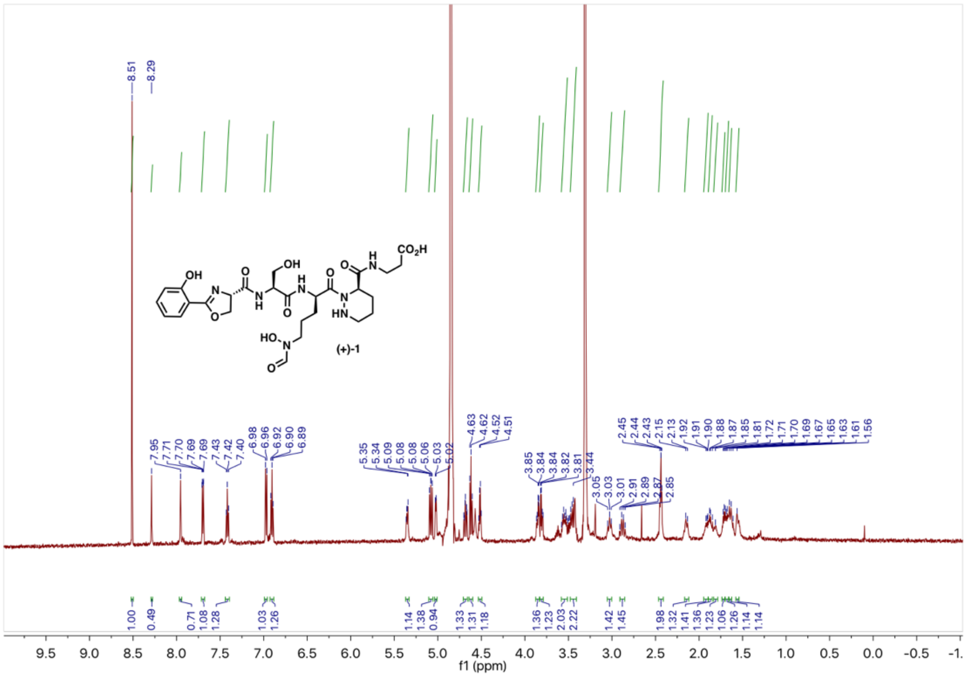

^{1}H NMR (600 MHz, CD_3OD) of (+)-1

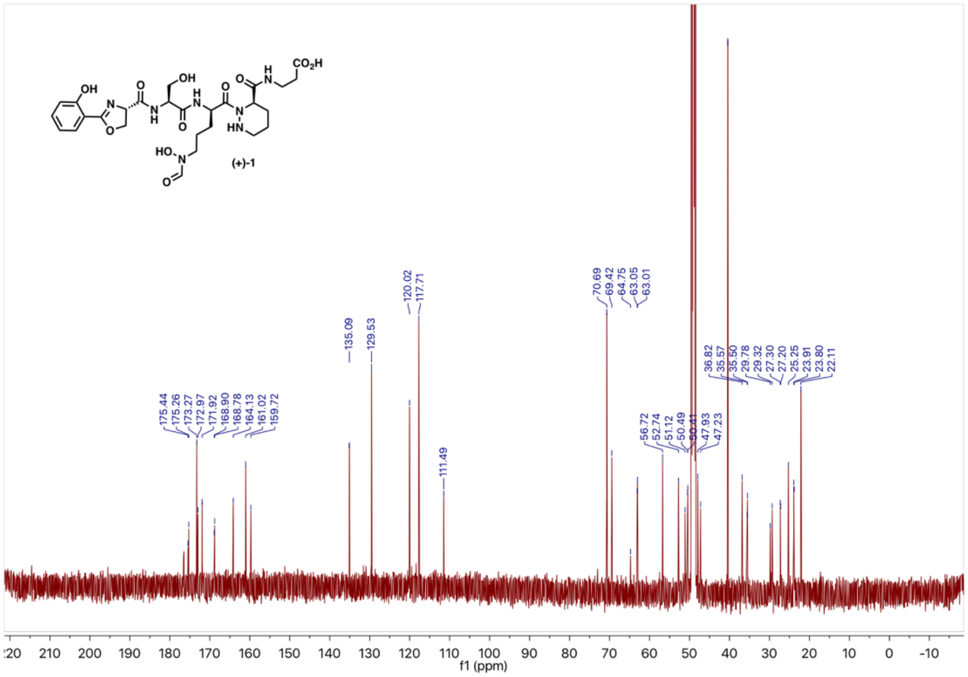

^{13}C NMR (151 MHz, CD_3OD) of (+)-1

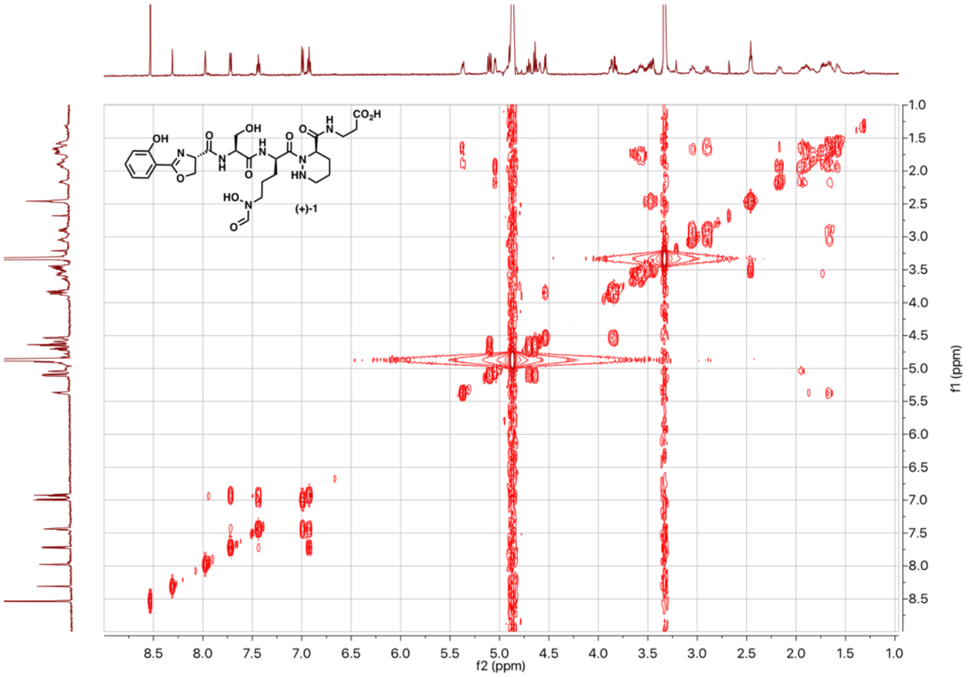

gCOSY (600 MHz, CD_3OD) of (+)-1

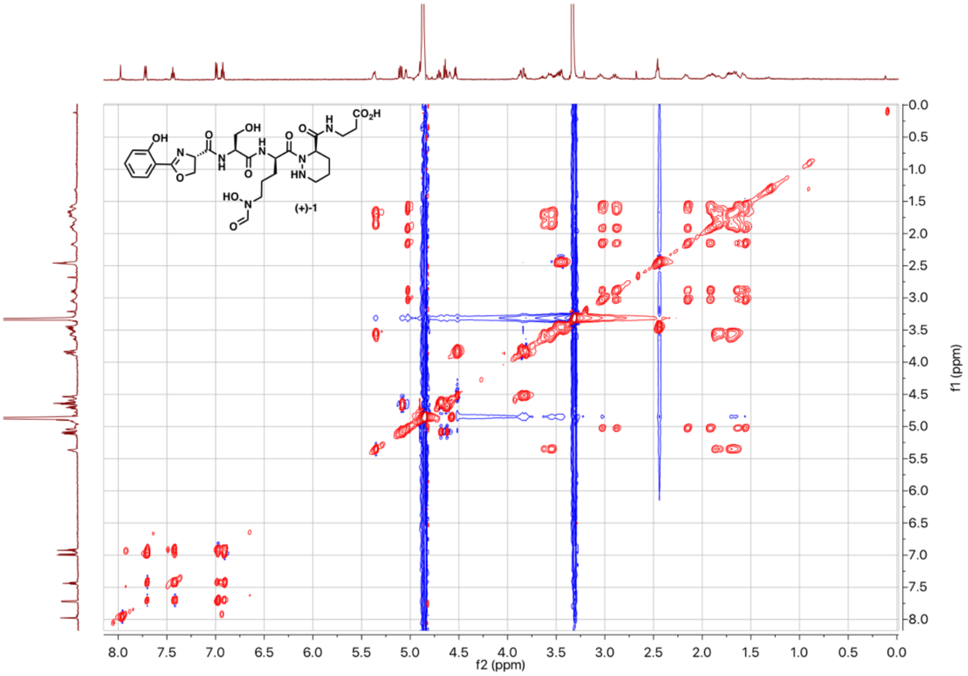

TOCSY (600 MHz, CD_3OD) of (+)-1

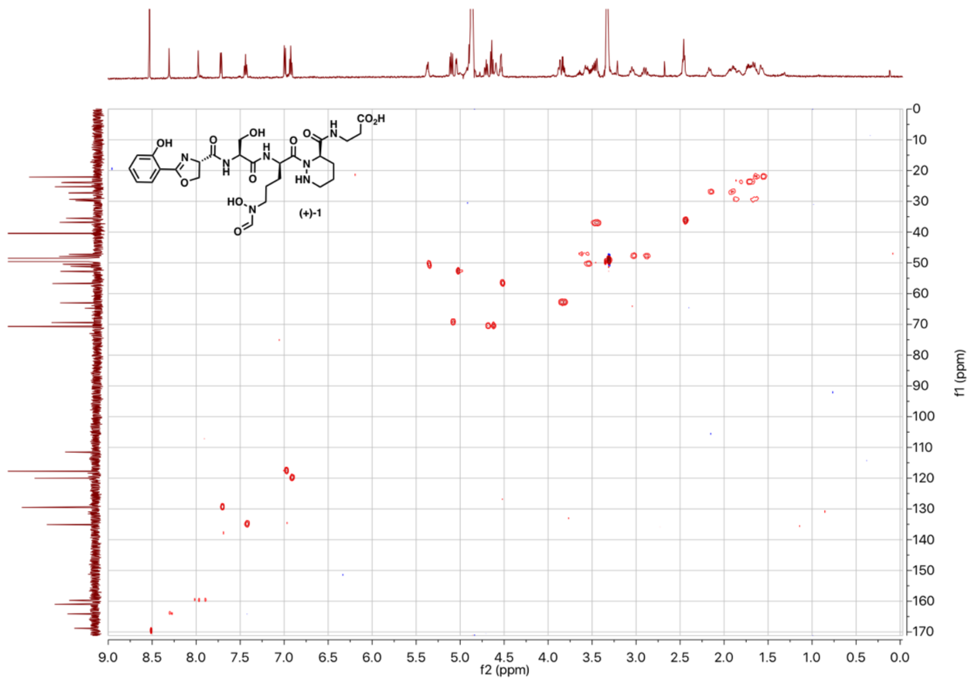

HSQC (600 MHz, CD$_3$OD) of (+)-1

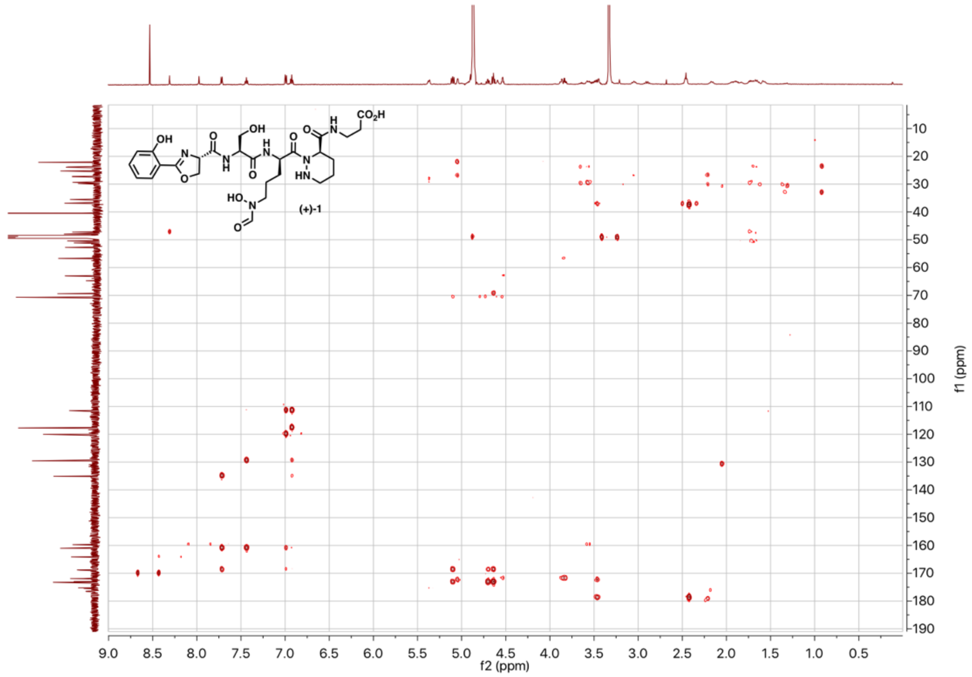

HMBC (600 MHz, CD$_3$OD) of (+)-1

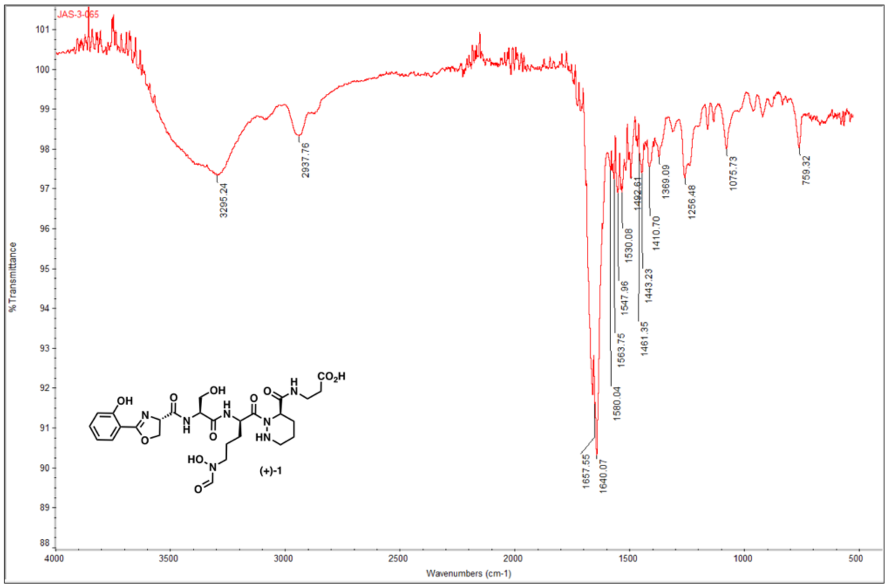

IR Spectrum of (+)-1

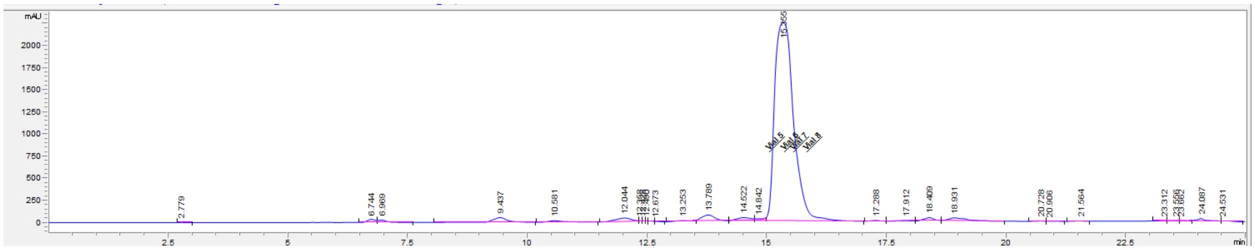

HPLC Trace of (+)-1 After Deprotection from (+)-19

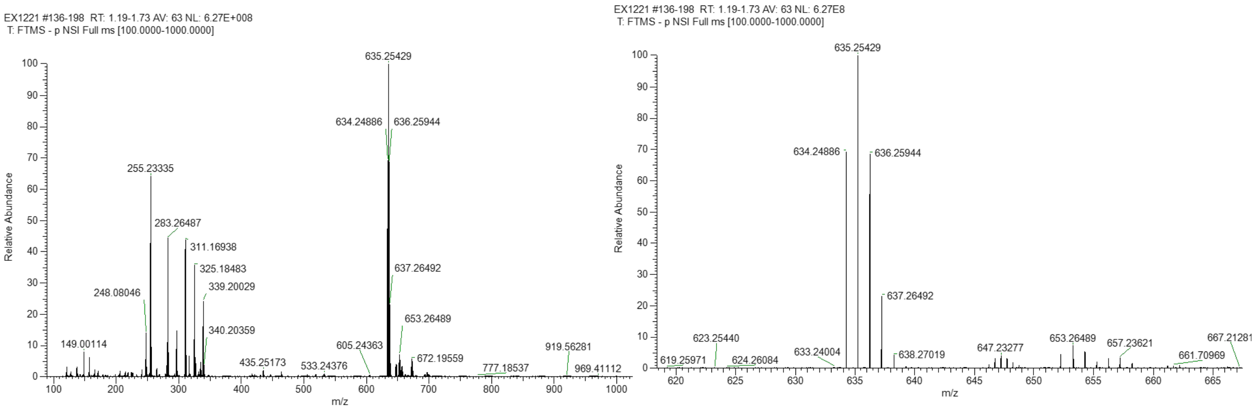

HRMS Spectrum (Negative Ion Mode) of (+)-1

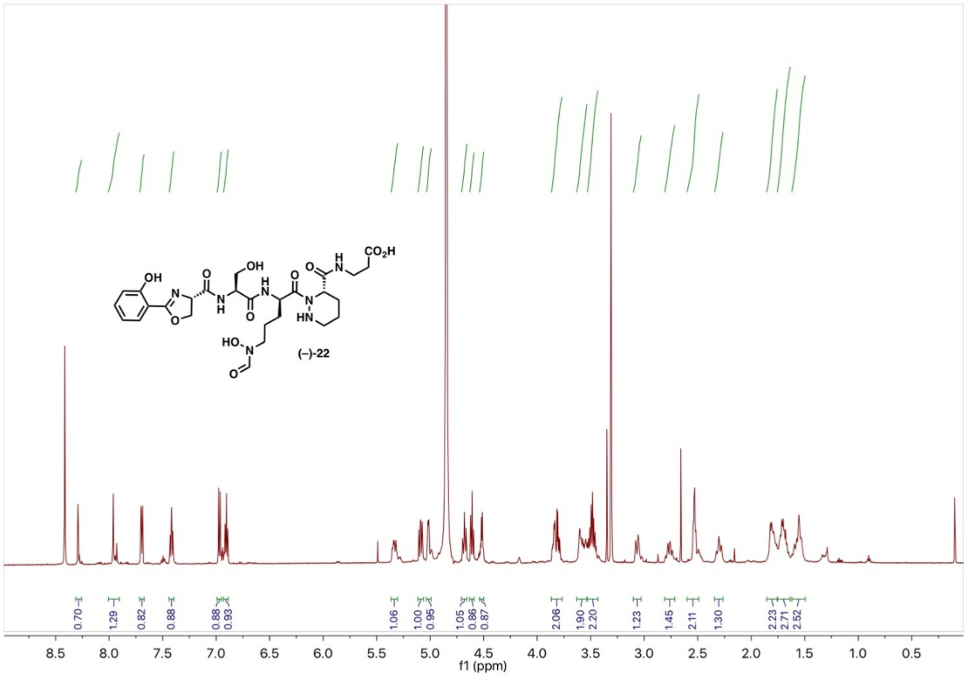

^{1}H NMR (600 MHz, CD_3OD) of (–)-22

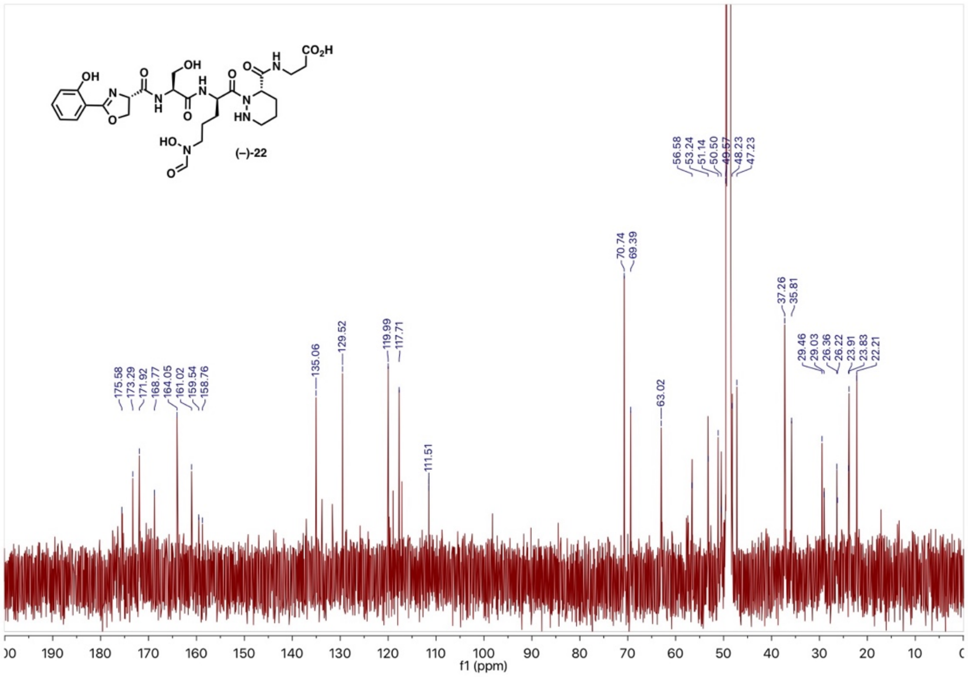

^{13}C NMR (151 MHz, CD_3OD) of (–)-22

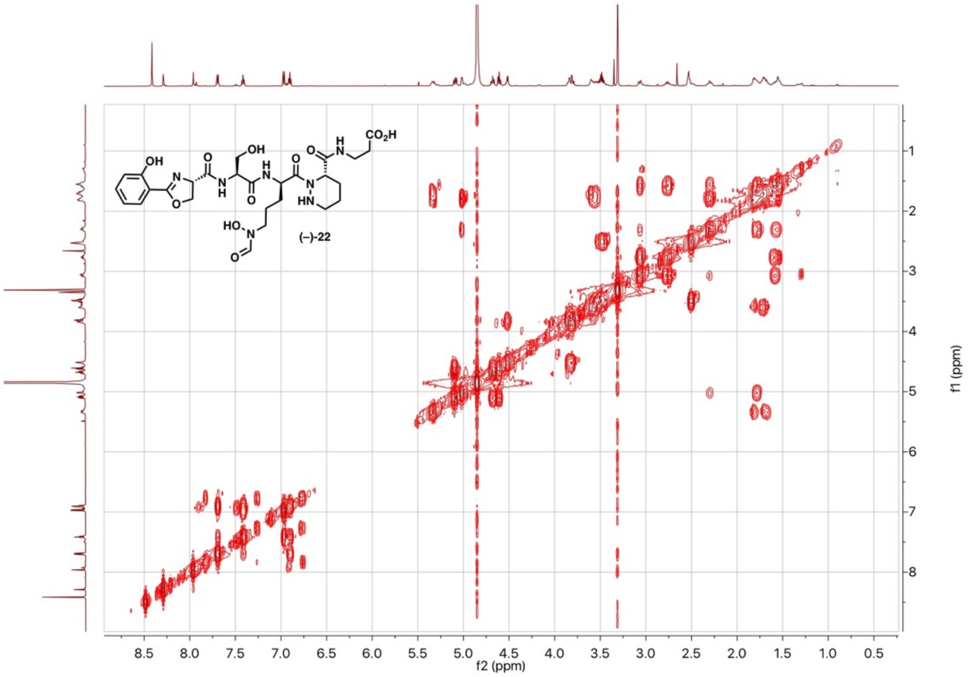

gCOSY (600 MHz, CD_3OD) of (–)-22

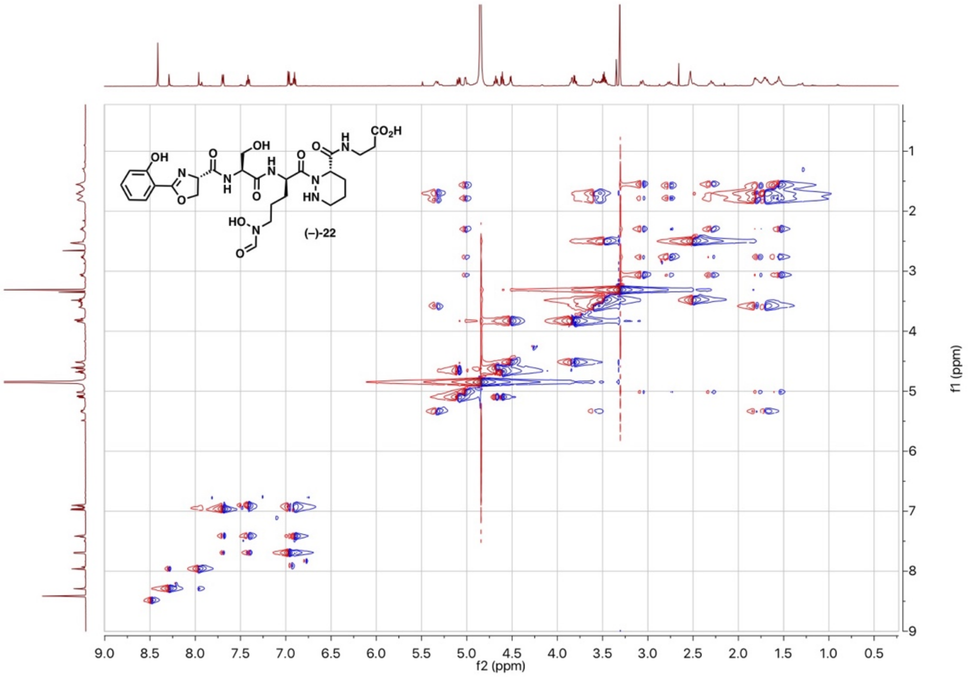

TOCSY (600 MHz, CD_3OD) of (–)-22

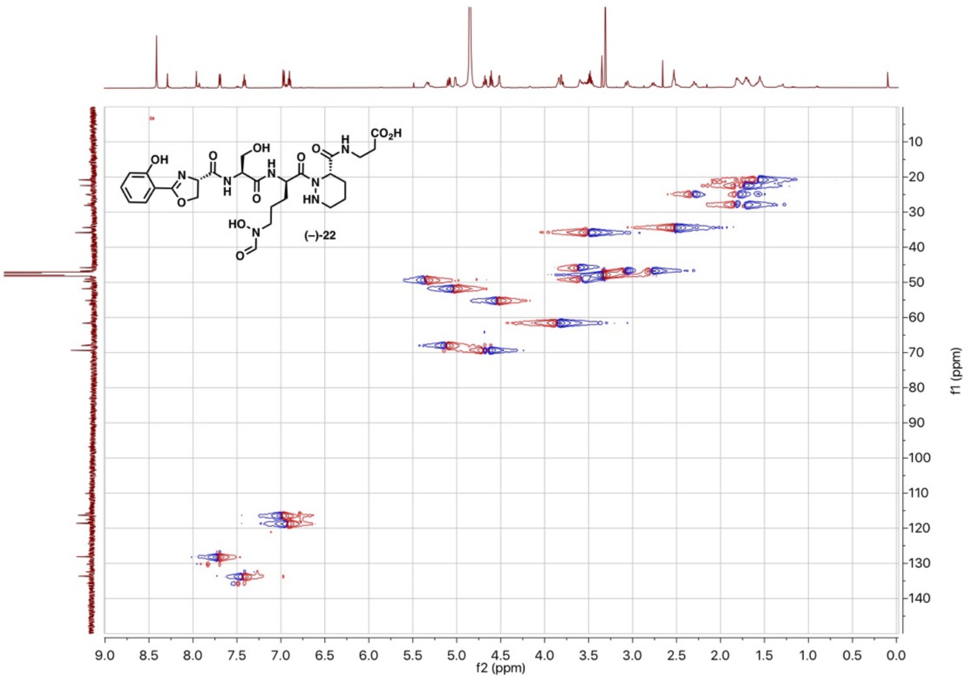

HSQC (600 MHz, CD_3OD) of (–)-22

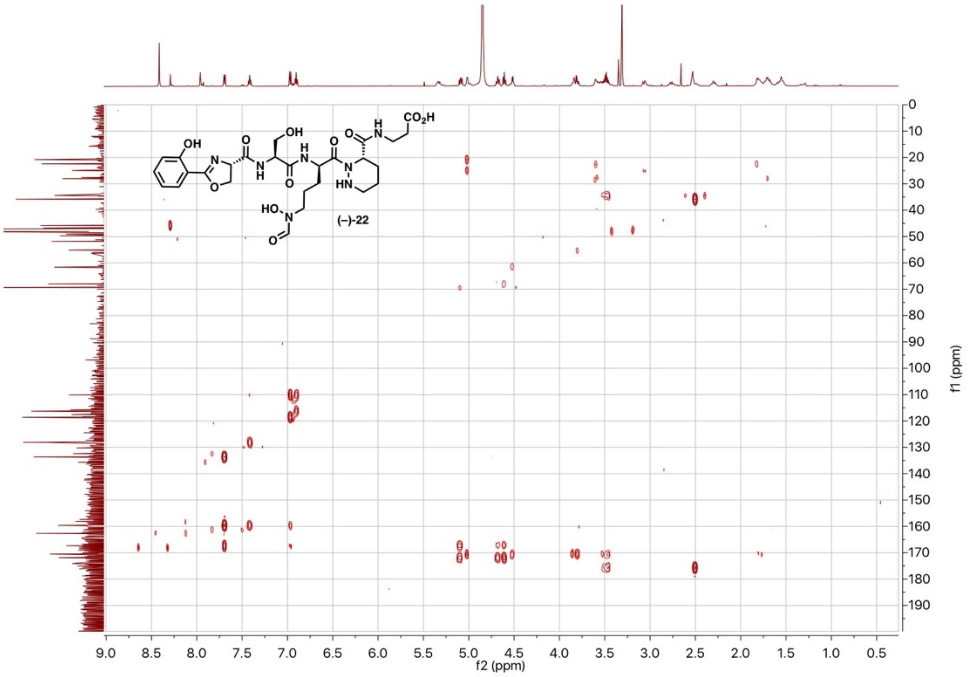

HMBC (600 MHz, CD_3OD) of (–)-22

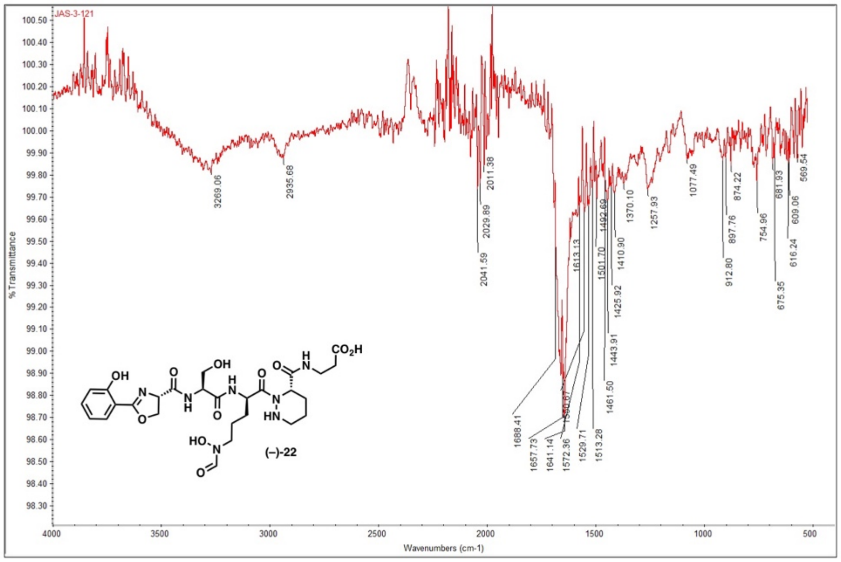

IR Spectrum of (–)-22

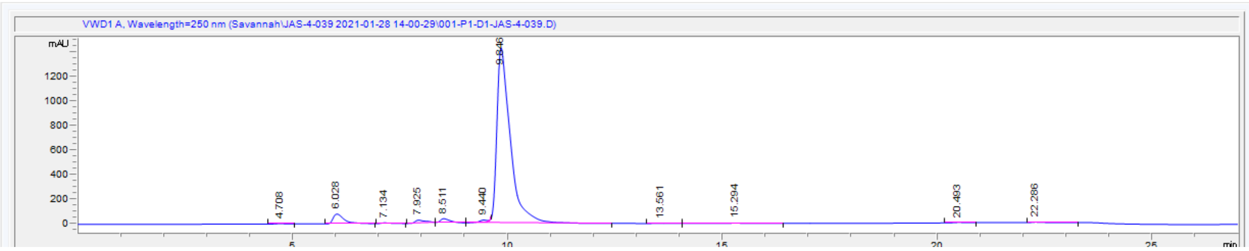

HPLC Trace of (–)-22 After Deprotection from (+)-S9

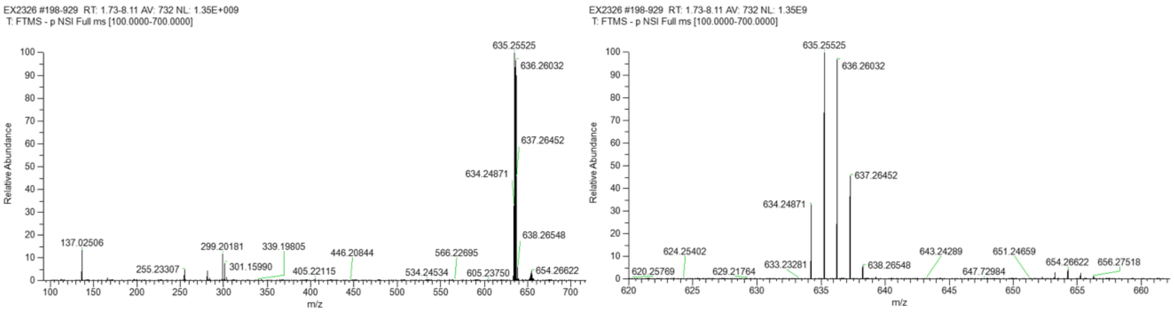

HRMS Spectrum (Negative Ion Mode) of (–)-22

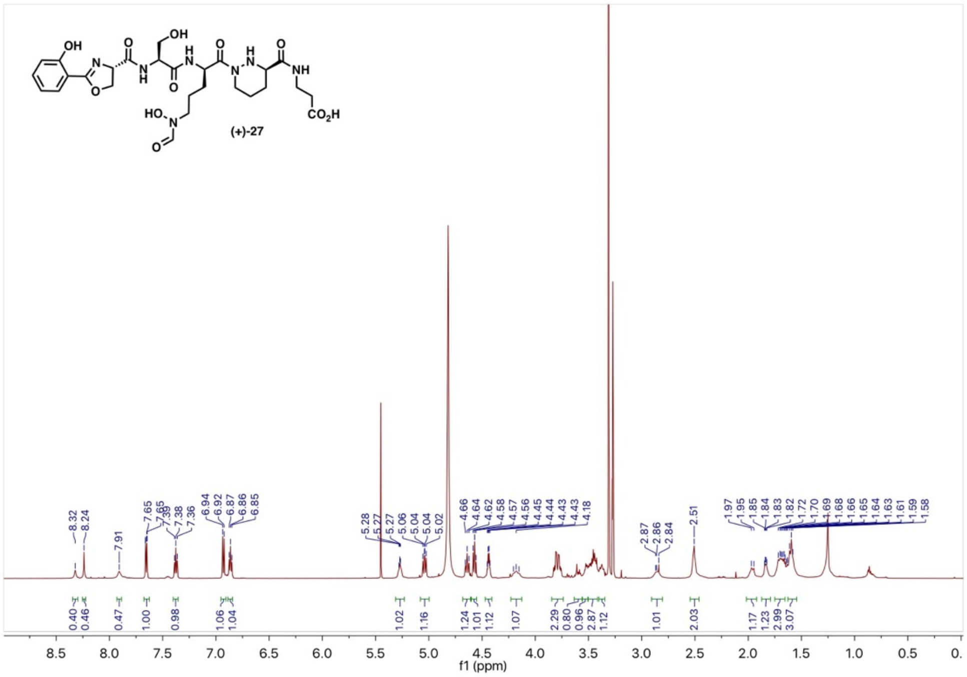

^{1}H NMR (600 MHz, CD_3OD) of (+)-27

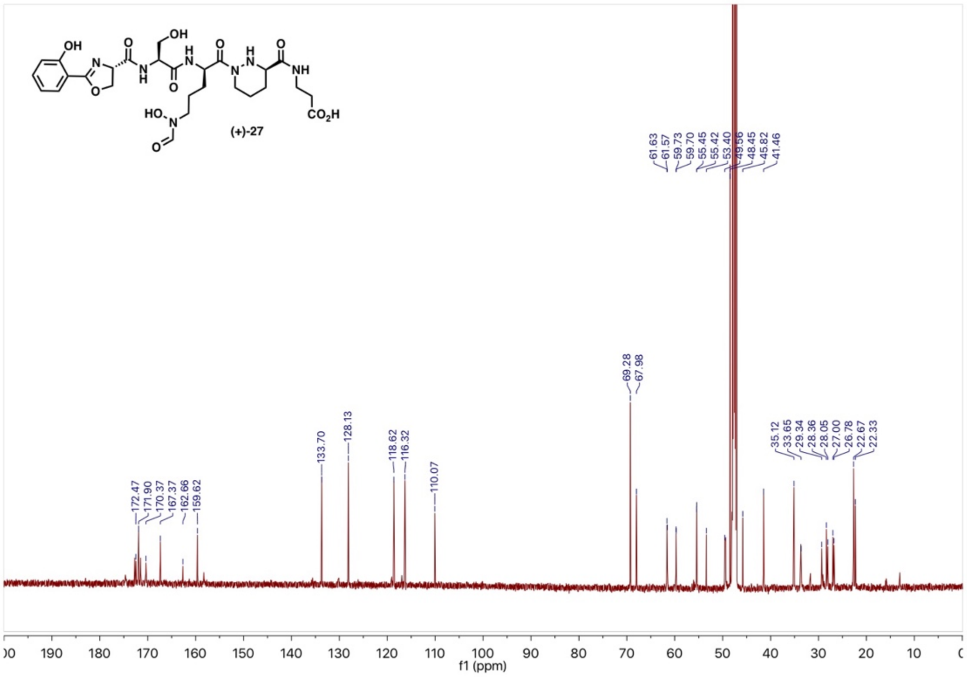

^{13}C NMR (151 MHz, CD_3OD) of (+)-27

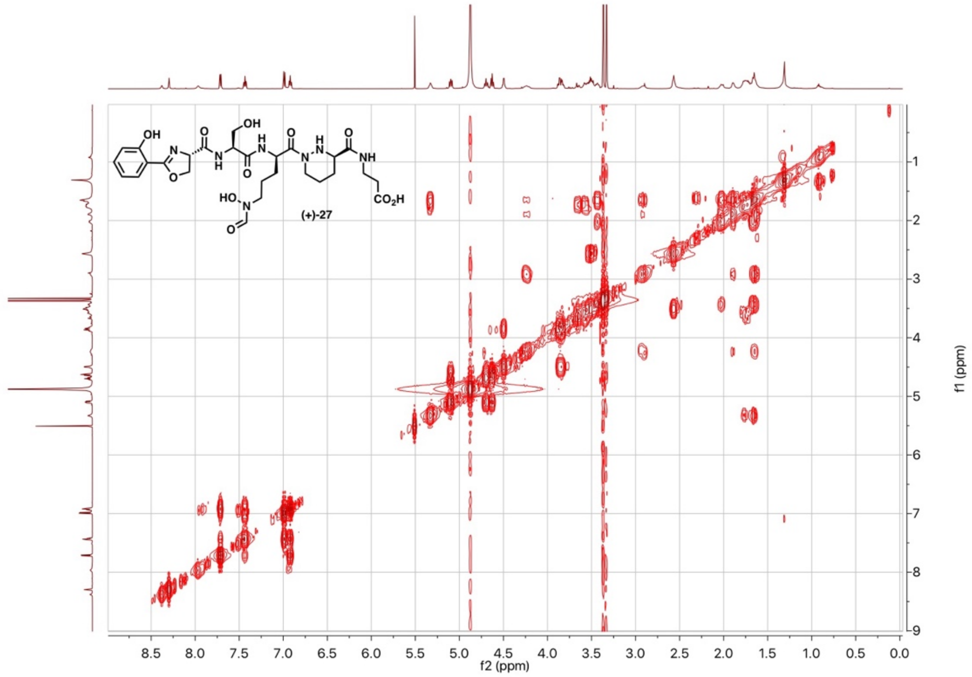

gCOSY (600 MHz, CD_3OD) of (+)-27

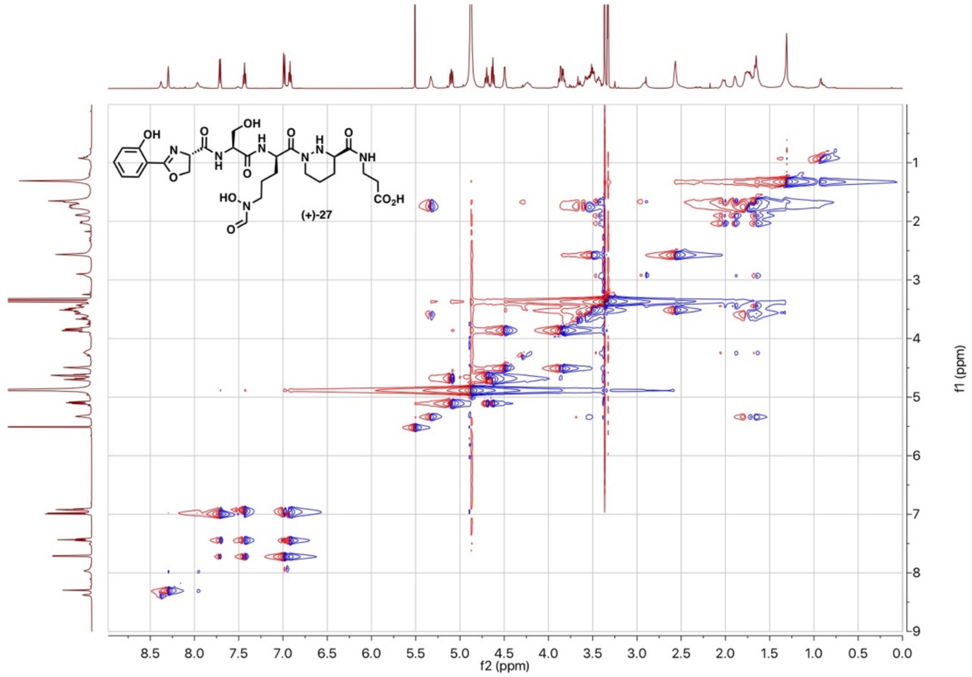

TOCSY (600 MHz, CD_3OD) of (+)-27

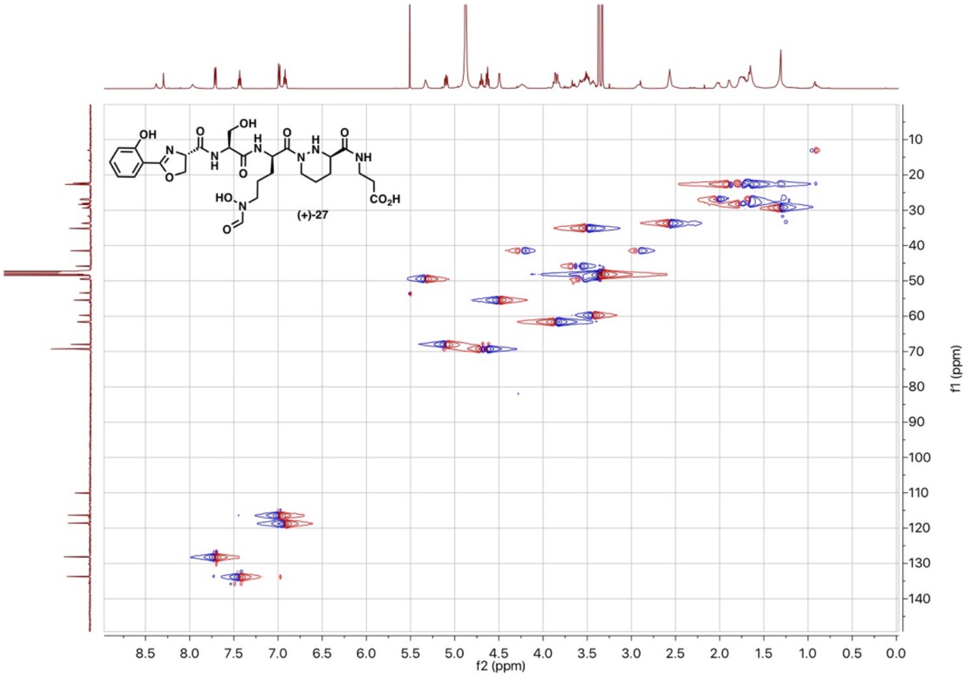

HSQC (600 MHz, CD_3OD) of (+)-27

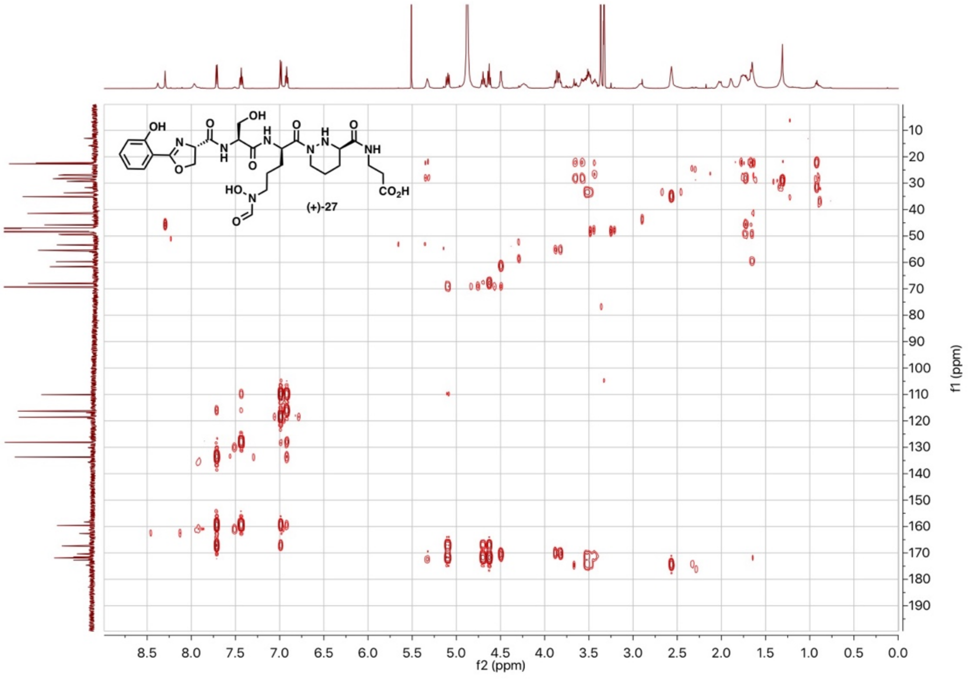

HMBC (600 MHz, CD_3OD) of (+)-27

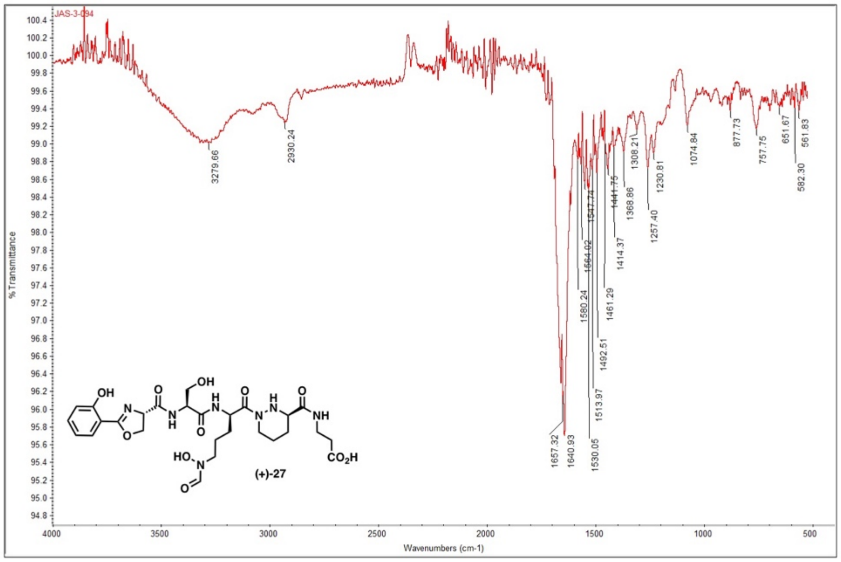

IR Spectrum of (+)-27

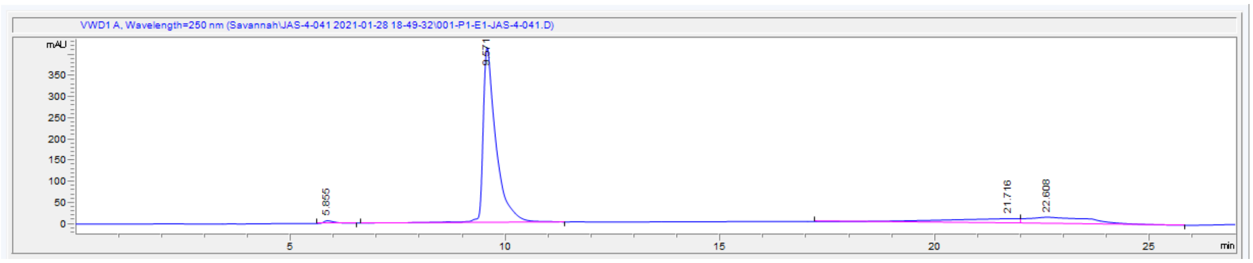

HPLC Trace of (+)-27 After Deprotection from (–)-S13

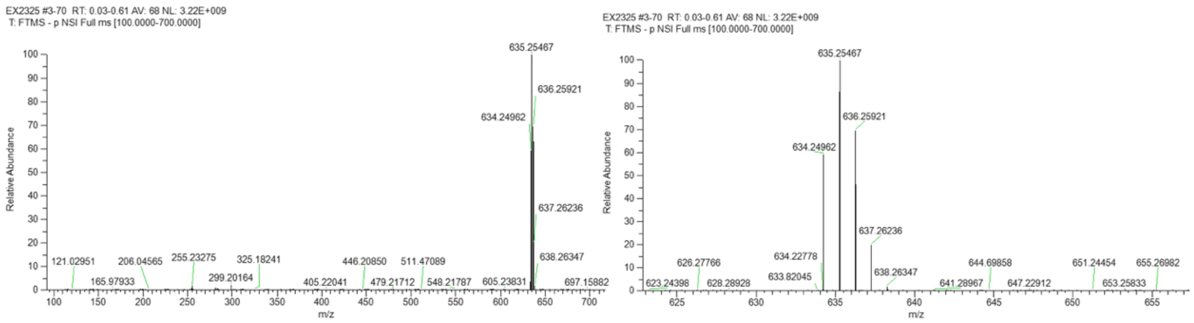

HRMS Spectrum (Negative Ion Mode) of (+)-27

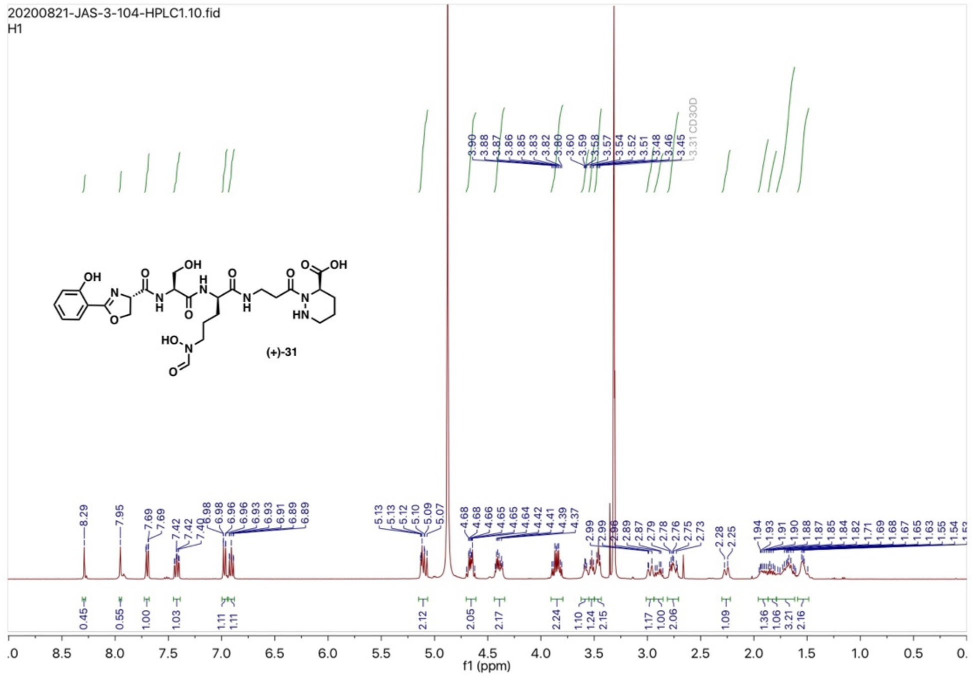

^{1}H NMR (600 MHz, CD$_3$OD) of (+)-31

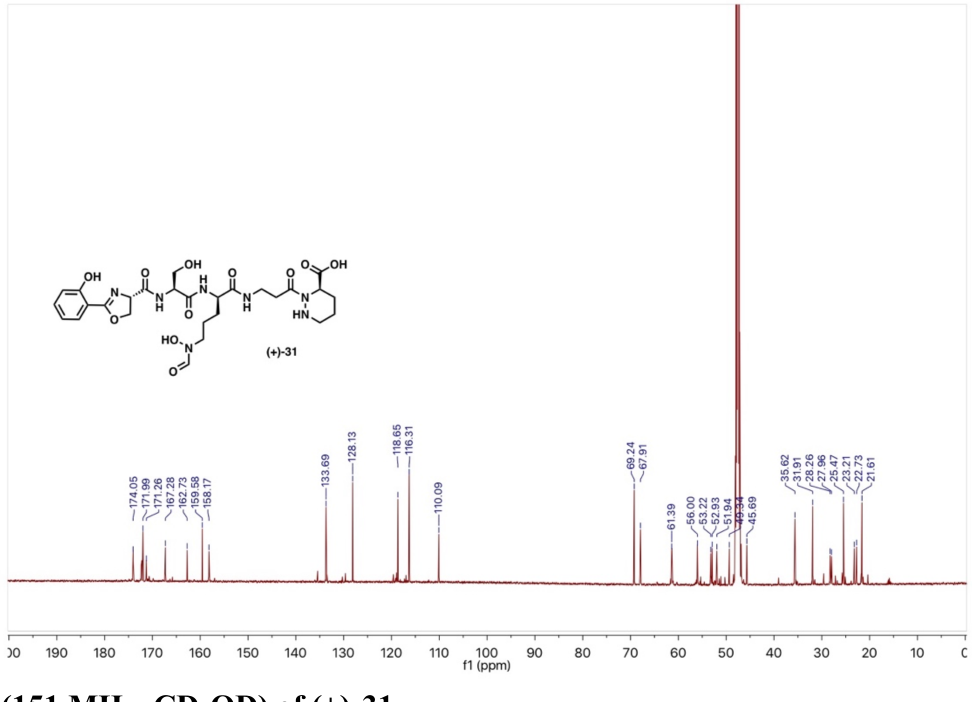

^{13}C NMR (151 MHz, CD$_3$OD) of (+)-31

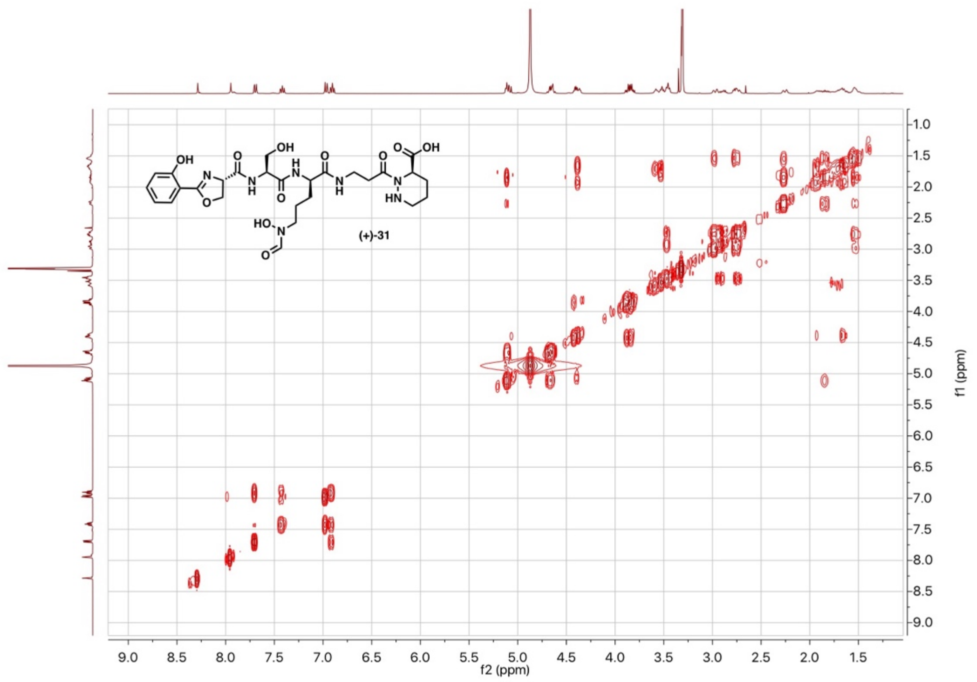

gCOSY (600 MHz, CD_3OD) of (+)-31

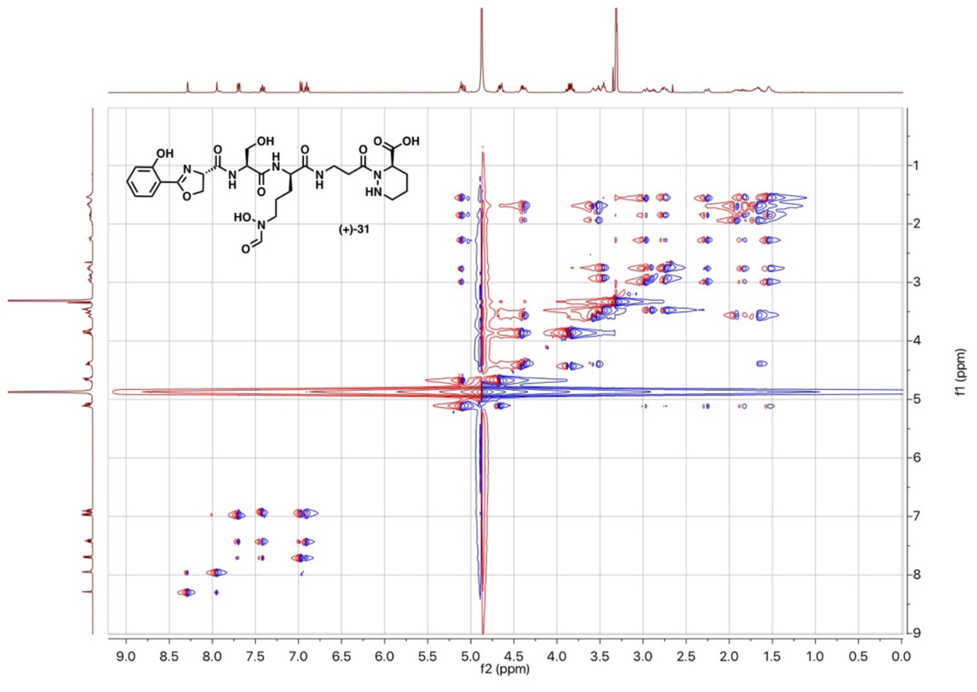

TOCSY (600 MHz, CD_3OD) of (+)-31

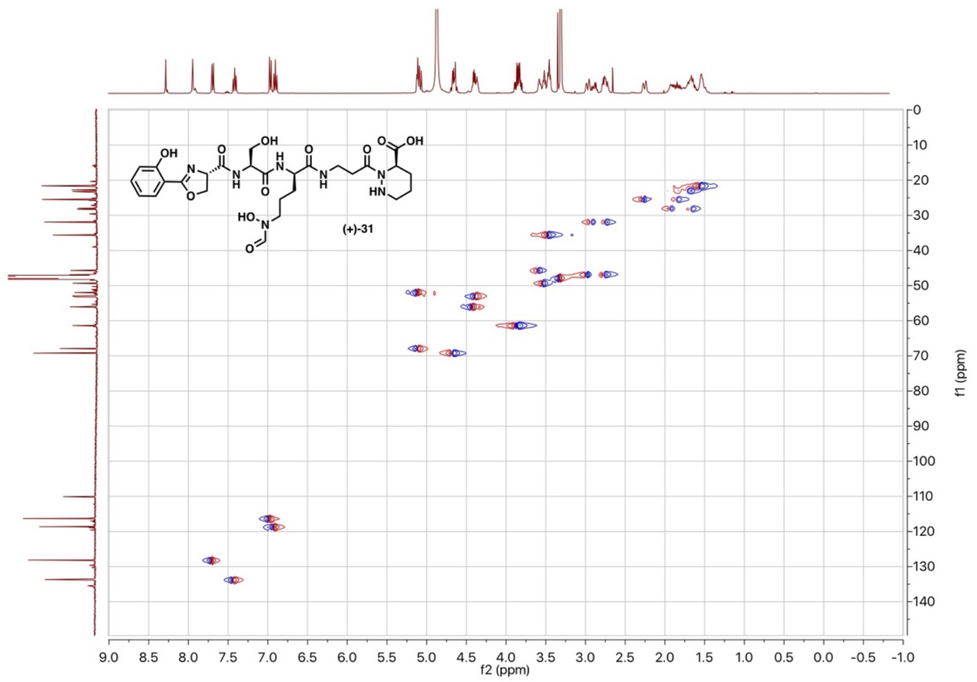

HSQC (600 MHz, CD_3OD) of (+)-31

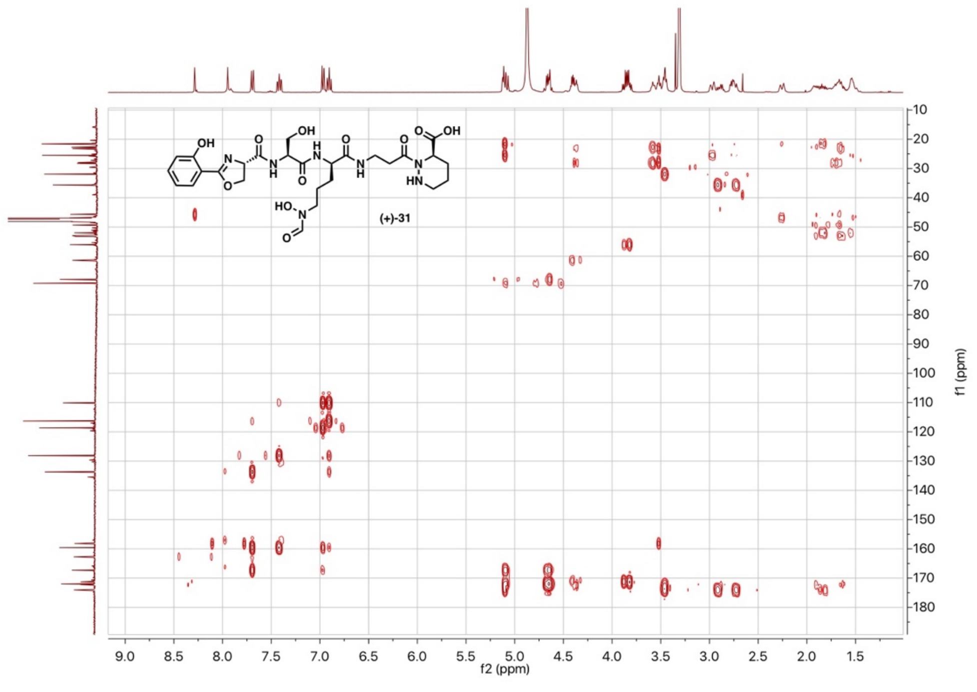

HMBC (600 MHz, CD_3OD) of (+)-31

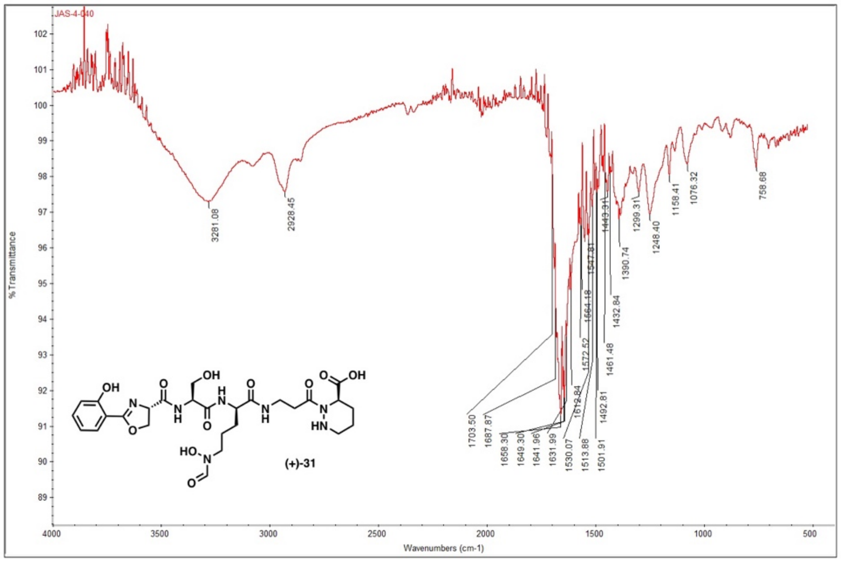

IR Spectrum of (+)-31

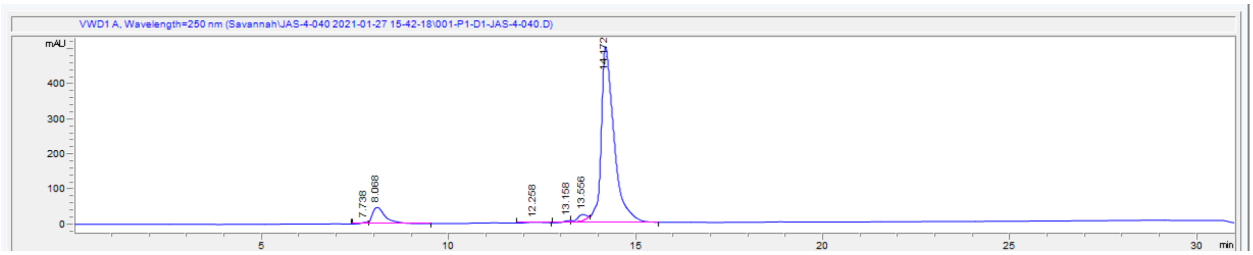

HPLC Trace of (+)-31 After Deprotection from (+)-S14

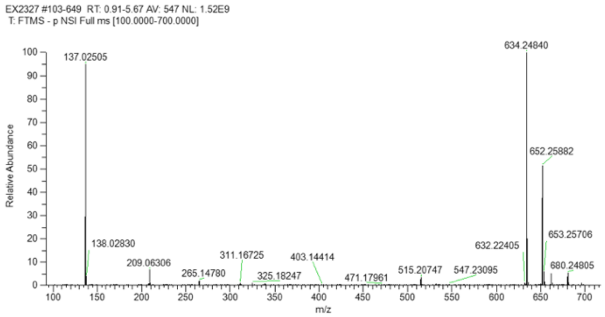

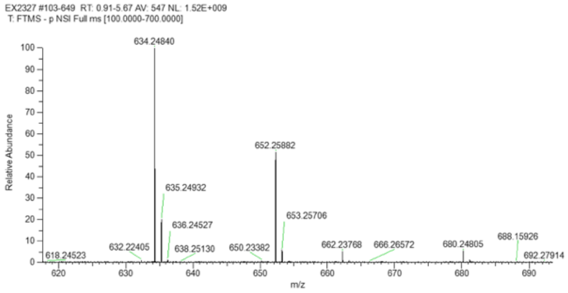

HRMS Spectrum (Negative Ion Mode) of (+)-31

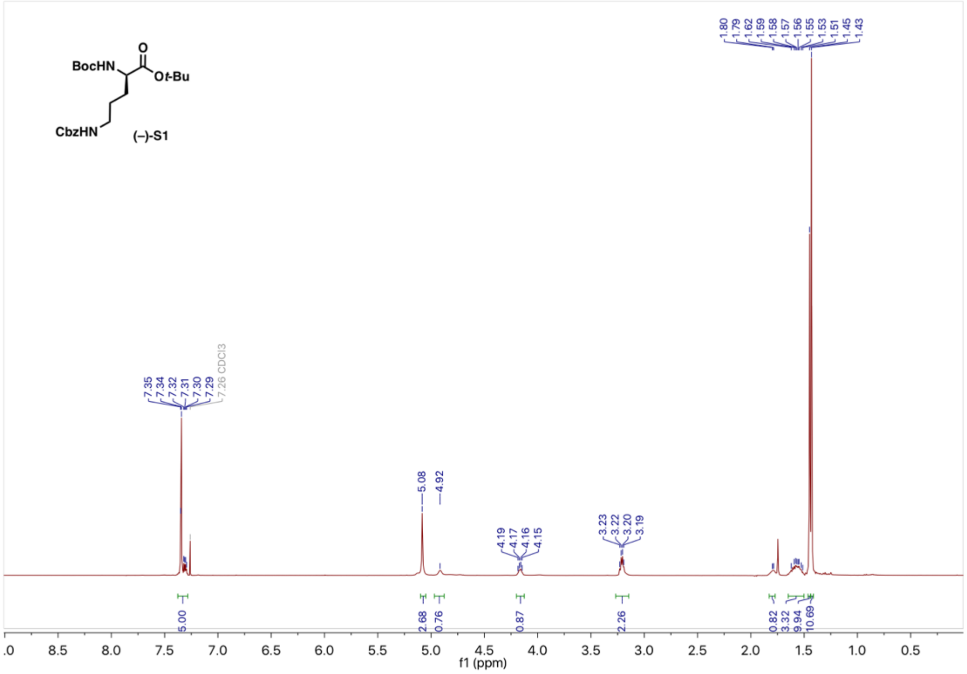

^{1}H NMR (500 MHz, CDCl₃) of (–)-S1

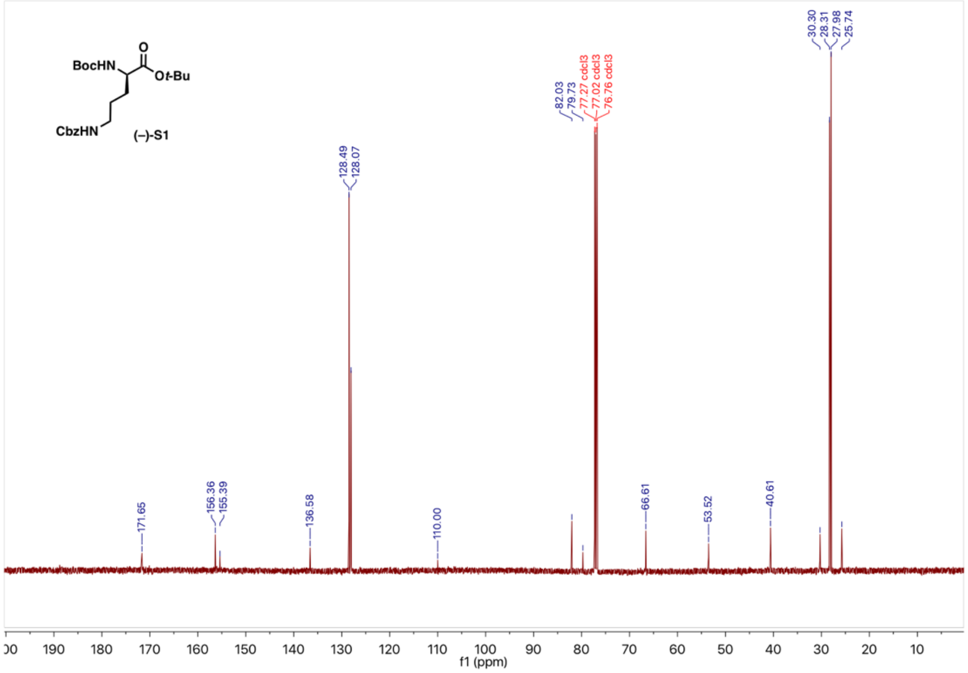

^{13}C NMR (126 MHz, CDCl₃) (–)-S1

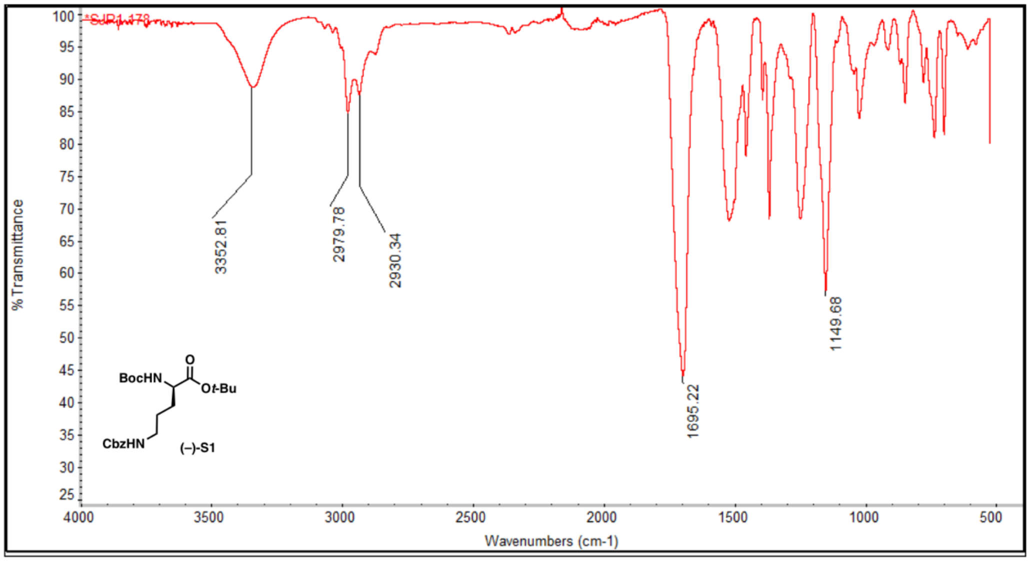

IR (neat) of (–)-S1

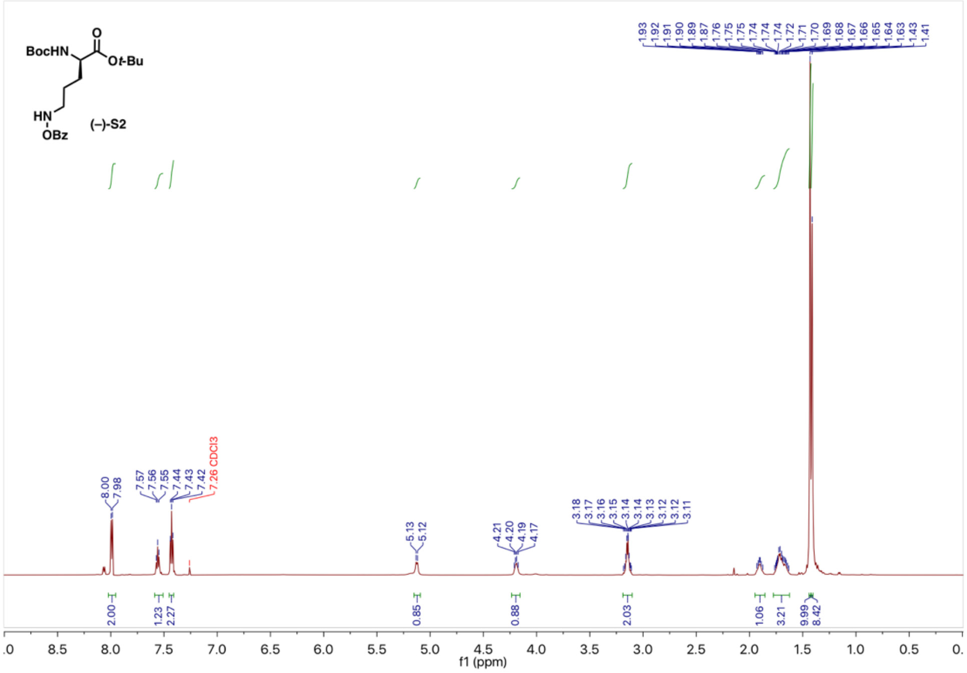

^{1}H NMR (600 MHz, $CDCl_3$) of (–)-S2

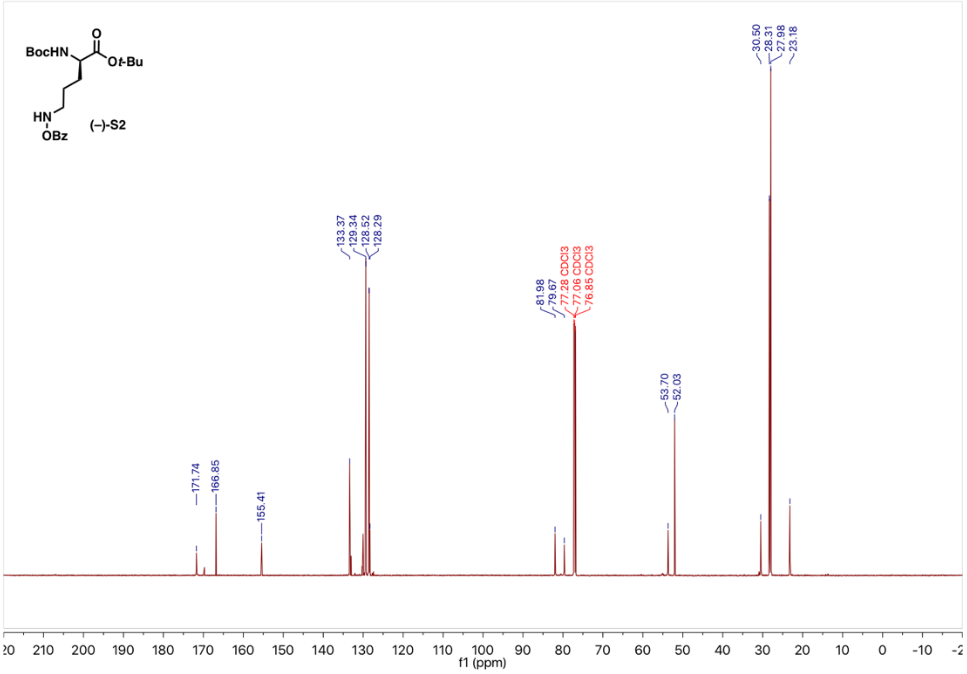

^{13}C NMR (151 MHz, $CDCl_3$) of (–)-S2

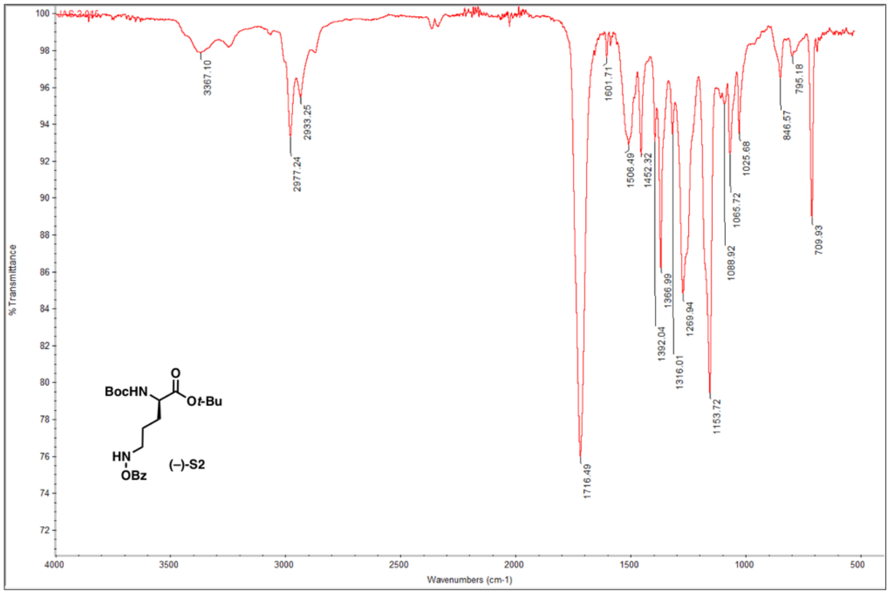

IR (neat) of (–)-S2

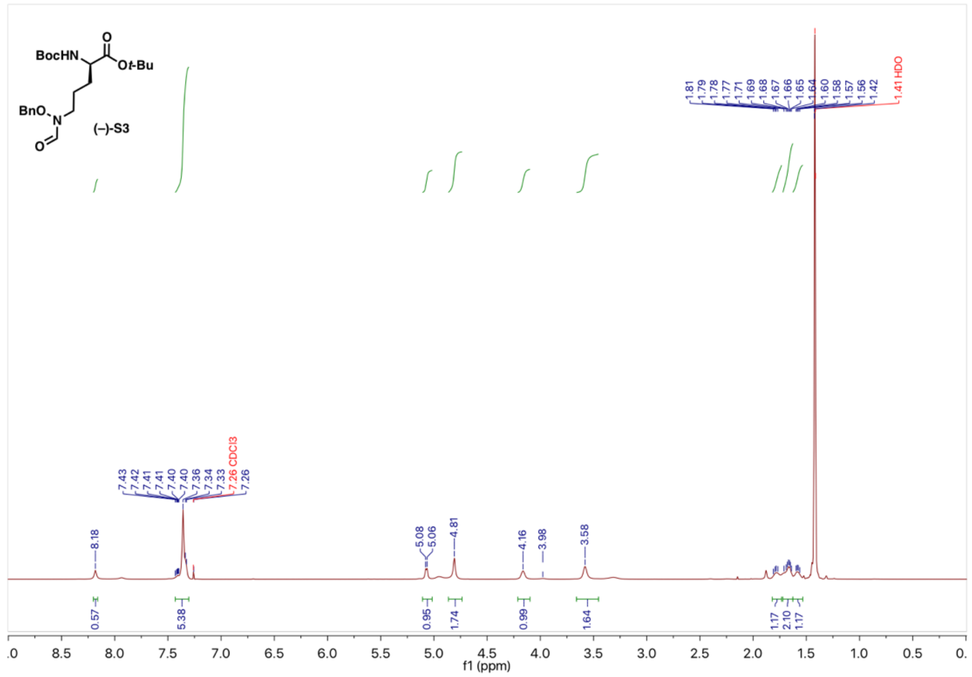

^{1}H NMR (600 MHz, CDCl3) of (–)-S3

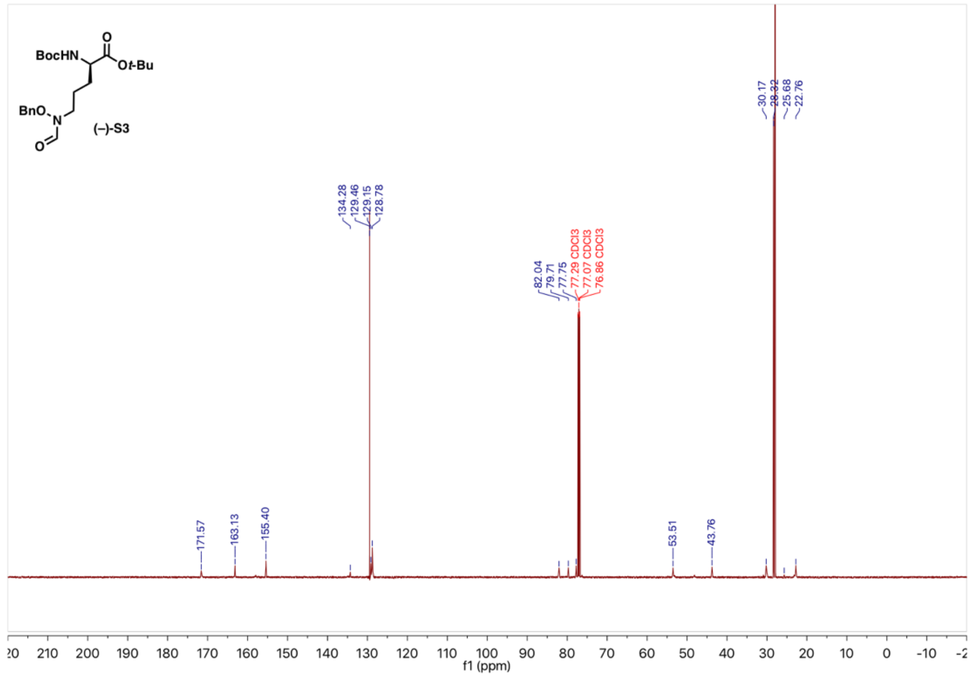

^{13}C NMR (151 MHz, CDCl3) of (–)-S3

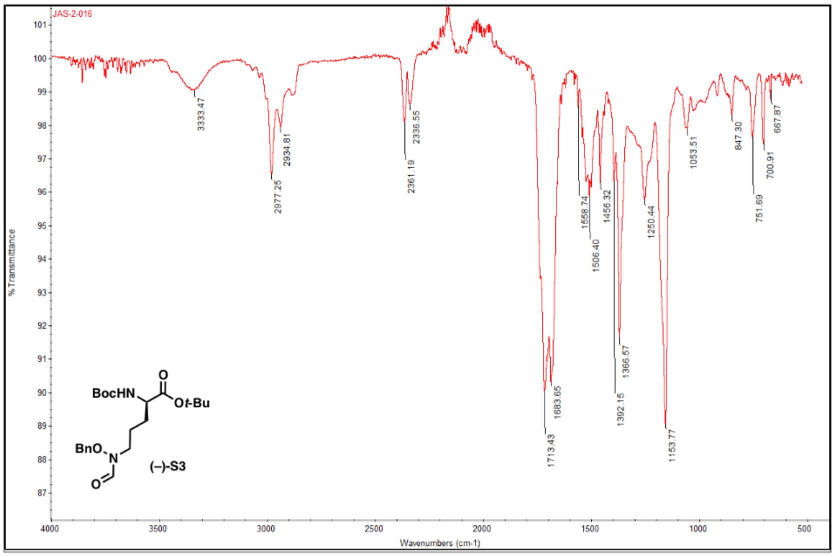

IR (neat) of (–)-S3

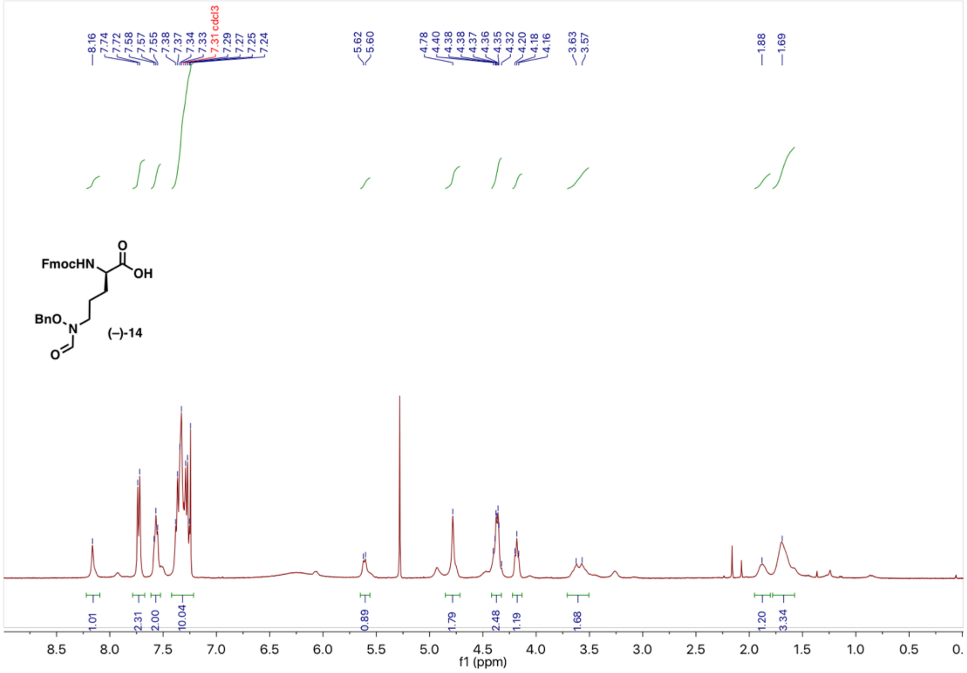

^{1}H NMR (400 MHz, $CDCl_3$) of (–)-14

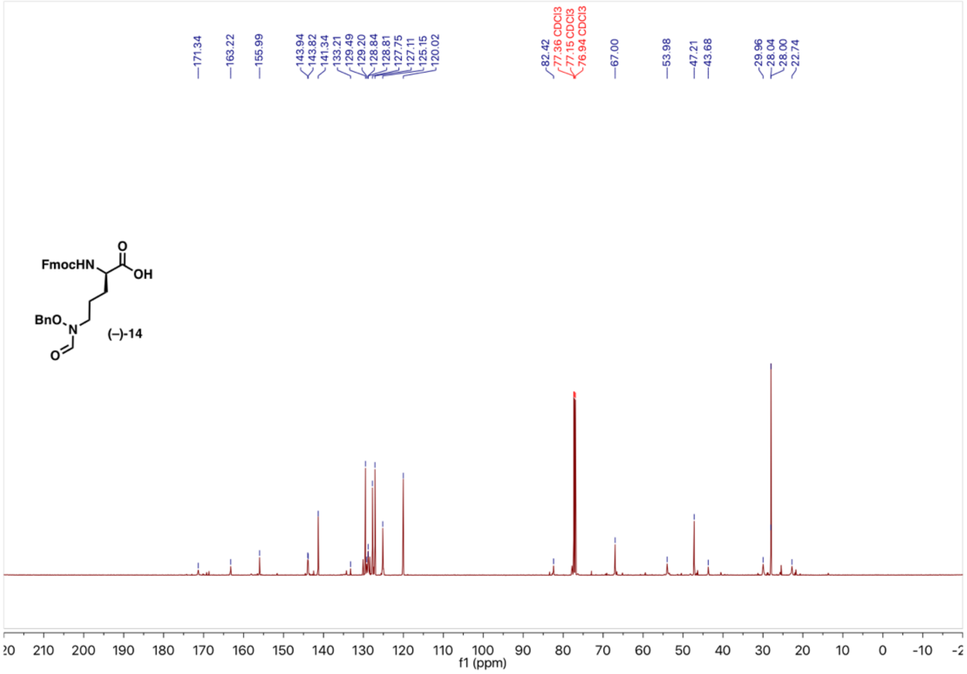

^{13}C NMR (151 MHz, $CDCl_3$) of (–)-14

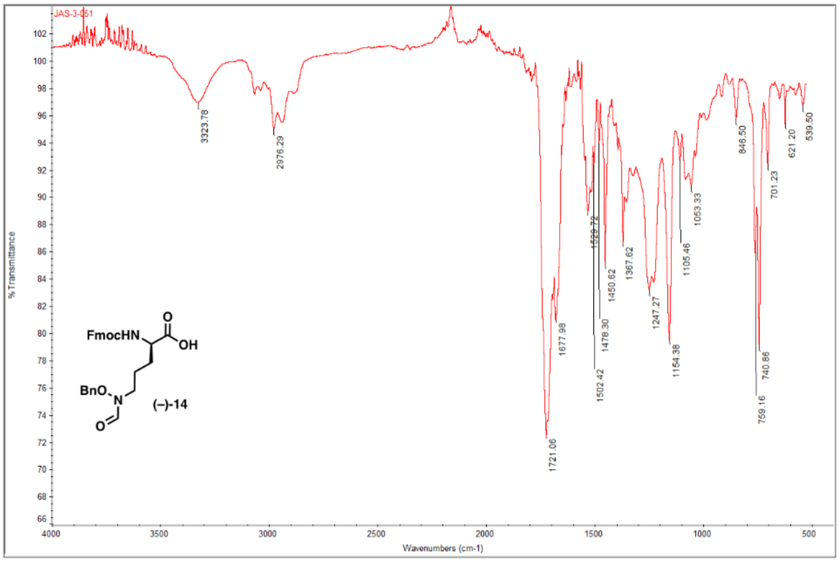

IR (neat) of (–)-14

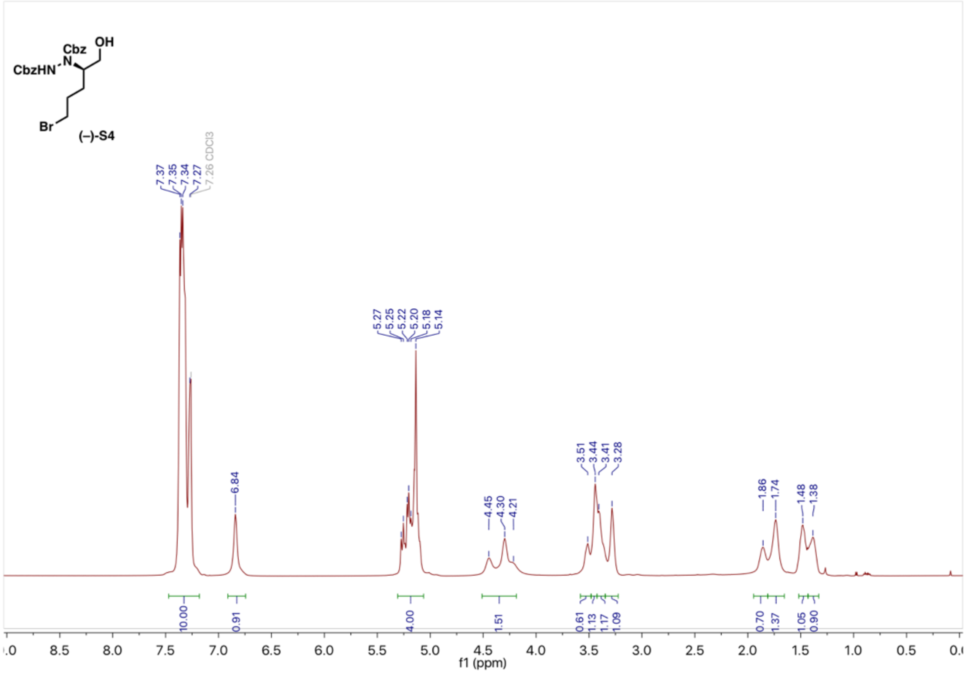

^{1}H-NMR (600 MHz, $CDCl_3$) of (–)-S4

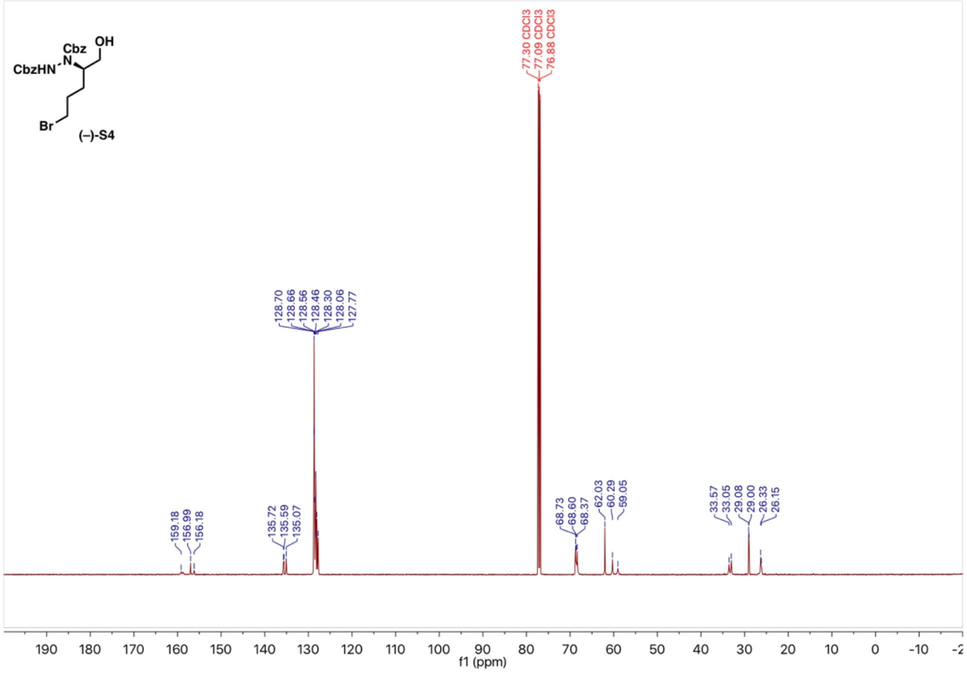

^{13}C NMR (151 MHz, $CDCl_3$) of (–)-S4

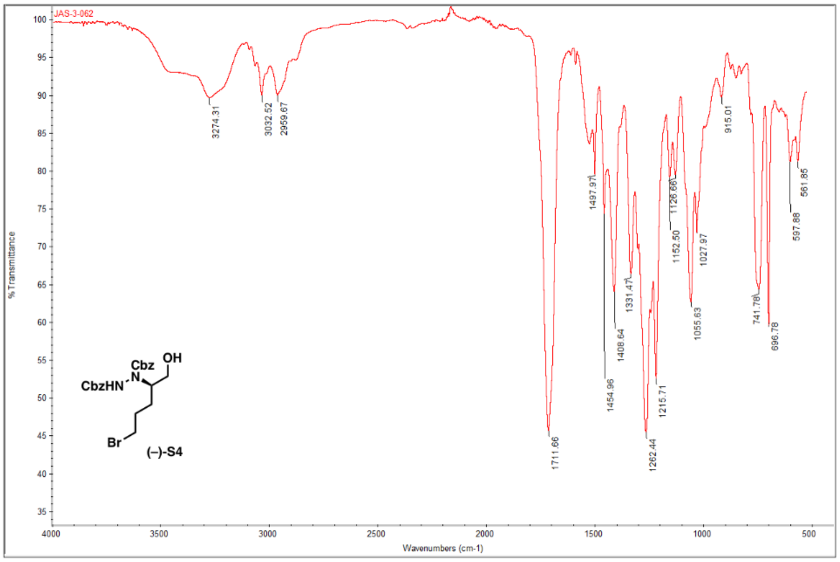

IR (neat) of (–)-S4

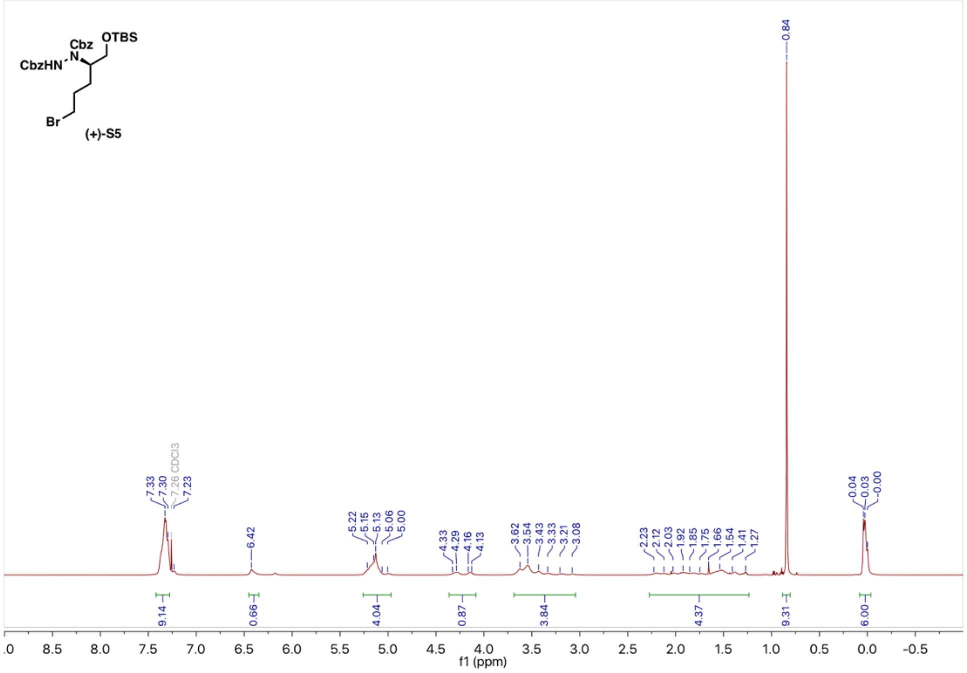

^{1}H NMR (600 MHz, $CDCl_3$) of (+)-S5

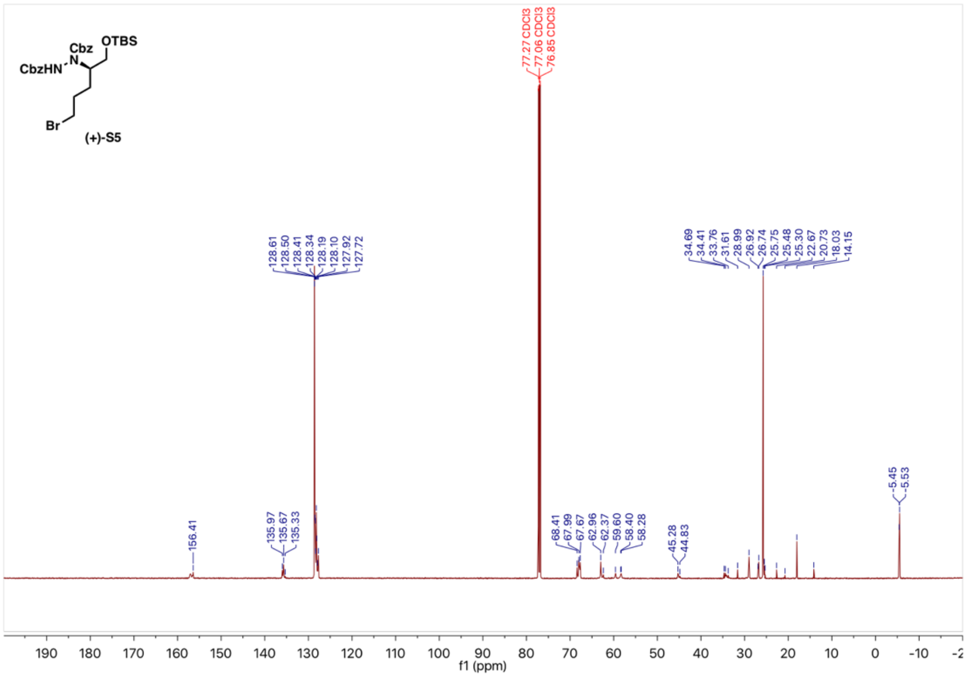

^{13}C NMR (151 MHz, $CDCl_3$) of (+)-S5

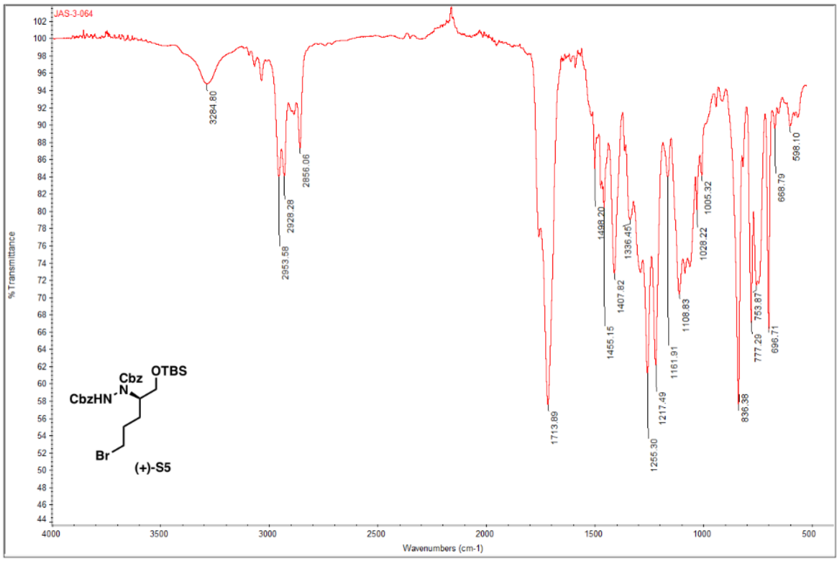

IR (neat) of (+)-S5

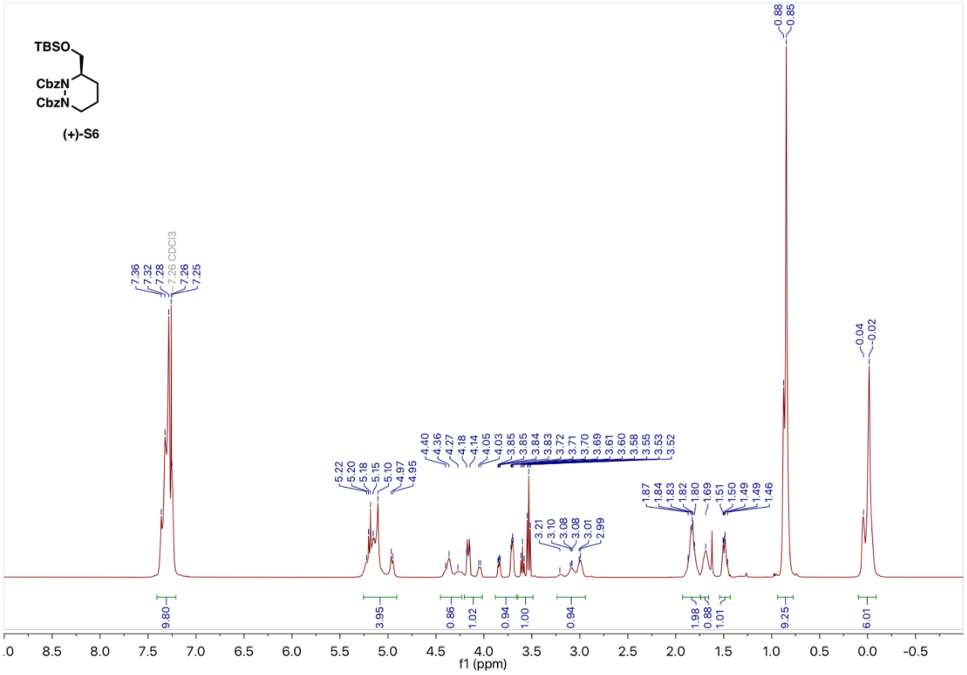

^{1}H NMR (600 MHz, $CDCl_3$) of (+)-S6

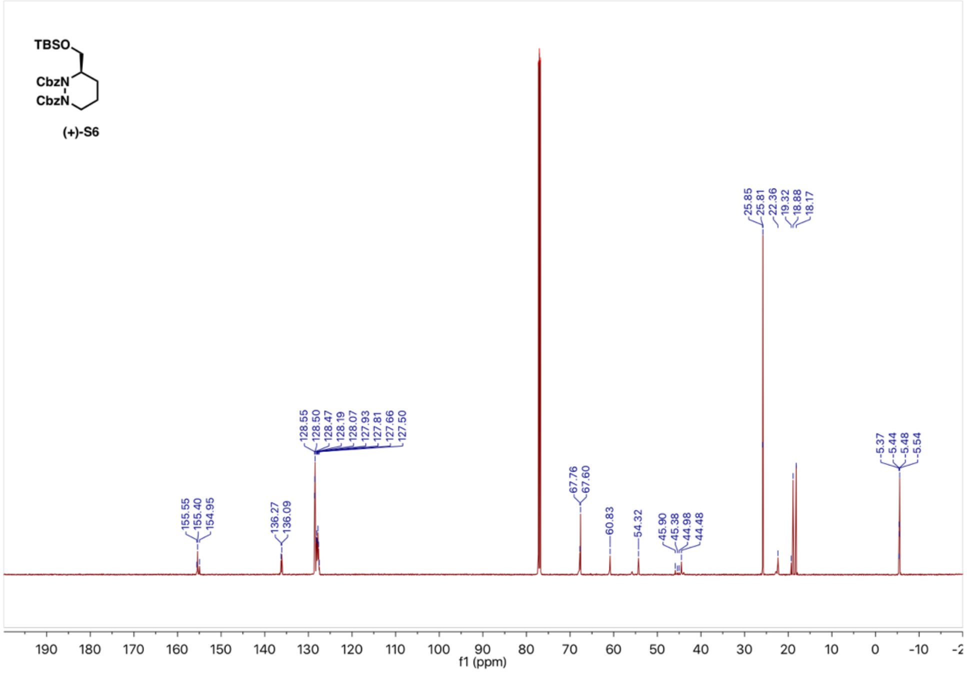

^{13}C NMR (151 MHz, $CDCl_3$) of (+)-S6

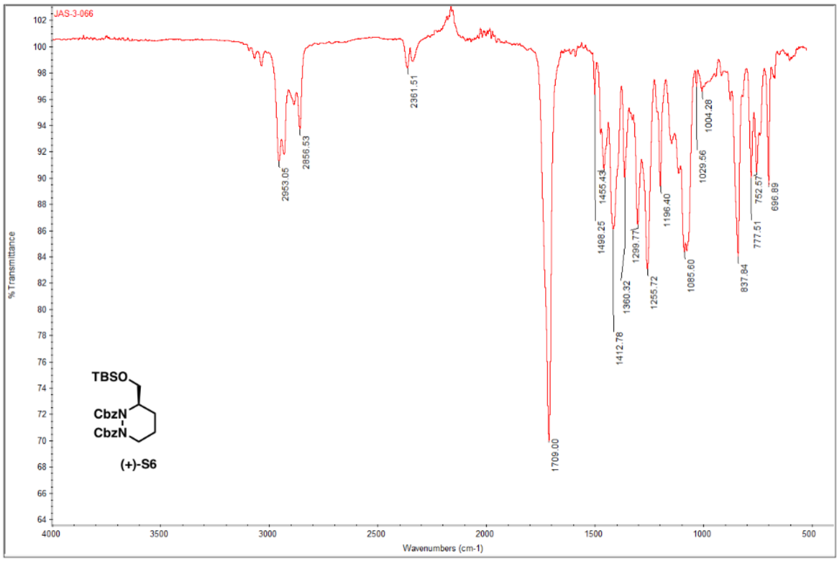

IR (neat) of (+)-S6

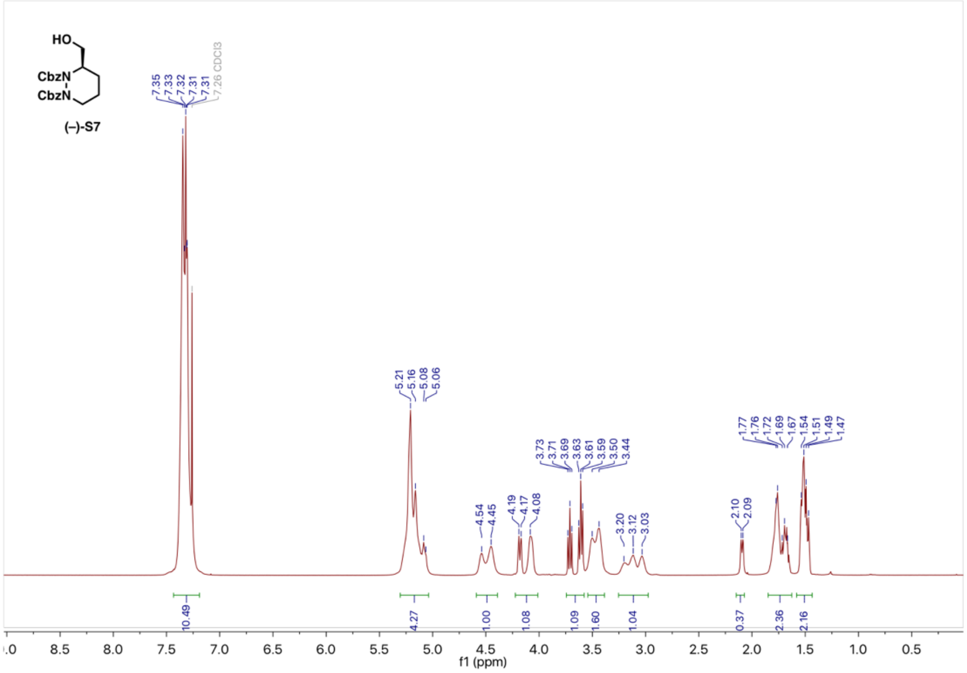

^{1}H NMR (600 MHz, CDCl$_3$) of (–)-S7

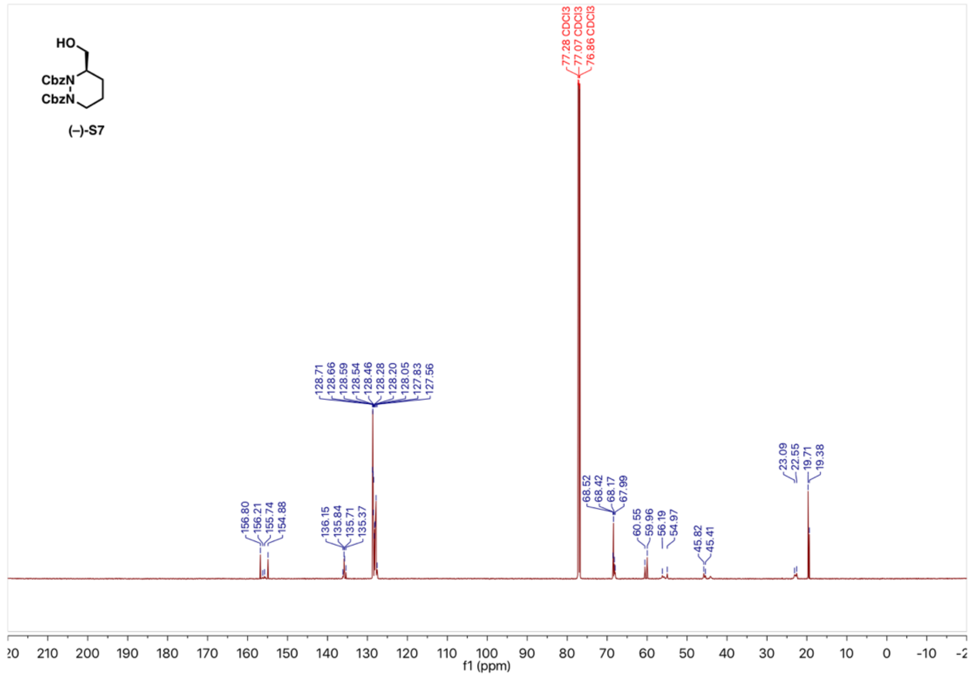

^{13}C NMR (151 MHz, $CDCl_3$) of (–)-S7

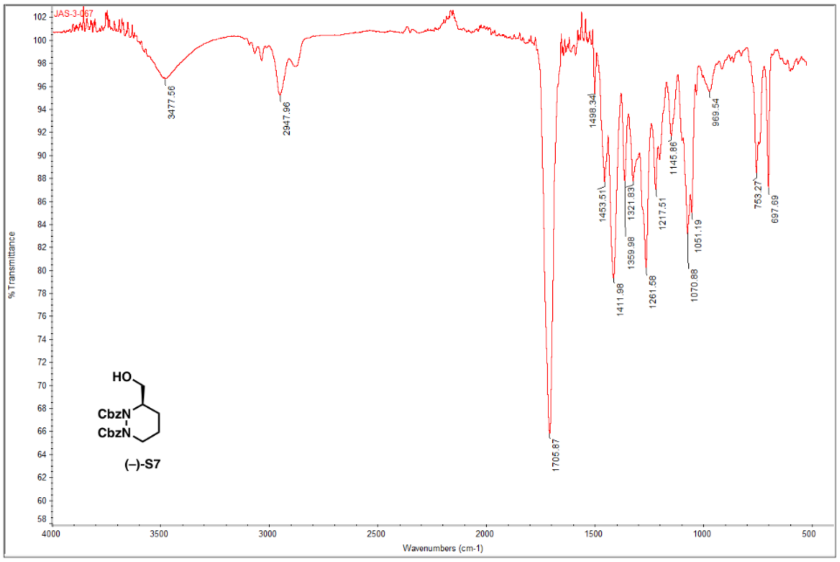

IR (neat) of (–)-S7

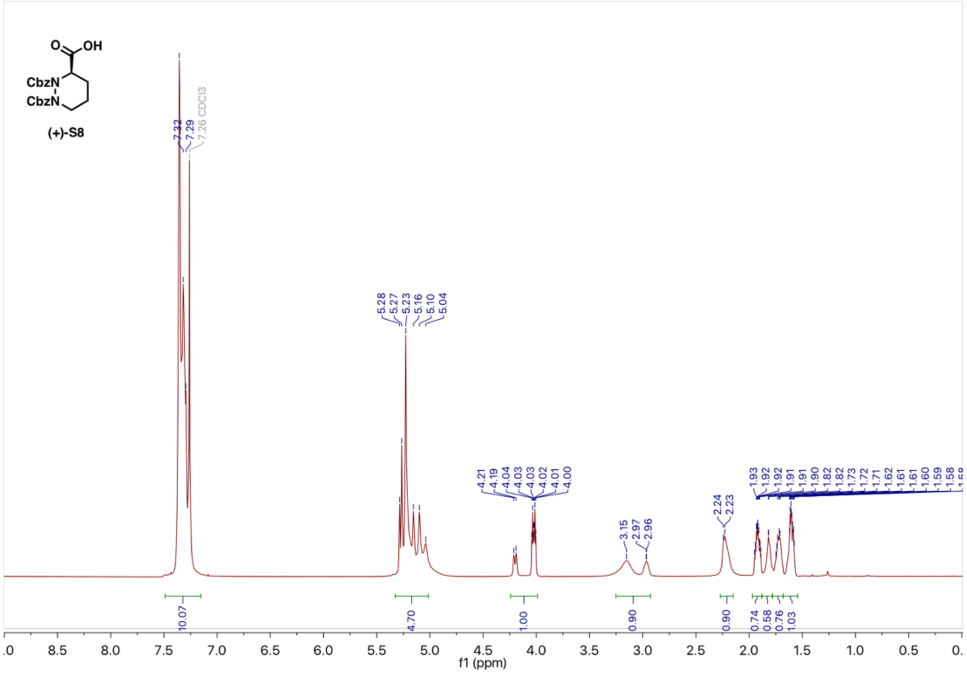

^{1}H NMR (600 MHz, $CDCl_3$) of (+)-S8

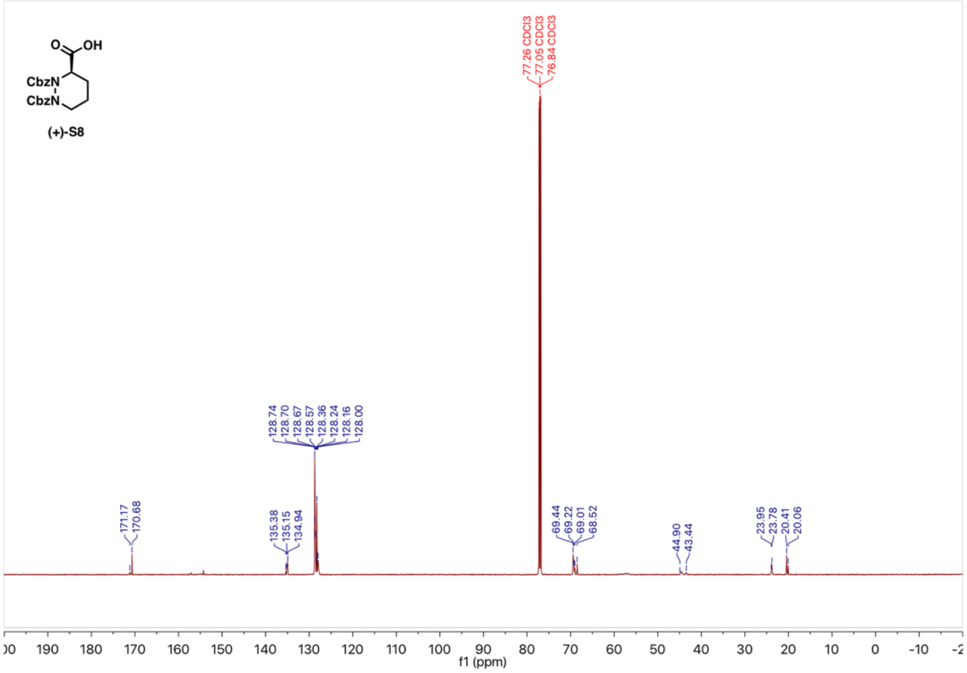

^{13}C NMR (151 MHz, $CDCl_3$) of (+)-S8

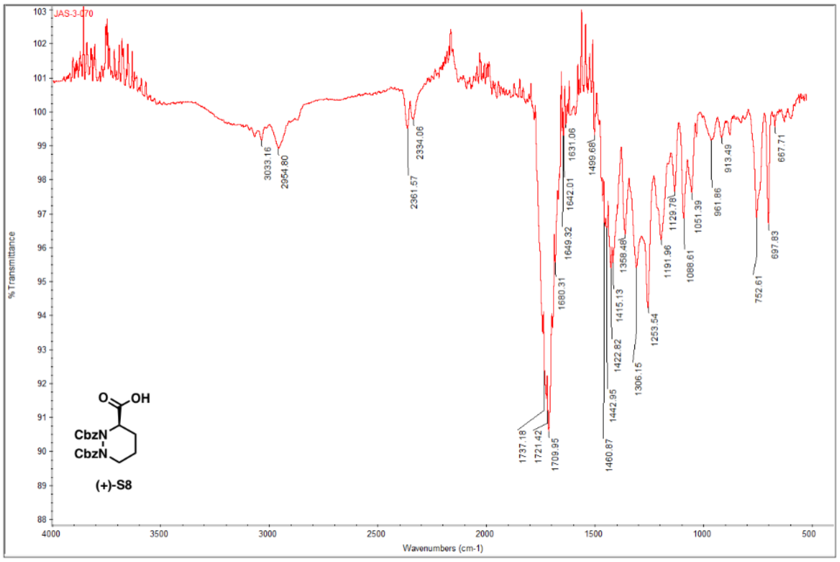

IR (neat) of (+)-S8

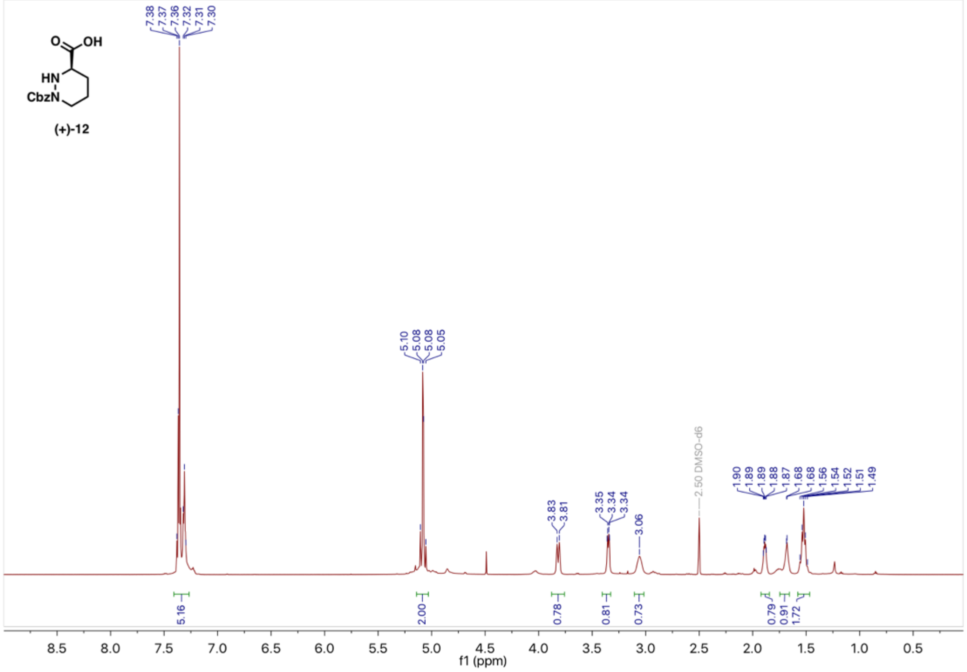

^{1}H NMR (600 MHz, DMSO-d_6) of (+)-12

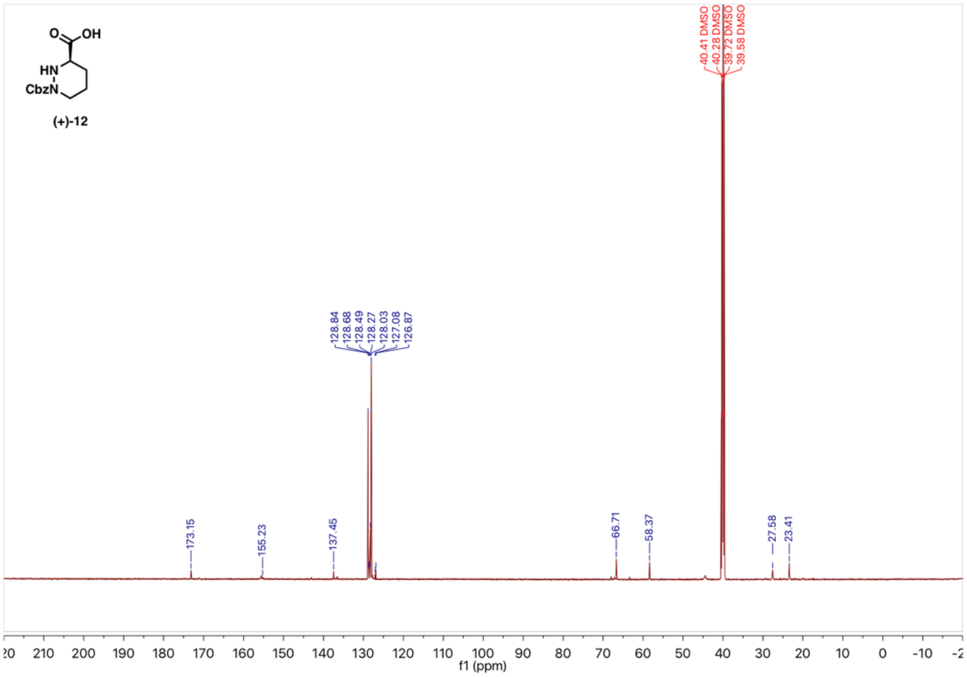

^{13}C NMR (151 MHz, DMSO-d_6) of (+)-12

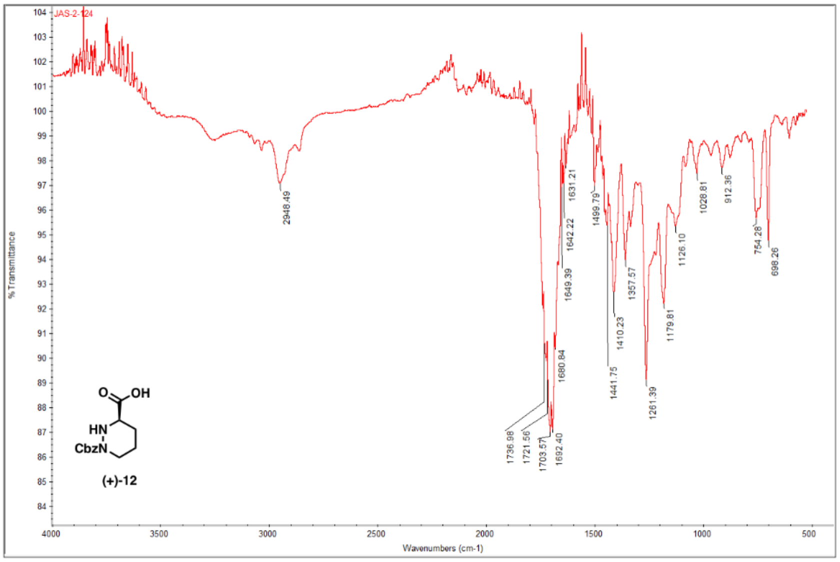

IR (neat) of (+)-12

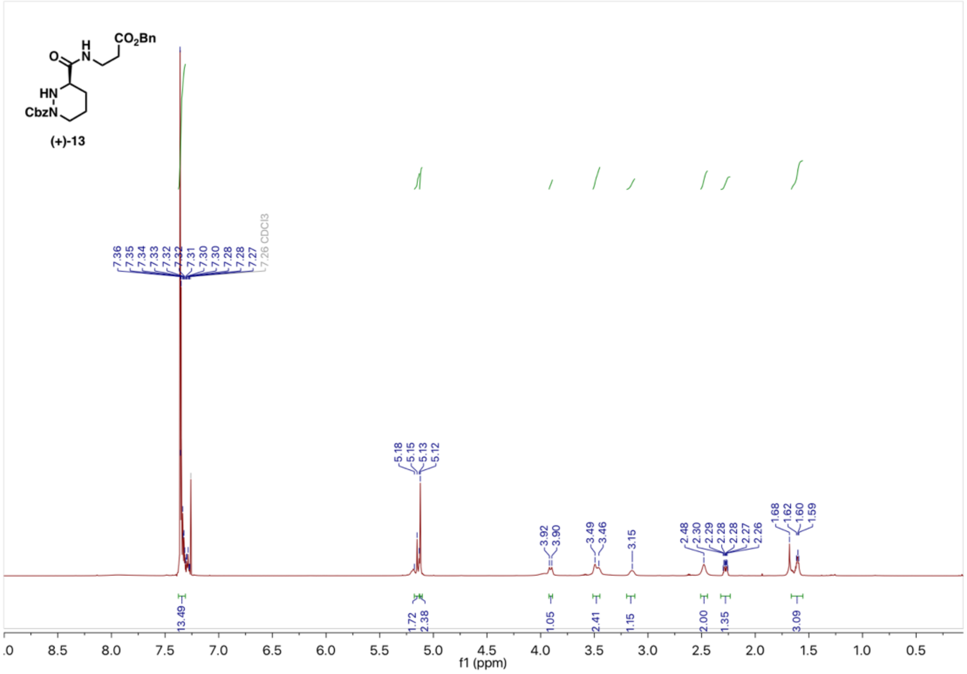

^{1}H NMR (600 MHz, $CDCl_3$) of (+)-13

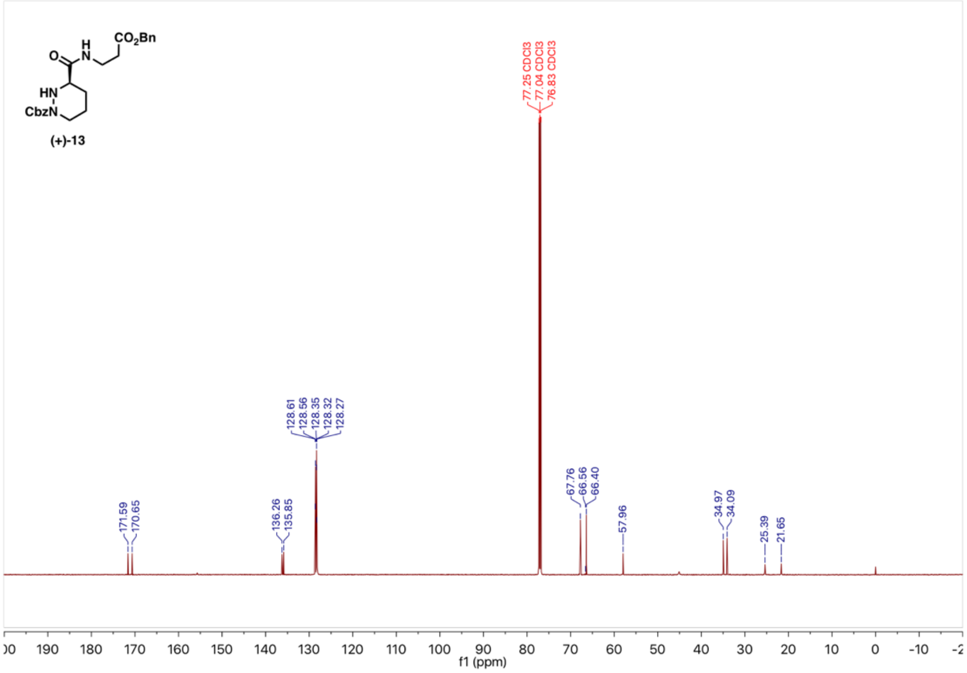

^{13}C NMR (151 MHz, CDCl3) of (+)-13

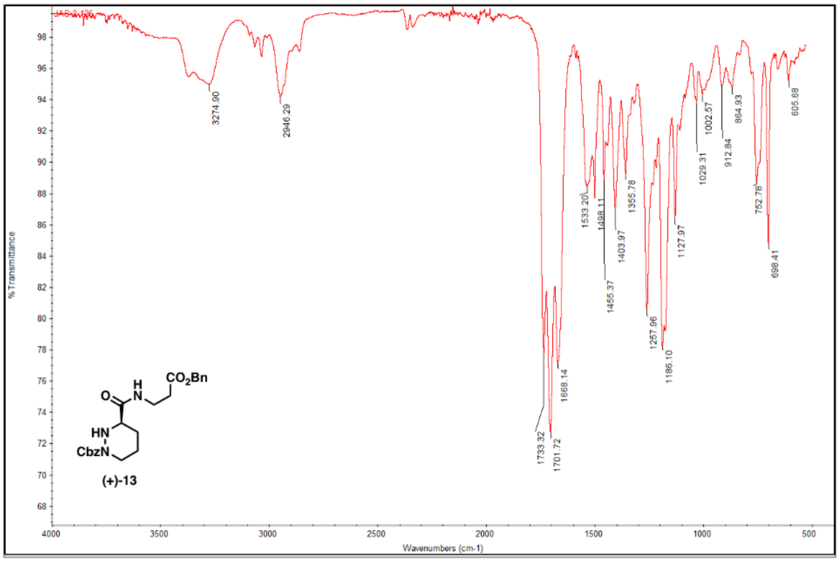

IR (neat) of (+)-13

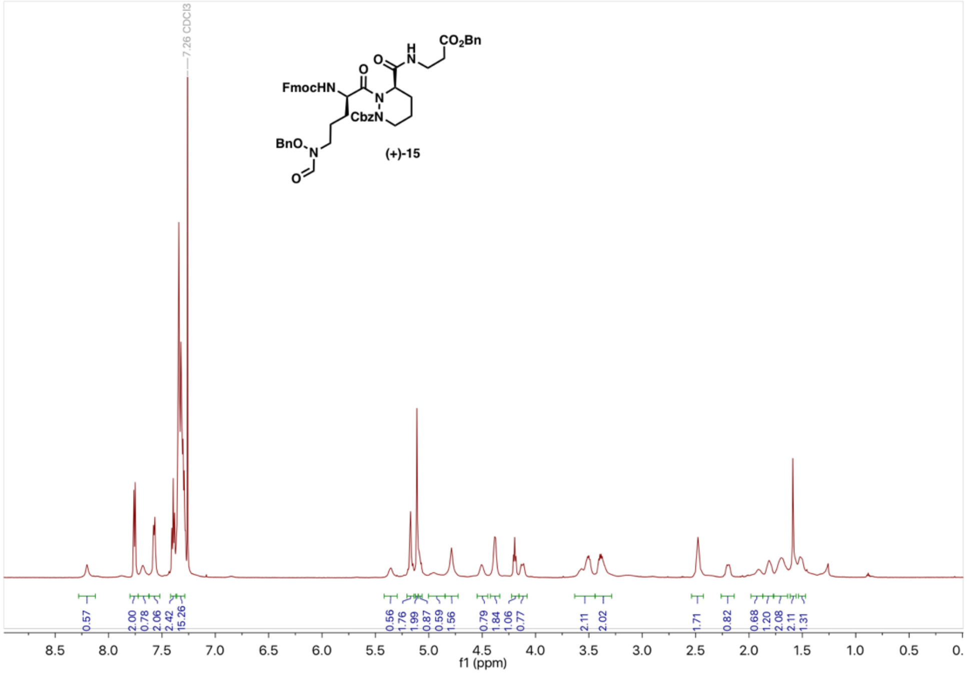

^{1}H NMR (600 MHz, $CDCl_3$) of (+)-15

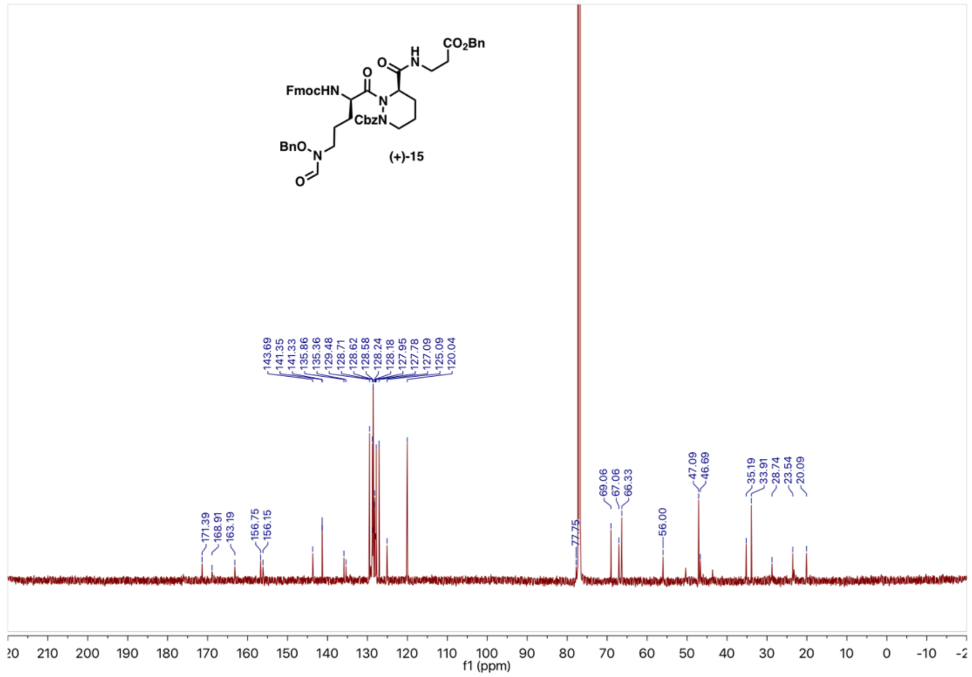

^{13}C NMR (151 MHz, CDCl$_3$) of (+)-15

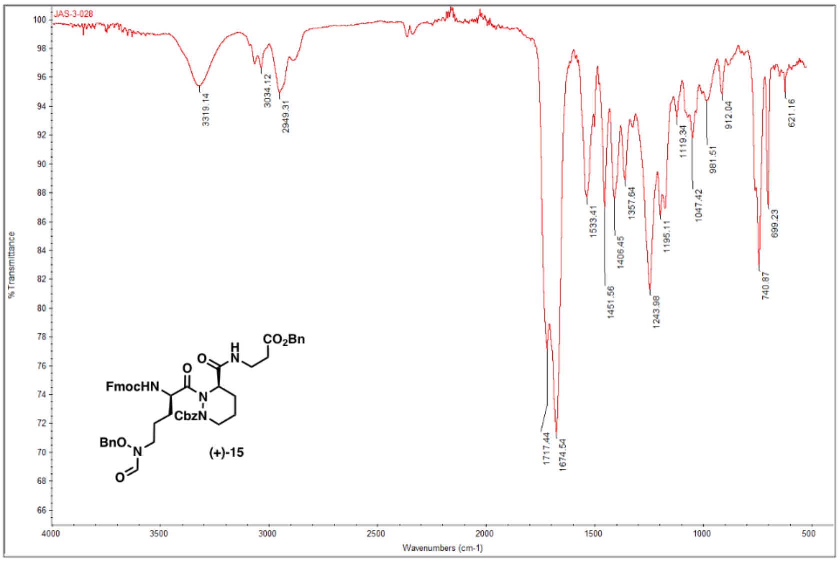

IR (neat) of (+)-15

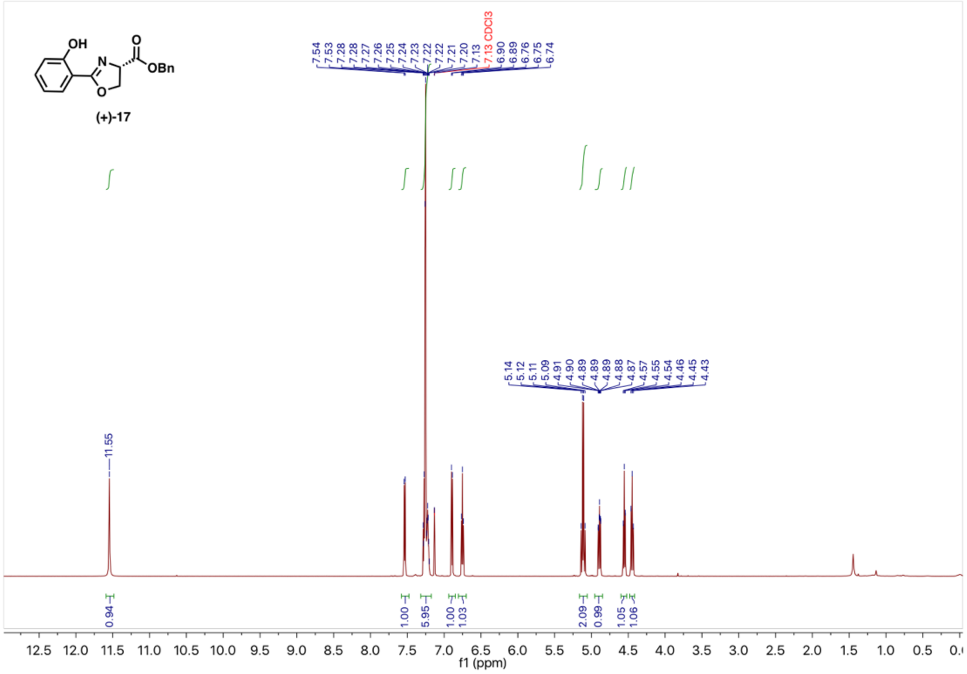

^{1}H NMR (600 MHz, $CDCl_3$) of (+)-17

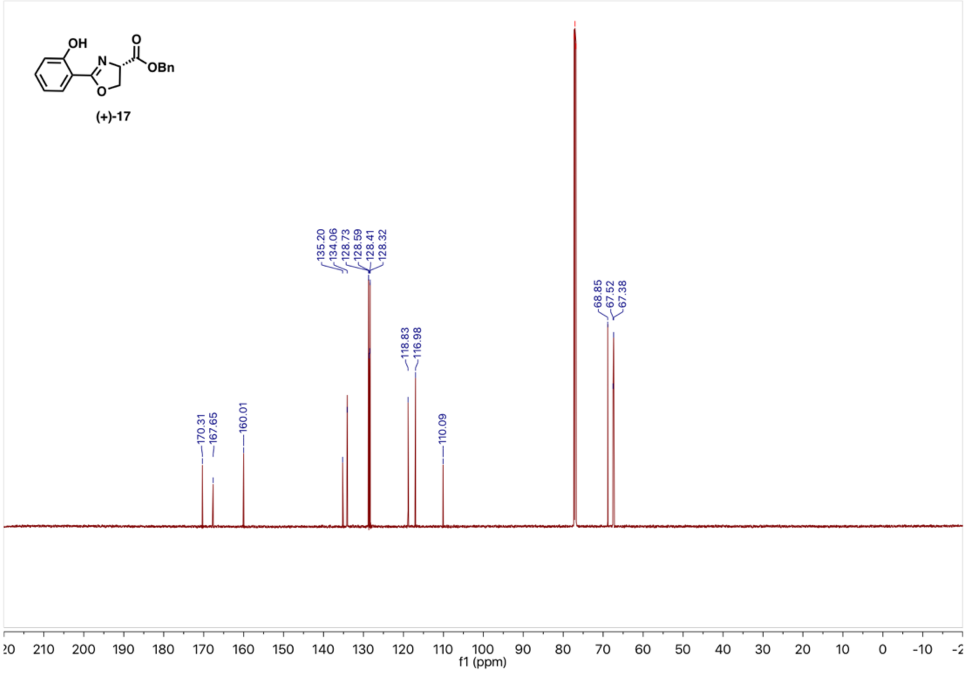

^{13}C NMR (151 MHz, $CDCl_3$) of (+)-17

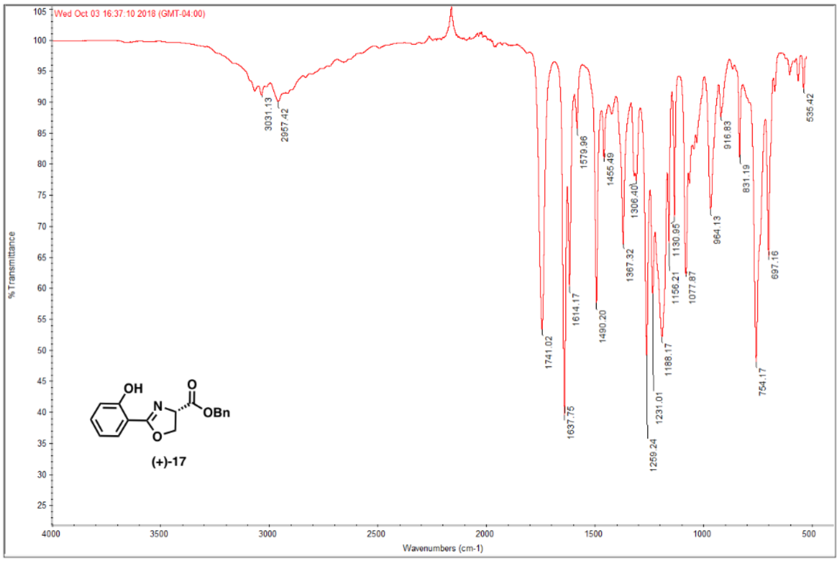

IR (neat) of (+)-17

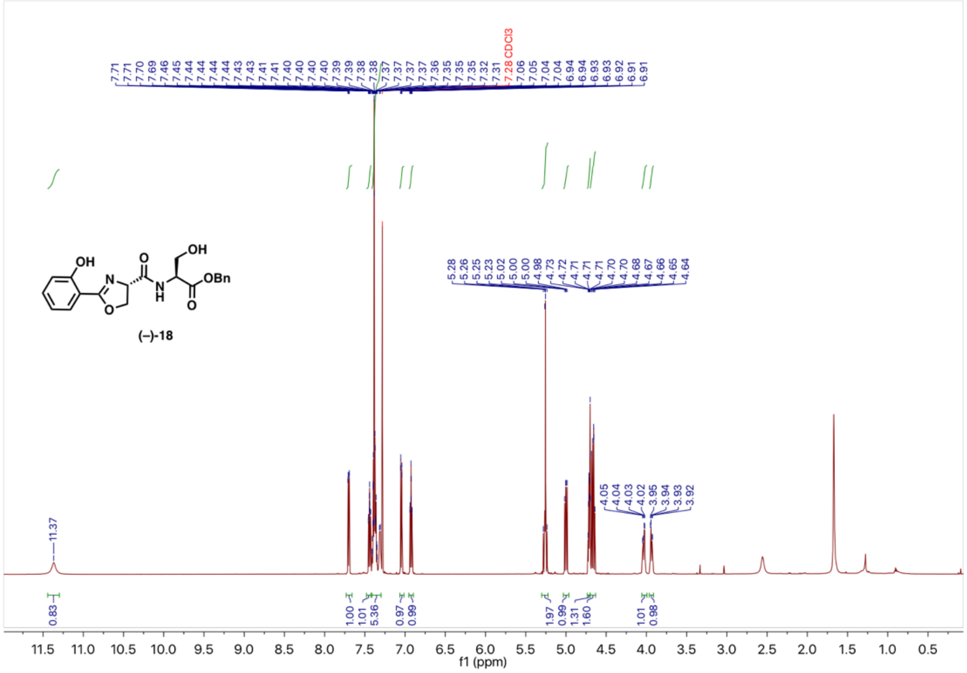

¹H NMR (600 MHz, CDCl₃) of (–)-18

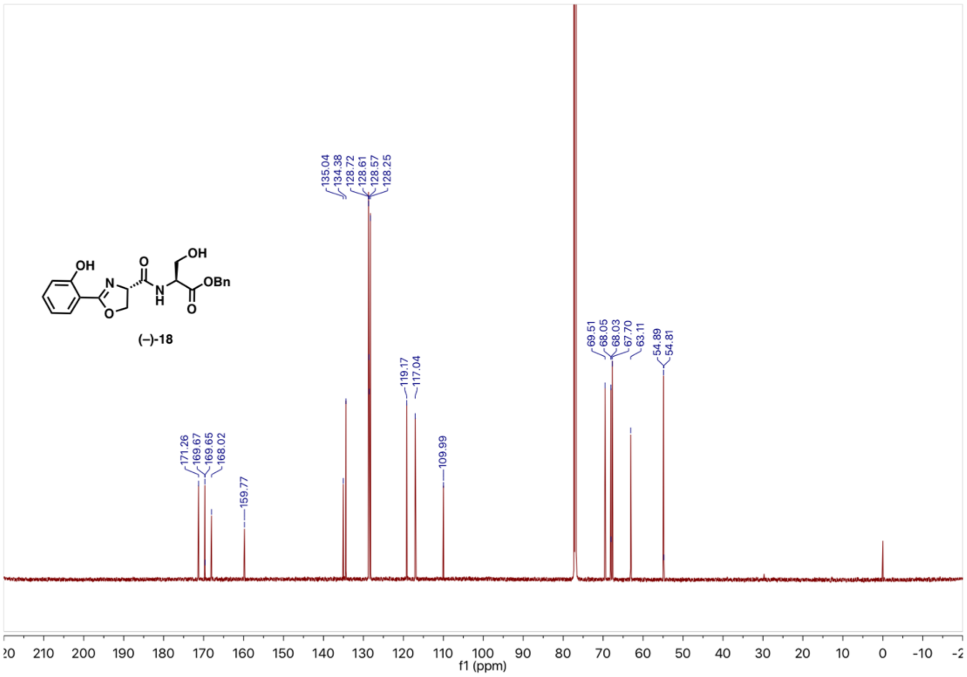

^{13}C NMR (151 MHz, $CDCl_3$) of (–)-18

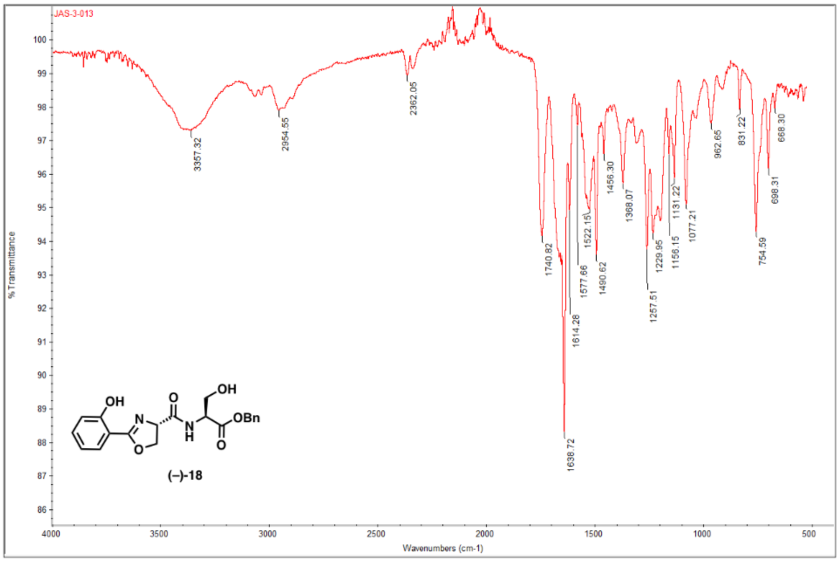

IR (neat) of (–)-18

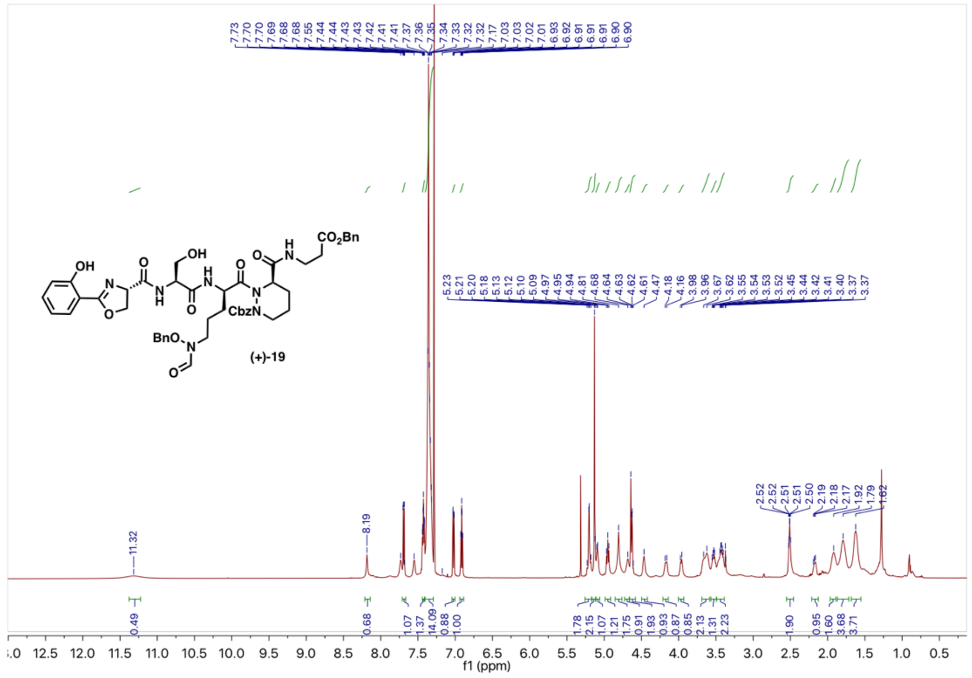

^{1}H NMR (600 MHz, $CDCl_3$) of (+)-19

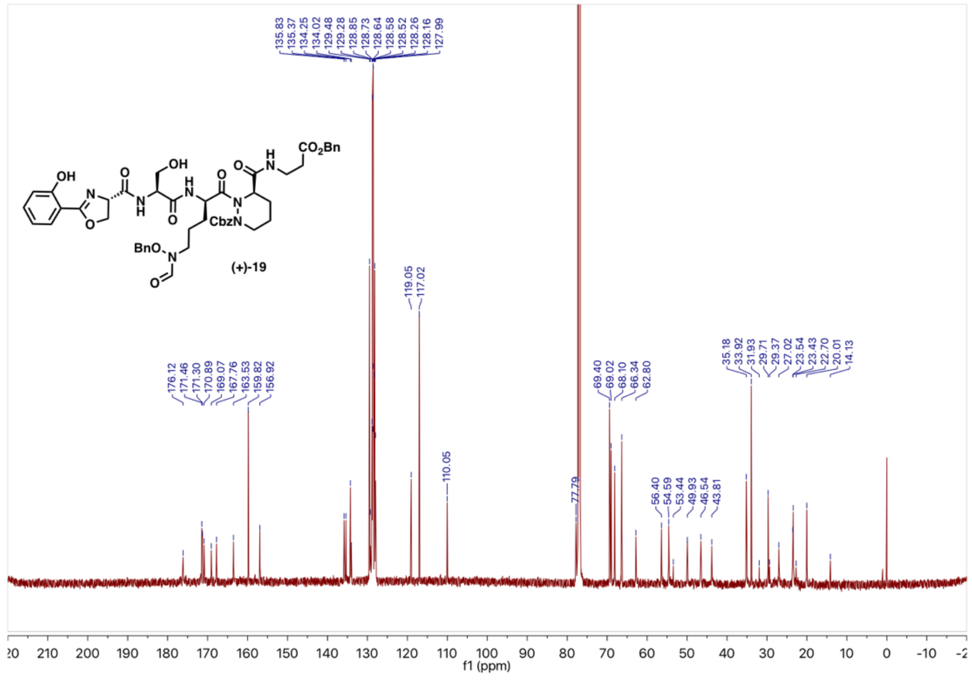

^{13}C NMR (151 MHz, $CDCl_3$) of (+)-19

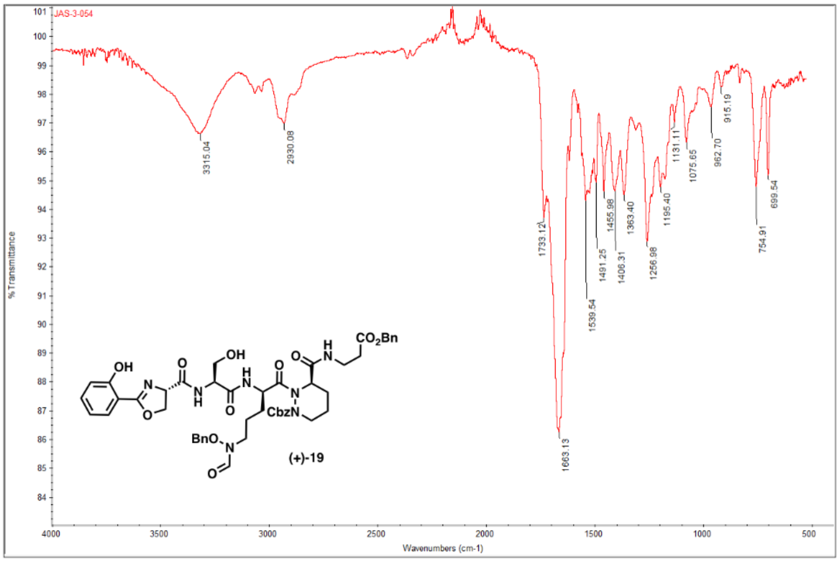

IR (neat) of (+)-19

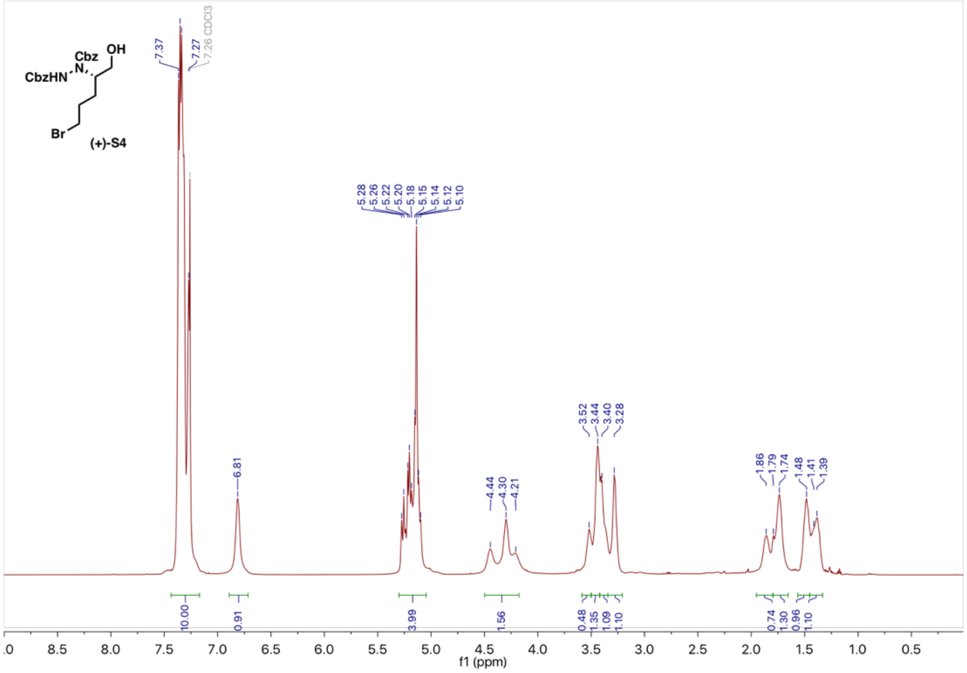

^{1}H-NMR (600 MHz, $CDCl_3$) of (+)-S4

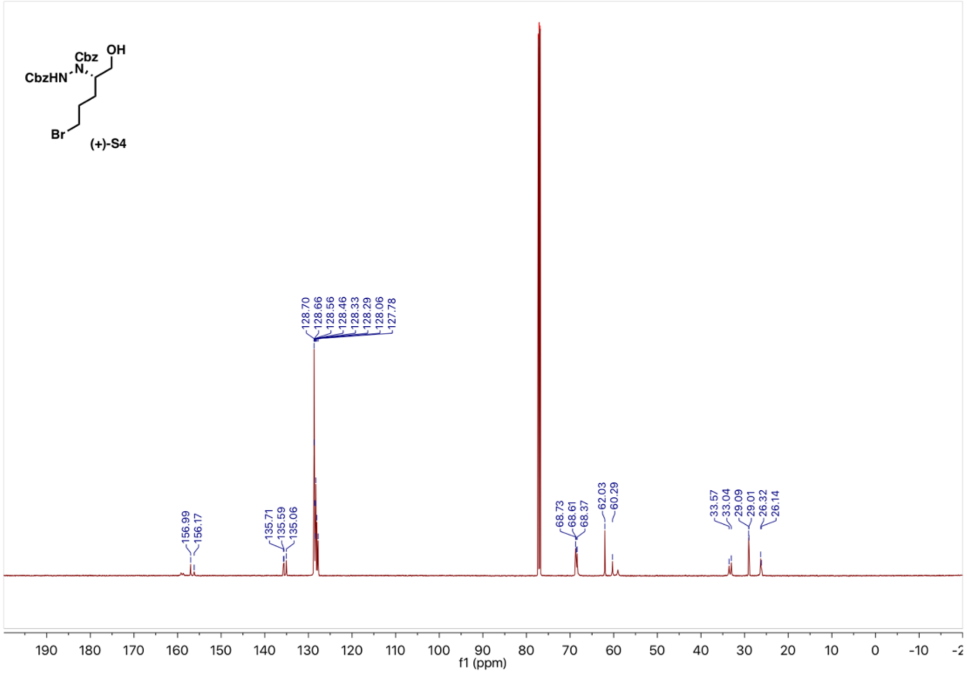

^{13}C NMR (151 MHz, CDCl$_3$) of (+)-S4

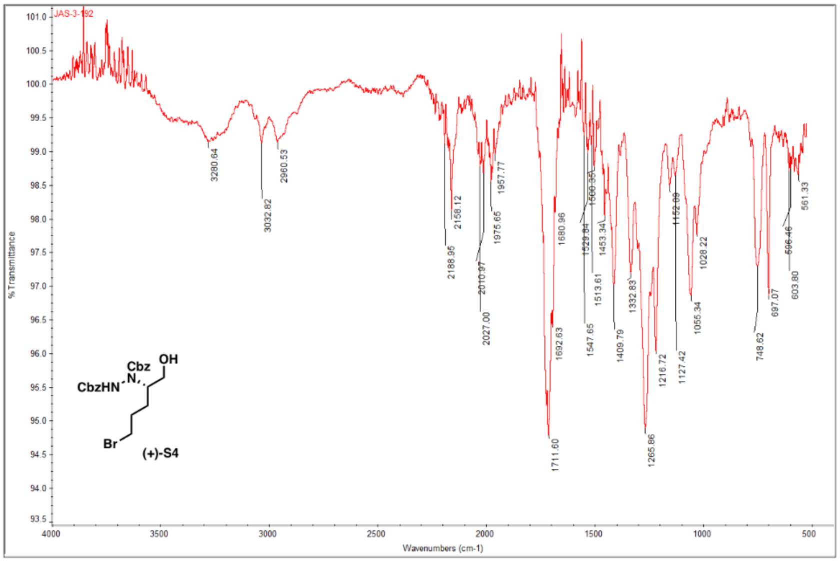

IR (neat) of (+)-S4

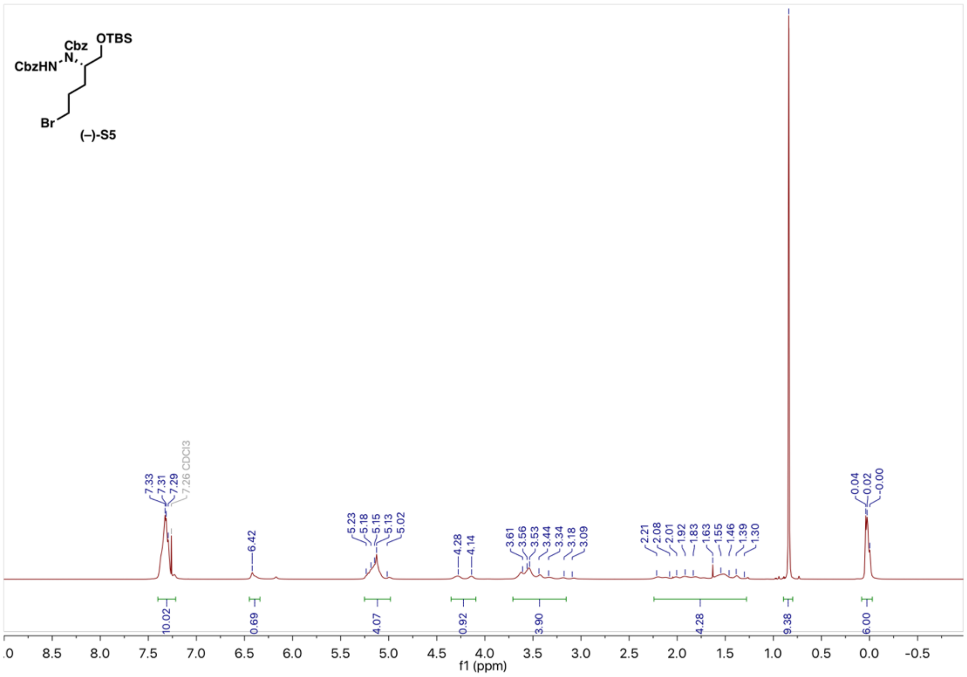

^{1}H NMR (600 MHz, CDCl₃) of (–)-S5

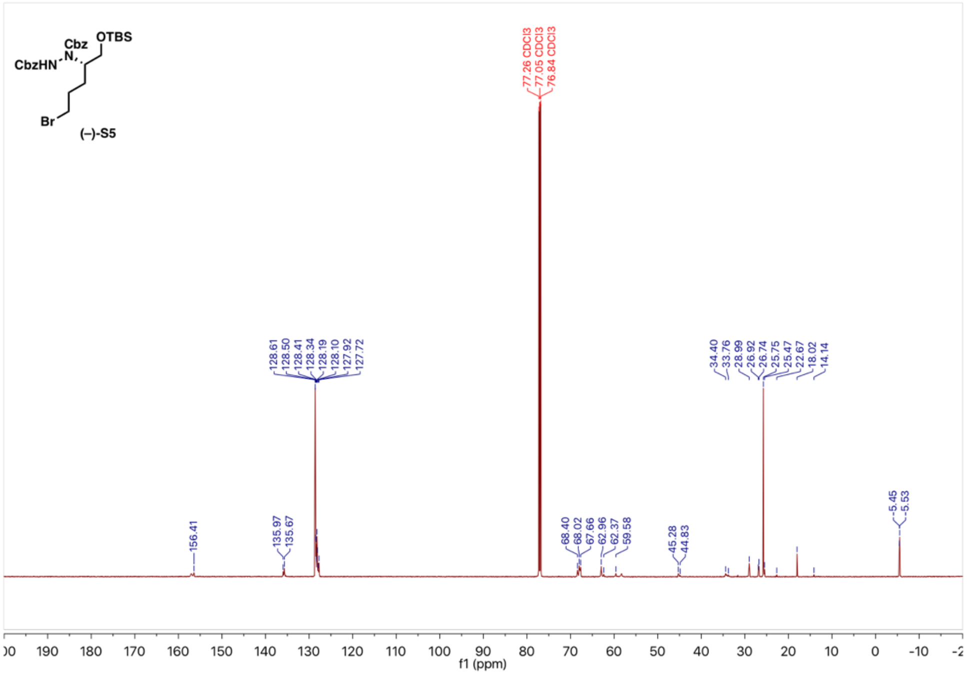

^{13}C NMR (151 MHz, CDCl₃) of (–)-S5

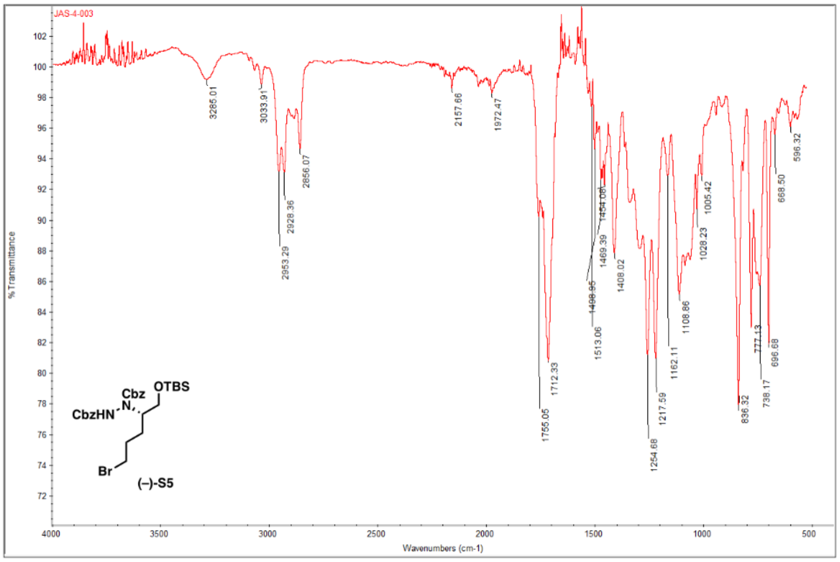

IR (neat) of (–)-S5

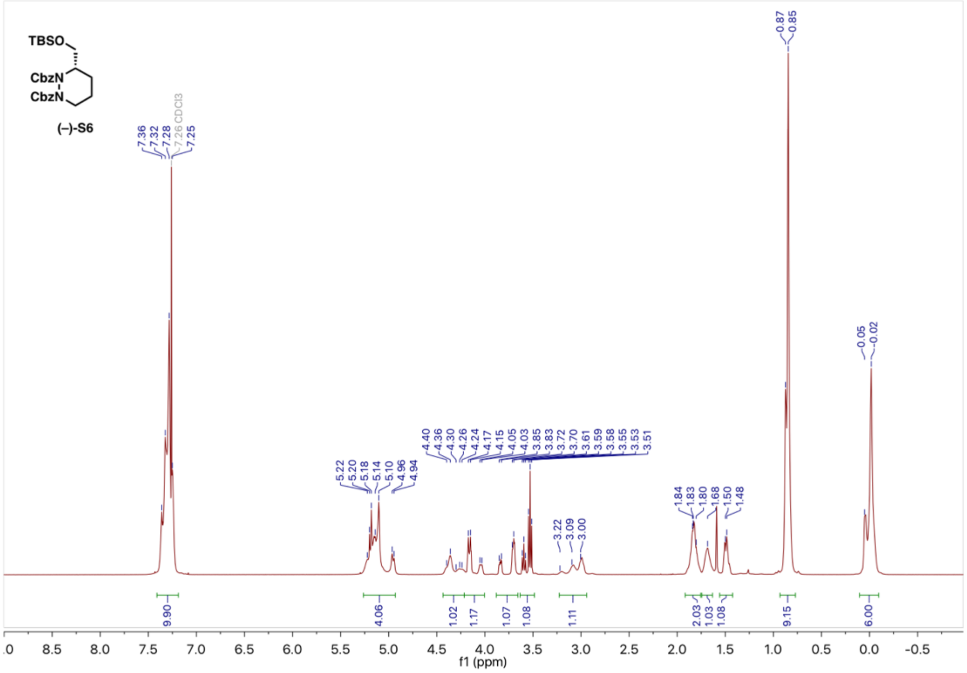

^{1}H NMR (600 MHz, $CDCl_3$) of (–)-S6

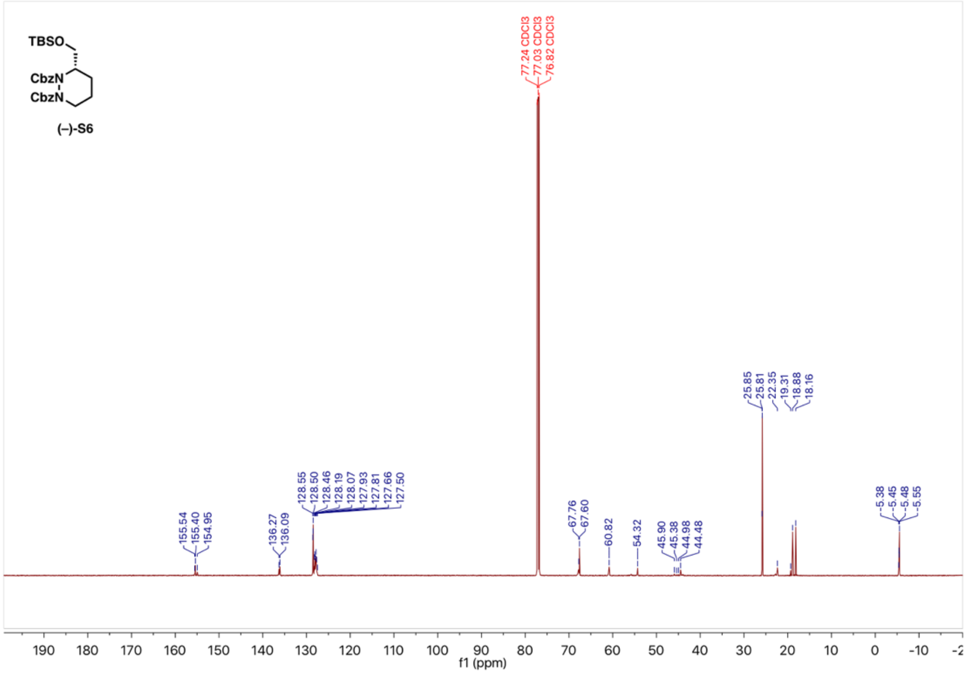

^{13}C NMR (151 MHz, $CDCl_3$) of (–)-S6

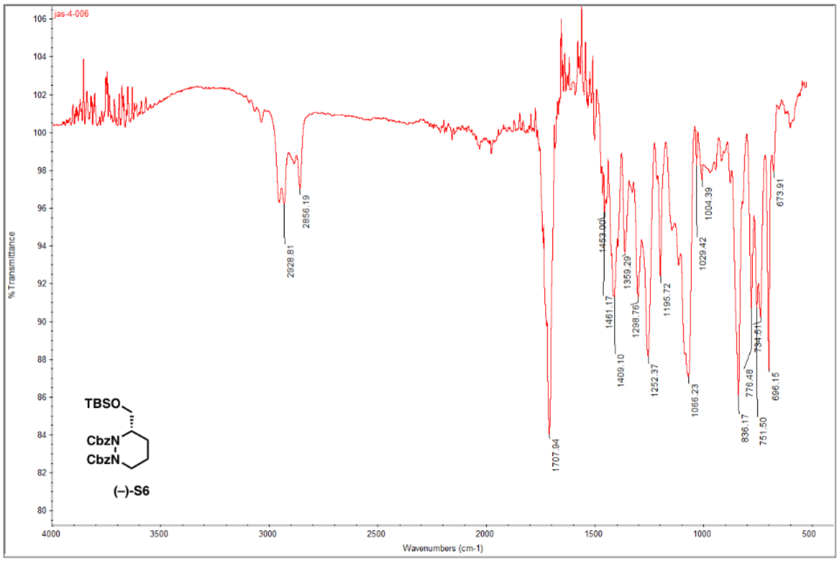

IR (neat) of (–)-S6

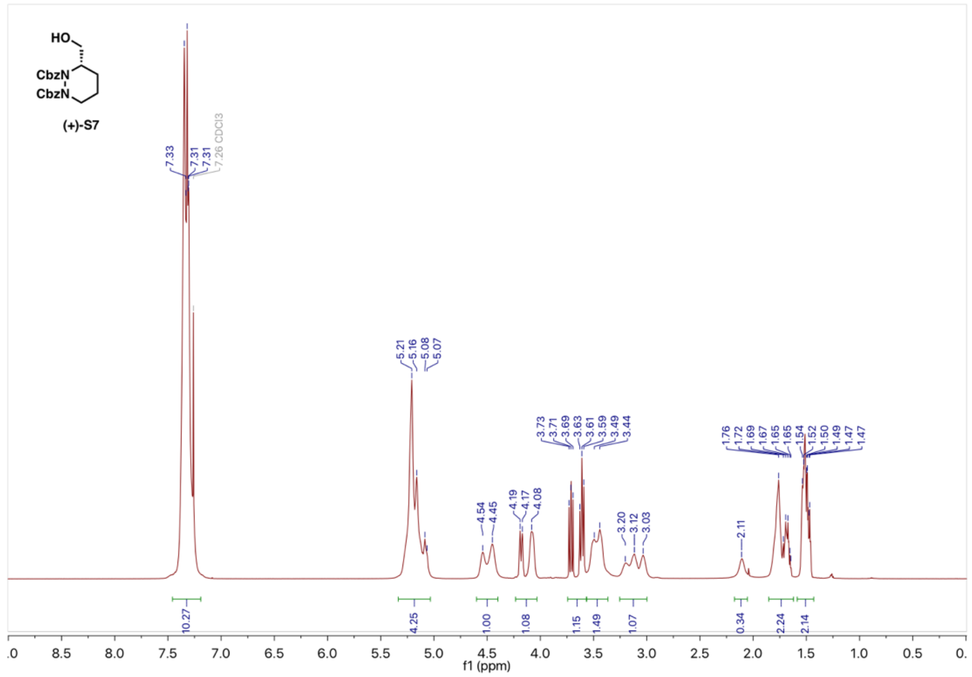

^{1}H NMR (600 MHz, $CDCl_3$) of (+)-S7

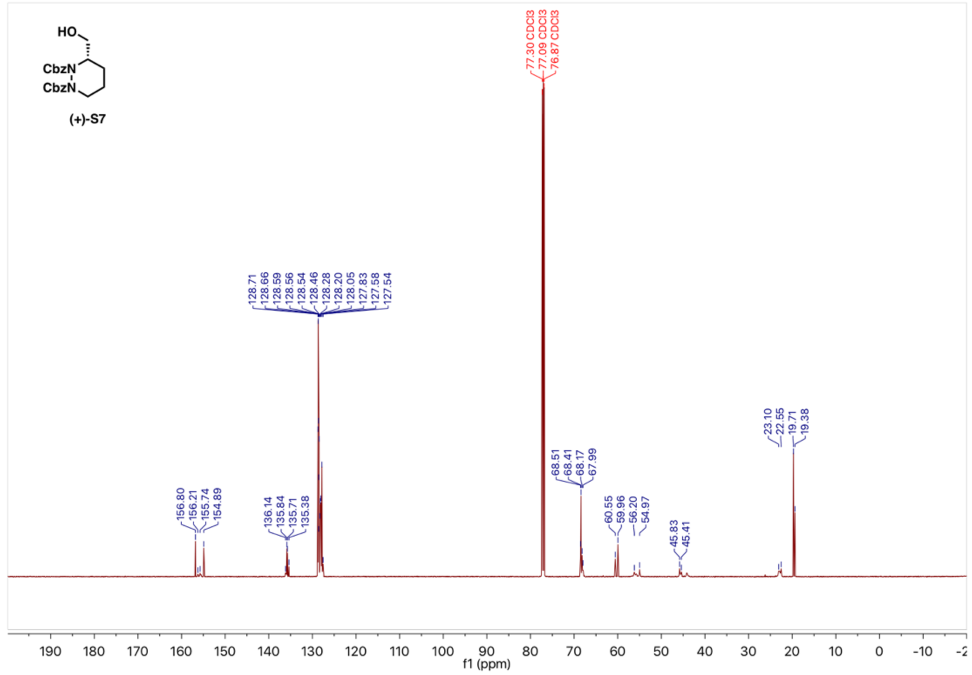

^{13}C NMR (151 MHz, $CDCl_3$) of (+)-S7

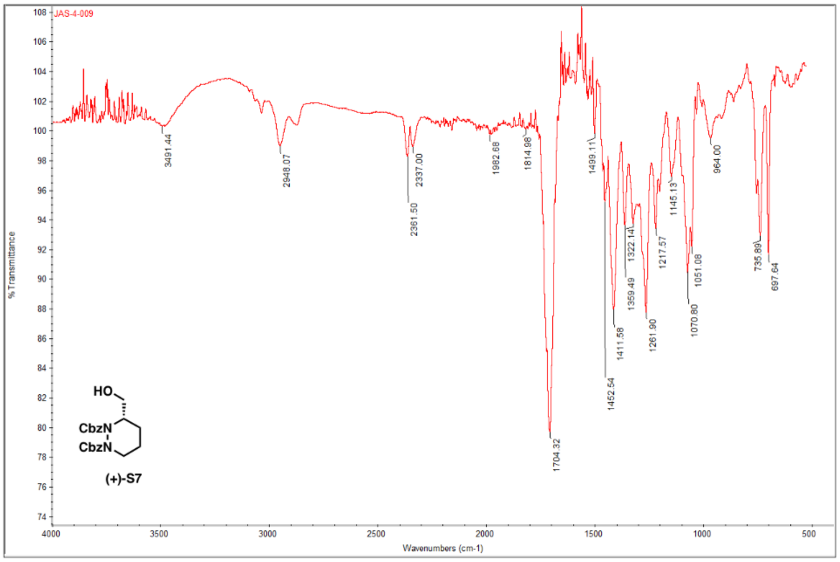

IR (neat) of (+)-S7

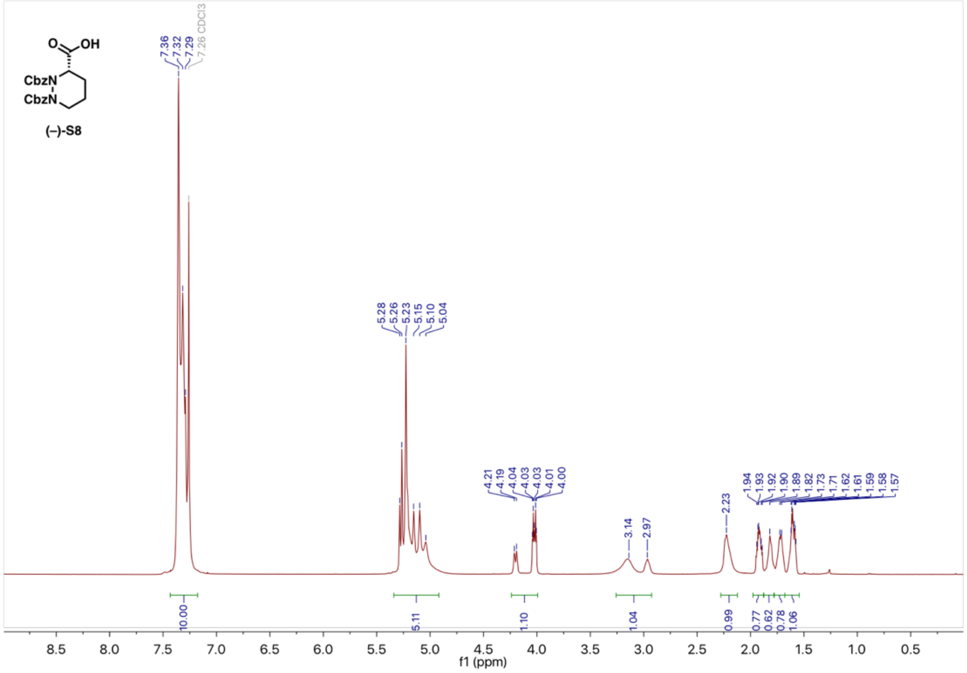

^{1}H NMR (600 MHz, $CDCl_3$) of (–)-S8

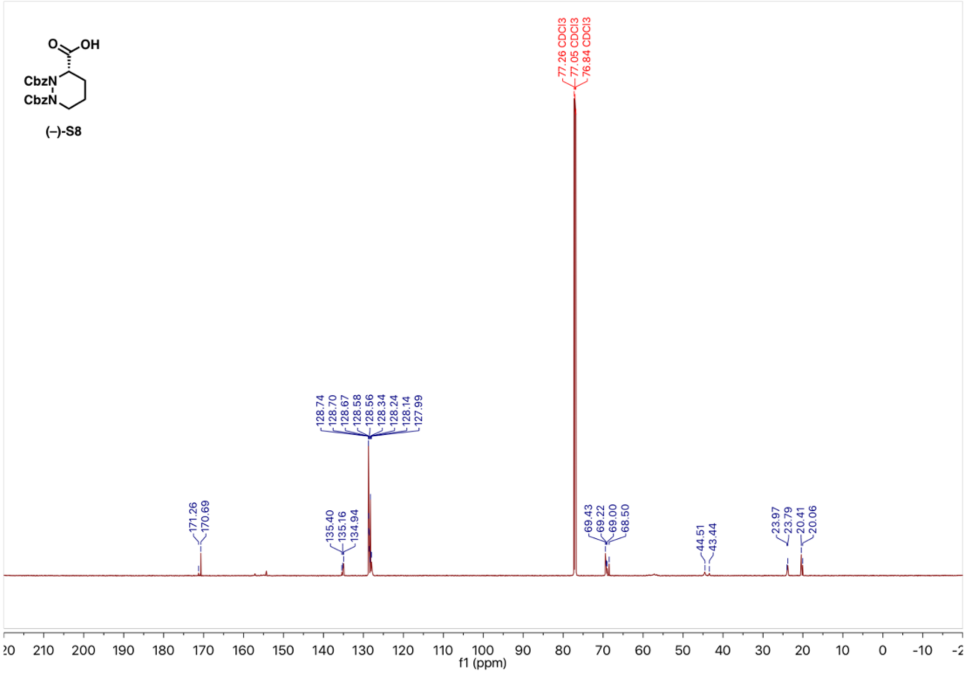

^{13}C NMR (151 MHz, $CDCl_3$) of (–)-S8

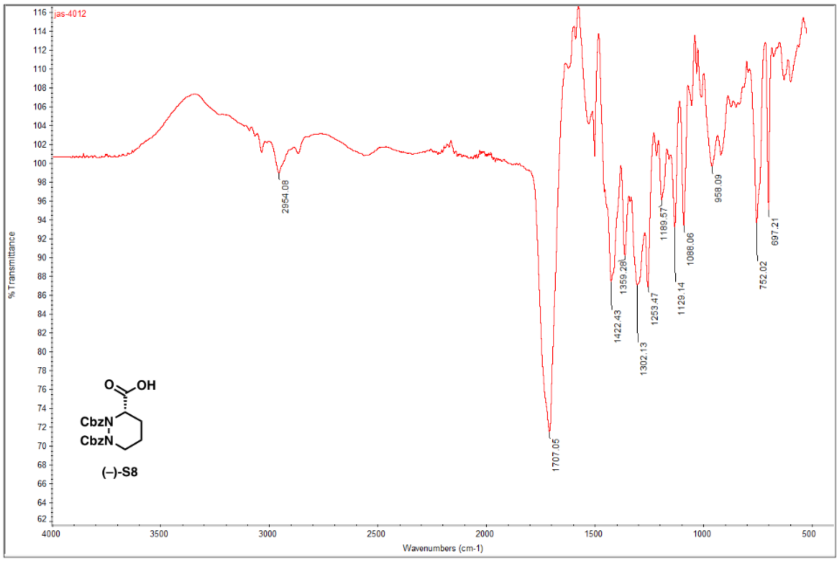

IR (neat) of (–)-S8

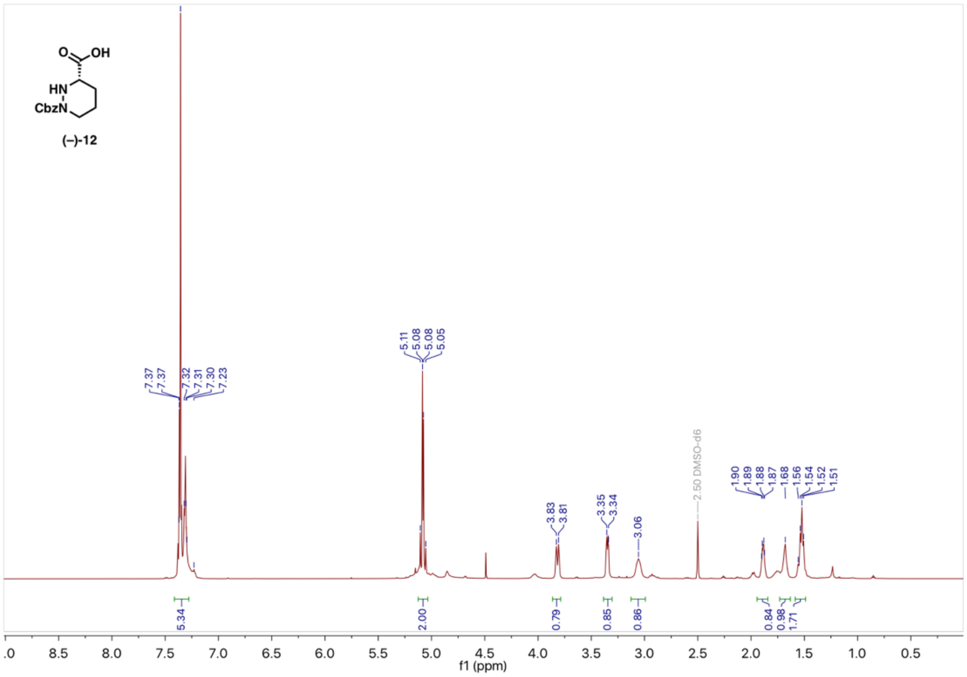

^{1}H NMR (600 MHz, DMSO-d_6) of (–)-12

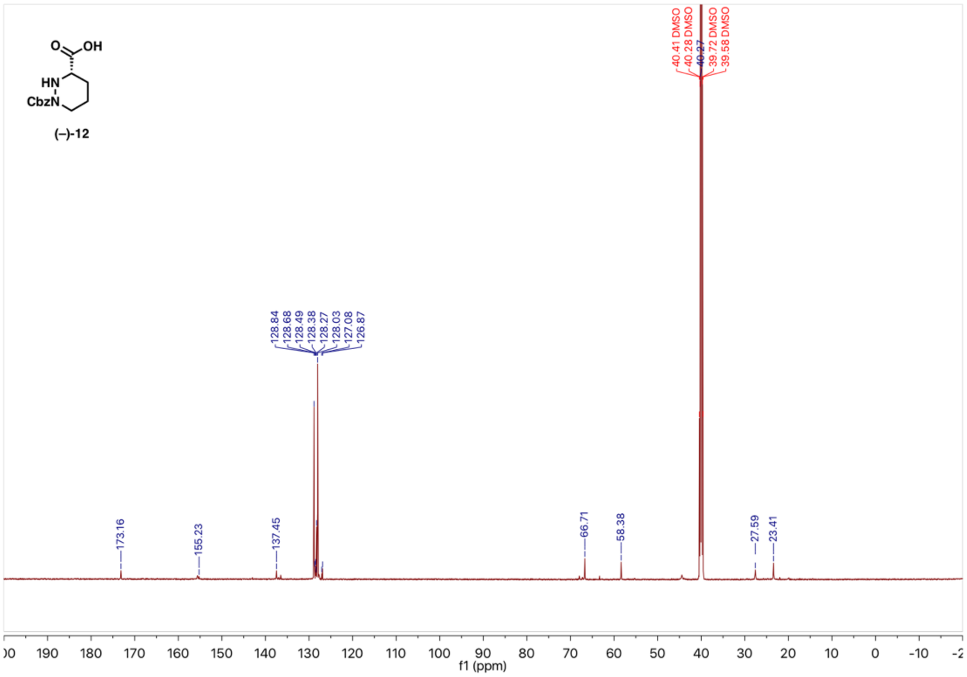

^{13}C NMR (151 MHz, DMSO-d_6) of (–)-12

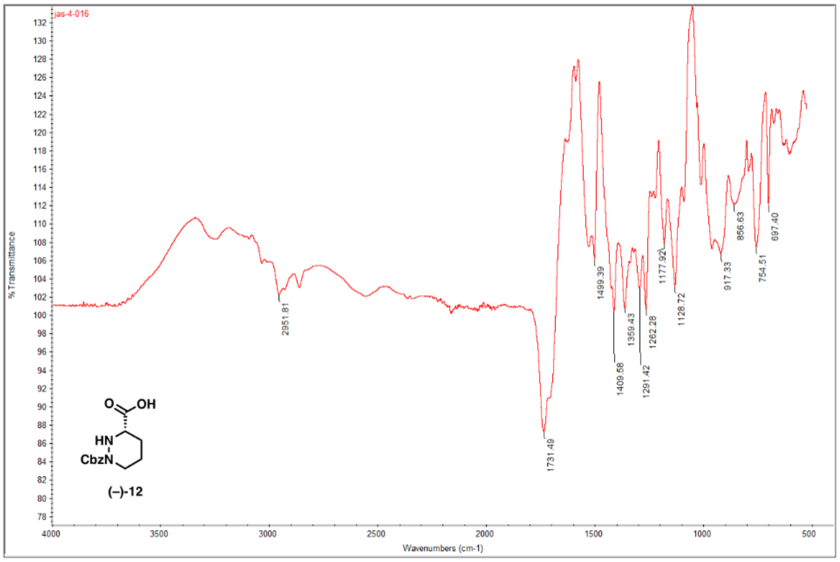

IR (neat) of (–)-12

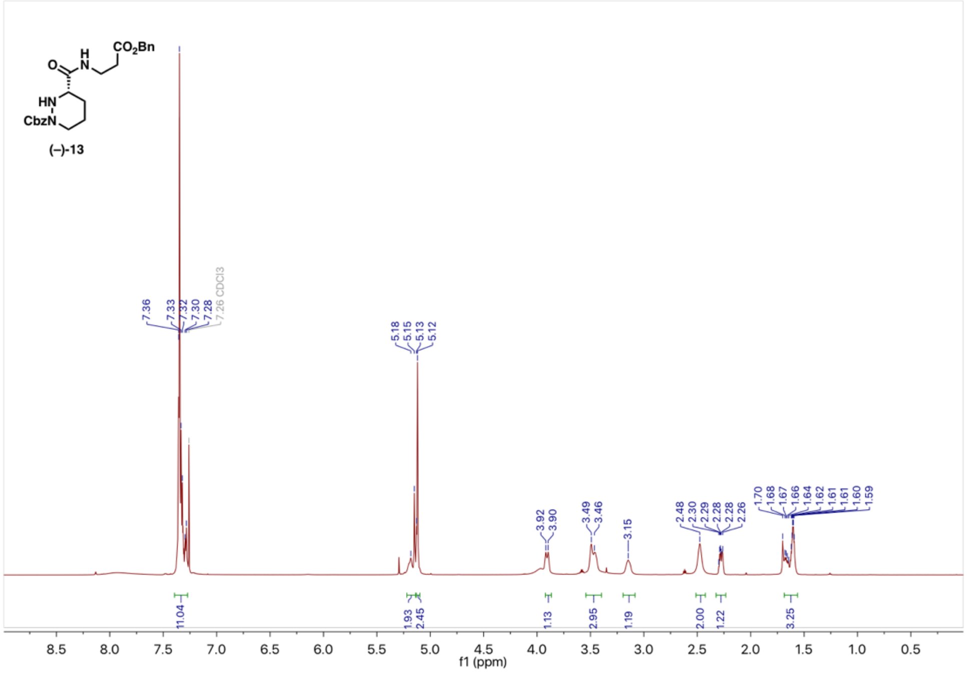

^{1}H NMR (600 MHz, $CDCl_3$) of (–)-13

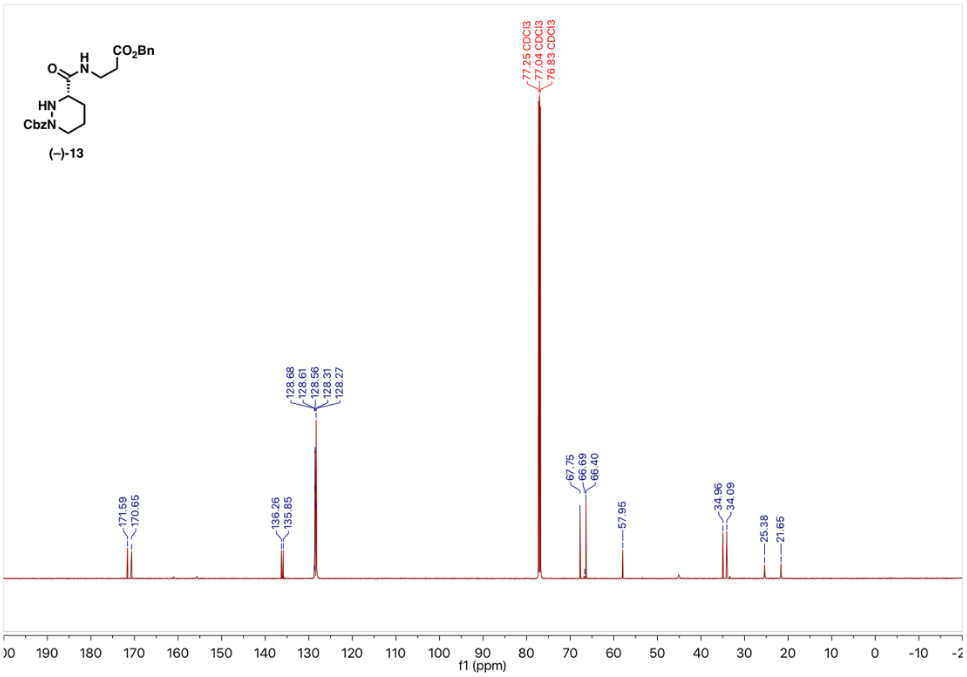

^{13}C NMR (151 MHz, CDCl3) of (–)-13

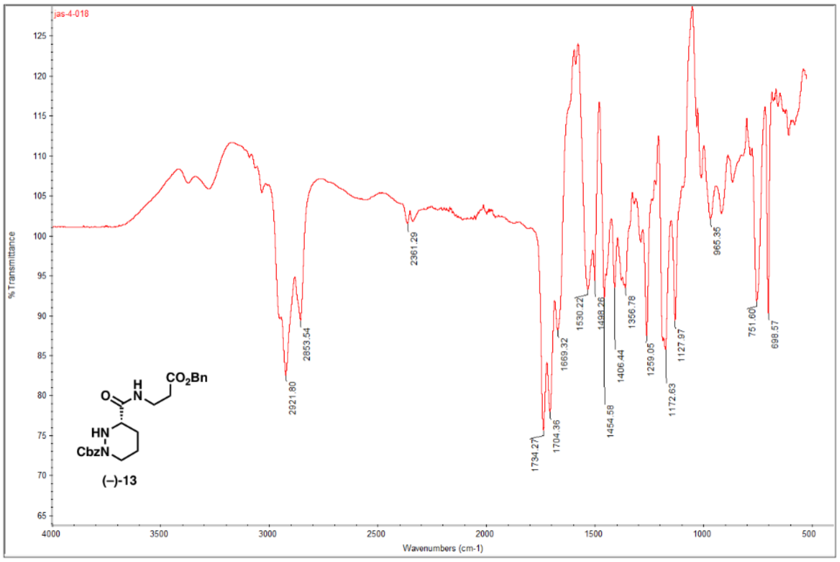

IR (neat) of (–)-13

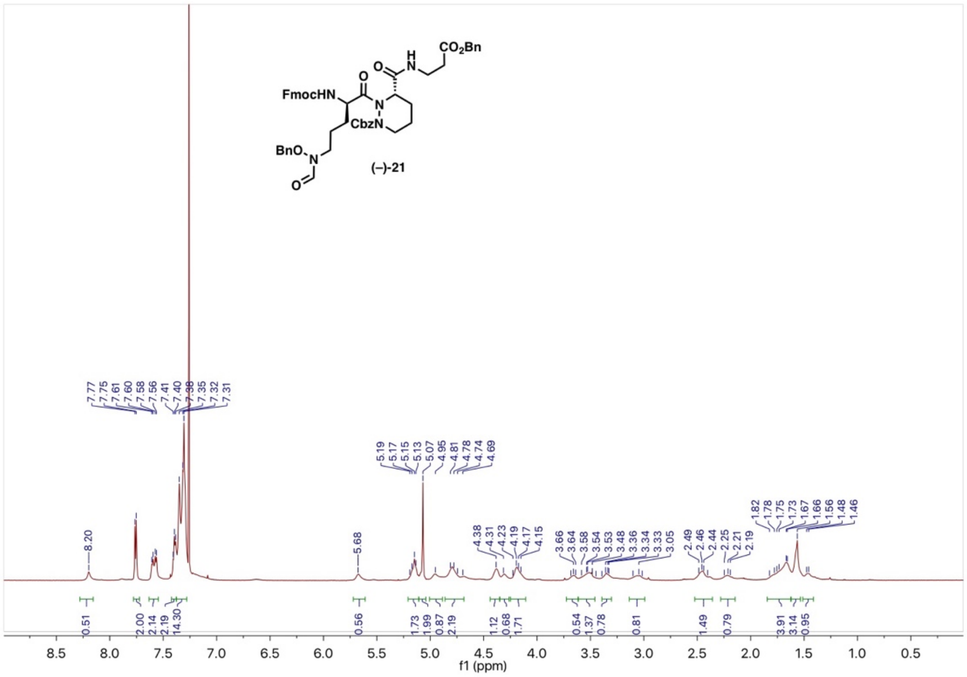

^{1}H NMR (600 MHz, $CDCl_3$) of (–)-21

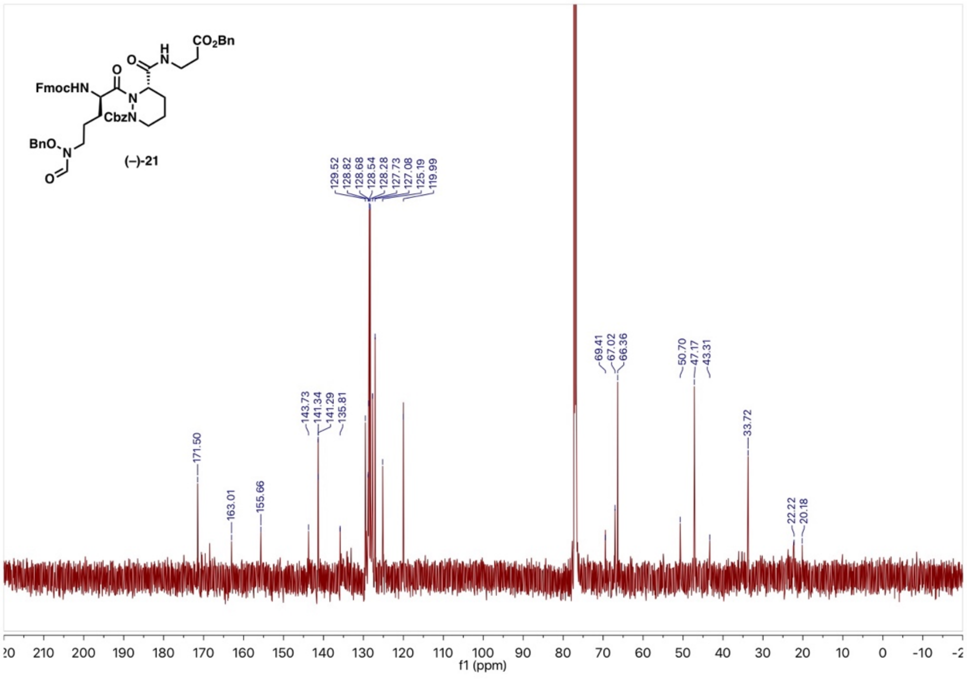

^{13}C NMR (151 MHz, $CDCl_3$) of (–)-21

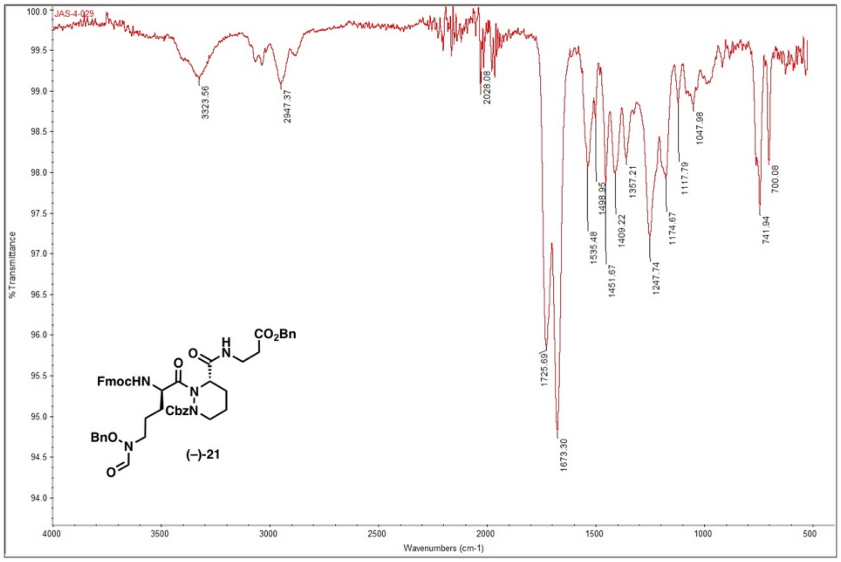

IR (neat) of (–)-21

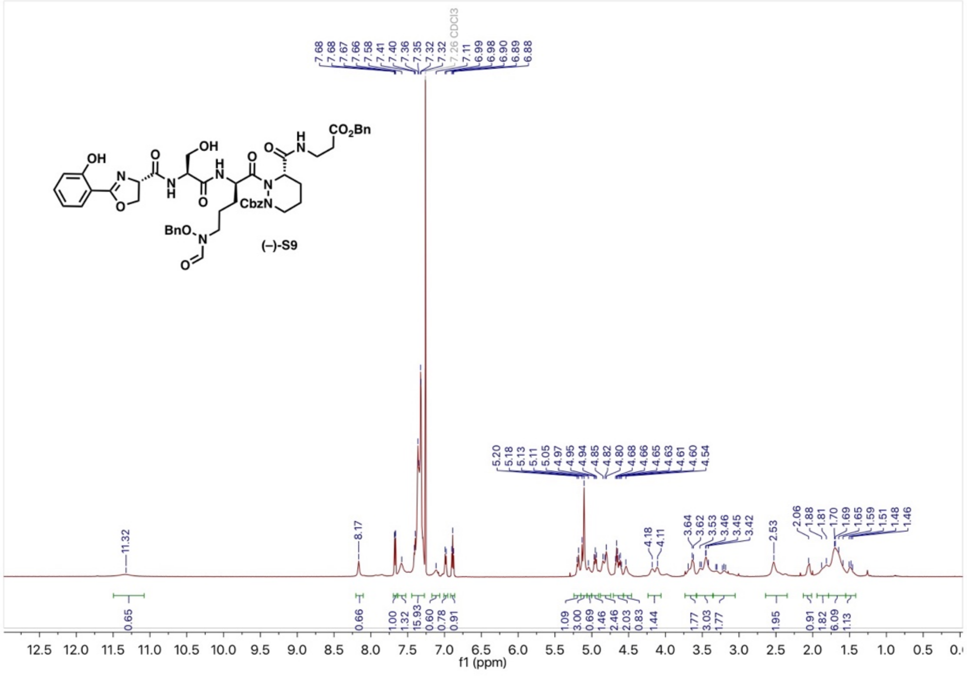

^{1}H NMR (600 MHz, $CDCl_3$) of (–)-S9

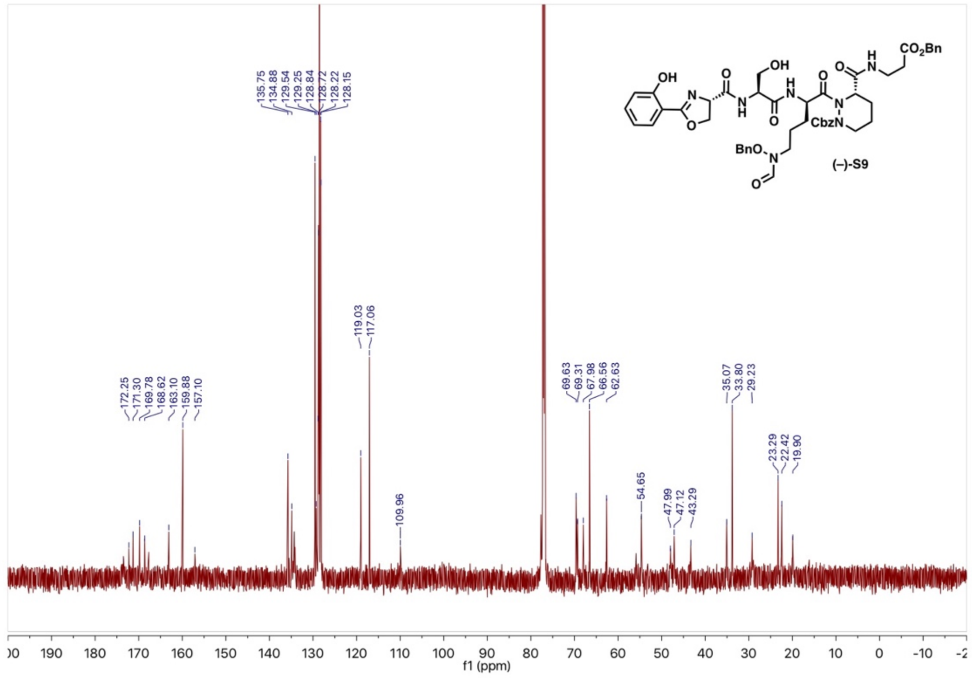

^{13}C NMR (151 MHz, $CDCl_3$) of (–)-S9

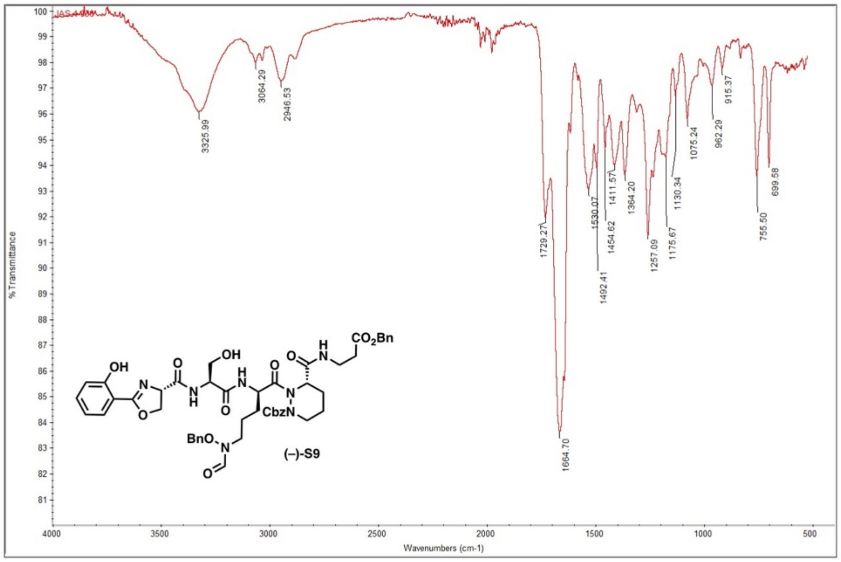

IR (neat) of (–)-S9

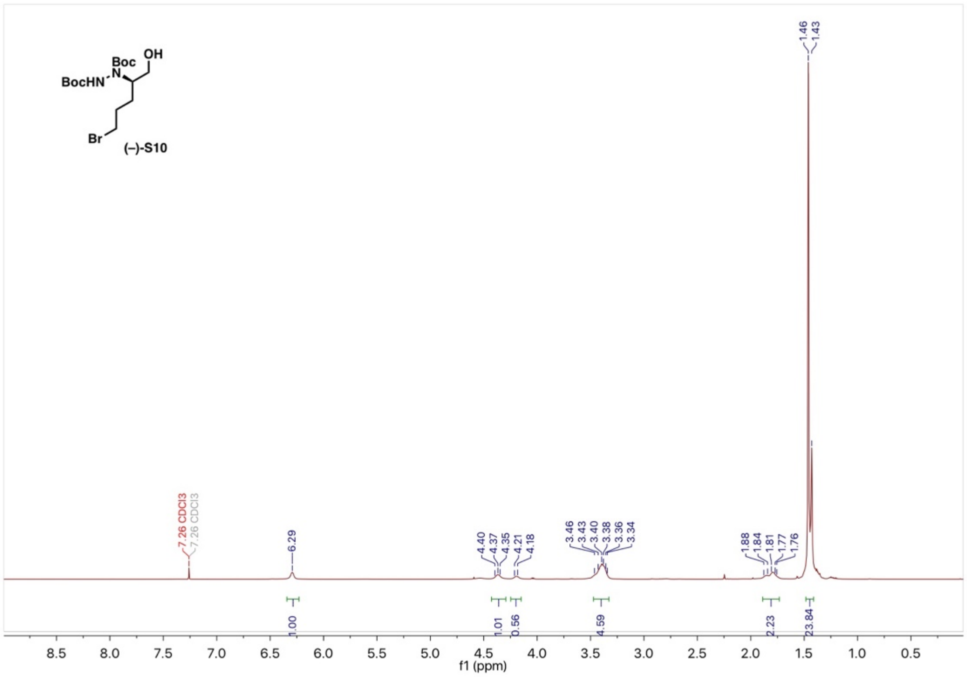

^{1}H-NMR (600 MHz, CDCl$_3$) of (–)-S10

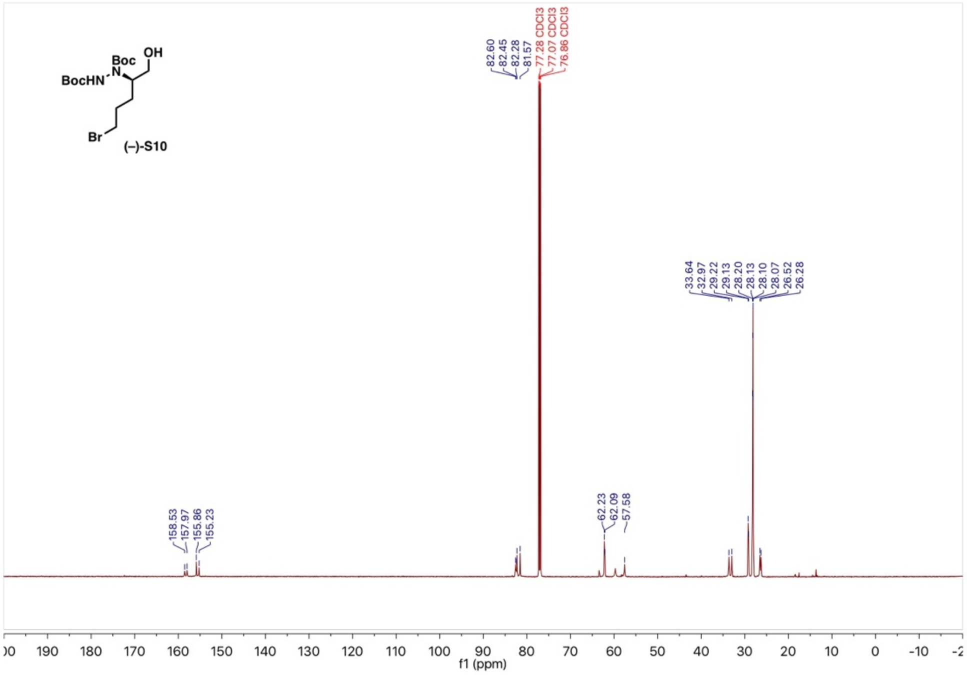

^{13}C NMR (151 MHz, CDCl$_3$) of (–)-S10

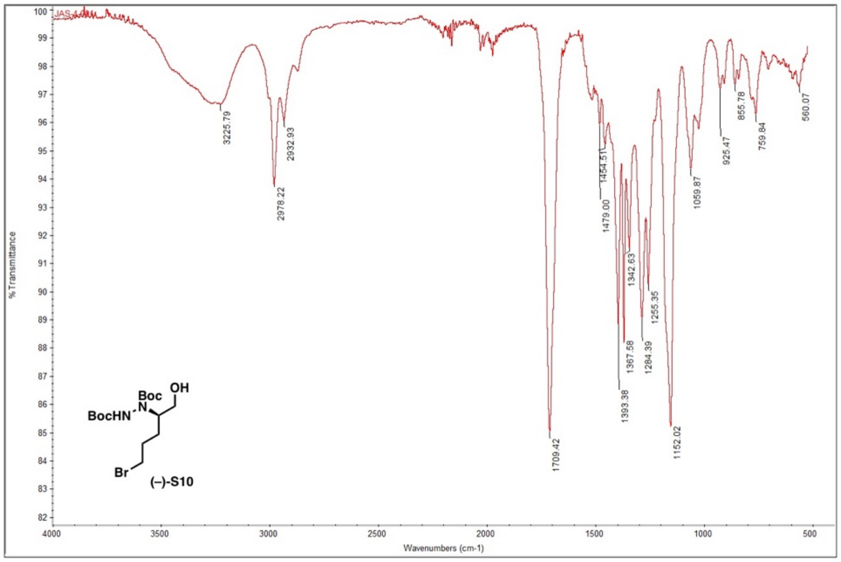

IR (neat) of (–)-S10

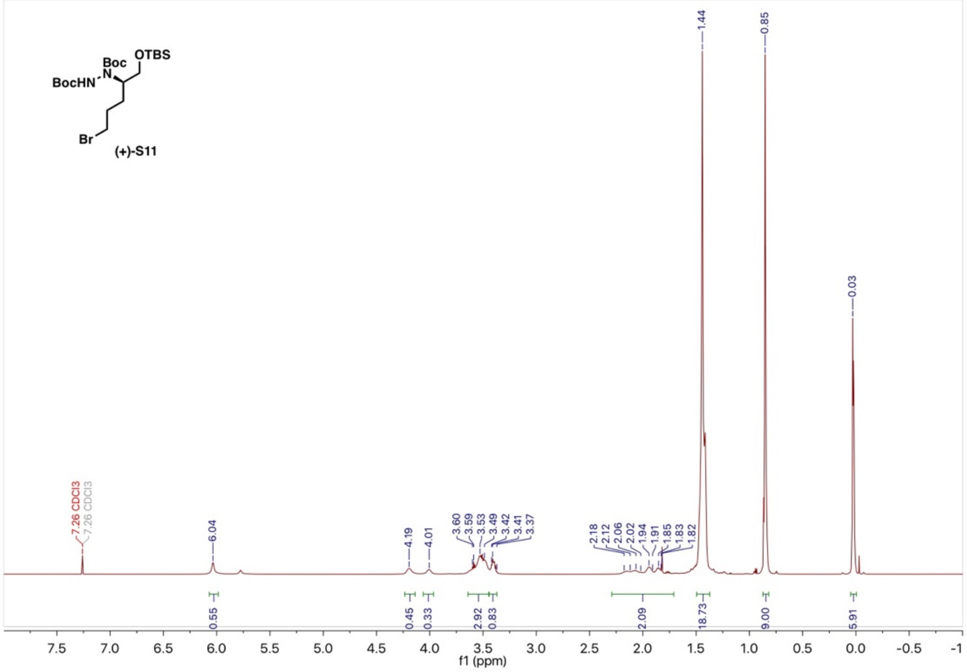

^{1}H NMR (600 MHz, CDCl$_3$) of (+)-S11

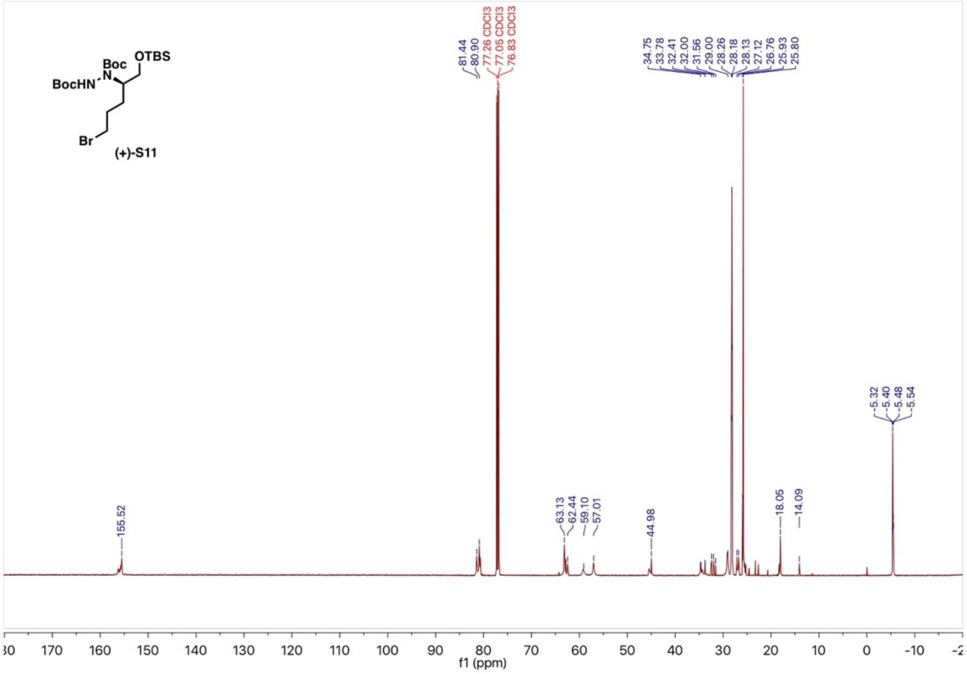

^{13}C NMR (151 MHz, CDCl$_3$) of (+)-S11

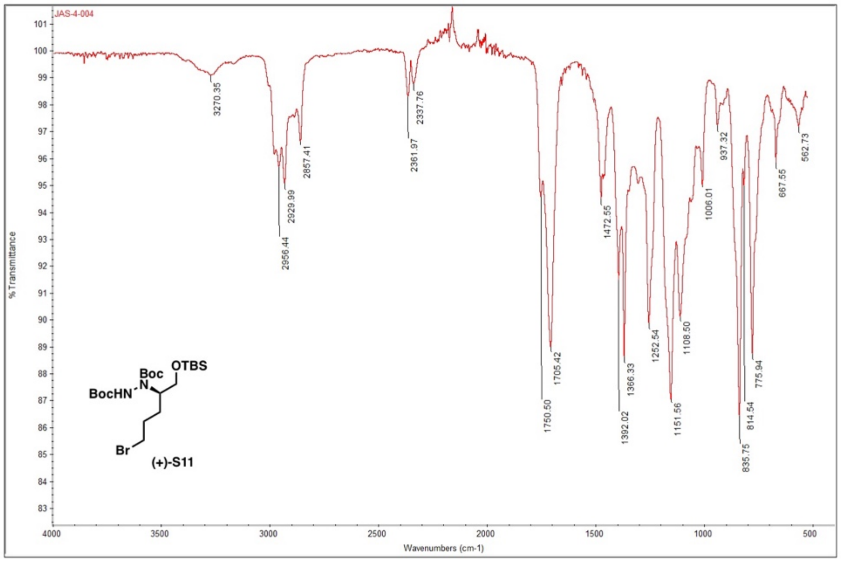

IR (neat) of (+)-S11

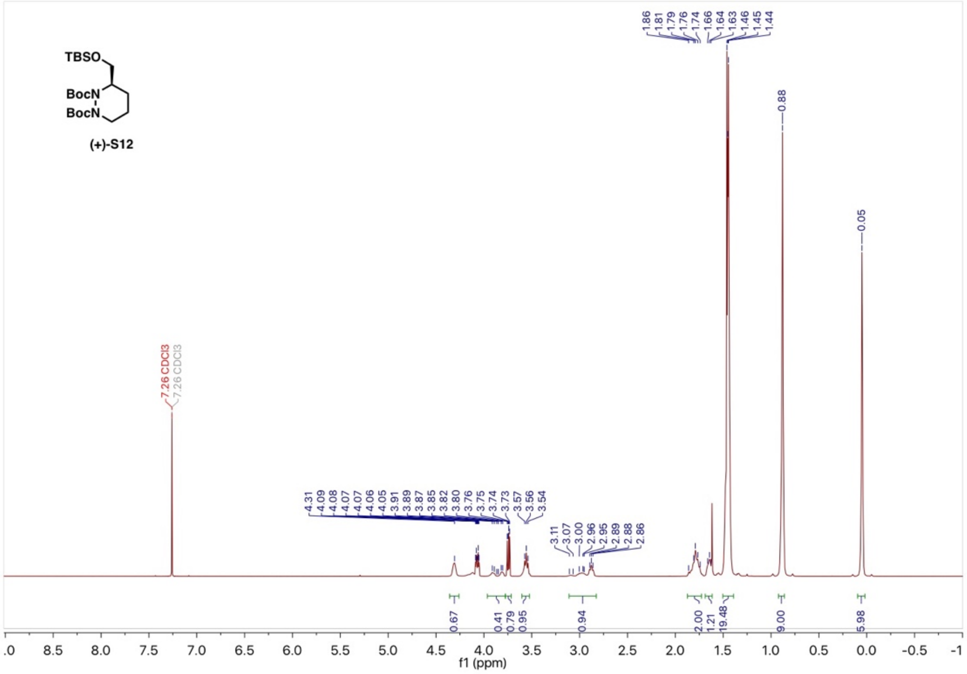

^{1}H NMR (600 MHz, $CDCl_3$) of (+)-S12

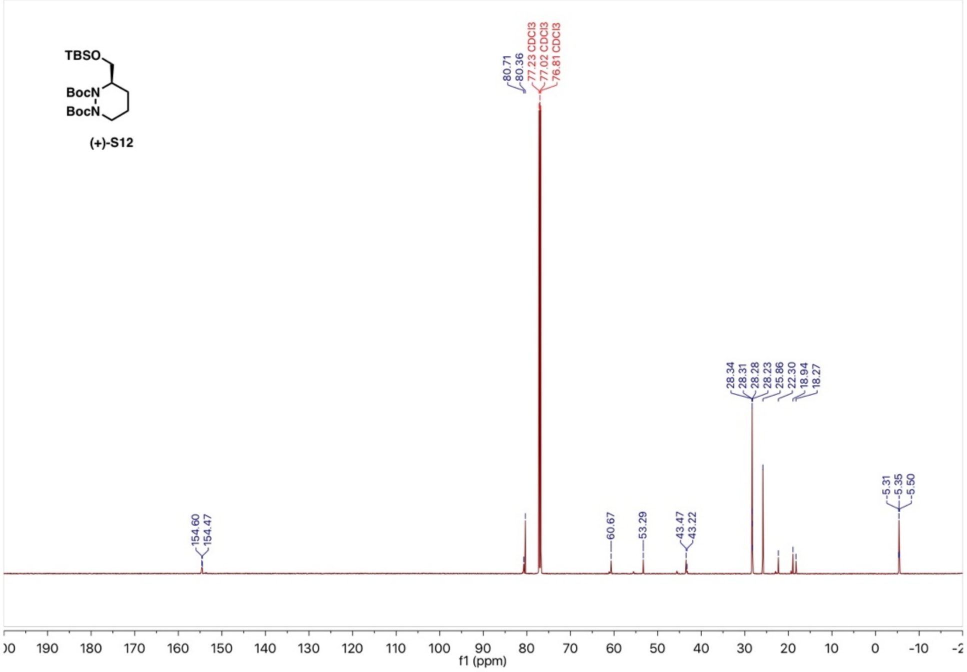

^{13}C NMR (151 MHz, $CDCl_3$) of (+)-S12

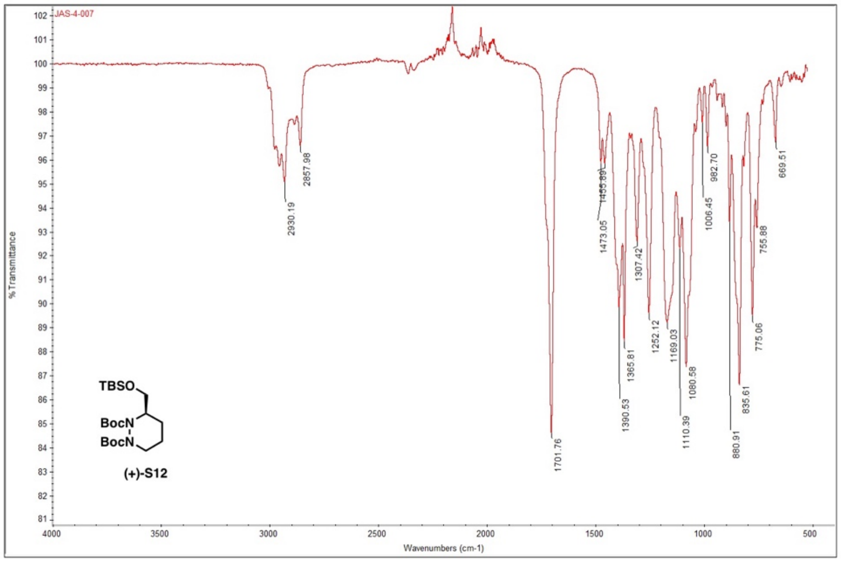

IR (neat) of (+)-S12

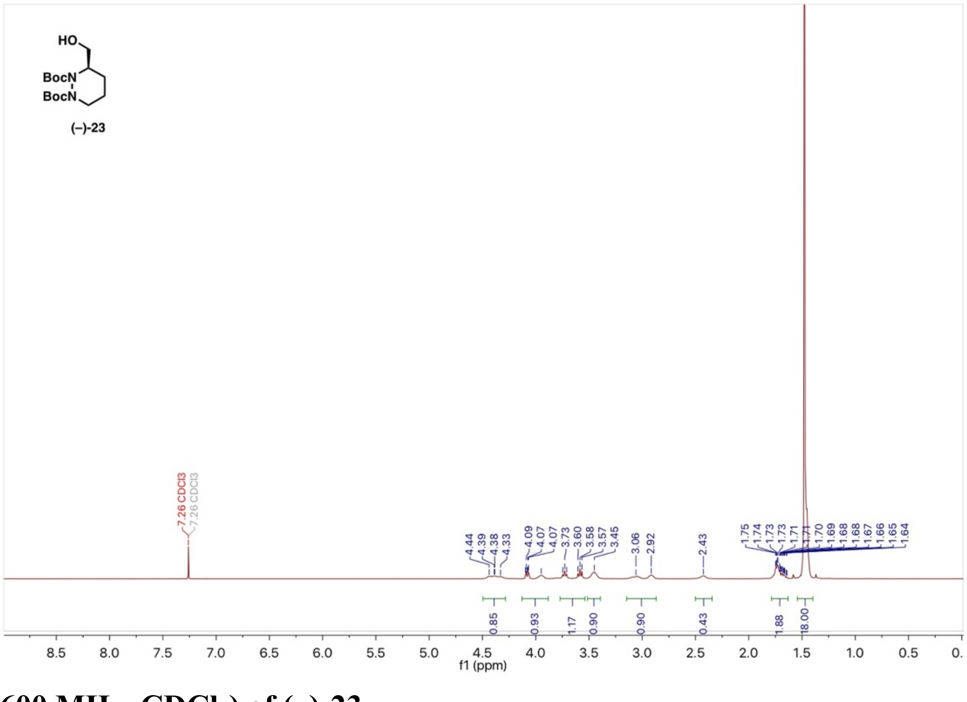

^{1}H NMR (600 MHz, $CDCl_3$) of (–)-23

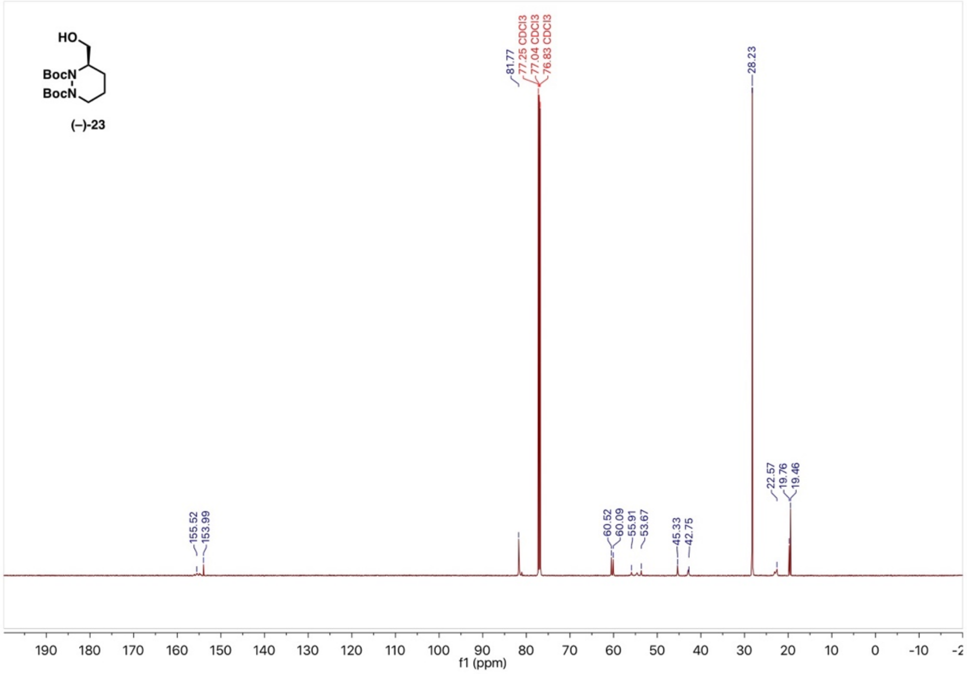

^{13}C NMR (151 MHz, CDCl$_3$) of (–)-23

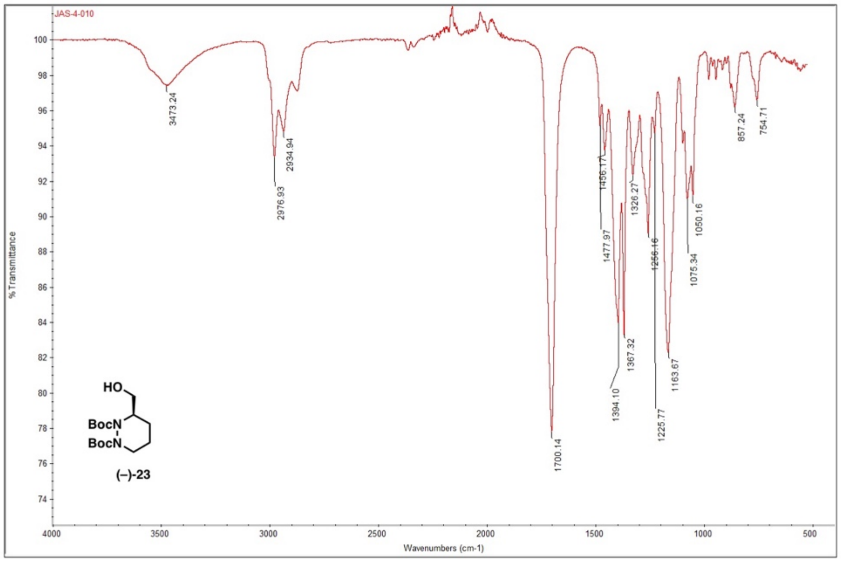

IR (neat) of (–)-23

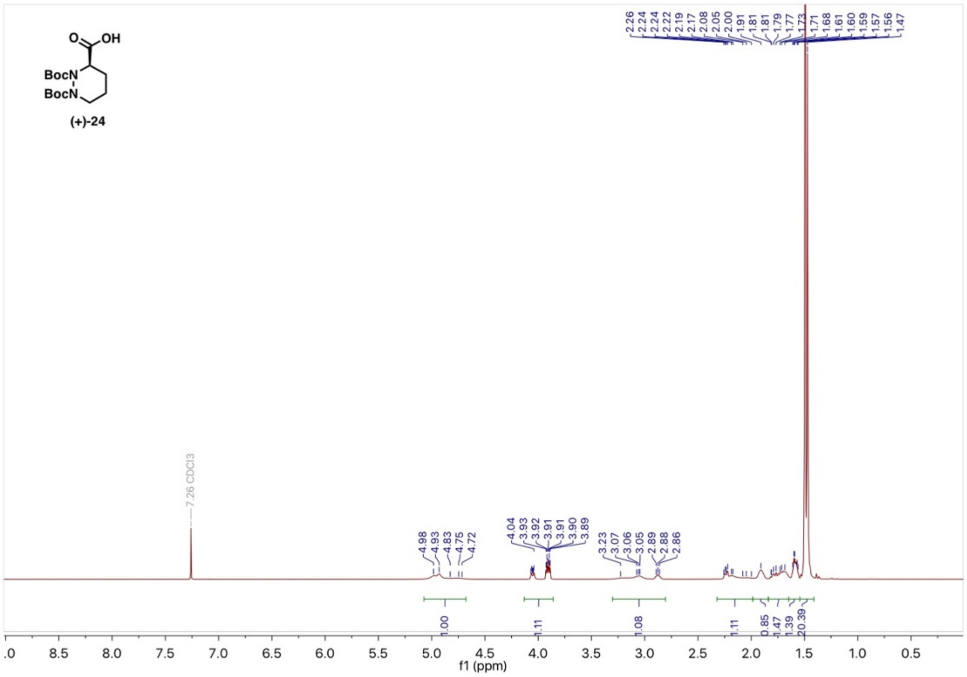

^{1}H NMR (600 MHz, CDCl$_3$) of (+)-24

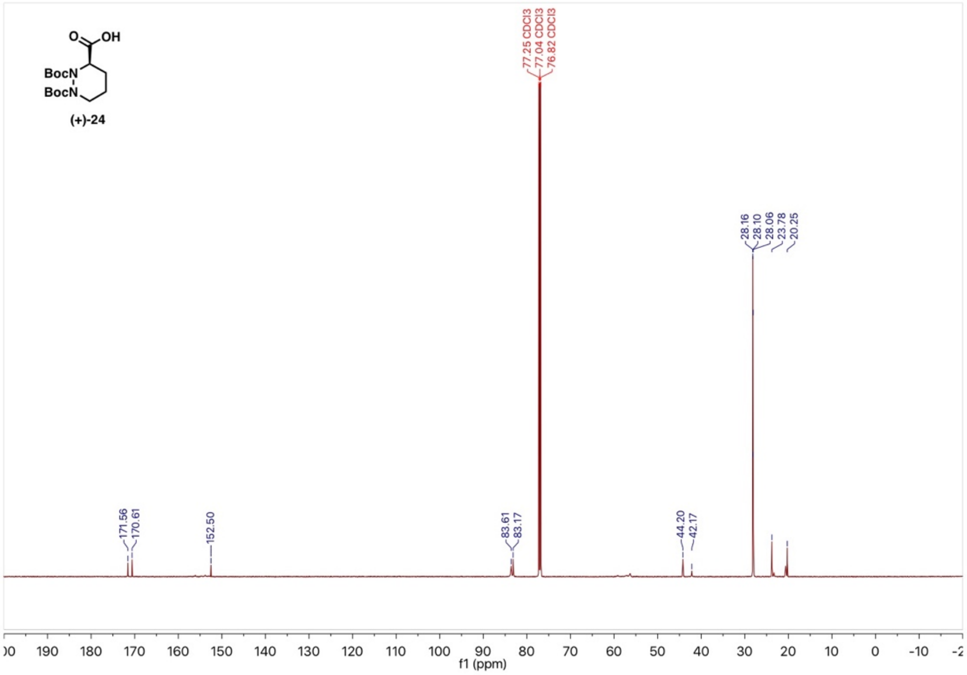

^{13}C NMR (151 MHz, CDCl$_3$) of (+)-24

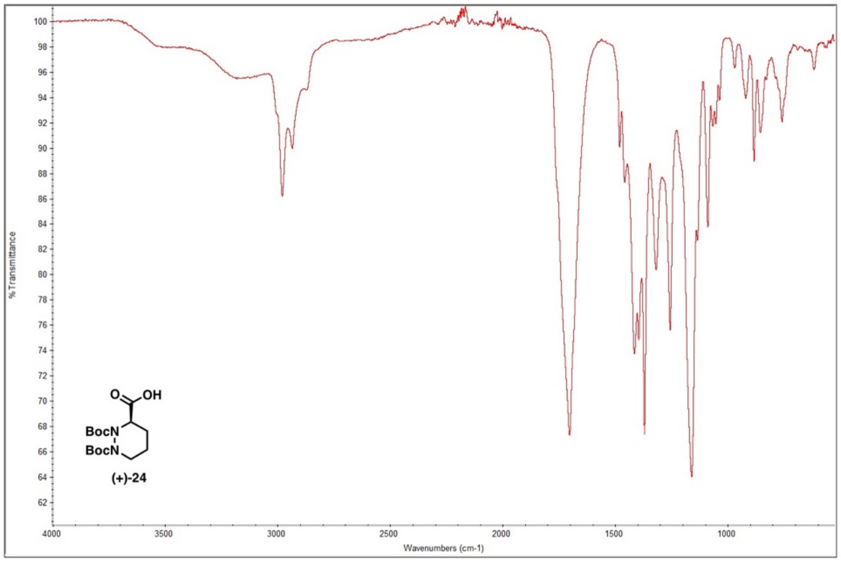

IR (neat) of (+)-24

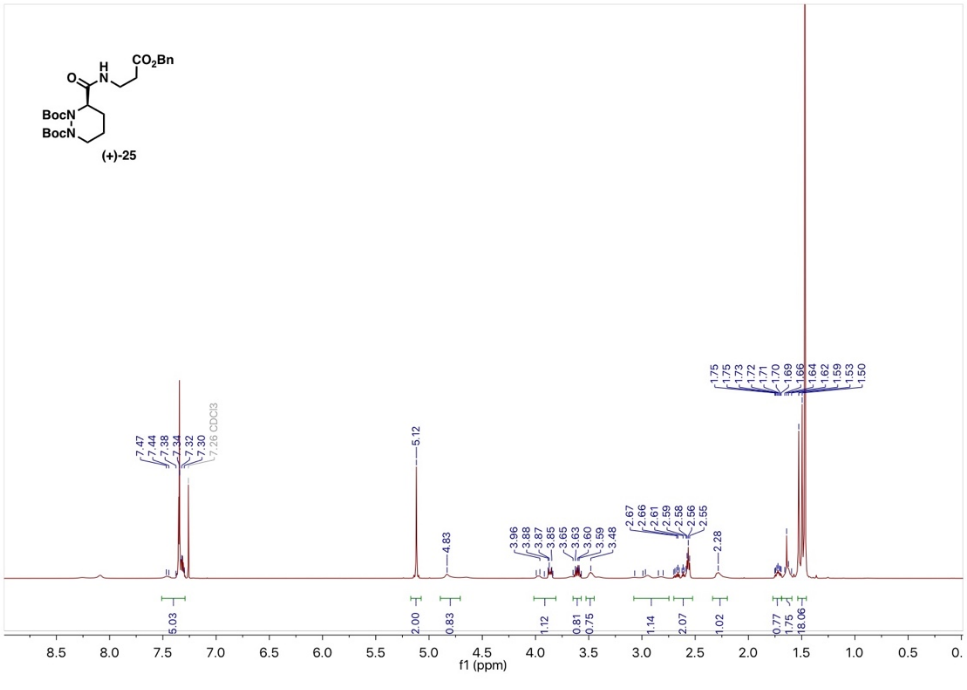

^{1}H NMR (600 MHz, $CDCl_3$) of (+)-25

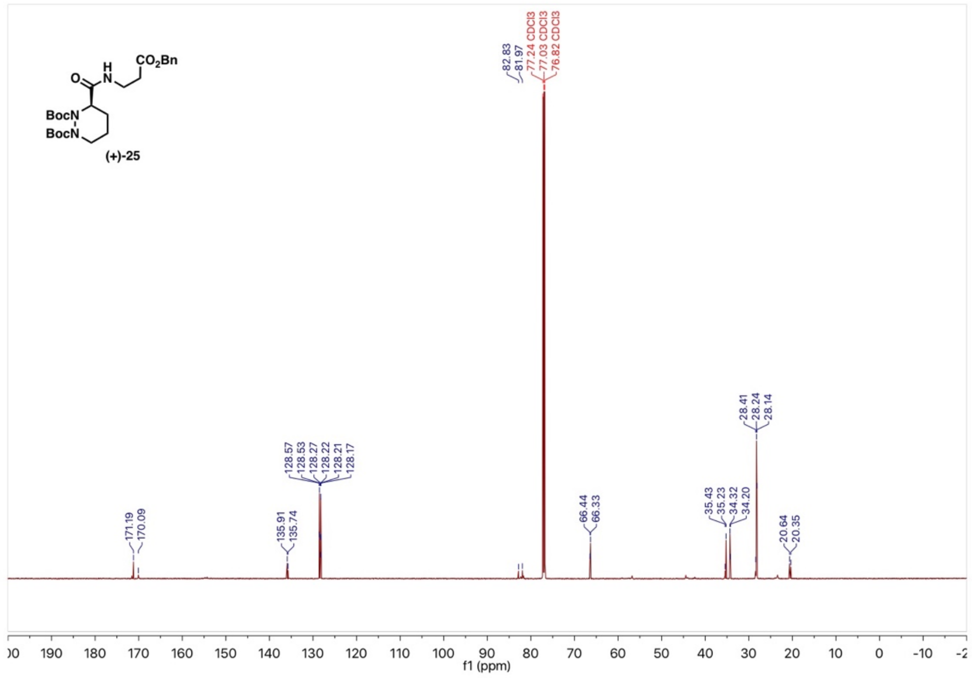

^{13}C NMR (151 MHz, $CDCl_3$) of (+)-25

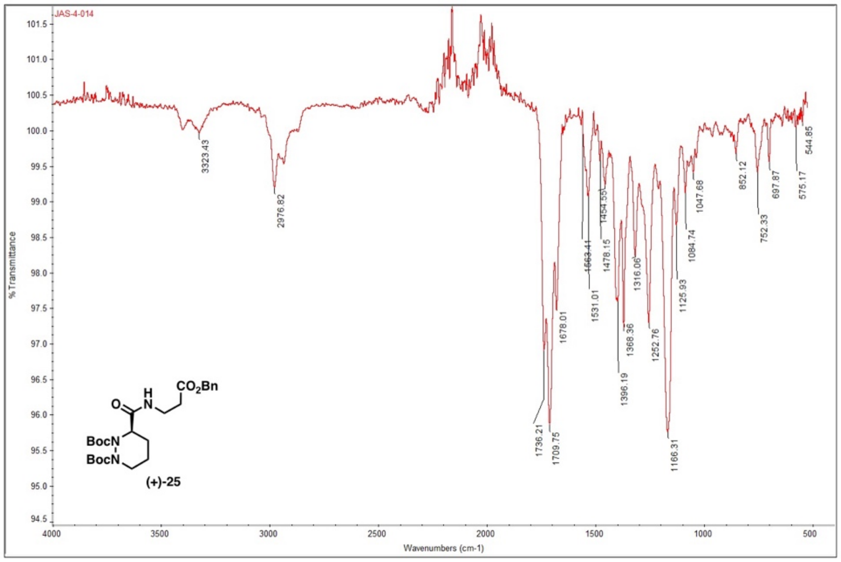

IR (neat) of (+)-25

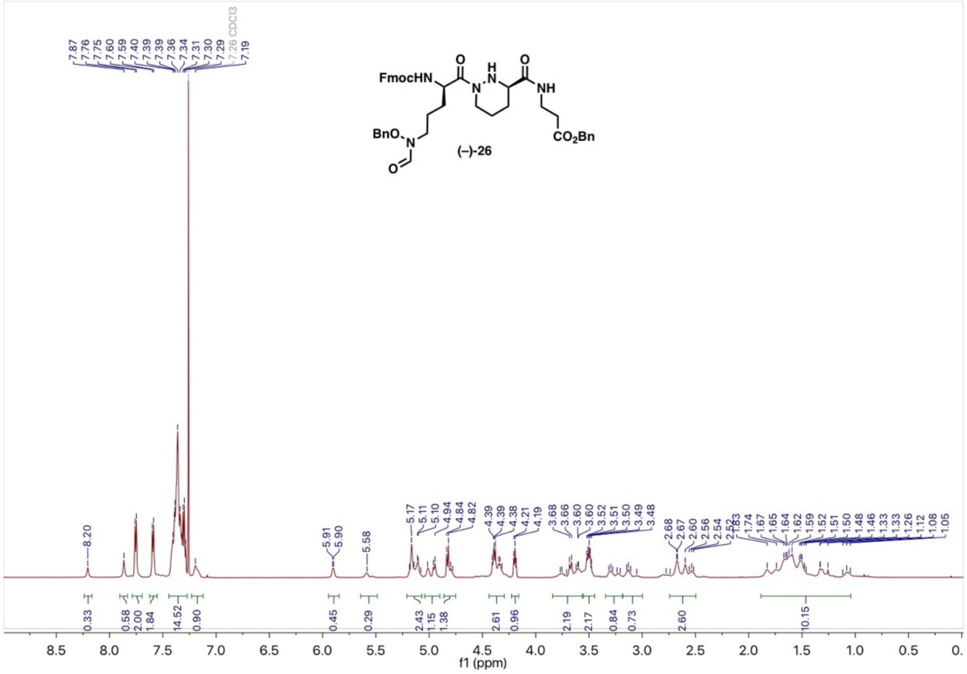

^{1}H NMR (600 MHz, CDCl3) of (–)-26

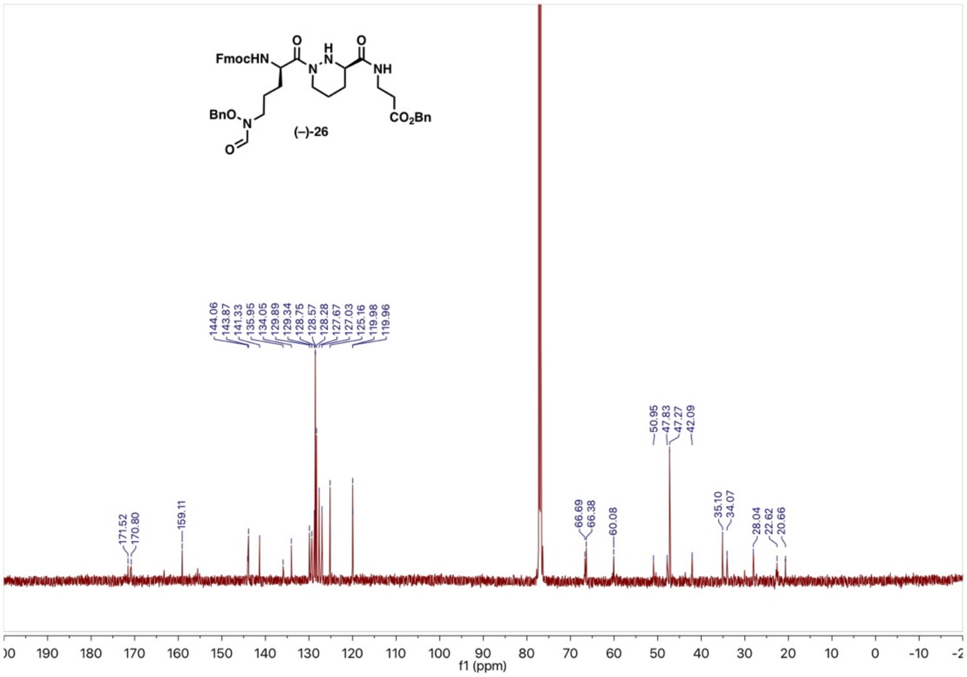

^{13}C NMR (151 MHz, CDCl3) of (–)-26

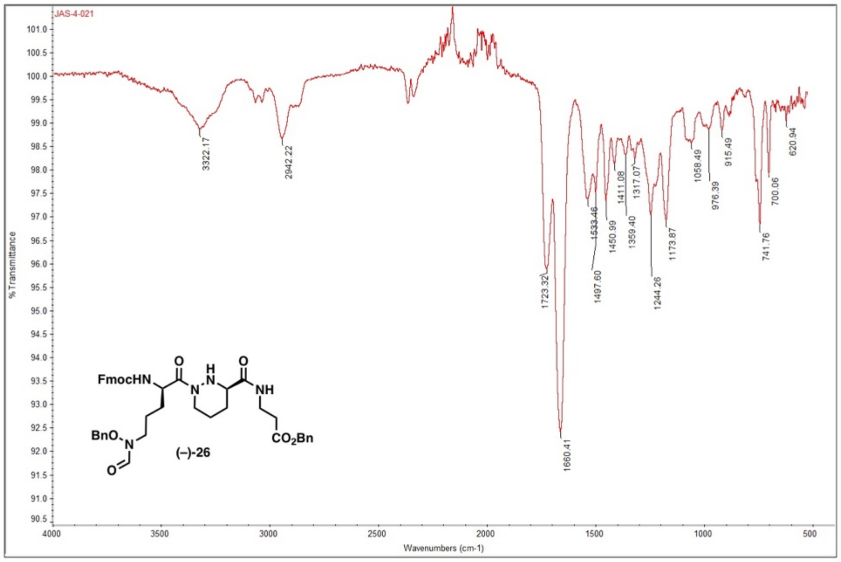

IR (neat) of (–)-26

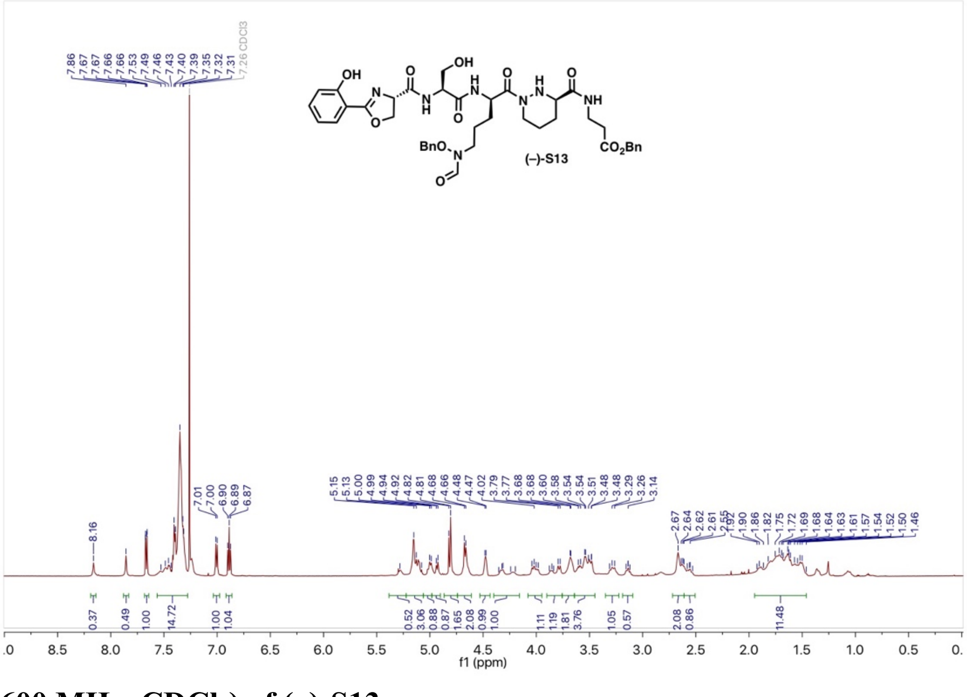

^{1}H NMR (600 MHz, $CDCl_3$) of (–)-S13

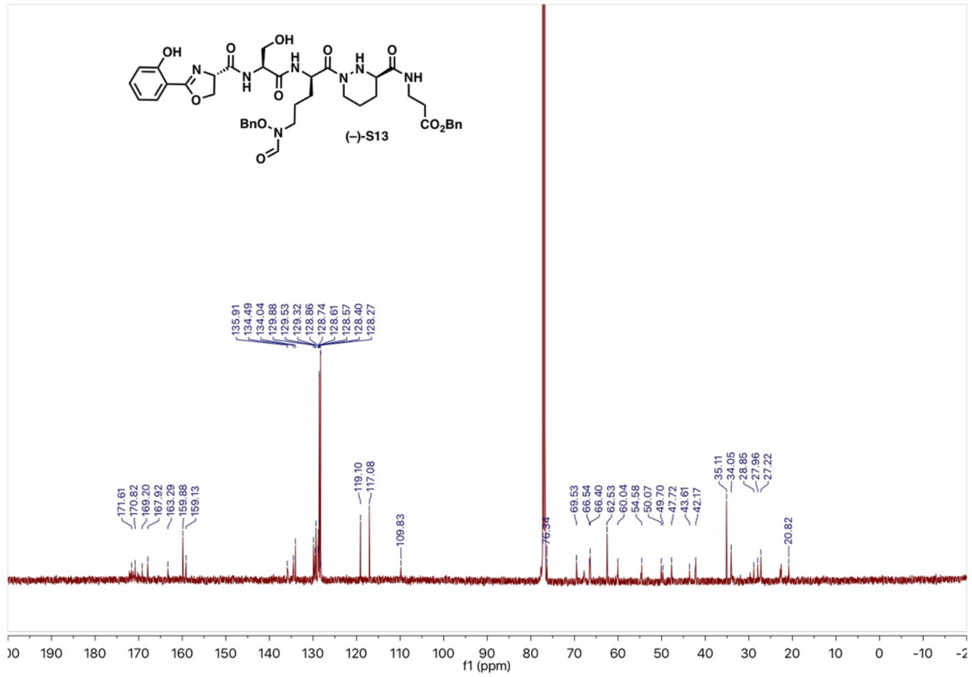

^{13}C NMR (151 MHz, CDCl$_3$) of (–)-S13

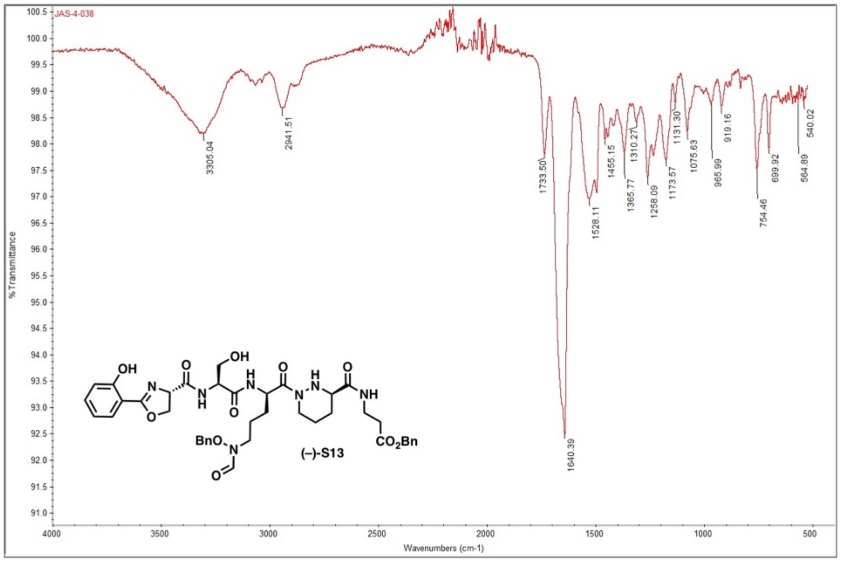

IR (neat) of (–)-S13

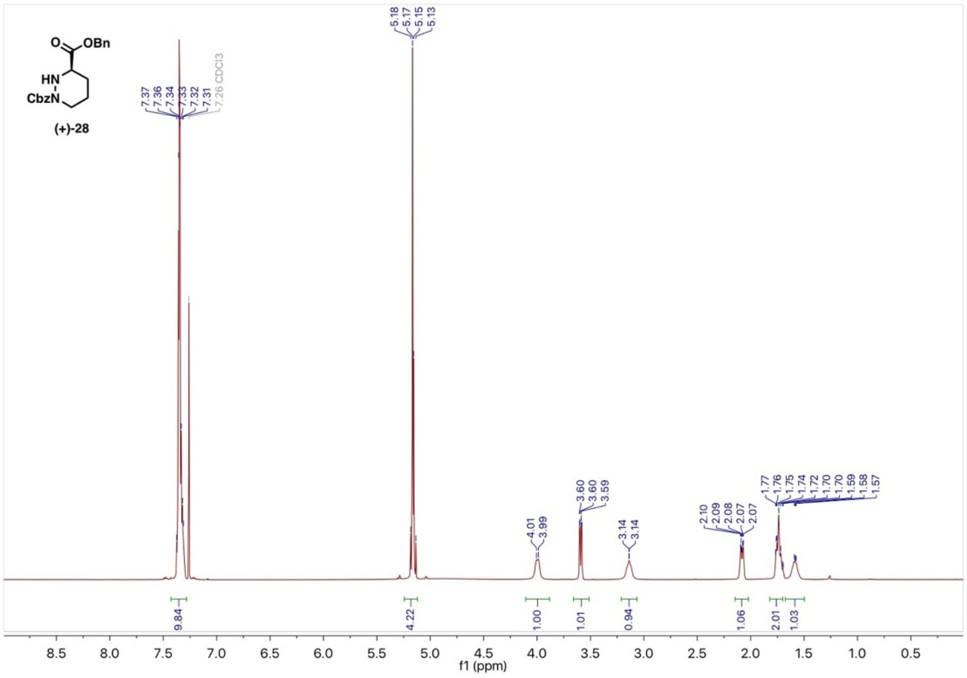

^{1}H NMR (600 MHz, CDCl$_3$) of (+)-28

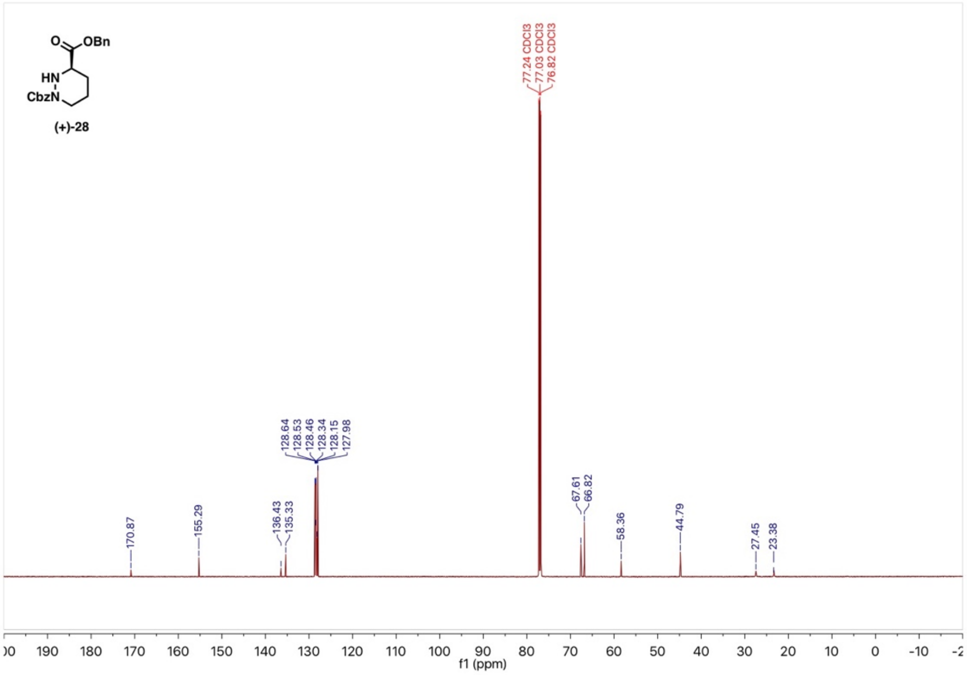

^{13}C NMR (151 MHz, CDCl$_3$) of (+)-28

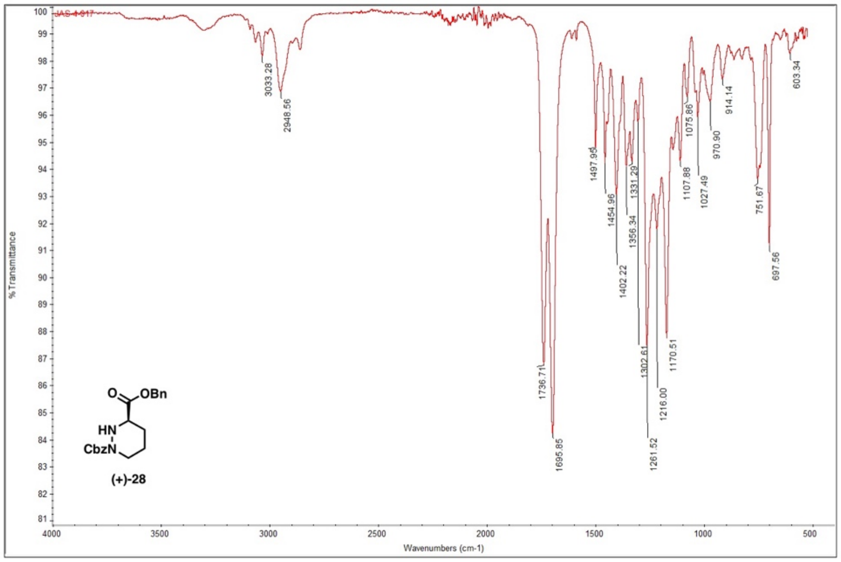

IR (neat) of (+)-28

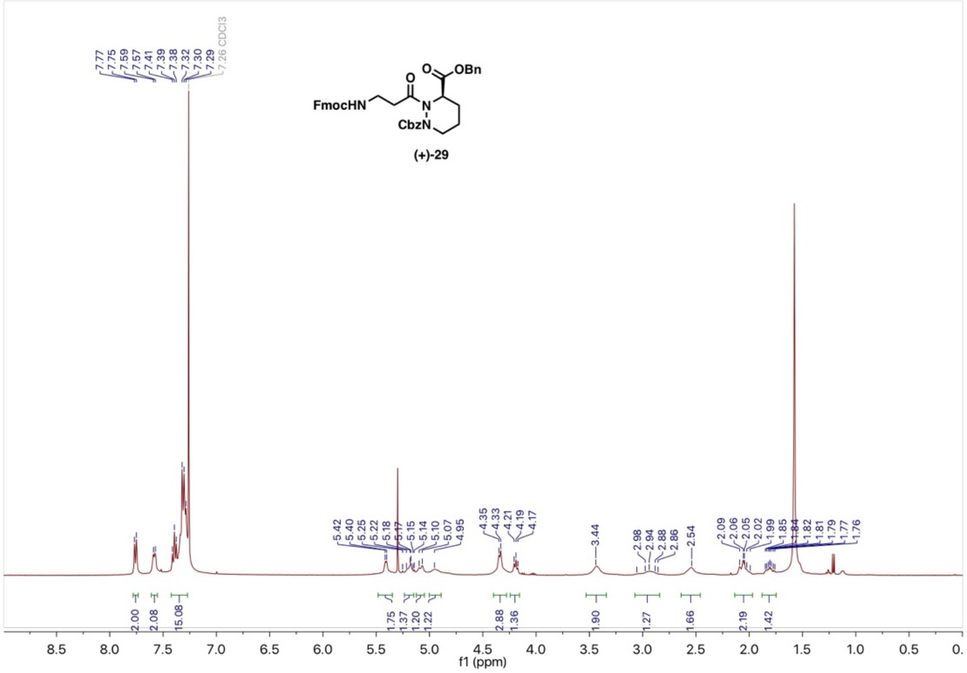

^{1}H NMR (600 MHz, CDCl$_3$) of (+)-29

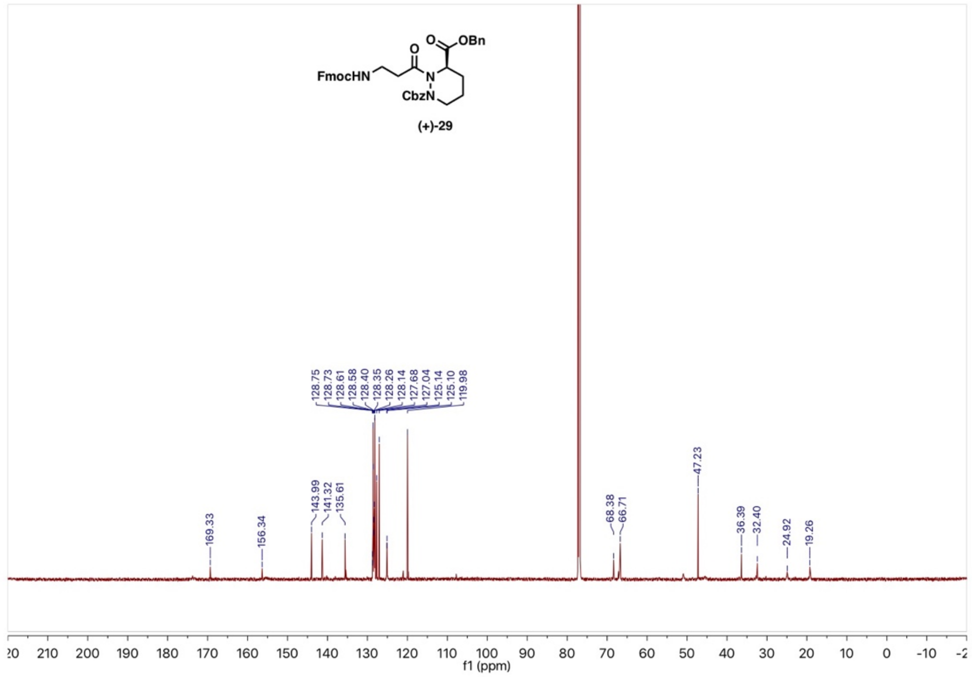

^{13}C NMR (151 MHz, $CDCl_3$) of (+)-29

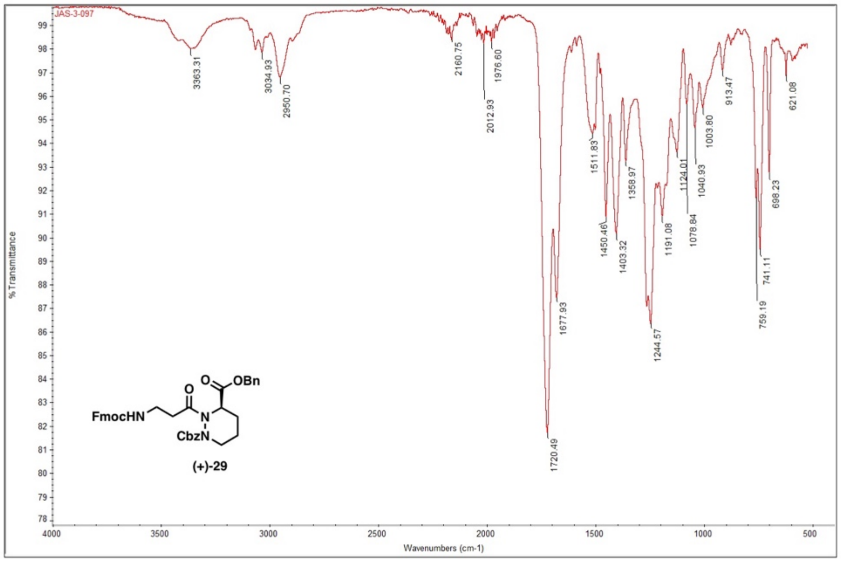

IR (neat) of (+)-29

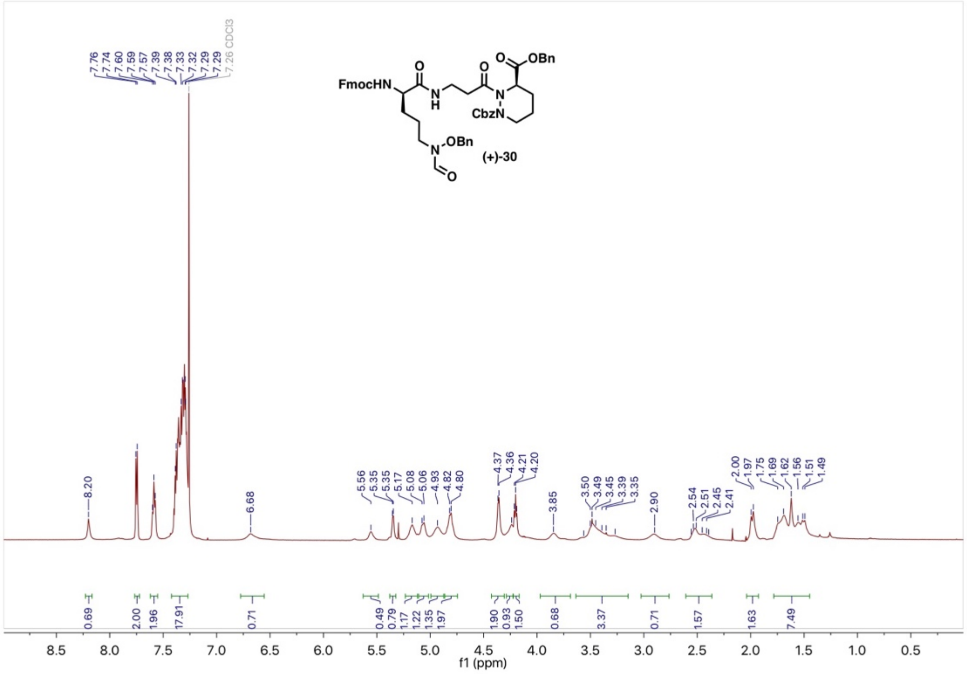

^{1}H NMR (600 MHz, $CDCl_3$) of (+)-30

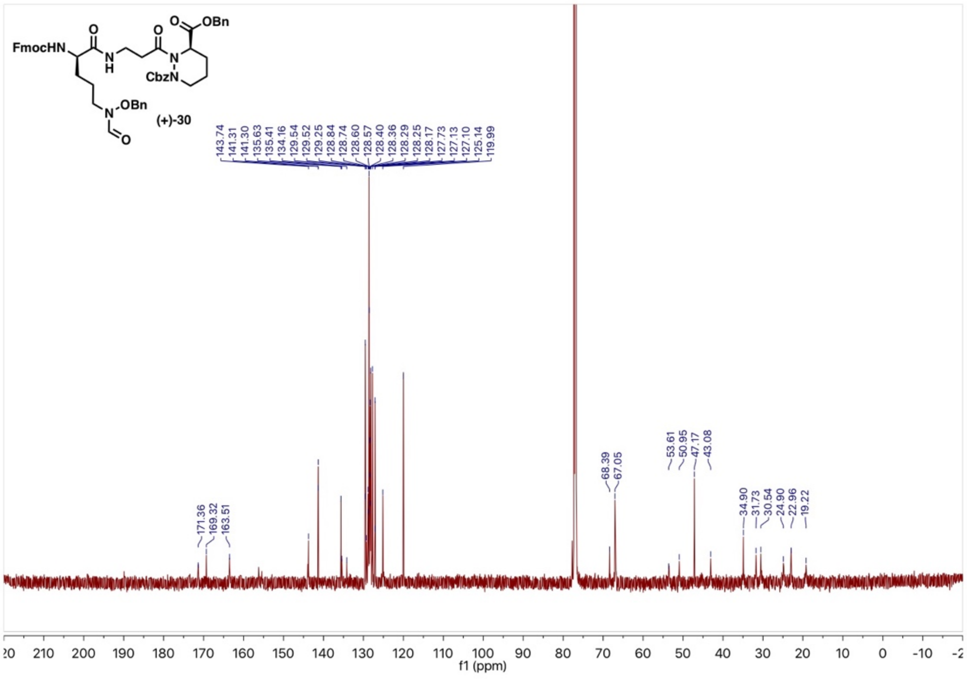

^{13}C NMR (151 MHz, $CDCl_3$) of (+)-30

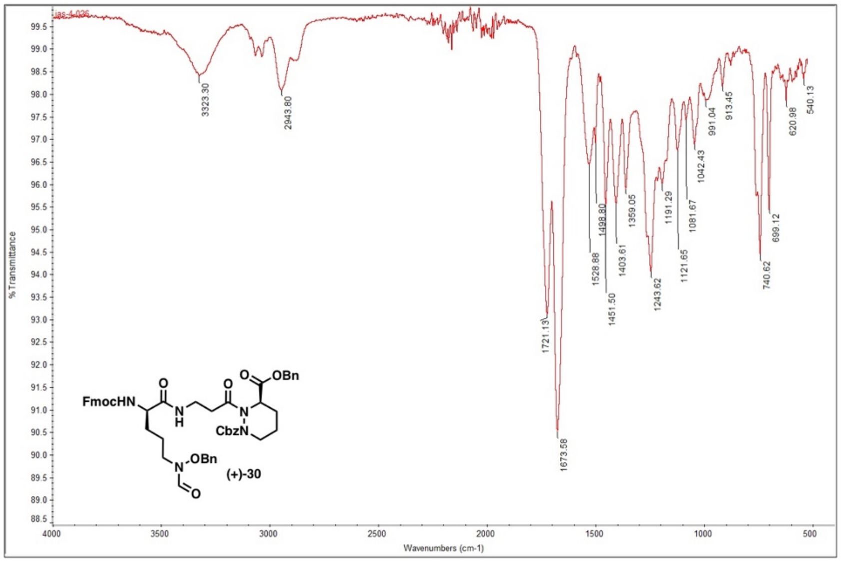

IR (neat) of (+)-30

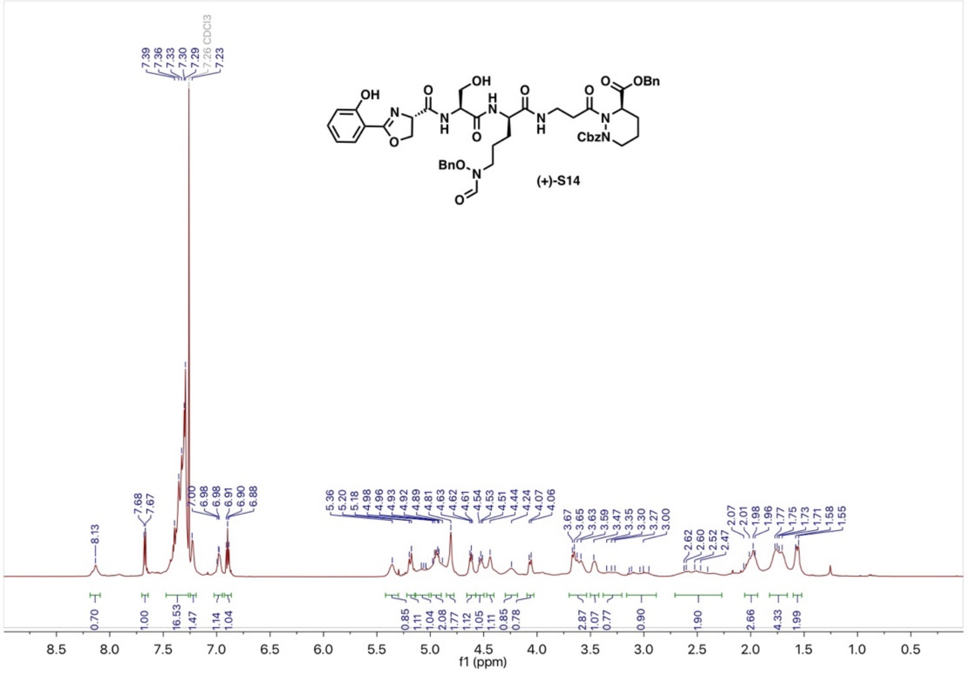

^{1}H NMR (600 MHz, CDCl$_3$) of (+)-S14

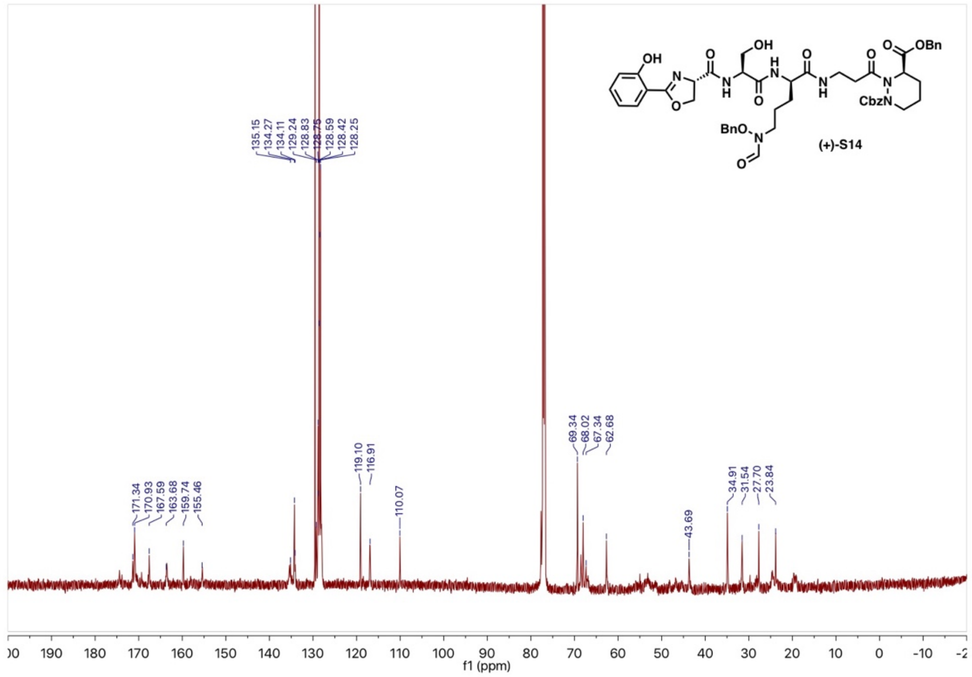

^{13}C NMR (151 MHz, $CDCl_3$) of (+)-S14

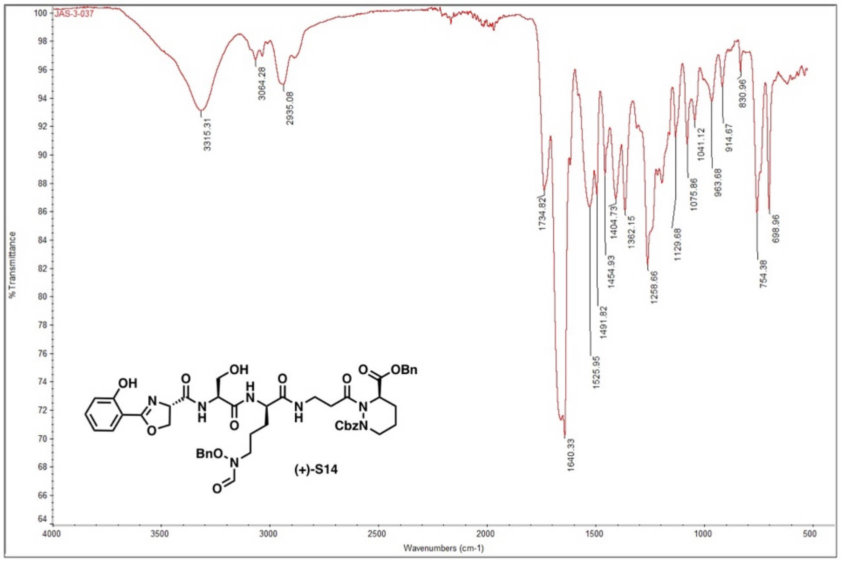

IR (neat) of (+)-S14
